# Activity-Based Funding of Hospitals and Its Impact on Mortality, Readmission, Discharge Destination, Severity of Illness, and Volume of Care: A Systematic Review and Meta-Analysis

**DOI:** 10.1371/journal.pone.0109975

**Published:** 2014-10-27

**Authors:** Karen S. Palmer, Thomas Agoritsas, Danielle Martin, Taryn Scott, Sohail M. Mulla, Ashley P. Miller, Arnav Agarwal, Andrew Bresnahan, Afeez Abiola Hazzan, Rebecca A. Jeffery, Arnaud Merglen, Ahmed Negm, Reed A. Siemieniuk, Neera Bhatnagar, Irfan A. Dhalla, John N. Lavis, John J. You, Stephen J. Duckett, Gordon H. Guyatt

**Affiliations:** 1 Faculty of Science Simon Fraser University, Burnaby, British Columbia, Canada; 2 Department of Clinical Epidemiology and Biostatistics, Faculty of Health Sciences, McMaster University, Hamilton, Ontario, Canada; 3 Divisions of General Internal Medicine and Clinical Epidemiology, University Hospitals of Geneva and Faculty of Medicine, University of Geneva, Geneva, Switzerland; 4 Department of Family and Community Medicine, Department of Health Policy Management and Evaluation, University of Toronto and Medical Affairs and Health Systems Solutions at Women's College Hospital, Toronto, Ontario, Canada; 5 Memorial University of Newfoundland, St. John's, Newfoundland, Canada; 6 Michael G. DeGroote School of Medicine, McMaster University, Hamilton, Ontario, Canada; 7 Faculty of Medicine, Dalhousie University, Halifax, Nova Scotia, Canada; 8 Paediatric Outcome Research Team, The Hospital for Sick Children, Toronto, Ontario, Canada and Division of General Pediatrics, University Hospitals of Geneva and Faculty of Medicine, University of Geneva, Geneva, Switzerland; 9 Rehabilitation School, McMaster University, Hamilton, Ontario, Canada; 10 Department of General Internal Medicine, Sunnybrook Health Sciences Centre, Toronto, Ontario, Canada; 11 Health Sciences Library, McMaster University, Hamilton, Ontario, Canada; 12 St. Michael's Hospital, Department of Medicine and Institute of Health Policy Management and Evaluation, University of Toronto, Toronto, Ontario, Canada; 13 McMaster Health Forum, Centre for Health Economics and Policy Analysis, Department of Political Science, McMaster University, Hamilton, Ontario, Canada and Department of Global Health and Population, Harvard School of Public Health, Boston, Massachusetts, United States of America; 14 Grattan Institute, Carlton, Victoria, Australia; School of Population Health, The University of Queensland, Australia

## Abstract

**Background:**

Activity-based funding (ABF) of hospitals is a policy intervention intended to re-shape incentives across health systems through the use of diagnosis-related groups. Many countries are adopting or actively promoting ABF. We assessed the effect of ABF on key measures potentially affecting patients and health care systems: mortality (acute and post-acute care); readmission rates; discharge rate to post-acute care following hospitalization; severity of illness; volume of care.

**Methods:**

We undertook a systematic review and meta-analysis of the worldwide evidence produced since 1980. We included all studies reporting original quantitative data comparing the impact of ABF versus alternative funding systems in acute care settings, regardless of language. We searched 9 electronic databases (OVID MEDLINE, EMBASE, OVID Healthstar, CINAHL, Cochrane CENTRAL, Health Technology Assessment, NHS Economic Evaluation Database, Cochrane Database of Systematic Reviews, and Business Source), hand-searched reference lists, and consulted with experts. Paired reviewers independently screened for eligibility, abstracted data, and assessed study credibility according to a pre-defined scoring system, resolving conflicts by discussion or adjudication.

**Results:**

Of 16,565 unique citations, 50 US studies and 15 studies from 9 other countries proved eligible (i.e. Australia, Austria, England, Germany, Israel, Italy, Scotland, Sweden, Switzerland). We found consistent and robust differences between ABF and no-ABF in discharge to post-acute care, showing a 24% increase with ABF (pooled relative risk  = 1.24, 95% CI 1.18–1.31). Results also suggested a possible increase in readmission with ABF, and an apparent increase in severity of illness, perhaps reflecting differences in diagnostic coding. Although we found no consistent, systematic differences in mortality rates and volume of care, results varied widely across studies, some suggesting appreciable benefits from ABF, and others suggesting deleterious consequences.

**Conclusions:**

Transitioning to ABF is associated with important policy- and clinically-relevant changes. Evidence suggests substantial increases in admissions to post-acute care following hospitalization, with implications for system capacity and equitable access to care. High variability in results of other outcomes leaves the impact in particular settings uncertain, and may not allow a jurisdiction to predict if ABF would be harmless. Decision-makers considering ABF should plan for likely increases in post-acute care admissions, and be aware of the large uncertainty around impacts on other critical outcomes.

## Introduction

As health care systems evolve, policymakers strive to design approaches to hospital funding that simultaneously boost efficiency, increase budget transparency to promote accountability [1], and expand volume of activity [2], all while maintaining quality of care [3], and assuring equitable access to hospital services [4].

Activity-based funding (ABF) is an alternative to other hospital funding mechanisms, such as negotiated funding through global budgets or block grants, per diem payments, or retrospective cost-based reimbursement. Increasingly popular, ABF is a significant policy intervention intended to re-shape incentives across health care systems [5].

Under ABF, hospitals receive a fixed amount for each episode of care delivered to each patient, regardless of length of stay or actual resources used (sometimes with refinements for outliers). The funding schedule is prospectively determined based on clinically meaningful diagnosis-based “bundles” of services within which patients can be expected to consume similar amounts of resources. The funding allocation for these bundles is intended to account for the anticipated complexity, type, volume, and intensity of care ordinarily provided to patients admitted with particular diagnoses. A variety of cost accounting systems underpin the processes used to set a prospective price for each bundle of services [6]. The price of each bundle coupled with the volume of bundles provided determines all or part of the facility's budget.

ABF was first developed in the United States (US) in response to rising health care costs coupled with economic stagnation, which together stimulated a radical restructuring of Medicare funding [7]. Beginning in 1983, the US implemented ABF based on Diagnosis-Related Groups (DRGs) to fund acute hospital care for Medicare beneficiaries [8]. Since then, other countries have adopted variants of this episode- or service-related bundling approach as the basis for their hospital funding.

Internationally, ABF is known by a variety of terms — often confused in translation leading to a lack of clarity in the literature — including Prospective Payment System (PPS in the US); Payment-by-Results (PbR in the English National Health Service); Fallpauschalen system/vergütung (in Germany); Innsatsstyrt finansiering (ISF in Norway); Forfaits par cas/leistungsbezogene Fallpauschale (in Switzerland); case-mix funding; volume-based funding; and service-based funding [9]. In this paper, we use the term ABF. All ABF funding systems are based on DRG-like grouping algorithms (such as AR-DRGs “Australian-refined”; case mix groups (CMG+) in Canada; health-resource groups (HRGs in England); Groupe Homogène des Malades (GHM) in France; G-DRGS in Germany; Diagnose Behandeling Combinatie (DBC in the Netherlands); Nord-DRGs in the Nordic countries; SwissDRG in Switzerland) [9]–[11]. ABF implementation differs in each jurisdiction where it is adopted, with many choices inherent in the precise design and accompanying rules.

Possible benefits of ABF may include reduced hospital costs [12], [13], greater funding and spending transparency [4], [9], improved efficiency [3], [14]; reduced length of stay [15]; and shorter wait times [6]. Enthusiasts also claim that a culture change — by which patients are seen not as cost drivers but as “revenue generators” [3], [5] — is a natural result of ABF. Possible detrimental consequences of ABF include increased mortality [16], increased readmission to hospital [17], rapid discharge of sick patients into ill-prepared community settings [18], an incentive to “upcode” diagnoses and thus “game” the system [19], [20], focus on “profitable” over “unprofitable” patients and procedures compromising equitable access to care [21], [22], and increased administrative and/or overall costs to the health care system [23], [24].

Because it is natural for advocates and opponents to selectively cite evidence supporting their positions, to better inform decision-makers we undertook a systematic review of the worldwide evidence bearing on how ABF affects quality of care, access to care, equity, hospital and total health care system costs, length of stay, and efficiency. For this report, we focus on how ABF affects six key measures, each with the potential to affect patients and health care system capacity: acute care mortality (AC mortality); and post-acute care mortality (PAC mortality); readmission rates; discharge destination measured by discharge to post-acute care (PAC) following hospitalization; severity of illness; and volume of care. We also explored whether the impact of ABF on these six key measures varied according to either contextual factors of the health care systems, or the research methods used to assess the impact of ABF, by examining six pre-specified sub-groups: study location; study design; time elapsed after ABF implementation; adjustment for confounders; credibility of the methods; time of outcome assessment.

## Methods

Our protocol (available upon request) provides a full description of our methods; a summary follows.

### Eligibility Criteria

We included all studies providing original analyses of quantitative data that compared the impact of ABF versus alternative funding systems implemented in acute care settings (hospitals and non-hospital medical or surgical facilities) published in any language. We included before-after studies in single jurisdictions (before ABF vs. after ABF implementation), parallel group studies in multiple jurisdictions (jurisdictions without ABF vs. jurisdiction with ABF), or a combination of both designs (e.g. difference-in-difference analyses, time-series). We excluded editorials, letters, news, and notes as defined by the databases. For this report, we included only studies reporting data on at least one of our primary outcomes: AC mortality, PAC mortality, readmission rates, and discharge destination. For eligible studies that reported at least one of these primary outcomes, we also abstracted data on severity of illness and volume of care.

We excluded studies that did not include a comparator group in which ABF was not implemented. We further excluded studies that focused only on activity-based cost accounting systems; on differences in capital costs/acquisitions; on the intervention of pay-for-performance (a financial incentive for attaining targeted service goals [25]); and on systems based on a flat rate per diem or for another period of time, such as the Japanese Diagnosis Procedure Combination system, Chinese Hainan system, and Medicare Resource Utilization Groups.

### Outcomes

*Acute Care Mortality (AC Mortality)* was defined as death rate per population per common time period in the ABF and no ABF comparator, starting at admission to acute care or at surgery. We excluded mortality measured only in-hospital or only from discharge since the potential influence of ABF may have been confounded by differences in length of stay in ABF and no ABF periods.

*Post-Acute Care Mortality (PAC Mortality)* was defined similarly to acute care mortality, except that mortality was measured starting from admission to PAC following a stay in acute care.

*Hospital Readmission* was defined as readmission rates per population per common time period (preferentially at 30 days) in the ABF and no ABF comparator.

*Discharge Destination* was defined as the proportion of patients discharged alive from acute care to PAC, rather than to home. PAC included: intermediate care facilities, nursing homes, PAC facilities designated under the US Centers for Medicare and Medicaid (i.e. skilled nursing facilities; in-patient rehabilitation facilities; long-term care hospitals; home health agencies), and similar facilities in other countries. If data were reported for different PAC destinations, we aggregated them for our analysis.

*Changes in the distributions of reported severity of illness* included differences in measures such as case mix, diagnostic codes, DRG points, or number of patients with comorbidities.

*Changes in volume of care* included differences in measures such as the number of patients treated or admitted or number of procedures or tests performed.

### Search Strategy

Our search was limited to articles published from 1980 through 2012. We worked with a health information specialist to develop search terms; identified subject headings, terms, and keywords from key articles; and designed search strategies for ABF and related terms, such as DRGs and PPS, in combination with terms related to hospital and facility costs, and patient care. Studies were identified through bibliographic database searches of OVID MEDLINE, EMBASE, OVID Healthstar, CINAHL, Cochrane Central Register of Controlled Trials (CENTRAL), Health Technology Assessment, NHS Economic Evaluation Database, Cochrane Database of Systematic Reviews, and Business Source Complete. In each database, we undertook an iterative process to customize and refine search strategies. Appendix 1 (see File S1) presents a sample of our electronic search strategies.

In addition to electronic databases, we hand-searched reference lists of eligible studies and those of previously published reviews, books, websites, policy papers, personal files, and consulted with experts.

### Study Selection

Using web-based systematic review software (DistillerSR) paired reviewers independently conducted screening; first title and abstracts, and then full texts of articles in which titles and abstracts appeared potentially eligible. We pre-defined screening rules, and reviewers completed calibration exercises prior to reviewing full texts.

### Data Abstraction

Adhering to pre-defined abstraction rules, paired reviewers independently abstracted the following data from each article using standardized data abstraction forms custom built in DistillerSR and adapted to each study design: country and year of ABF implementation, data source, sampling methods, study population (type and number of patients and institutions), outcomes assessed, and results.

When studies reported data for several time periods before and after ABF implementation, we abstracted data from three time periods: 1) *no ABF*, defined as the first data point prior to ABF implementation within three years of implementation; 2) *early ABF*, defined as the first data point after ABF implementation irrespective of the proportion of ABF; 3) *late ABF*, defined as the data point farthest from ABF implementation up to 5 years following implementation, irrespective of the proportion of funding under ABF.

Whenever possible, we abstracted data to enable a meta-analysis for our primary outcomes. When data were not poolable, two abstractors independently abstracted narrative summaries including outcome direction, magnitude, and statistical significance.

### Credibility Assessment

Pairs of reviewers independently assessed the credibility of individual studies according to a pre-defined scoring system documenting three criteria: 1) quality of data sources; 2) number of eligible outcomes simultaneously examined in the study; and 3) comprehensiveness and appropriateness of the adjustment for potential confounders. The maximum credibility score was 6 and the minimum 0, and higher credibility was *a priori* categorized as a score of 4 or more.

Eligibility, data abstraction, and credibility assessment conflicts between the two independent reviewers in each pair were resolved by discussion or if necessary with adjudication by a senior team member.

### Heterogeneity and Hypothesized Sub-Group Effects

We hypothesized *a priori* that variability in results across studies might be due to the following: study location (US vs. international); study design (before-after vs. parallel-controlled study); time after ABF implementation (2 years or less  =  early ABF vs. more than 2 years  =  late ABF); adjustment for confounders (adjusted vs. unadjusted); credibility (higher vs. lower); and time of assessment of mortality or readmission (within 30 days vs. more than 30 days).

The comparison US vs. International may also be considered as an indirect comparison of: i) older studies (US) vs. more recent studies (International); ii) mainly cost-based reimbursement before ABF (US) vs. non-cost-based funding before ABF (International).

### Data analysis

We pre-specified a set of conditions necessary to pool reported data in a meta-analysis. Data had to be either detailed unadjusted frequencies in the ABF and no ABF group, or effect estimates with: 1) a measure of variation (e.g. standard deviation, variance); or 2) sufficient statistical information (e.g. standard error, confidence intervals, exact *p*-values) along with exact number of patients at risk (Appendix 2, see File S1).

For studies that reported poolable data, we conducted a quantitative meta-analysis of 4 dichotomous outcomes – AC mortality, PAC mortality, hospital readmission, discharge to PAC – using random effects models. When both adjusted and unadjusted data were reported, we used adjusted data. The adjusted estimates determined the type of measure used in the pooling: risk ratios (RR) for discharge destination and readmission, and odds ratios (OR) for mortality data. When only hazard ratios (HR) or incidence rate ratios were reported we converted them to RR.

We assessed heterogeneity in results using the Cochrane's Q test [26] and calculated the I^2^ [27]. We conducted all pre-specified sub-group analyses performing tests for interaction, with a *p* value <0.05 considered as statistically significant. When seven or more studies were available we addressed publication bias graphically using funnel plots.

For non-poolable data we recorded direction of outcome with ABF vs. no ABF (increase, decrease, mixed, no difference); magnitude (relative difference ≥5%, ≥1% to <5%, <1%, indeterminate or mixed); and statistical significance (p>.05, p≤.05–.02, p≤.01–.002, p≤.001, p-value not reported), and considered possible differences in effect of study location (US vs. international) and study credibility (higher vs. lower).

## Results

**Figure 1:**
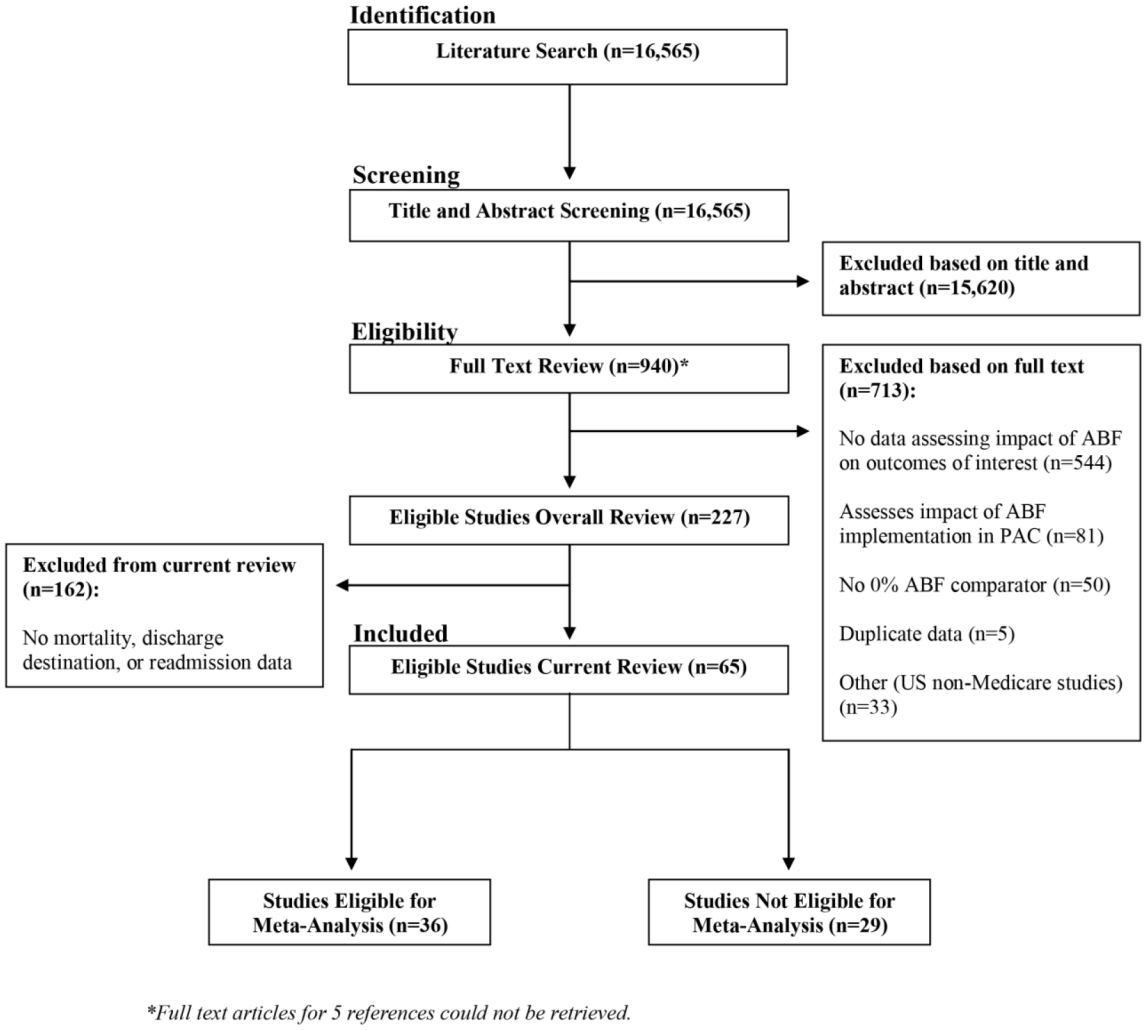
Prisma flow diagram.

Figure 1 presents the flow of acquisition of eligible studies. Of 16,565 unique citations, 227 reported data from 64 countries addressing the impact of ABF on quality or access to care, equity, cost, or efficiency. Of these, 65 studies [16]–[18], [28]–[89] reporting data from 10 countries — 50 US studies and 15 studies from other countries (i.e. Australia, Austria, England, Germany, Israel, Italy, Scotland, Sweden, Switzerland)— provided data on at least one of our primary outcomes. The weighted kappa coefficient for overall agreement across reviewer pairs screening full texts ranged from 0.72 to 0.86.

Table 1 presents the location, design, and sampling of the eligible studies, of which 36 provided data that contributed to the pooled analysis for at least one variable. The median number of outcomes reported per study was 3.0 (inter-quartile range 2.0 to 3.0).

**Table 1:** Study Characteristics (Pooled and Non-Pooled).

Variable	Frequency N = 65		
	Pooled studies (n = 36)	Non-Pooled studies (n = 29)	Total (N = 65)
**Study Demographics**			
Type of Publication:			
*Full-text journal article*	35	29	64
*Government report*	1	0	1
*Non-government report*	0	0	0
Geographic Location of Study:			
*USA*	28	22	50
*Australia*	2	1	3
*Austria*	0	1	1
*England*	0	1	1
*Germany*	1	1	2
*Israel*	0	1	1
*Italy*	3	1	4
*Scotland*	0	1	1
*Sweden*	1	0	1
*Switzerland*	1	1	2
**Methods**			
Study Design			
*Before vs. After ABF (single jurisdiction)*	34	25	59
*Parallel groups (multiple jurisdictions)*	2	1	3
*Parallel groups & before/after*	0	3	3
Sampling Method			
*Random*	6	5	11
*Convenience*	20	16	36
*All eligible institutions in a jurisdiction*	10	9	19
Number of Patients Evaluated			
*Less than 1,000*	14	1	15
*1,000 to 10,000*	9	3	12
*Greater than 10,000*	7	7	14
Number of Institutions evaluated			
*Less than 5*	13	3	16
*5 to 50*	8	3	11
*Greater than 50*	6	15	21

*Note*: frequencies may not add to 65 as not all categories are mutually exclusive.

### Credibility of study results

Overall credibility of included studies was generally low, but varied across outcomes (Appendix 3, see File S1). The number of higher credibility studies ranged between 27% to 63% across studies with poolable outcomes and from 0% to 58% across studies with non-poolable outcomes.

The majority of studies did not provide satisfactory documentation of the quality of their data sources. From 64% to 80% performed only minimal or no adjustment of their results, and 14% to 20% performed a comprehensive adjustment.

### Main Findings

Table 2 summarizes the key results for pooled and non-pooled analyses across outcomes. Consistent and credible differences between ABF and no ABF were found only in discharge to PAC, which was greater with ABF (RR 1.24, 95% CI 1.18 to 1.31). Results also suggested an apparent increase in severity of illness with ABF, and possibly in readmission (with early ABF in the US and late ABF in other countries). In general, results were consistent between US studies and international studies, as well as between early and late implementation of ABF. Additional details of results by outcome follow.

**Table 2:** Summary of Findings of the Impact of ABF (Pooled and Non-Pooled).

	Pooled Analysis*	Non-Pooled Analysis*
**AC Mortality**	**No difference** (modest variability)	**Early ABF: Increase** (2 studies consistent)
		**Late ABF: No difference** (2 studies mixed)
**PAC Mortality**	**No difference** (modest variability)	**No difference** (1 study)
**Readmission**	**No difference** (high variability)	**Early ABF: Increase** (modest variability)
		**Late ABF: US studies: No difference** (high variability) **International: Increase** (2 studies mixed)
**Discharge to PAC**	**Overall increase** (modest variability) Possible subgroup effect: increase in US, no increase in international	**Increase** (modest variability)
**Severity of illness**	N/A	**Increase** (high variability)
**Volume of Care**	N/A	**No difference** (high variability)

*Note: In general, results were consistent between US studies and international studies, as well as between early and late implementation of ABF. We have specified occurrences where results credibly differed.

### AC Mortality

#### AC Mortality pooled results

**Figure 2:**
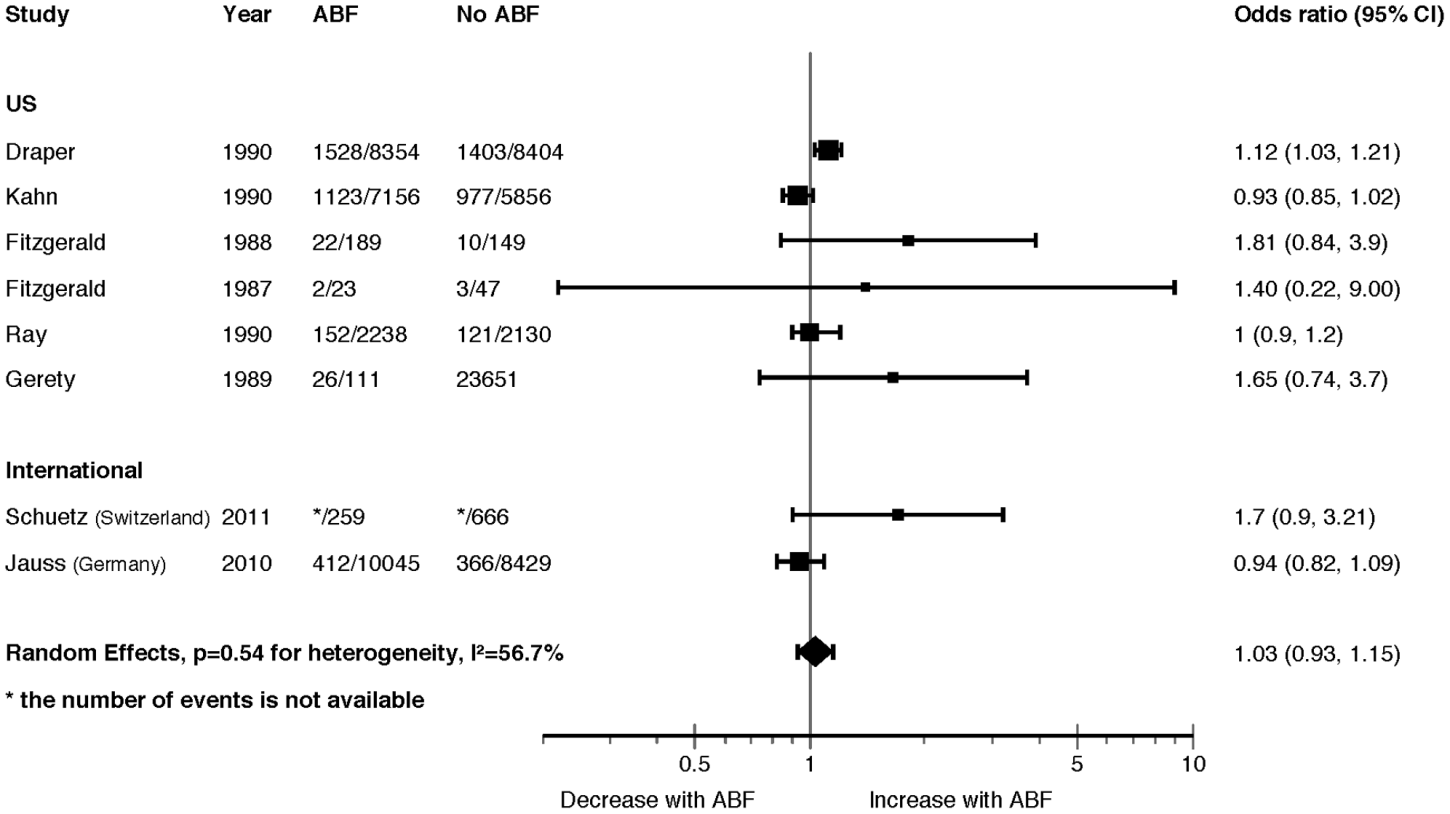
Acute Care Mortality Forest Plot.

Eight studies (six US; Switzerland; Germany) reporting poolable data demonstrated no difference in AC mortality between ABF and no ABF (OR = 1.03, 95% CI 0.93 to 1.15, p = 0.54, I^2^ = 57%) (Figure 2). Although the I^2^ was relatively large, the results of the large studies were very consistent in showing no difference in AC mortality. None of our pre-specified sub-group analyses explained heterogeneity in findings (Appendix 4, see File S1). The funnel plot did not suggest publication bias (Appendix 5, see File S1). Appendix 6 (see File S1) presents details of study descriptions.

#### AC Mortality non-pooled results (Early ABF and Late ABF)

Two studies (US; Scotland/England) reported that, early after ABF was implemented, mortality increased with small or indeterminate magnitude and without reporting p-values. One study (Scotland/England) reported no difference in mortality late after ABF was implemented. A second study (US) reported that mortality increased in some sub-groups and decreased in others without reporting p-values. Appendices 7, 8, 9 (see File S1) present study descriptions, main findings, and analyses.

#### AC Mortality Summary

The pooled analysis showed no impact of ABF on mortality; non-pooled results show a possible increase in mortality in the early stages of ABF implementation, and mixed results later.

### PAC Mortality

#### PAC Mortality results (pooled and non-pooled)

Three US studies reported poolable data with very wide confidence intervals including both substantial decrease and appreciable increase in PAC mortality with ABF (RR = 0.80, 95% CI 0.52 to1.24, p = 0.32, I^2^ = 34%). The single additional US study reporting non-poolable data, suggested no difference in mortality with ABF. Appendices 10, 11, 12, 13 (see File S1) present the forest plot, study descriptions, main findings, and analyses.

### Hospital Readmission

#### Readmission Pooled results

**Figure 3:**
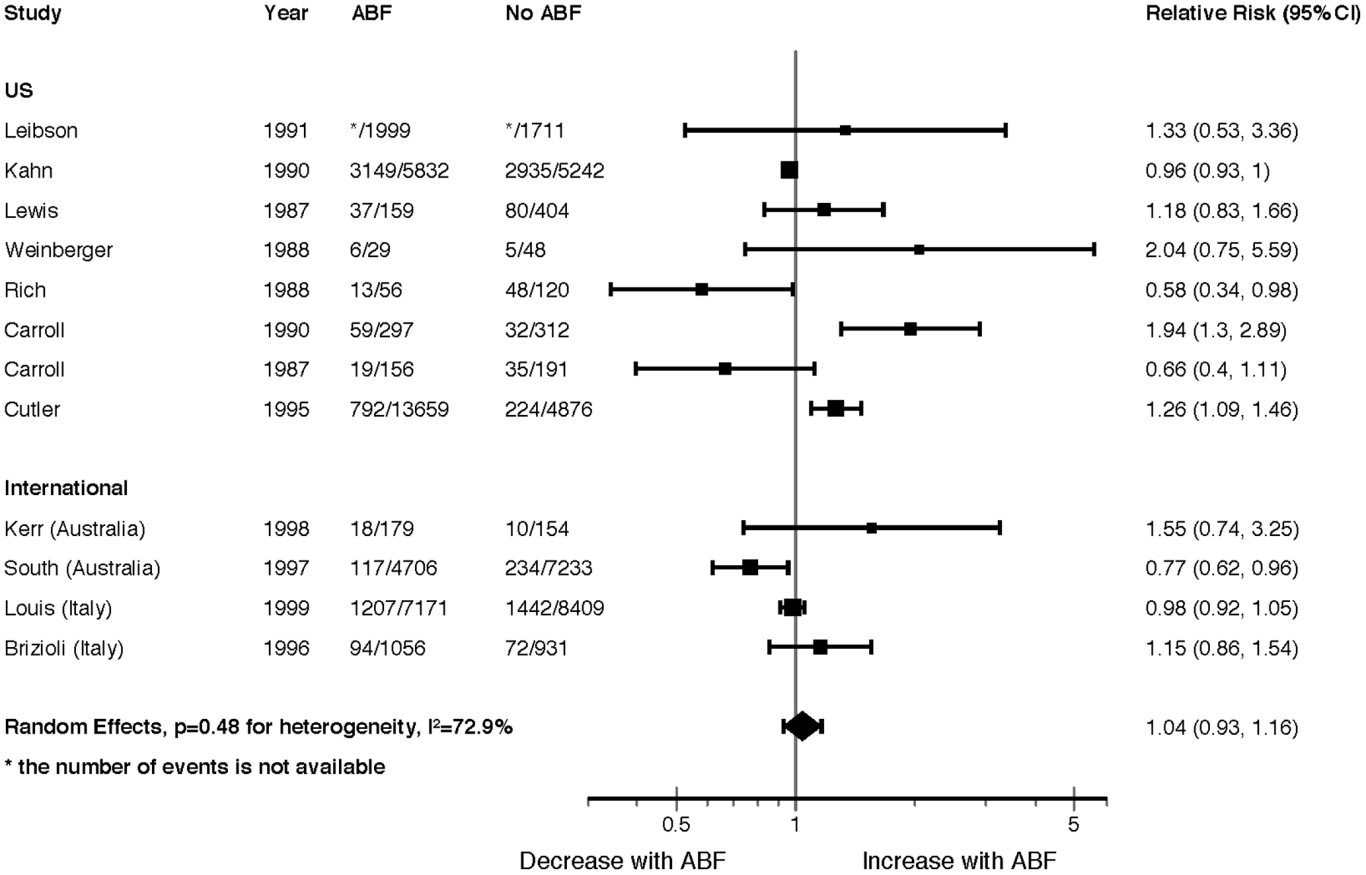
Readmission Forest Plot.

Twelve studies (eight US; two Australia; two Italy) reporting poolable data suggested no overall difference in readmissions (RR = 1.04, 95% CI 0.93 to 1.16, p = 0.48, I^2^ = 73%) (Figure 3). None of our pre-specified sub-group analyses explained the high variability in findings across studies, with some studies showing a significant increase [17], [32] and others a significant decrease [78], [83] in readmission rates with ABF (Appendix 14, see File S1). We found no suggestion of publication bias (Appendix 15, see File S1). Appendix 16 (see File S1) presents study descriptions.

Even though it presented data amenable to statistical analysis, we excluded one US study [49] with a RR of readmission greater than 20 from our primary analysis. We attributed the results, which we found not credible, to “unbundling” cataract surgeries. Whereas prior to ABF, patients requiring bilateral cataract surgery would have both cataracts done in a single admission, under ABF they were discharged after the first cataract procedure and readmitted for the second eye. A sensitivity analysis in which we included this study showed a marked increase in heterogeneity, and thus a considerably widened confidence interval in our random effects model (RR = 1.40, 95% CI 0.66 to 2.94, *p* = 0.38, I^2^  = 100%).

#### Readmission Non-poolable results (Early ABF and Late ABF)

Six studies (three US; Italy; Israel; England/Scotland) reported that early after ABF, readmissions increased, of which three reported a large increase. One US study reported a large decrease in readmissions. None of these studies reported p-values. Three studies (one US; Australia; Austria) reported no difference.

Three studies (one US; Switzerland; Germany) reported that late after ABF, readmissions increased, two of which reported a large increase. None of these studies reported p-values. Four studies (three US; England/Scotland) reported a decrease in readmissions of which two reported a large and, at least for some sub-groups, statistically significant difference. Three studies (all US) reported no difference. Appendices 17, 18, 19 (see file S1) present study descriptions, main findings, and analyses.

#### Readmission Summary

The pooled analysis showed no difference in readmissions after ABF, but some studies showed a significant increase and others a significant decrease. In the non-pooled analysis, most studies showed an increase in readmissions early in ABF. In the later ABF period, results were mixed.

### Discharge Destination

#### Discharge to PAC Pooled results

**Figure 4:**
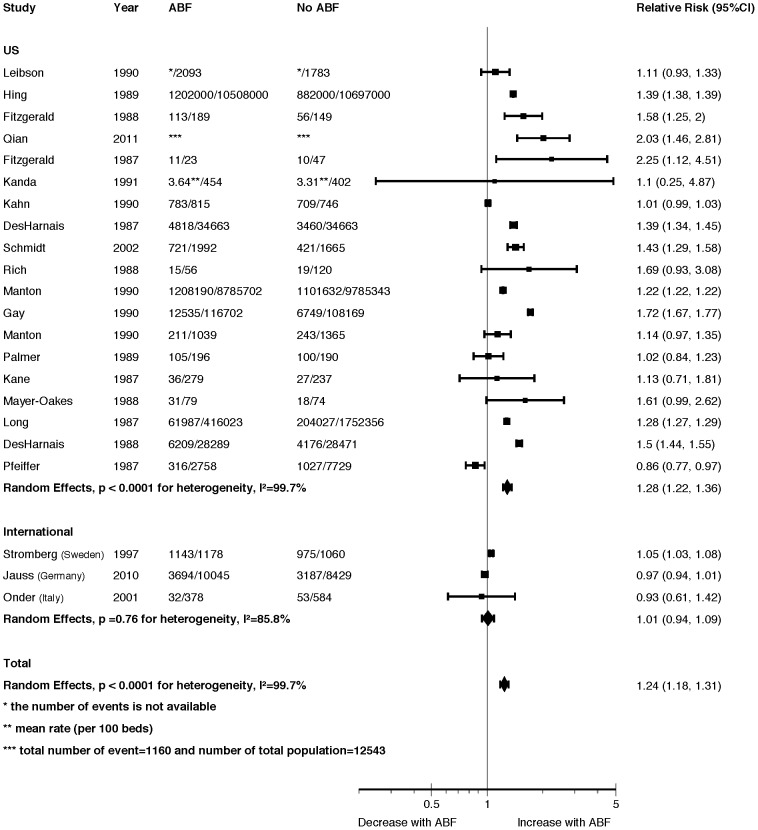
Discharge Destination Forest Plot.

Pooled data from twenty-two studies (19 US; Sweden; Germany; Italy) showed an increase in discharge to PAC with ABF (pooled RR = 1.24, 95% CI 1.18 to 1.31, I^2^ = 100%) (Figure 4). In a sub-group analysis, there was a 28% relative increase in discharge to PAC in the 19 US studies (RR = 1.28, 95% CI 1.22 to 1.36, I^2^ = 100%), but no difference in the 3 international studies (RR = 1.01, 95% CI 0.94 to1.09, I^2^ = 86%) (test for interaction *p* = 0.04) (Appendix 20, see File S1). Only one US study of lowest credibility showed a decrease in discharges to PAC with ABF [76]. Despite the very high I^2^, the U.S. studies were consistent in showing an increase in discharge to PAC. None of our other pre-specified sub-groups explained the residual heterogeneity. The funnel plot did not suggest publication bias (Appendix 21, see File S1). Appendix 22 (see File S1) presents study descriptions.

#### Discharge to PAC Non-Pooled Results (Early ABF and Late ABF)

Seven studies (all US) reported that early after ABF implementation there was a large increase in discharge to PAC; one reported p<0.01, the others did not report p-values. One US study reported a large decrease in discharge to PAC without reporting an associated p-value. One US study reported mixed results.

Five studies (all US) reported that late after ABF, discharge to PAC increased; in four the increases were large. None of these studies reported p-values. One US study reported a large decrease in admissions to PAC (p<0.05). One US study reported an increase to some types of PAC and a decrease to others, and one an increase in two sub-groups and a decrease in one other. Study descriptions, main findings, and analyses are found in Appendices 23, 24, 25 (see File S1).

#### Discharge to PAC Summary

The pooled analysis showed a 24% increased risk of discharge to some form of PAC, a result consistent with the non-pooled data. The pooled analysis also showed a possible sub-group effect with a 28% increased risk of discharge to PAC in the 19 US studies, but the effect was not observed in the three international studies.

### Severity of Illness

#### Severity of Illness Non-pooled results (Early ABF and Late ABF)

Nine studies (six US; two Italy; Australia) reported that early after ABF implementation there was an increase in severity of illness of which seven reported a large effect (five US; Italy; Australia); of these, five reported some significant p-values. In two studies, the magnitude of the increase was not reported; of these, one reported a p-value of <0.01. Three studies (two US; Germany) reported that early after ABF there was a decrease in severity of illness. Three studies (two US; Australia) reported no difference and three studies (two US; Italy) reported mixed results.

Nine studies (eight US; Germany) reported that late after ABF there was an increase in severity of illness, large in five studies, moderate in two, and mixed in two; none reported p-values. Three studies (two US; Germany) reported a large decrease in severity of illness, two without reporting p-values and one reporting p<0.01. One study (US) reported no difference and four reported mixed results (three US; Switzerland). Study descriptions, main findings, and analyses tables are found in Appendices 26, 27, 28 (see File S1).

#### Severity of Illness Summary

Reported severity of illness increased both early and late after ABF in the majority of studies, though results varied across studies. There was no difference between US and international.

### Volume of Care

#### Volume of Care Non-pooled results (Early ABF and Late ABF)

Six studies (two US; Italy; Australia; Sweden; England/Scotland) reported that early after ABF implementation volume of care increased, of which five reported a large increase, but without reporting p-values. One study reported a significant increase in volume (p<0.01) but without reporting the magnitude. Eight studies (five US; two Italy; Switzerland) reported a decrease in volume of care of which six reported a large and one a moderate decrease; seven of these studies did not report p-values and one a large decrease with a p-value of <0.001. Three studies (two US; Austria) reported no difference and two (US; Australia) mixed results.

Six studies (five US; England/Scotland) reported that late after ABF volume of care increased, large in four studies and moderate in one (but without reporting p-values) and significant in one (p<0.01, but without reporting magnitude). Four studies (three US; Australia) reported a decrease in volume of care, of which three reported a large decrease but no p-values, and one reported p<0.05. One US study reported no difference and one mixed results. Study descriptions, main findings, and analyses tables are found in Appendices 29, 30, 31 (see File S1).

#### Volume of Care Summary

Results regarding the impact of ABF on volume of care showed high variability, with approximately the same number of studies showing an increase as decrease both early and late after ABF was implemented. There was no difference between US and international studies.

## Discussion

### Principal Findings

Our study is the first systematic review of the international literature addressing the impact of activity-based funding of hospitals. The evidence suggests no consistent impact of ABF on mortality in either acute or post-acute care. There was no impact on rates of readmission to hospital in the pooled analysis, but a suggestion of an increase in readmission early after ABF from the studies without poolable data. We found an apparent though highly variable increase in reported severity of illness, and no difference in volume of care, though this, too, was highly variable. Our most notable finding was a large increase in admissions to PAC after a hospital stay, though this appeared restricted to US and not international settings.

### Strengths and Limitations

Strengths of this review included: explicit eligibility criteria; a comprehensive search that yielded a large number of eligible studies; assessment of the credibility of each eligible study; a rigorous approach to data abstraction and summarization of studies, both those that provided data amenable to statistical analysis and those that did not; tests of *a priori* hypotheses of possible explanations for heterogeneity; and duplicate assessment of eligibility, credibility, and data abstraction with third party adjudication when necessary.

The main limitation of our review lies in the deficiencies of the primary studies. The majority of eligible studies were before-after studies; thus, when differences are evident, they may be the result of temporal trends independent of ABF. This is of particular concern because hospital funding reform is rarely implemented as a solitary intervention. For instance, adoption of ABF in the English NHS was accompanied by a wave of competitive internal market reforms [90]–[92]. ABF introduction in the United States was followed by a restructuring of peer review organizations to externally audit physicians and hospitals for quality indicators [93]. Other secular trends that may confound attribution of outcome differences to ABF include changes in technology and related shifts from inpatient to outpatient care, and changes in other funding policies (for instance, funding for post-acute care).

Low credibility of many studies also limits strong inferences from the evidence. Credibility problems included lack of documentation of quality control and error rates in the administrative databases that provided data for the analyses; lack of statistical adjustment and, when present, lack of comprehensive and appropriate adjustment. However, we assessed whether the higher quality studies yielded different results than the lower quality studies. This was not the case, suggesting that inferences can be drawn from the whole body of evidence. In addition, many studies did not provide information necessary for inclusion in the pooled statistical analysis, nor did they report on the magnitude of effects, their statistical significance, or both. Inconsistency in study results, and the failure of our *a priori* hypotheses to explain the inconsistency, also decreases the strength of inferences from the results. There may be concomitantly implemented policies (e.g. quality improvement incentives) that may make it more likely that jurisdictions will see the potential benefits and not the potential harms of ABF; if this is so, our review provides no insight into what these strategies may be.

Finally, another limitation is the danger that upcoding in ABF settings led to differences in the recorded classification of severity after ABF was implemented [19]. Generally, in observational studies such as these, adjustment for key variables is crucial for making causal inferences. In this case, to the extent that upcoding exists, all adjusted analyses will be biased in favor of ABF. This concern is somewhat ameliorated by the similarity of effects in the adjusted and unadjusted analyses.

### Relation to prior work

Prior reviews of ABF have shown variable results [2], [4], [24], [94]–[98]. This is not surprising since none was systematic or comprehensive, and thus may be susceptible to study selection bias.

Prior studies have consistently established that the transition to ABF for hospitals initially decreased the length of hospital stay in the US and internationally, though it appeared to stabilize after the initial decrease [42], [57], [99]. Although we did not review this evidence systematically, prior non-systematic reviews have been highly consistent in their findings and conclusions. Accepting this finding as definitive, we did not include length of hospital stay as one of our outcomes.

### Implications of our Findings

Our results represent the best available evidence, and thus the best guide for current decision-making regarding ABF. The results demonstrate that strong claims of either benefits or harms of ABF in the outcomes we studied are unwarranted. The appreciable inconsistency of results across studies in most outcomes suggests that there may be contexts in which ABF has substantial positive or negative consequences. For example, it may be the case that specific attributes of how ABF is implemented, such as with activity limits, or policies of non-payment for readmissions, could affect the outcomes we studied. We were not, however, able to abstract and analyze data for contextual factors that clearly explained differences in effect, and thus to determine the circumstances in which ABF is likely to produce positive or negative outcomes.

Reducing hospital length of stay is a worthwhile policy objective. Unless there are clear and substantial negative consequences to ABF, this alone might provide a rationale for its implementation. Our review failed to identify compelling evidence for such negative consequences, at least in terms of mortality or hospital readmissions, although some studies raise the possibility of detrimental effects on these outcomes under some circumstances. Again, however, the evidence does not allow us to identify the circumstances in which this is more likely to be the case.

ABF models have provided, in theory, a disincentive for hospitals to re-admit patients quickly [8], [100]–[102]. Our data, however, provide low credibility evidence for a possible increase in readmission early after the implementation of ABF. Results, however, are highly variable and also consistent with no impact whatsoever of ABF on readmission.

The evidence may be obscuring a true increase in readmissions. For instance, patients may have been readmitted with a different admission diagnosis to avoid financial penalties for excess readmissions (even though this may attract charges of fraud) [103]–[105]; others may have been held in hospital under “out-patient observation status” but never technically readmitted [106], [107]. This interpretation speaks to the literature on the opportunities for “gaming” under ABF systems [4], [108], such as the cataract unbundling we have described in the results [49]. It is also possible that in response to earlier discharge, physicians intensified their follow-up care, thereby preventing readmissions [78]. The increased rate of discharge to post-acute care suggests that more intensive or more frequent use of post-hospital care may indeed have prevented an increase in readmission to acute care hospitals.

Our robust finding that, at least in the US, ABF is associated with increased discharge from hospital to PAC settings including rehabilitation facilities, home health agencies, and other forms of intermediate non-hospital health care suggests that introducing ABF may come at a price for patients recently discharged from acute care hospitals, but still needing care outside the hospital sector before returning home. The finding is not unanticipated: a funding model designed to reduce length of hospital stay provides a powerful incentive to discharge patients not yet medically stable enough to leave the health care system entirely.

Earlier discharge from hospital to PAC is not necessarily undesirable: assuming there is sufficient post-acute care capacity to meet demand it may be preferable for patients to enter a PAC facility, or to return home earlier with professional home care, rather than spend extra time in hospital. The danger, however, is that if health care systems are not equipped to deal with the additional burden of a potential 24% increase in patients requiring some form of post-acute care following their earlier discharge, then patients and their family caregivers at home will suffer [109]. Such a large increase in PAC admissions might also offset potential savings from any improved efficiency in acute care settings.

Further evidence supporting the increase in PAC discharges in the US following introduction of ABF includes the associated proliferation of cost-based post-acute facilities and programs that were initially exempt from ABF [110]. Between 1988 and 1997, expenditures on PAC increased at an average annual rate of 25 percent [111]. The increase in patients discharged to PAC, even in the US, may have been a temporary phenomenon. In 1997, the passage of legislation dramatically altered Medicare's PAC payment policies, shifting payment to PAC providers from a cost-basis to prospective payment. This change decreased the financial incentive for PAC facilities to admit from hospital those patients needing a relatively high intensity of care [112]. In an effort to ensure that care is provided in the most appropriate setting with shared resources, some health care systems are now considering other forms of bundled payment, beyond ABF of hospital-specific care, to cover the entire scope of services, across settings and providers —before, during, and after hospitalization — for a particular episode of care [113]–[115].

The evidence suggests that the rate of discharge to PAC may be a phenomenon restricted to the US. The credibility of this sub-group finding is, however, only moderate: although one of a relatively small number of *a priori* hypotheses and unlikely to be explained by chance (interaction p-value 0.04), the finding is based on only three studies outside of the US and is not consistent (one study conducted in Stockholm, Sweden showed a small but statistically significant increase in discharge to PAC with ABF [84]).

In countries like the US, where a post-acute care sector was highly developed as a policy response to the implementation of ABF in hospitals in 1983, the patient route to PAC was well-travelled. But in countries in which ABF is a more recent innovation, such as Sweden [84], Italy [74], and Germany [56], the apparent absence of increased PAC discharges suggests that underlying system and cultural differences may influence the impact of ABF. It may be, for instance, that having witnessed the substantial PAC sector that developed to accommodate the transformative effect of ABF in the US, European social service support systems developed primarily outside the institutional health care sector, meeting the post-acute care needs of patients at home, through community caregivers, or by families' shouldering the care burden. It may also be that patients in some countries are moved to PAC settings within the same hospital, thereby not being counted as discharged to PAC. Funding design outside the US may also have incorporated volume capping or volume growth moderation strategies (e.g. tapering payment for additional volume) which would have reduced the benefit to hospitals of earlier discharge to PAC – but also would make it less likely to shorten hospital stay [9].

Perhaps the most optimistic interpretation of the sub-group difference between international and US studies in PAC settings would be one of policy learning. Europe implemented ABF 20 years or more after the US. The US studies report data mostly from the 1980s, and their relevance may be somewhat limited given the refinements in ABF over time, such as the addition to the DRG classification system of a severity measure that may have, for example, increased the European LOS sufficiently that patients require less PAC. Alternatively, perhaps we have yet to see the full effect of ABF on Europe's health care systems, especially in the PAC sector. Published evidence about the development of Europe's PAC sector in the post-ABF era is scant [4]. That we have only three international studies in our review further limits the strength of any inferences about how ABF impacts discharges to PAC outside the US.

The policy implications of our results are uncertain. Should health systems now implementing ABF increase their capacity in the PAC setting to accommodate anticipated PAC discharges? Or can they anticipate that, as we found in the three international studies, discharges to PAC may not increase? In jurisdictions with limited informal networks of care providers or where community-based services are generally less well-developed, the risk of increased need for PAC facilities may be particularly high. If funding changes give rise to new demands for PAC, and if social protection systems are slow to adapt, hardship will follow. In countries in which hospital funding is almost exclusively provided by governments, but in which funding and delivery of post-acute care is a mixed public-private enterprise with families frequently paying out-of-pocket for long term care or home care services, any shift of care out of hospital and into the community sector has the potential to threaten equitable access to care.

The shortened LOS that appears to be associated with ABF should, in theory, pave the way for increasing volume of care, particularly number of admissions. Improving access to care by reducing waiting times is one potential policy objective of ABF and faster patient turnover could enable this. We found, however, no difference in volume of care, particularly number of acute admissions, with ABF, though the results were highly variable.

One concern, expressed by critics of ABF, is the potential to threaten equity of access by creating “profitable” and “unprofitable” diagnoses, or procedures, or programs — and thus patients — with resulting avoidance of unprofitable services [95], [116], [117]. For instance, one study found that following ABF implementation in South Carolina, the provision of TURP surgery declined by 25% for the “old-old” who tended to require longer hospital stays and have more complications, but increased 100% for the “young-old” [49]. To the extent that sicker patients are liable to be less profitable, our analysis does not support this concern: we did not find a decrease in severity of illness across populations admitted to hospital before and after ABF. Rather, we found an overall apparent increase, albeit highly variable, in the severity of illness.

This finding may be explained by differences in coding. Since ABF models tend to adjust hospital compensation for acuity, there is a financial incentive to code patients so they appear as sick as possible, thus maximizing reimbursement. Upcoding may be appropriate (if it represents legitimately better coding), questionable, or inappropriate. In Germany, for example, the introduction of ABF led to a very large increase in low birth weight babies and a decrease in normal birth weight babies over a 5-year period, with no biological or epidemiological explanation for this effect [118]. In the US, one study showed a decrease in overall case-fatality rate with ABF, coupled with a large increase in volume of septicaemia diagnoses, a combination highly suggestive of inappropriate upcoding [54]. To the extent that inappropriate upcoding occurs, it is likely to undermine at least one ABF policy objective: controlling costs to payer.

## Conclusions

Inferences regarding the impact of ABF are limited both by inevitable study design constraints (randomized trails of ABF are unlikely to be feasible) and by avoidable weaknesses in methodology of many studies. Further, the variation in results across studies suggests that the impact of ABF may vary across settings, though the evidence does not provide strong clues of the determinants of differential effects. The ABF story thus provides testimony to how modifications in health policy without adequate evaluation leave their impact open to great uncertainty.

The most compelling evidence of any impact on ABF is on an initial, but likely not sustained, decrease in hospital length of stay following introduction of ABF, a possible increase in readmissions, and an increase in discharges to post-acute care. The latter impact may be restricted to the introduction of ABF in the US. Other effects are likely absent, or restricted to specific settings, and possibly manifest in different directions depending on the setting. Thus, the evidence on the variables we studied does not support either strong advocacy for, or strong rejection of, a change to ABF from other hospital funding methods.

Those considering adoption of ABF should be aware of the probable increase in admissions to post-acute care and other possible unintended adverse consequences that may arise. A paucity of consistent and conclusive evidence on the outcomes we studied, however, does not allow a jurisdiction to predict if ABF would be harmless. To this extent, despite the long and extensive experience, changing to an ABF system represents a leap of faith.

## Supporting Information

File S1 **Appendices 1–31.**
(PDF)

Checklist S1 **PRISMA Checklist.**
(DOC)

Protocol S1 (DOCX)
