# Correction: Activity-Based Funding of Hospitals and Its Impact on Mortality, Readmission, Discharge Destination, Severity of Illness, and Volume of Care: A Systematic Review and Meta-Analysis

**DOI:** 10.1371/journal.pone.0121163

**Published:** 2015-03-24

**Authors:** The PLOS ONE Staff

There is an error in affiliation 1 for the author Karen Palmer. Affiliation 1 should be: Faculty of Health Sciences and Faculty of Science Simon Fraser University, Burnaby, British Columbia, Canada. The publisher apologizes for this error.
