# Supplementary material for: Activity-Based Funding of Hospitals and Its Impact on Mortality, Readmission, Discharge Destination, Severity of Illness, and Volume of Care: A Systematic Review and Meta-Analysis
Source: PLoS One. 2014 Oct 27;9(10):e109975. doi: 10.1371/journal.pone.0109975 (PMC4210200; doi:10.1371/journal.pone.0109975)
Supplement: File S1 — Appendices 1–31. (PDF) [file pone.0109975.s001.pdf]

Appendices

TABLE OF CONTENTS

TABLE OF CONTENTS 1
Appendix 1: Search Strategy (e.g. EMBASE) 3
Appendix 2: Statistical Analysis Methodology (Pooled) 5
Appendix 3: Summaries of Study Credibility Tables 6
Appendix 4: Acute Care Mortality Sub-Group Meta-Regression Table 9
Appendix 5: Acute Care Mortality Funnel Plot 10
Appendix 6: Acute Care Mortality Study Descriptions Table (Pooled) 11
Appendix 7: Acute Care Mortality Study Descriptions and Main Findings Table (Non-Pooled) 13
Appendix 8: Acute Care Mortality Analysis Table (Non-Pooled) 14
Appendix 9: Acute Care Mortality Vote Count Table (Non-Pooled) 15
Appendix 10: Post-Acute Care Mortality Forest Plot 16
Appendix 11: Post-Acute Care Mortality Study Descriptions and Main Findings Table (Pooled and Non-Pooled) 17
Appendix 12: Post-Acute Care Mortality Analysis Table (Non-Pooled) 19
Appendix 13: Post-Acute Care Mortality Vote Count Table (Non-Pooled) 20
Appendix 14: Readmission Sub-Group Meta-Regression Table 21
Appendix 15: Readmission Funnel Plot 22
Appendix 16: Readmission Study Descriptions Table (Pooled) 23
Appendix 17: Readmission Study Descriptions and Main Findings Table (Non-Pooled) 26
Appendix 18: Readmission Analysis Table (Non-Pooled) 31
Appendix 19: Readmission Vote Count Table (Non-Pooled) 32
Appendix 20: Discharge Destination Sub-Group Meta-Regression Table 33
Appendix 21: Discharge Destination Funnel Plot 34
Appendix 22: Discharge Destination Study Descriptions Table (Pooled) 35
Appendix 23: Discharge Destination Study Descriptions and Main Findings Table (Non-Pooled) 39
Appendix 24: Discharge Destination Analysis Table (Non-Pooled) 44
Appendix 25: Discharge Destination Vote Count Table (Non-Pooled) 45
Appendix 26: Severity of Illness Study Descriptions and Main Findings Table (Non-Pooled) 46

Appendix 27: Severity of Illness Analysis Table (Non-Pooled) 58
Appendix 28: Severity of Illness Vote Count Table (Non-Pooled) 59
Appendix 29: Volume of Care Study Descriptions and Main Findings Table (Non-Pooled) 60
Appendix 30: Volume of Care Analysis Table (Non-Pooled) 67
Appendix 31: Volume of Care Vote Count Table (Non-Pooled) 68

Appendix 1: Search Strategy (e.g. EMBASE)

Studies were identified through searching nine electronic bibliographic databases: OVID MEDLINE (1946 – June week 2 2012), EMBASE (1980 – 2012 week 12), OVID Healthstar (1966 to May 2012), CINAHL (1981 – June 2012), Cochrane Central Register of Controlled Trials (CENTRAL) (July 2012), Health Technology Assessment (3rd Quarter 2012), NHS Economic Evaluation Database (3rd Quarter 2012), Cochrane Database of Systematic Reviews (3rd Quarter 2012), and Business Source Complete (1886 – mid July 2012).

Database: Embase <1980 to 2012 Week 25>
Search Strategy:

----

1 (activity based adj3 (fund: or financ: or resource: or system: or reimburs: or management or payment: or income: or account: or allocation or information)).mp.
2 ("payment by results" or "payment by result").mp.
3 (case payment adj2 (mechanism: or system: or management)).mp.
4 case mix group:.mp.
5 health resource group:.mp.
6 ((case mix or casemix) adj2 (fund: or financ: or resource: or system: or reimburs: or cost: or management or payment: or income: or account: or allocation or information)).mp.
7 patient based payment:.mp.
8 volume based fund:.mp.
9 "payment for volume".mp.
10 service based fund:.mp.
11 (patient focused fund: or patient focussed fund:).mp.
12 healthcare resource group:.ti,ab.
13 diagnosis procedure combination.mp.
14 or/1-13
15 diagnosis related group:.mp. or exp Diagnosis-Related Groups/
16 ((drg or drgs) not (dorsal or neurons or protein:)).mp.
17 15 or 16
18 prospective payment system:.mp. or Prospective Payment System/
19 17 and 18
20 19 not (home.ti,ab. not hospital:.mp.)
21 14 or 20
22 diagnosis related group:.ti,ab. or exp *diagnosis related groups/
23 prospective payment system:.ti,ab. or *prospective payment/
24 ((drg or drgs) not (dorsal or neurons or proteins)).ti,ab.
25 22 or 23 or 24
26 reimbursement/ or reimburs:.ti,ab.
27 hospital finance/ or "hospital running cost"/ or "hospitalization cost"/
28 "health care cost"/
29 health care financing/
30 "hospital cost"/
31 incentive:.mp.
32 (funding adj2 system:).mp.
33 health services research/
34 health care policy/
35 hospital economics.mp.
36 health care.ti.
37 access.mp.
38 budgets.mp. or budget/ or funding/
39 "cost control"/ or cost control.mp.
40 "cost benefit analysis"/ or "cost effectiveness analysis"/

41 resource allocation.mp. or resource allocation/
42 cost effectiveness.mp.
43 cost efficiency.mp.
44 economic analysis.mp.
45 length of stay.mp. or "length of stay"/
46 patient care/
47 exp hospital/ or hospital:.ti,ab.
48 hospital administrator/
49 ambulatory surgery/
50 ((surgical or surgery) adj2 (facilit: or center: or centr:)).mp.
51 inpatients.mp. or exp hospital patient/
52 inpatient.mp.
53 ("in patient" or "in patients").mp.
54 exp mortality/ or mortality.mp.
55 morbidity.mp. or exp morbidity/
56 hospital charge/
57 financial management/
58 national health service/
59 or/26-58
60 25 and 59
61 prospective payment/
62 (bundled adj2 payment).mp.
63 61 or 62
64 26 or 27 or 28 or 30 or 32 or 35 or 39 or 40 or 41 or 42 or 43 or 45 or 47 or 48 or 49 or 50 or 51 or 52 or 56 or 57 or 58
65 63 and 64
66 65 or 60
67 21 or 66
68 limit 67 to yr="1980 -Current"
69 (oig pps or hsfa pps or hsfa rule or pprs rule or drg rates).mp.
70 (drg revised rates or drg rule or (final rule and drg)).mp.
71 (pps rates or (hcfa and pps) or (oig and pps) or pps rule).mp.
72 (drg rates or (drg and revised rates) or (final rule and drg) or (drg and rule)).mp.
73 (drg leptin or (drg and icd-9) or drg 541 or drg adjusted).mp.
74 (apr drg or a drg or drg database or drg data base).mp.
75 (drg kit or drg scores or ms drg).mp.
76 (medicare severity adjusted and drg).mp.
77 or/69-76
78 68 not 77
79 drug design/ or drug industry/ or drug marketing/ or drug research/ or drug information/ or drug storage/
80 dentistry/ or medicolegal.mp. or medico legal.mp.
81 (nicotine or vaccine).sh. or organ donor/ or prescription drugs.ti,ab.
82 pharmac*.mp.
83 (nursing home: or home care).ti.
84 (drug monitoring or drug benefit: or drug usage).mp.
85 community medicine/
86 (coding or malpractice).ti.
87 or/79-86
88 78 not 87
89 limit 88 to (editorial or letter or note)
90 88 not 89
91 "18795557".ui.
92 90 or 91

****

Appendix 2: Statistical Analysis Methodology (Pooled)

We used SAS, v9.2. statistical package to analyze the data.

We used relative risk (RR) or odds ratio (OR) as the effect estimate for the pooling. The choice of which estimate to be used for each outcome was mainly determined by the type of adjusted estimates that was(were) available among the studies. When there was no adjusted result available for the study, the 2x2 table was used for calculating the RR or OR. In addition, we used the HR as the RR (averaged-over-time) when the adjusted RR was not available. Moreover, in one occasion, the mean incident rate ratio was used to approximate the RR since it was the only relevant estimate available from the article.

We estimated the pooled log (RR or OR) using a random effects inverse variance meta-analysis and then, for purposes of presentation, back transformed to the RR or OR. To explore factors that might be associated with the heterogeneity among the studies, we performed a uni-variate meta-regression analysis on log (RR or OR) using the following independent variables:

US vs. International: US vs. non-US

Credibility: High vs. Low

Analysis: Adjusted VS. Unadjusted

Study design: Before-after VS. Parallel groups

ABF (time after implementation): Early VS. Late

Readmission measured over a period of: less than or equal to 30 days VS. greater than 30 days

Appendix 3: Summaries of Study Credibility Tables

Appendix 3.1: Summary of Study Credibility (Pooled)

Credibility Question	Variable			
	AC Mortality (n=8)	PAC Mortality (n=3)	Readmission (n=13)	Discharge to PAC (n=22)
1. What was the quality of the adjustments?				
Comprehensive and appropriate	2	1	2	2
Limited	2	0	0	2
Minimal	4	2	11	18
2. Did the study use original data collection, or document the quality of the data source?				
Yes	4	0	5	4
Mostly yes	0	0	0	2
Mostly no	3	1	4	6
No	1	2	4	10
3. How many variables were simultaneously assessed in the study?				
1	0	0	1	2
2	1	0	1	5
3	3	1	6	7
4	2	1	3	6
5	2	1	2	2
6	0	0	0	0
4. Credibility Score				
0	0	0	1	3
1	1	2	3	8
2	1	0	3	5
3	1	0	1	0
4	4	1	4	5
5	0	0	0	1
6	1	0	1	0
5. Credibility Classification				
High	5	1	5	6
Low	3	2	8	16

Appendix 3.2: Summary of Study Credibility (Non-Pooled)

Credibility Question	Variable					
	AC Mortality (n=4)	PAC Mortality (n=1)	Readmission (n=17)	Discharge to PAC (n=14)	Severity of Illness (n=30)	Volume of Care (n=26)
1. What was the quality of the adjustments?						
Comprehensive and appropriate	0	0	3	4	30	3
Limited	1	0	4	3	0	3
Minimal	3	1	10	7	0	20
2. Did the study use original data collection, or document the quality of the data source?						
Yes	1	0	0	0	8	5
Mostly yes	1	0	2	2	1	2
Mostly no	1	0	9	7	9	6
No	1	1	6	5	12	13
3. How many variables were simultaneously assessed in the study?						
1	0	0	4	4	0	0
2	3	0	5	8	3	10
3	1	0	5	2	13	6
4	0	0	1	0	9	5
5	0	1	2	0	5	5
6	0	0	0	0	0	0
4. Credibility Score						
0	1	0	1	1	0	3
1	0	1	5	8	0	10
2	1	0	8	1	0	4
3	2	0	2	2	15	6
4	0	0	1	2	6	2
5	0	0	0	0	1	0
6	0	0	0	0	8	1
5. Credibility Classification						
High	0	0	1	2	15	3
Low	4	1	16	12	15	23

Appendix 3.3: Summary of Study Credibility (Pooled and Non-Pooled)

**Credibility Question**	**Variable**					
	AC Mortality (n=12)	PAC Mortality (n=4)	Readmission (n=30)	Discharge to PAC (n=36)	Severity of Illness (n=30)	Volume of Care (n=26)
1. What was the quality of the adjustments?						
Comprehensive and appropriate	2	1	5	6	30	3
Limited	3	0	4	5	0	3
Minimal	7	3	21	25	0	20
2. Did the study use original data collection, or document the quality of the data source?						
Yes	5	0	5	4	8	5
Mostly yes	1	0	2	4	1	2
Mostly no	4	1	13	13	9	6
No	2	3	10	15	12	13
3. How many variables were simultaneously assessed in the study?						
1	0	0	5	6	0	0
2	4	0	6	13	3	10
3	4	1	11	9	13	6
4	2	1	4	6	9	5
5	2	2	4	2	5	5
6	0	0	0	0	0	0
4. Credibility Score						
0	1	0	2	4	0	3
1	1	3	8	16	0	10
2	2	0	11	6	0	4
3	3	0	3	2	15	6
4	4	1	5	7	6	2
5	0	0	0	1	1	0
6	1	0	1	0	8	1
5. Credibility Classification						
High	5	1	6	8	15	3
Low	7	3	24	28	15	23

Appendix 4: Acute Care Mortality Sub-Group Meta-Regression Table

Exploratory variables in meta-regression analysis		OR (95%CI)	p-value	R^2
US vs. International	US (n=6)	1.04(0.88, 1.24)	0.83	0.01
	International (n=2)	1.01 (0.74, 1.38)		
Credibility	High (n=5)	1.07 (0.88, 1.29)	0.57	0.06
	Low (n=3)	0.99 (0.79, 1.24)		
Analysis	Adjusted (n=4)	1.03 (0.87, 1.23)	0.99	0
	Unadjusted (n=4)	1.03 (0.76, 1.41)		
Study design	Before-after (n=7)	1.02 (0.90, 1.16)	0.17	0.29
	Parallel groups (n=1)	1.70 (0.77, 3.73)		
ABF (Time after implementation)	Early (<= 2 years; n=2)	0.95 (0.69, 1.3)	0.48	0.09
	Late (>2 years; n=6)	1.06 (0.90, 1.24)		
Mortality measured over a period of	less than or equal to 30 days (n=4)	1.02 (0.86, 1.21)	0.65	0.04
	greater than 30 days (n=4)	1.09 (0.81, 1.47)		

Appendix 5: Acute Care Mortality Funnel Plot

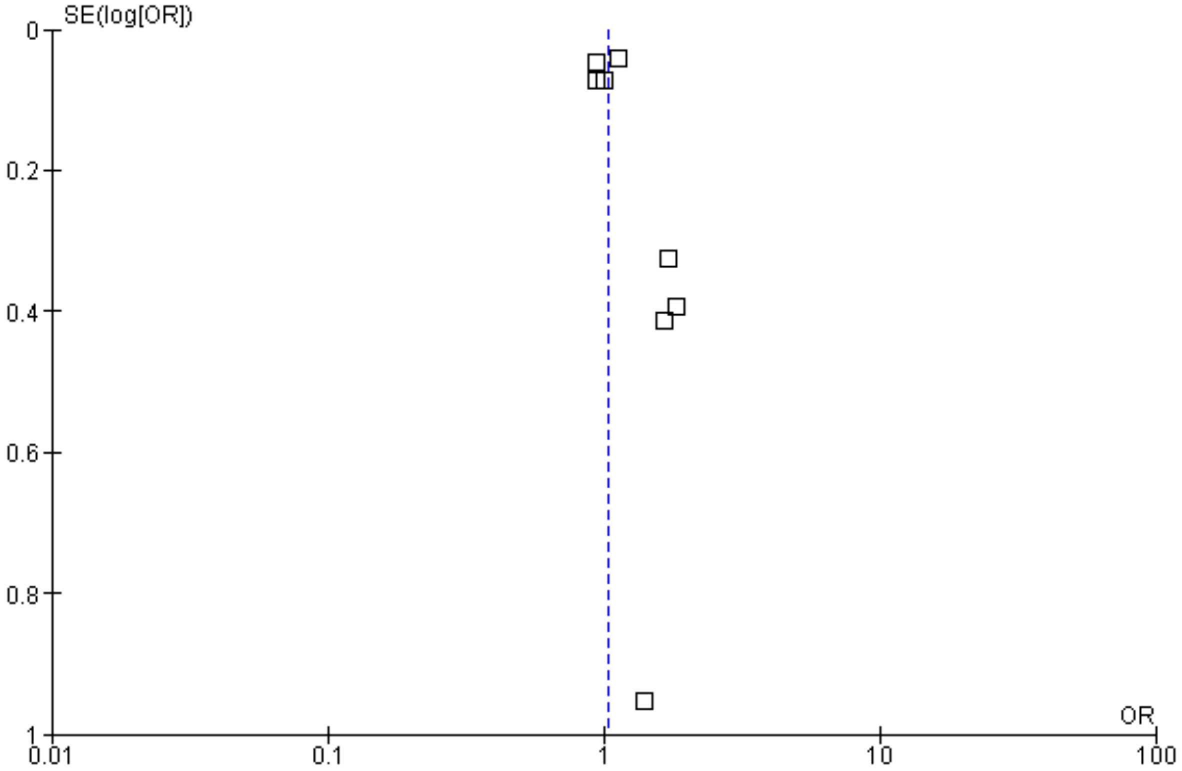

Appendix 6: Acute Care Mortality Study Descriptions Table (Pooled)

Author (year)	Country (start year ABF)	Study Design	Data Dates (% funding from ABF)*	Primary data source<br><br>Sampling method	Study population<br><br>(N patients / N institutions)	Overall Credibility Score (0-6)	Variable and main findings (outcome direction, magnitude, statistical significance)
**US**							
Draper (1990)<br><br>RefID: 12938	USA (1983)	Before/after	No ABF:<br>1981-1982 (0%)<br><br>Late ABF:<br>1985-1986 (50-75%)	Health Care Financing Administration (HCFA) MedPAR file on all hospitalized patients for mortality. Individual medical record.<br><br>Random sample	Medicare patients ≥ 65 hospitalized in each study year with one of the study diseases (congestive heart failure, acute myocardial infarction, pneumonia, cerebrovascular accident, hip fracture, and depression)<br><br>16758/297	4	**AC mortality: 30 days**<br><br>See forest plot
Kahn (1990)<br><br>RefID: 1461	USA (1983)	Before/After	No ABF:<br>1981-1982 (0%)<br><br>Late ABF:<br>1985-1986 (50-75%)	Medical record as source of in-hospital mortality information and Health Care Financing Administration (HCFA) files. Also, Medicare's Part B files<br><br>Convenience sample	Medicare patients hospitalized in 1981 through 1982 and 1985 with one or more of:<br>Congestive Heart Failure<br>Acute Myocardial Infarction<br>Pneumonia<br>Cerebrovascular accident<br>Hip Fracture<br><br>14012/ns	4	**AC mortality: 30 days**<br><br>See forest plot
Fitzgerald (1988)<br><br>RefID: 5101	USA (1983)	Before/After	No ABF:<br>1981-1983 (0%)<br><br>Late ABF:<br>1984-1986 (25-75%)	1. Hospital records in a community hospital in a large Midwestern city.<br>2. Telephone interviews.<br><br>All eligible institutions in a jurisdiction	All Medicare patients ≥ 65 years old admitted to hospital with a new hip fracture between October 1, 1981 and March 1, 1986.<br><br>338/1	4	**AC mortality: 1 year**<br><br>See forest plot
Fitzgerald (1987)<br><br>RefID: 5100	USA (1983)	Before/After	No ABF:<br>1981-1983 (0%)<br><br>Early ABF:<br>1984-1985 (25-50%)	Patient hospital charts.<br><br>All eligible institutions in a jurisdiction	All patients admitted to one Indianapolis tertiary hospital with a non-pathologic hip fracture<br><br>70/1	4	**AC mortality: 6 months**<br><br>See forest plot
Ray (1990)<br><br>RefID: 14607	USA (1983)	Before/after	No ABF:<br>1981-1983 (0%)<br><br>Late ABF:<br>1984-1986 (25-75%)	Central Health Care Financing Records for Michigan<br><br>Random sample	Michigan Medicare patients ≥ 65 years admitted with a hip fracture<br><br>4368/ns	3	**AC mortality: 1 year**<br><br>See forest plot
Gerety (1989)<br><br>RefID: 1058	USA (1983)	Before/After	No ABF:<br>1982-1984 (0%)<br><br>Late ABF:<br>1984-1986 (25-75%)	Patient medical charts<br><br>Convenience sample	Patients ≥ 69 years with ICD or DRG codes for hip fracture admitted to Stanford University Medical Centre<br><br>180/1	2	**AC mortality: 1 year**<br><br>See forest plot
**International**							
Schuetz (2011)<br><br>RefID: 8140	Switzerland (2001)	Parallel groups	No ABF:<br>2006-2008 (0%)<br><br>Late ABF:<br>2006-2008 (100%)	Post-hoc analysis from previous RCT<br><br>Convenience sample	Patients with community acquired pneumonia, >18 years old<br><br>925/6	6	**AC mortality: 30 days**<br><br>See forest plot

Author (year)	Country (start year ABF)	Study Design	Data Dates (% funding from ABF)*	Primary data source<br><br>Sampling method	Study population<br><br>(N patients / N institutions)	Overall Credibility Score (0-6)	Variable and main findings (outcome direction, magnitude, statistical significance)
Jauss (2010)<br><br>RefID: 1421	Germany (2004)	Before/After	No ABF:<br>2003 (0%)<br><br>Early ABF:<br>2004 (100%)<br><br>Late ABF:<br>2006 (100%)	Administrative database of the German state of Hessen (6.1 million inhabitants) including all patients with stroke.<br><br>All eligible institutions in a jurisdiction	All patients diagnosed with ischemic stroke (excluding transitory ischemic stroke, hemorrhagic stroke).<br><br>27005/ns	1	**AC mortality: 7 days**<br><br>See forest plot

***US Medicare ABF Phase-in Schedule:**
Beginning on or after October 1, 1983 and before October 1, 1984: 25% ABF; Beginning on or after October 1, 1984 and before October 1, 1985: 50% ABF; Beginning on or after October 1, 1985 and before October 1, 1986: 75% ABF; Beginning on or after October 1, 1986: 100% ABF; If the percentage of ABF changed during the data reporting period, we used the percent ABF present for the majority of the period.
**ns=not specified

Appendix 7: Acute Care Mortality Study Descriptions and Main Findings Table (Non-Pooled)

Author (year)	Country (start year ABF)	Study Design	Data Dates (% funding from ABF)*	Primary data source<br><br>Sampling method	Study population<br><br>(N patients / N institutions)	Overall Credibility Score (0-6)	Variable and main findings (outcome direction, magnitude, statistical significance)
**US**							
Eggers (1987)<br><br>RefID: 4917	US (1983)	Before/after	Before ABF:<br>1981 (0%)<br><br>Early ABF:<br>1984 (25%)	Health Care Financing Administration (HCFA) database.<br><br>All eligible institutions in jurisdiction	All Medicare patients.<br><br>ns /ns	3	**Deaths within 6 weeks of first-time hospitalizations per 1000 beneficiaries:**<br><br>Before ABF: 29.2<br>Early ABF: 29.3<br>*(Outcome summary: increase early, magnitude < 1%, statistical significance not reported)*
Rogers (1990)<br><br>RefID: 2293	US (1983)	Before/after	Before ABF:<br>1981-1982 (0%)<br><br>Late ABF:<br>1985-1986 (50-75%)	Health Care Financing Administration (HCFA) database.<br><br>Convenience sample	All patients hospitalized in each study year with one of the study diseases.<br><br>17000/300	2	**Adjusted change in 180 day mortality rates by diagnosis, following admission to acute care:**<br><br>Before ABF vs. late ABF: Congestive heart failure -1.8%; Acute MI +1.4%; Pneumonia +1.1%; CVA -0.5%; Hip Fracture -2.9%<br><br>*(Outcome summary: mixed late, magnitude indeterminate, statistical significance not reported)*
Helms (1987)<br><br>RefID: 5724	US (1983)	Before/after	Before ABF:<br>1980 (0%)<br><br>Early ABF:<br>1984 (25%)<br><br>Late ABF:<br>1986 (75%)	1980-83: Medical records.<br><br>1984-86: Blue Cross billing records.<br><br>All eligible institutions in jurisdiction	Patients >18 admitted septicaemia.<br><br>4888/7	0	**Case-fatality rate (%) per year following admission to acute care:**<br><br>Before ABF (1980): 25.1%<br>Early ABF (1984): 10.8%<br>Late ABF (1986): 18.9%<br>*(Outcome summary: decrease early*, magnitude ≥ 5%, statistical significance; decrease late*, magnitude ≥ 5%, statistical significance*<br>*NB.<br>Dramatic decrease in case-fatality rate with ABF, coupled with dramatic increase in volume of septicaemia diagnoses, highly suggestive of upcoding. Not included in vote count.
**International**							
Farrar (2009)<br><br>RefID: 924	England and Scotland (2004 in England)	Before/after + Parallel Groups	No ABF:<br>2003-2004 (0%)<br><br>Early ABF:<br>2003-2005 (100%)<br><br>Late ABF:<br>2003-2006 (100%)	Hospital episode statistics (England, ABF-adopter).<br><br>Scottish morbidity records (Scotland, ABF non-adopter).<br><br>All eligible institutions in jurisdiction	All patients admitted to acute care hospitals in England and Scotland.<br><br>ns /297	3	**30-day post surgical mortality; % difference-in-difference between England and Scotland across time:**<br><br>Difference-in-Difference Early ABF: 0.03 % greater increase in mortality in ABF adopters (England) vs. non-ABF adopters (Scotland)<br>*(Outcome summary: greater increase early, magnitude indeterminate, statistical significance not reported)*<br><br>Difference-in-Difference Late ABF: 0% difference in mortality in ABF adopters (England) vs. non-ABF adopters (Scotland)<br>*(Outcome summary: no difference late, statistical significance not reported)*

*****US Medicare ABF Phase-in Schedule:
Beginning on or after October 1, 1983 and before October 1, 1984: 25% ABF; Beginning on or after October 1, 1984 and before October 1, 1985: 50% ABF; Beginning on or after October 1, 1985 and before October 1, 1986: 75% ABF; Beginning on or after October 1, 1986: 100% ABF; If the percentage of ABF changed during the data reporting period, we used the percent ABF present for the majority of the period.
**ns=not specified
Note: Studies stratified by US and International and ordered by credibility (highest to lowest) and then alphabetical by first author's last name.

Appendix 8: Acute Care Mortality Analysis Table (Non-Pooled)

Significance of Effect	Magnitude ≥5%*		Magn ≥1% to <5%		Magn <1%		Magn Indeterminate or mixed			
	Direction									
	Inc	Dec	Inc	Dec	Inc	Dec	Inc	Dec	No diff	Mixed
**Early ABF**										
p >.05										
p ≤ .05-.02										
p ≤ .01-.002										
p ≤ .001										
**Stat sig not reported					4917		924✓			
**Late ABF**										
p >.05										
p ≤ .05-.02										
p ≤ .01-.002										
p ≤ .001										
**Stat sig not reported									924✓	2293

* Formula for relative percent difference = (T2-T1/T1)(100)
**Abstracter judged increase, decrease, or no diff, but authors did not report statistical significance OR authors state results were significant or not significant, but did not report test
✓International study
Notes:
If both early and late data reported, we counted each period once (study gets two counts, one for early, one for late)
If p-value > 0.05 we counted as no difference irrespective of magnitude of effect

Appendix 9: Acute Care Mortality Vote Count Table (Non-Pooled)

	Early ABF			Late ABF		
	Increase	Decrease	No diff/mixed	Increase	Decrease	No diff/mixed
Magnitude ≥ 5% and p ≤ .01						
Magnitude ≥ 1% to < 5% and p ≤ .01						
Magnitude <1% and p ≤ .01;<br>or<br>Mag.≥ 5% and stat sig. not reported or mixed						
Other results*	924✓<br>4917					2293<br>924✓

*Other results" refers to studies reporting combinations of magnitude and p-value other than the classifications in this table

✓International study

Appendix 10: Post-Acute Care Mortality Forest Plot

Study	Year	ABF	No ABF	Relative Risk (95% CI)
Qian	2011	*	*	1.47 (0.59, 3.69)
Carroll	1990	32/297	41/312	0.82 (0.53, 1.27)
Carroll	1987	13/157	28/190	0.56 (0.30, 1.05)
**Random Effects, p=0.32 for heterogeneity, I²=33.9%**				0.80 (0.52, 1.24)

*** total number of event=104 and total number of population at risk of death=1160**

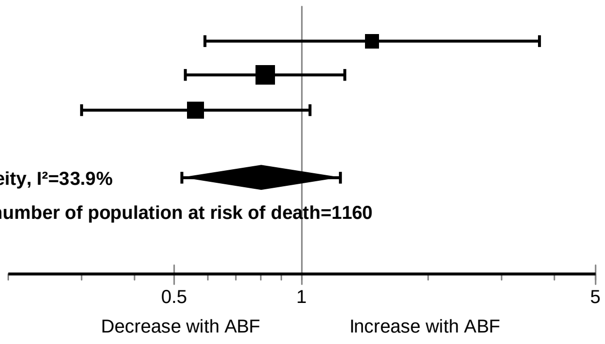

Appendix 11: Post-Acute Care Mortality Study Descriptions and Main Findings Table (Pooled and Non-Pooled)

Post-Acute Care Mortality Study Descriptions Table (Pooled)

**Author (year)**	**Country (start year ABF)**	**Study Design**	**Data Dates (% funding from ABF)***	**Primary data source**<br><br>**Sampling method**	**Study population**<br><br>**(N patients / N institutions)**	**Overall Credibility Score (0-6)**	**Variable and main findings** (outcome direction, magnitude, statistical significance)
**US**							
Qian (2011)<br><br>RefID 7634	USA (1983)	Before/After	No ABF:<br>1977-1982 (0%)<br><br>Late ABF:<br>1983-1992 (25-100%)	National Health and Nutrition Examination Survey (NHANES I) and NHANES I Epidemiologic Follow-up Study (NHEFS).<br><br>Convenience sample	Adults ≥ 65 years with complete information on variables used in analysis, Maryland data excluded.<br><br>4242/ns	4	**PAC mortality 31 days**<br><br>See forest plot
Carroll (1990)<br><br>RefID: 4265	USA (1983)	Before/after	No ABF:<br>1982-1983 (0%)<br><br>Late ABF:<br>1985-1986 (50-75%)	Data collected from medical records of seven Pennsylvania long-term care facilities for patients admitted August 1982 through July 1983 (pre-PPS) and August 1985 through July 1986 (post-PPS). All data abstracted from hospital transfer forms, physicians' or nurses' assessment forms and progress notes, state certification for level of care forms, and physicians' drug order forms.<br><br>Convenience sample	Medicare patients ≥ 65 years admitted directly from hospitals<br><br>609/7	1	**PAC mortality: 30 days**<br><br>See forest plot
Carroll (1987)<br><br>RefID: 4266	USA (1983)	Before/after	No ABF:<br>1982-1983 (0%)<br><br>Late ABF:<br>1984-1985 (25-50%)	Hospital transfer forms, physician or nurse assessment forms and progress notes, state certification for level of care forms, and physician drug order forms.<br><br>Convenience sample	Medicare patients ≥ 65 years discharged directly from hospital to long-term care facility, with hospital stay covered by Medicare.<br><br>353/10	1	**PAC mortality: 30 days**<br><br>See forest plot

*****US Medicare ABF Phase-in Schedule:
Beginning on or after October 1, 1983 and before October 1, 1984: 25% ABF; Beginning on or after October 1, 1984 and before October 1, 1985: 50% ABF; Beginning on or after October 1, 1985 and before October 1, 1986: 75% ABF; Beginning on or after October 1, 1986: 100% ABF; If the percentage of ABF changed during the data reporting period, we used the percent ABF present for the majority of the period.
**ns=not specified

Post-Acute Care Mortality Study Descriptions and Main Findings Table (Non-Pooled)

**Author (year)**	**Country (start year ABF)**	**Study Design**	**Data Dates (% funding from ABF)***	**Primary data source**<br><br>**Sampling method**	**Study population**<br><br>**(N patients / N institutions)**	**Overall Credibility Score (0-6)**	**Variable and main findings** (outcome direction, magnitude, statistical significance)
**US**							
Easton (1991)<br><br>RefID: 4900	USA (1983)	Before/after	No ABF:<br>1982-1983 (0%)<br><br>Late ABF:<br>1984-1987 (25-100%)	Patient charts, admitted to large Home Health Agency (HHA) over 5-year period.<br><br>Random sample	Elderly patients admitted to a large Home Health Agency.<br><br>329/1	1	**Mortality during first 2 weeks following admission to post-acute homecare services after discharge from acute care hospital:**<br><br>Before ABF vs. Late ABF: No deaths in either group<br>*(Outcome direction: no difference late, statistical significance not reported)*

***US Medicare ABF Phase-in Schedule:**
Beginning on or after October 1, 1983 and before October 1, 1984: 25% ABF; Beginning on or after October 1, 1984 and before October 1, 1985: 50% ABF; Beginning on or after October 1, 1985 and before October 1, 1986: 75% ABF; Beginning on or after October 1, 1986: 100% ABF; If the percentage of ABF changed during the data reporting period, we used the percent ABF present for the majority of the period.
Note: Studies stratified by US and International and ordered by credibility (highest to lowest) and then alphabetical by first author's last name.

Appendix 12: Post-Acute Care Mortality Analysis Table (Non-Pooled)

**Significance of Effect**	**Magnitude ≥5%***		**Magn ≥1% to <5%**		**Magn <1%**		**Magn Indeterminate or mixed**			
	**Direction**									
	**Inc**	**Dec**	**Inc**	**Dec**	**Inc**	**Dec**	**Inc**	**Dec**	**No diff**	**Mixed**
**Early ABF**										
p >.05										
p ≤ .05-.02										
p ≤ .01-.002										
p ≤ .001										
**Stat sig not reported										
**Late ABF**										
p >.05										
p ≤ .05-.02										
p ≤ .01-.002										
p ≤ .001										
**Stat sig not reported									4900	

* Formula for relative percent difference = (T2-T1/T1)(100)
**Abstracter judged increase, decrease, or no diff, but authors did not report statistical significance OR authors state results were significant or not significant, but did not report test
✓International study
Notes:
If both early and late data reported, we counted each period once (study gets two counts, one for early, one for late)
If p-value > 0.05 we counted as no difference irrespective of magnitude of effect

Appendix 13: Post-Acute Care Mortality Vote Count Table (Non-Pooled)

	Early ABF			Late ABF		
	**Increase**	**Decrease**	**No diff/mixed**	**Increase**	**Decrease**	**No diff/mixed**
Magnitude ≥ 5% and p ≤ .01						
Magnitude ≥ 1% to < 5% and p ≤ .01						
Magnitude <1% and p ≤ .01; or Mag.≥ 5% and stat sig. not reported or mixed						
Other results*						4900

*Other results" refers to studies reporting combinations of magnitude and p-value other than the classifications in this table

Appendix 14: Readmission Sub-Group Meta-Regression Table

Exploratory variables in meta-regression analysis		RR (95%CI)	p-value	R^2
US vs. International	US (n=8)	1.1 (0.86, 1.39)	0.48	0.05
	International (n=4)	0.97 (0.73, 1.29)		
Credibility	High (n=5)	1.08 (0.78, 1.5)	0.77	0.01
	Low (n=7)	1.02 (0.82, 1.28)		
Analysis	Adjusted (n=2)	0.98 (0.66, 1.47)	0.73	0.01
	Unadjusted (n=10)	1.06 (0.86, 1.31)		
Study design	Before-after (n=12)	1.04 (0.93, 1.16)	/	/
	Parallel groups	/		
ABF (time after implementation)	Early (<= 2 years; n=6)	1.05 (0.79, 1.41)	0.92	0.001
	Late (> 2 years; n=6)	1.03 (0.81, 1.32)		
Readmission measured over a period of	less than or equal to 30 days (n=7)	1.06 (0.83, 1.36)	0.77	0.01
	greater than 30 days (n=5)	1.01 (0.75, 1.35		

Appendix 15: Readmission Funnel Plot

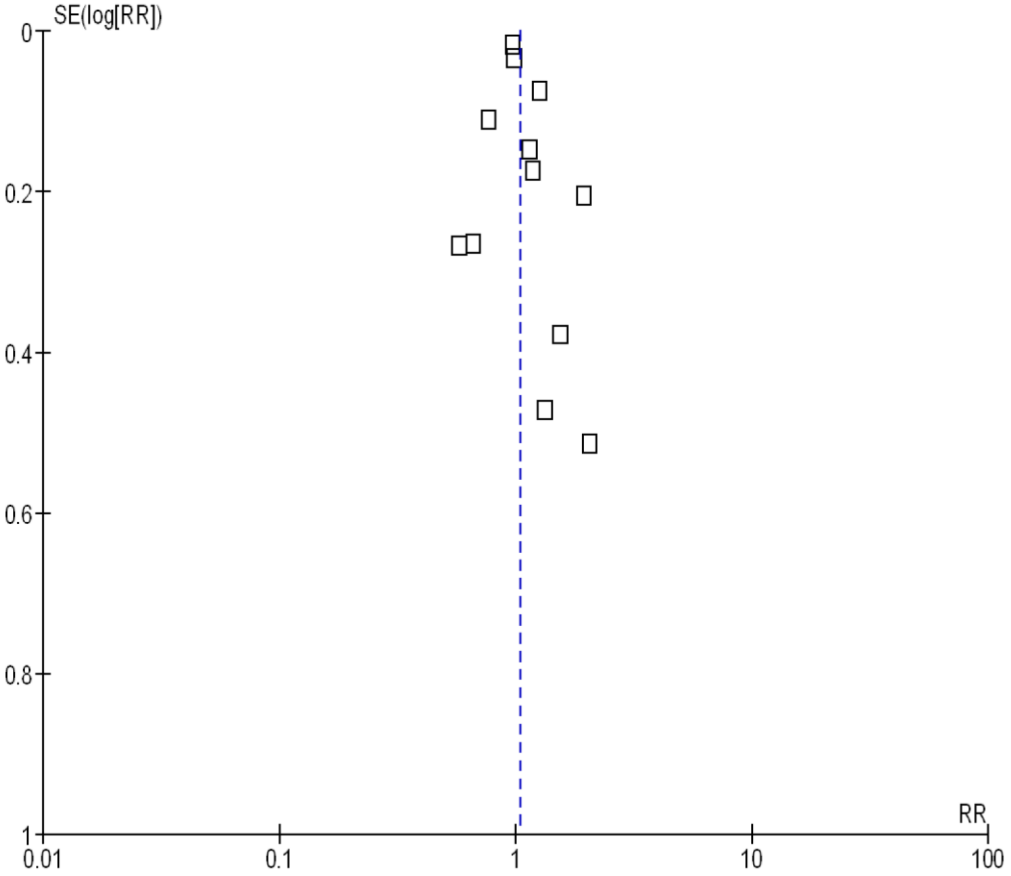

Appendix 16: Readmission Study Descriptions Table (Pooled)

Author (year)	Country (start year ABF)	Study Design	Data Dates (% funding from ABF)*	Primary data source<br><br>Sampling method	Study population<br><br>(N patients / N institutions)	Overall Credibility Score (0-6)	Variable and main findings (outcome direction, magnitude, statistical significance)
**US**							
Leibson (1991)<br><br>RefID: 1654	USA (1983)	Before/After	No ABF:<br>1980 (0%)<br><br>Late ABF:<br>1987 (100%)	Medical records in Olmsted County, Minnesota (Mayo Clinic and affiliated hospitals)<br><br>All eligible institutions in a jurisdiction	All patients ≥ 65 years<br><br>3710/5	6	**Readmission: 30 days**<br><br>See forest plot
Kahn (1990)<br><br>RefID: 1461	USA (1983)	Before/After	No ABF:<br>1981-1982 (0%)<br><br>Late ABF:<br>1985-1986 (50-75%)	Medical record as source of in-hospital mortality information and Health Care Financing Administration (HCFA) files. Also, Medicare's Part B files<br><br>Convenience sample	Medicare patients ≥ 65 years hospitalized 1981 through 1982 and 1985 with one or more of:<br>Congestive Heart Failure<br>Acute Myocardial Infarction<br>Pneumonia<br>Cerebrovascular accident<br>Hip Fracture<br><br>14012/ ns	4	**Readmission: 1 year**<br><br>See forest plot
Lewis (1987)<br><br>RefID: 1664	USA (1983)	Before/After	No ABF:<br>1980-1983 (0%)<br><br>Early ABF:<br>1984 (25%)	Medical records of SNF in Southern California<br><br>Random sample	Medicare patients ≥ 65 years admitted to SNF for the first time.<br><br>563/45	4	**Readmission: 60 days**<br><br>See forest plot
Weinberger (1988)<br><br>RefID: 8981	USA (1983)	Before/After	No ABF:<br>1981 (0%)<br><br>Early ABF:<br>1983-1984 (25-50%)	Individual medical records<br><br>All eligible institutions in a jurisdiction	Non-insulin dependent diabetics admitted to ward with admitting and discharge diagnosis of uncomplicated diabetes.<br><br>84/1	4	**Readmission: 1 year**<br><br>See forest plot
Rich (1988)<br><br>RefID: 7763	USA (1983)	Before/After	No ABF:<br>1983 (0%)<br><br>Early ABF:<br>1984 (25%)<br><br>Late ABF:<br>1986 (75%)	Computerized medical records database at Jewish Hospital, St. Louis, US<br><br>Convenience sample	All patients discharged from Jewish Hospital between January 1, 1983 through June 30th, 1986 who are ≥ 70 years with cardiovascular DRG codes, excluding those with prior cardiac admissions, and those who died during initial hospitalization (title specifies CHF patients).<br><br>410/1	2	**Readmission: 90 days**<br><br>See forest plot
Gay (1990)<br><br>RefID: 1049	USA (1983)	Before/After	No ABF:<br>1981 (0%)<br><br>Early ABF:<br>1984 (25%)	Medicare discharge abstracts from all reporting South Carolina, short-term, non-federal acute care hospitals<br><br>All eligible institutions in a jurisdiction	All Medicare patients<br><br>227771/68	1	**Readmission: 7 days**<br><br>See forest plot

Author (year)	Country (start year ABF)	Study Design	Data Dates (% funding from ABF)*	Primary data source<br><br>Sampling method	Study population<br><br>(N patients / N institutions)	Overall Credibility Score (0-6)	Variable and main findings (outcome direction, magnitude, statistical significance)
Carroll (1990)<br><br>RefID: 4265	USA (1983)	Before/After	No ABF:<br>1982-1983 (0%)<br><br>Late ABF:<br>1985-1986 (50-75%)	Records of seven long-term care facilities patients for August 1982 through July 1983 (pre-PPS) and August 1985 through July 1986 (post-PPS). All data were abstracted from hospital transfer forms, physicians' or nurses' assessment forms and progress notes, state certification for level of care forms, and physicians' drug order forms.<br><br>Convenience sample	Data collected from medical records of patients admitted to seven LTCFs in Pennsylvania. To be included in the sample, patients must have been admitted directly from hospitals and had their hospital stays reimbursed by Medicare.<br><br>609/7	1	**Readmission: 30 days**<br><br>See forest plot
Carroll (1987)<br><br>RefID: 4266	USA (1983)	Before/After	No ABF:<br>1982-1983 (0%)<br><br>Early:<br>1984-1985 (25-50%)	All data were abstracted from hospital transfer forms, physician or nurse assessment forms and progress notes, state certification for level of care forms, and physician drug order forms.<br><br>Convenience sample	Patients discharged directly from the hospital to the long-term care facility, and hospital stay covered by Medicare.<br><br>353/10	1	**Readmission: 30 days**<br><br>See forest plot
Cutler (1995)<br><br>RefID: 15603	USA (1983)	Before/After	No ABF:<br>1981-1983 (0%)<br><br>Early ABF:<br>1984-1985 (25-50%)<br><br>Late ABF:<br>1986-1988 (75-100%)	Final data contains about 1% of Medicare admissions (N=24,373) in six New England federal PPS states over eight year period; 16,308 admissions in the Massachusetts<br><br>Random sample	Medicare patients<br><br>30000/ ns	0	**Readmission: 30 days**<br><br>See forest plot
**International**							
Kerr (1998)<br><br>RefID: 1510	Australia (1993)	Before/After	No ABF:<br>1992 (0%)<br><br>Early ABF:<br>1993 (100%)	Randomized prospective study<br><br>Convenience sample	All patients admitted to the coronary care unit with uncomplicated, suspected unstable angina<br><br>336/1	4	**Readmission: 28 days**<br><br>**See forest plot**
South (1997)<br><br>RefID: 2524	Australia (1993)	Before/After	No ABF:<br>1989-1993 (0%)<br><br>Late ABF:<br>1993-1996 (?)	Prospective data collection<br><br>Convenience sample	All children discharged from Royal Children's Hospital general medical and thoracic units with primary diagnosis of asthma.<br><br>11939/1	3	**Readmission: 14 days**<br><br>See forest plot
Louis (1999)<br><br>RefID: 1719	Italy (1995)	Before/After	No ABF:<br>1993 (0%)<br><br>Early ABF:<br>1996 (ns)	Hospital discharge abstract data from 1993 through 1996 for all hospitals (N = 32) in Friuli-Venezia-Giulia region of Italy. Regional population data were used to calculate rates<br><br>All eligible institutions in a jurisdiction	not specified<br><br>ns/32	2	**Readmission: 30 days**<br><br>See forest plot
Brizioli (1996)<br><br>RefID: 477	Italy (1995)	Before/After	No ABF:<br>1994 (0%)<br><br>Early ABF:<br>1995 (100%)	Italian Health Ministry Official Reports Regione Marche Hospital Activity Annual Report, hospital specific data<br><br>Convenience sample	Elderly patients included in DRG127 (heart failure and shock)<br><br>1987/4	2	**Readmission: 90 days**<br><br>See forest plot

*****US Medicare ABF Phase-in Schedule:
Beginning on or after October 1, 1983 and before October 1, 1984: 25% ABF; Beginning on or after October 1, 1984 and before October 1, 1985: 50% ABF; Beginning on or after October 1, 1985 and before October 1, 1986: 75% ABF; Beginning on or after October 1, 1986: 100% ABF; If the percentage of ABF changed during the data reporting period, we used the percent ABF present for the majority of the period.
**ns=not specified

Appendix 17: Readmission Study Descriptions and Main Findings Table (Non-Pooled)

Author (year)	Country (start year ABF)	Study Design	Data Dates (% funding from ABF)*	Primary data source<br><br>Sampling method	Study population<br><br>(N patients / N institutions)	Overall Credibility Score (0-6)	Variable and main findings (outcome direction, magnitude, statistical significance)
**US**							
Gianfrancesco (1990)<br><br>RefID: 5320	USA (1983)	Before/After	No ABF:<br>1981-1983 (0%)<br><br>Late ABF:<br>1984-1986 (25-75%)	Tracer Discharge Episode Files.<br><br>Random sample<br><br>All eligible institutions in a jurisdiction	Medicare patients admitted with stroke, pneumonia, or for hip replacement.<br><br>32657/ns	4	60 day rehospitalization:<br><br>Before ABF vs. Late ABF: ABF predicted increased readmissions for stroke, but not for hip replacements (arthritis or fracture) or for pneumonia<br>*(Outcome summary: increase late, magnitude indeterminate, statistical significance not reported)*
DesHarnais (1991)<br><br>RefID: 4736	USA (1983)	Before/After	No ABF:<br>1983 (0%)<br><br>Early ABF:<br>1984 (25%)	Commission on Professional and Hospital Activities (CPHA) hospital abstracts, along with surveys done by the American Hospital Association.<br><br>Convenience sample	Not specified.<br><br>ns/245	2	30-day index of unanticipated readmissions for US Medicare:<br><br>Before ABF: 1.00<br>Early ABF: 1.06<br>*(Outcome summary: increase early, magnitude ≥ 5%, statistical significance not reported)*
Epstein (1991)<br><br>RefID: 897	USA (1986)	Before/After	No ABF:<br>1982-1985 (0%)<br><br>Early ABF:<br>1986 (75%)	Massachusetts Rate Setting Commission database, consisting of all acute care hospital discharges in the state from October 1, 1982 - September 30, 1986.<br><br>Additional sources of data: Massachusetts Department of Public Health (1984).<br><br>Convenience sample	Medicare patients ≥ 65years.<br><br>835871/73	2	% change in overall readmission rate within 30 days of discharge for all conditions:<br><br>Before ABF vs. Early ABF: No difference<br>*(Outcome summary: no difference early, statistical significance not reported)*
Gerety (1989)<br><br>RefID: 1058	USA (1983)	Before/After	No ABF:<br>1982-1984 (0%)<br><br>Late ABF:<br>1984-1986 (25-75%)	Patient medical charts.<br><br>Convenience sample	Patients ≥ 69 years with ICD or DRG codes for hip fracture admitted to Stanford University Medical Centre.<br><br>180/1	2	Number of readmissions to hospital within 1 year of fracture:<br><br>Before ABF vs. Late ABF: No difference<br>*(Outcome summary: no difference late, statistical significance not reported)*

Author (year)	Country (start year ABF)	Study Design	Data Dates (% funding from ABF)*	Primary data source<br><br>Sampling method	Study population<br><br>(N patients / N institutions)	Overall Credibility Score (0-6)	Variable and main findings (outcome direction, magnitude, statistical significance)
Qian (2011)<br><br>RefID: 7634	USA (1983)	Before/After	No ABF:<br>1977-1982 (0%)<br><br>Early ABF:<br>1983-1992 (25-100%)<br><br>Late ABF:<br>1986-1992 (75-100%)	National Health and Nutrition Examination Survey (NHANES I) and NHANES I Epidemiologic Follow-up Study (NHEFS).<br><br>Convenience sample	Adults ≥ 65 years with complete information on variables used in analysis, Maryland data excluded.<br><br>4242/ns	2	Readmission to hospital within 31 days following transition from hospital to nursing home (estimated HR):<br><br>Before ABF vs. Early ABF (31 days): 1.716<br>*(Outcome summary: increase early, magnitude ≥ 5%, p≥0.10)*<br><br>Before ABF vs. Late ABF (31 days): 0.756<br>*(Outcome summary: decrease late, magnitude ≥ 5%, p≥0.10)*<br><br>Readmission to hospital within 31 days following transition from hospital to community (estimated HR):<br><br>Before ABF vs. Early ABF (31 days): 1.013<br>*(Outcome summary: increase early, magnitude ≥ 1% to < 5%, p≥0.10)*<br><br>Before ABF vs. Late ABF (31 days): 0.856<br>*(Outcome summary: decrease late, magnitude ≥ 5%, p< .01)*<br><br>*Overall outcome summary: increase early, magnitude mixed, p≥0.10; decrease late, magnitude ≥ 5%, p value mixed*
Wells (1993)<br><br>RefID: 9035	USA (1983)	Before/After	No ABF:<br>1981-1982 (0%)<br><br>Late ABF:<br>1985-1986 (50-75%)	Primary: explicit and implicit information from patients' medical records.<br><br>Secondary: PPS Quality of Care study, and 1981-1982 and 1985-1986 Medpar files (HCFA).<br><br>Convenience sample	Sample of 2,746 US Medicare in-patients with first-list diagnosis of uni-polar or unspecified depression (ICD-9 Code) from 297 hospitals in five US states, excluding patients with at least one immediately life-threatening condition, or surgery during hospitalization.<br><br>2746/297	2	Readmission rate (%) within 1 year after discharge from acute care:<br><br>Before ABF: 60%<br>Late ABF: 52.4%<br>*(Outcome summary: decrease late, magnitude ≥ 5%, p <0.01)*
DesHarnais (1991)<br><br>RefID: 783	USA (1983)	Before/After	No ABF:<br>1980-1983 (0%)<br><br>Late ABF:<br>1984-1987 (25-100%)	Hospital abstract data files from the Professional Activity Study (PAS) data base of the Commission on Professional and Hospital Activities (CPHA).<br><br>Convenience sample	Medicare psychiatric patients in six vulnerable sub-groups discharged in the third quarters of 1980-1987, all with subsequent readmission to the same hospital.<br><br>Subgroup 1= patients more/less than 75 years old;<br><br>Subgroup 2=patients with/without comorbidities<br><br>Subgroup 3= patients with/without secondary diagnoses indicating additional psychiatric and/or substance abuse diagnoses<br><br>ns/151	1	**% patients readmitted to same hospital within 30 days of discharge (for six Medicare patient sub-groups according to age, presence of comorbidities, presence of any psychiatric or substance abuse secondary diagnoses):**<br><br>Before vs. Early ABF: Increase in 2 sub-groups, decrease in patients with psychiatric or substance abuse secondary diagnosis<br>*(Outcome summary: increase early, magnitude indeterminate, statistical significance not reported)*<br><br>Before vs. Late ABF: Decrease in 2 sub-groups, increase in patients with co-morbidities<br>*(Outcome summary: decrease late, magnitude indeterminate, statistical significance not reported)*

Author (year)	Country (start year ABF)	Study Design	Data Dates (% funding from ABF)*	Primary data source<br><br>Sampling method	Study population<br><br>(N patients / N institutions)	Overall Credibility Score (0-6)	Variable and main findings (outcome direction, magnitude, statistical significance)
Easton (1991)<br><br>RefID: 4900	USA (1983)	Before/After	No ABF:<br>1982-1983 (0%)<br><br>Late ABF:<br>1984-1987 (25-100%)	Patient charts, admitted to large Home Health Agency (HHA) over 5-year period.<br><br>Random sample	Elderly patients admitted to a large Home Health Agency.<br><br>329/1	1	14 day rehospitalization during the initial 2 weeks post-discharge:<br><br>Before ABF: 5.9%<br>Late ABF 5.5%<br>*(Outcome summary: decrease late, magnitude ≥ 5%, p≥ .05)*
Guterman (1988)<br><br>RefID: 5562	USA (1983)	Before/After + Parallel Groups	No ABF:<br>1981-1983 (0%)<br><br>Late ABF:<br>1984-1986 (25-75%)	Health Care Financing Administration (HCFA), National Centre for Health Statistics, American Hospital Association.<br><br>All eligible institutions in a jurisdiction	Not specified.<br><br>ns/ns	1	30 day readmission rate:<br><br>Before ABF vs. Late ABF: remained relatively stable in PPS states and non-PPS waiver states<br>*(Outcome summary: no difference late, statistical significance not reported)*
Fuchs (1986)<br><br>RefID: 5226	USA (1983)	Before/After	No ABF:<br>1982 (0%)<br><br>Early ABF:<br>1985 (50%)	National Nosocomial Infections Surveillance (NNIS).<br><br>Convenience sample	All patients in St. Vincent Hospital and Medical Centre, Portland, Oregon.<br><br>38872/1	0	Annual readmission for post-discharge nosocomial infections:<br><br>Before ABF: 0.29%<br>Early ABF: 0.26%<br>*(Outcome summary: decrease early, magnitude ≥ 5%, statistical significance not reported)*
**International**							
Farrar (2009)<br><br>RefID: 924	England and Scotland (2004 in England)	Before/After + Parallel Groups	No ABF:<br>2003-2004 (0%)<br><br>Early ABF:<br>2003-2005 (100%)<br><br>Late ABF:<br>2003-2006 (100%)	Hospital episode statistics (England, ABF-adopter).<br><br>Scottish morbidity records (Scotland, ABF non-adopter).<br><br>All eligible institutions in each jurisdiction	All patients admitted to acute care hospitals in England and Scotland.<br><br>ns/297	3	% difference-in-difference in hip fractures emergency readmission between England and Scotland:<br><br>No ABF vs. Early ABF: 0.73 % greater increase in hip fracture emergency readmission rate for ABF-adopters (England) as compared to non-ABF adopters (Scotland) between 2003-2004<br>*(Outcome summary: greater increase early, magnitude indeterminate, statistical significance not reported)*<br><br>No ABF vs. Late ABF: -1.20 % smaller increase in hip fracture emergency readmissions for ABF-adopters (England) as compared to non-ABF adopters (Scotland) between 2003-2005<br>*(Outcome summary: smaller increase late, magnitude indeterminate, statistical significance not reported)*
Xiao (2000)<br><br>RefID: 2859	Australia (1996)	Before/After	No ABF:<br>1991-1996 (0%)<br><br>Early ABF:<br>1996-1997 (100%)	Northern Territory aggregated Hospital Morbidity Databases, Australia.<br><br>Convenience sample	Patients admitted to five Northern Territory public hospitals, excluding renal dialysis patients.<br><br>ns/5	3	Readmission rates within 28 days of discharge (time series and intervention analysis), stratified among teaching and non-teaching hospitals:<br><br>Before ABF vs. Early ABF: No difference, though similar declining tendencies of about 1-2% reduction in readmission for teaching and non-teaching hospitals (generally higher rates for non-teaching hospitals, without apparent seasonality patterns)<br>*(Outcome summary: no difference early, statistical significance not reported)*
Frick (2001)<br><br>RefID: 1015	Austria (1997)	Before/After	No ABF:<br>1991-1996 (0%)<br><br>Early ABF:<br>1997-1998 (100%)	Complete hospital discharge statistics of the Salzburg province and residents from Salzburg hospitalized outside the province (compulsory by law).<br><br>All eligible institutions in a jurisdiction	All inpatients in psychiatry (regardless of specific diagnosis).<br><br>37215/ns	2	30 day readmission rate:<br><br>Before ABF vs. Early ABF: No difference<br>*(Outcome summary: no difference early, statistical significance not reported)*

Author (year)	Country (start year ABF)	Study Design	Data Dates (% funding from ABF)*	Primary data source<br><br>Sampling method	Study population<br><br>(N patients / N institutions)	Overall Credibility Score (0-6)	Variable and main findings (outcome direction, magnitude, statistical significance)
Shmueli (2002)<br><br>RefID: 8254	Israel (1990)	Before/After	No ABF:<br>1988-1990 (0%)<br><br>Early ABF:<br>1990-1991 (ns)	17,400 hospitalization records identified by relevant ICD-9 codes.<br><br>Convenience sample	Not specified.<br><br>ns/4	2	Rate of readmissions within 60 days (%):<br><br>Cholecysectomy<br>Before ABF: 5.2%<br>Early ABF: 9.9%<br>*(Outcome summary: increase early, magnitude ≥ 5%, statistical significance not reported)*<br><br>Hysterectomy<br>Before ABF: 6.3%<br>Early ABF: 7.0%<br>*(Outcome summary: increase early, magnitude ≥ 5%, statistical significance not reported)*<br><br>Hip replacement<br>Before ABF: 6.2%<br>Early ABF 10.1%<br>*(Outcome summary: increase early, magnitude ≥ 5%, statistical significance not reported)*<br><br>Operation on lens<br>Before ABF: 4.8%<br>Early ABF: 5.1%<br>*(Outcome summary: increase early, magnitude ≥ 5%, statistical significance not reported)*<br><br>Heart surgeries<br>Before ABF:4.8%<br>Early ABF: 7.7%<br>*(Outcome summary: increase early, magnitude ≥ 5%, statistical significance not reported)*<br><br>*Overall outcome summary: increase early, magnitude ≥ 5%, statistical significance not reported*
von Eiff (2011)<br><br>RefID: 8845	Germany (2004)	Before/After	No ABF:<br>2003-2004 (0%)<br><br>Late ABF:<br>2008-2009 (100%)	Prospective, multi-centered, national-wide study assessing the impact of ABF.<br><br>Convenience sample	Patients admitted in rehabilitation after cardiac (myocardial infarction, coronary bypass) or orthopedic procedure (hip or knee surgery).<br><br>1562/24	2	Orthopedics patients readmission rate:<br><br>Before ABF: 1.16%<br>Late ABF: 1.62%<br>*(Outcome summary: increase late, magnitude ≥ 5%, statistical significance not reported)*<br><br>Cardiology patients readmission rate:<br><br>Before ABF: 2.26%<br>Late ABF: 4.05%<br>*(Outcome summary: increase late, magnitude ≥ 5%, statistical significance not reported)*<br><br>*Overall outcome summary: increase late, magnitude ≥ 5%, statistical significance not reported*

Author (year)	Country (start year ABF)	Study Design	Data Dates (% funding from ABF)*	Primary data source<br><br>Sampling method	Study population<br><br>(N patients / N institutions)	Overall Credibility Score (0-6)	Variable and main findings (outcome direction, magnitude, statistical significance)
Busato (2010)<br><br>RefID: 4207	Switzerland (2003)	Parallel Groups	No ABF:<br>2003-2007 (0%)<br><br>Late ABF:<br>2003-2007 (ns)	All hospital discharges in Switzerland 2003-2007 (Swiss Federal Statistical Office) and on the complete claims data at the expense of basic health insurance of physicians in own practice for the same period.<br><br>All eligible institutions in a jurisdiction	All hospitalized patients except for those in psychiatry and rehabilitation institutions.<br><br>ns/ns	1	90 day re-hospitalization rate %:<br><br>No ABF vs. Late ABF: 13.45% higher<br>*(Outcome summary: increase late, magnitude ≥ 5%, statistical significance not reported)*
Researchers of the Italian Group of Pharmaco-epidemiology in the Aged (GIFA) (1986)<br><br>RefID: 3228	Italy (1994)	Before/After	No ABF:<br>1993 (0%)<br><br>Early ABF:<br>1995 (ns)	GIFA (Group of Pharmaco-epidemiology in the Aged).<br><br>All eligible institutions in a jurisdiction	Not specified<br><br>ns/83	1	Readmission rate %:<br><br>Before ABF: 1.3<br>Early ABF: 2.1<br>*(Outcome summary: increase early, magnitude ≥ 5%, statistical significance not reported)*

***US Medicare ABF Phase-in Schedule:**
Beginning on or after October 1, 1983 and before October 1, 1984: 25% ABF; Beginning on or after October 1, 1984 and before October 1, 1985: 50% ABF; Beginning on or after October 1, 1985 and before October 1, 1986: 75% ABF; Beginning on or after October 1, 1986: 100% ABF; If the percentage of ABF changed during the data reporting period, we used the percent ABF present for the majority of the period.
**ns=not specified
Note: Studies stratified by US and International and ordered by credibility (highest to lowest) and then alphabetical by first author's last name.

Appendix 18: Readmission Analysis Table (Non-Pooled)

Significance of Effect	Magnitude ≥5%*		Magn ≥1% to <5%		Magn <1%		Magn Indeterminate or mixed			
	Direction									
	Inc	Dec	Inc	Dec	Inc	Dec	Inc	Dec	No diff	Mixed
**Early ABF**										
p >.05							7634			
p ≤ .05-.02										
p ≤ .01-.002										
p ≤ .001										
**Stat sig not reported	3228✓<br>4736<br>8254✓	5226					783<br>924✓		897<br>2859✓<br>1015✓	
**Late ABF**										
p >.05		4900								
p ≤ .05-.02										
p ≤ .01-.002		9035								
p ≤ .001										
**Stat sig not reported	4207✓<br>8845✓						5320	783<br>924✓	1058<br>5562	
Stat sig mixed		7634								

* Formula for relative percent difference = (T2-T1/T1)(100)
**Abstracter judged increase, decrease, or no diff, but authors did not report statistical significance OR authors state results were significant or not significant, but did not report test
✓International study
Notes:
If both early and late data reported, we counted each period once (study gets two counts, one for early, one for late)
If p-value > 0.05 we counted as no difference irrespective of magnitude of effect

Appendix 19: Readmission Vote Count Table (Non-Pooled)

	Early ABF			Late ABF		
	Increase	Decrease	No diff/mixed	Increase	Decrease	No diff/mixed
Magnitude ≥ 5% and p ≤ .01					9035	
Magnitude ≥ 1% to < 5% and p ≤ .01						
Magnitude <1% and p ≤ .01;<br>or<br>Mag.≥ 5% and stat sig. not reported or mixed	3228✓<br>4736<br>8254✓	5226		4207✓<br>8845✓	7634	
Other results*	7634<br>924 ✓<br>783		897<br>2859✓<br>1015✓	5320	783<br>924✓	1058<br>5562<br>4900

*Other results" refers to studies reporting combinations of magnitude and p-value other than the classifications in this table

✓International study

Appendix 20: Discharge Destination Sub-Group Meta-Regression Table

Exploratory variables in meta-regression analysis		RR (95%CI)	p-value	R^2
US vs. International	US (n=19)	1.30 (1.18, 1.42)	0.04	0.20
	International (n=3)	1.01 (0.81, 1.25)		
Credibility	High (n=6)	1.30 (1.07, 1.58)	0.61	0.01
	Low (n=16)	1.23 (1.10, 1.37)		
Analysis	Adjusted (n=3)	1.25 (0.92, 1.70)	0.96	0.0002
	Unadjusted (n=19)	1.24 (1.12, 1.38)		
Study design	Before-after (n=21)	1.27 (1.16, 1.39)	0.0496	0.18
	Parallel groups (n=1)	0.86 (0.59, 1.26)		
ABF (Time after implementation)	Early (<= 2 years; n=13)	1.25 (1.12, 1.40)	0.80	0.003
	Late (> 2 years; n=9)	1.22 (1.02, 1.47)		

Appendix 21: Discharge Destination Funnel Plot

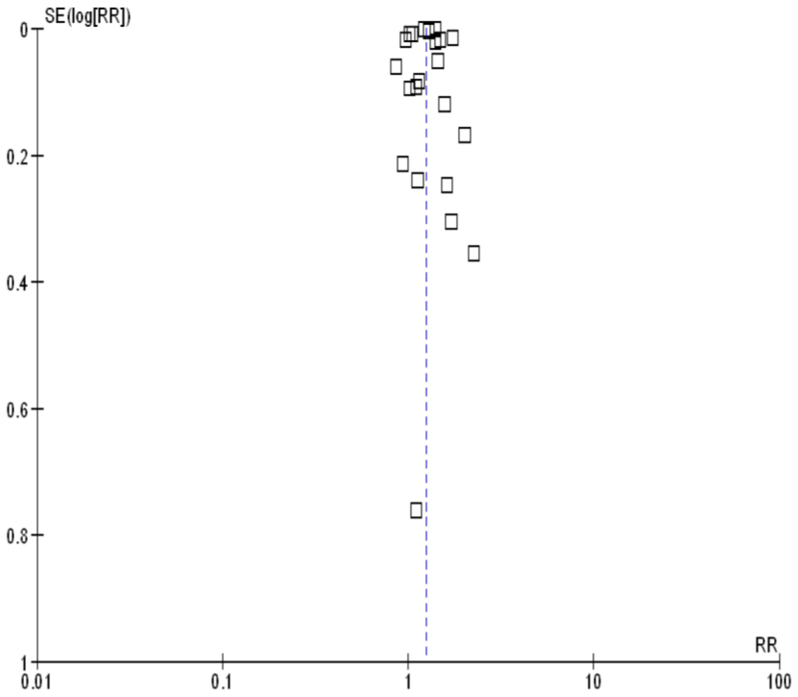

Appendix 22: Discharge Destination Study Descriptions Table (Pooled)

Author (year)	Country (start year ABF)	Study Design	Data Dates (% funding from ABF)*	Primary data source<br><br>Sampling method	Study population<br><br>(N patients / N institutions)	Overall Credibility Score (0-6)	Variable and main findings (outcome direction, magnitude, statistical significance)
**US**							
Leibson (1990)<br><br>RefID: 6510	USA (1983)	Before/after	No ABF:<br>1980 (0%)<br><br>Early ABF:<br>1985 (50%)<br><br>Late ABF:<br>1987 (100%)	Medical record of two Mayo affiliated hospital and Olmsted community hospital.<br><br>All eligible institutions in a jurisdiction	Patients ≥ 65 years discharged from 3 hospitals in Olmsted County<br><br>5854/3	5	**Discharge Destination:**<br><br>See forest plot
Hing (1989)<br><br>RefID: 5806	USA (1983)	Before/after	No ABF:<br>1982 (0%)<br><br>Early ABF:<br>1985 (50%)	1982-1985 National Hospital Discharge Survey (NHDS) and the current resident and discharged resident surveys of the 1977-1985 National Nursing Home Survey (NNHS)<br><br>All eligible institutions in a jurisdiction	Persons ≥ 65 using non-Federal short-stay hospitals and nursing homes<br><br>ns/ ns	4	**Discharge Destination:**<br><br>See forest plot
Fitzgerald (1988)<br><br>RefID: 5101	USA (1983)	Before/after	No ABF:<br>1981-1983 (0%)<br><br>Late ABF:<br>1984-1987 (25-100%)	1. Hospital records in a community hospital in a large Midwestern city.<br><br>2. Telephone interviews.<br><br>All eligible institutions in a jurisdiction	All patients ≥ 65 years old admitted to hospital with a new hip fracture between October 1, 1981 and March 1, 1986.<br><br>338/1	4	**Discharge Destination:**<br><br>See forest plot
Qian (2011)<br><br>RefID 7634	USA (1983)	Before/After	No ABF:<br>1977-1982 (0%)<br><br>Late ABF:<br>1983-1992 (25-100%)	National Health and Nutrition Examination Survey (NHANES I) and NHANES I Epidemiologic Follow-up Study (NHEFS).<br><br>Convenience sample	Adults ≥ 65 with complete information on variables used in analysis, Maryland data excluded.<br><br>4242/ns	4	**Discharge Destination:**<br><br>See forest plot
Fitzgerald (1987)<br><br>RefID: 5100	USA (1983)	Before/after	No ABF:<br>1981-1983 (0%)<br><br>Early ABF:<br>1984-1985 (25-50%)	Patient hospital charts.<br><br>All eligible institutions in a jurisdiction	All patients admitted to one Indianapolis tertiary hospital with a non-pathologic hip fracture<br><br>70/1	4	**Discharge Destination:**<br><br>See forest plot
Kanda (1991)<br><br>RefID: 6126	USA (1983)	Before/after	No ABF:<br>1980-1982 (0%)<br><br>Late ABF:<br>1985-1987 (50-100%)	Long term care facilities survey (LTCFS) conducted by State Health Data Centre, Pennsylvania Department of Health<br><br>All eligible institutions in a jurisdiction	Patients ≥ 65 years in Pennsylvania nursing homes<br><br>ns/1658	2	**Discharge Destination:**<br><br>See forest plot

Author (year)	Country (start year ABF)	Study Design	Data Dates (% funding from ABF)*	Primary data source<br><br>Sampling method	Study population<br><br>(N patients / N institutions)	Overall Credibility Score (0-6)	Variable and main findings (outcome direction, magnitude, statistical significance)
Kahn (1990)<br><br>RefID: 1461	USA (1983)	Before/after	No ABF:<br>1981-1982 (0%)<br><br>Late ABF:<br>1985-1986 (50-75%)	Medical record as source of in-hospital mortality information and Health Care Financing (HCFA) files. Also Medicare's Part B files<br><br>Convenience sample	Medicare patients hospitalized in 1981 through 1982 and 1985 with one or more of:<br>Congestive Heart Failure<br>Acute Myocardial Infarction<br>Pneumonia<br>Cerebrovascular accident<br>Hip Fracture<br><br>14012/ ns	2	**Discharge Destination:**<br><br>See forest plot
DesHarnais (1987)<br><br>RefID: 4737	USA (1983)	Before/after	No ABF:<br>1980 (0%)<br><br>Early ABF:<br>1984 (25%)	1. The Professional Activity Study of the CPHA<br>2. Computer tape from Annual Survey of Hospitals of American Hospital Association<br><br>Convenience sample	All discharges during 3rd quarter of each year from hospitals included<br><br>650596/729	2	**Discharge Destination:**<br><br>See forest plot
Schmidt (2002)<br><br>RefID: 8109	USA (1983)	Before/after	No ABF:<br>1981-1982 (0%)<br><br>Late ABF:<br>1995-1996 (100%)	1. Retrospective chart review of 1665 stroke patients discharged from 13 Cincinnati-area hospitals.<br><br>2. Retrospective chart review of medical records of all hospitalized stroke patients.<br><br>Convenience sample	Stroke patients discharged from 15 acute care hospitals in 1995-1996 compared with patients studied in the same geographic area in 1981-1982.<br><br>3534/15	2	**Discharge Destination:**<br><br>See forest plot
Rich (1988)<br><br>RefID: 7763	USA (1983)	Before/after	No ABF:<br>1983 (0%)<br><br>Early ABF:<br>1984 (25%)<br><br>Late ABF:<br>1986 (75%)	Computerized medical records database at Jewish Hospital, St. Louis, US<br><br>Convenience sample	All patients discharged from Jewish Hospital between January 1, 1983 through June 30th, 1986 $\geq$ 70 years with cardiovascular DRG codes, excluding those with prior cardiac admissions, and those who died during initial hospitalization.<br><br>410/1	2	**Discharge Destination:**<br><br>See forest plot
Manton (1990)<br><br>RefID: 1774	USA (1983)	Before/after	No ABF:<br>1982 (0%)<br><br>Early ABF:<br>1984 (25%)	1. National Centre for Health Statistics (NCHS) annual micro-level multiple cause mortality files<br>2. US Bureau of the Census population counts<br>3. National Technical Information Service (NLTCS) surveys<br><br>Convenience sample	not specified<br><br>ns/ ns	1	**Discharge Destination:**<br><br>See forest plot
Gay (1990)<br><br>RefID: 1049	USA (1983)	Before/after	No ABF:<br>1981 (0%)<br><br>Early ABF:<br>1984 (25%)	Medicare discharge abstracts from all reporting South Carolina, short-term, non-federal acute care hospitals<br><br>All eligible institutions in a jurisdiction	All Medicare patients<br><br>227771/68	1	**Discharge Destination:**<br><br>See forest plot

Author (year)	Country (start year ABF)	Study Design	Data Dates (% funding from ABF)*	Primary data source<br><br>Sampling method	Study population<br><br>(N patients / N institutions)	Overall Credibility Score (0-6)	Variable and main findings (outcome direction, magnitude, statistical significance)
Manton (1990)<br><br>RefID: 6774	USA (1983)	Before/after	No ABF:<br>1982-1983 (0%)<br><br>Early ABF:<br>1984-1985 (25-50%)	1982 and 1984 National long-term care surveys Health care financing Administration (HCFA) of disabled elderly Medicare beneficiaries and their Medicare part A bills and Medicare records on mortality.<br><br>Random sample	Nationally representative samples (~6000 people in each) of disabled non-institutionalized Medicare beneficiaries<br><br>6000/ ns	1	**Discharge Destination:**<br><br>See forest plot
Palmer (1989)<br><br>RefID: 7376	USA (1983)	Before/after	No ABF:<br>1981-1984 (0%)<br><br>Late ABF:<br>1984-1987 (25-100%)	Medical chart review<br><br>Random sample	Patients over ≥ 65 years hospitalized between January 1, 1981 and December 31, 1987, with newly diagnosed hip fractures from a teaching hospital in Indianapolis.<br><br>386/1	1	**Discharge Destination:**<br><br>See forest plot
Kane (1987)<br><br>RefID: 6128	USA (1983)	Before/after	No ABF:<br>1983-1984 (0%)<br><br>Early ABF:<br>1984-1985 (25-50%)	Medical records of patients admitted to rehab hospital<br><br>Convenience sample	not specified<br><br>516/1	1	**Discharge Destination:**<br><br>See forest plot
Mayer-Oakes (1988)<br><br>RefID: 6863	USA (1983)	Before/after	No ABF:<br>1981-1982 (0%)<br><br>Early ABF:<br>1984-1985 (25-50%)	MICU log books, medical record.<br><br>Convenience sample	Medicare patients ≥ 65 and comparison patients aged 50-64, not admitted to MICUs during the study period. Medicare patients < 65 years old, primarily those with end stage renal disease, were excluded given oversampling of the group.<br><br>398/3	1	**Discharge Destination:**<br><br>See forest plot
Long (1987)<br><br>RefID: 1711	USA (1983)	Before/after	No ABF:<br>1980-1983 (0%)<br><br>Early ABF:<br>1984 (25%)	Third-quarter cohort in the Professional Activity Study of the Commission on Professional and Hospital Activities (CPHA).<br><br>Convenience sample	Medicare patients<br><br>ns/729	0	**Discharge Destination:**<br><br>See forest plot
DesHarnais (1988)<br><br>RefID: 4735	USA (1983)	Before/after	No ABF:<br>1980 (0%)<br><br>Early ABF:<br>1984 (25%)<br><br>Late ABF:<br>1985 (50%)	Primary source: The Professional Activity Study (PAS) of the Commission on Professional and Hospital Activities (CPHA)<br><br>American Hospital Association's Annual Survey of Hospitals<br><br>Convenience sample	Medicare patients ≥ 65 years where Medicare is designated as the principal source of payment on hospital abstract<br><br>Non-Medicare patients: Patients < 65 where payment sources other than Medicare designated on hospital abstract<br><br>ns/646	0	**Discharge Destination:**<br><br>See forest plot
Pfeiffer (1987)<br><br>RefID: 7496	USA (1983)	Parallel groups	No ABF:<br>1981-1984 (0%)<br><br>Early ABF:<br>1981-1984 (25%)	Health Care Financing Administration (HCFA) data source for 5 representative states<br><br>Convenience sample	Sample from 5 representative states of 18 DRGs most closely related to long term care plus 9 DRGs most closely related to long term care in Massachusetts. 57% women, 5% <60 years old, 8% less than 65 years old, 45% <75 years old, 83% less than 83%.<br><br>9895/ ns	0	**Discharge Destination:**<br><br>See forest plot
**International**							

Author (year)	Country (start year ABF)	Study Design	Data Dates (% funding from ABF)*	Primary data source<br><br>Sampling method	Study population<br><br>(N patients / N institutions)	Overall Credibility Score (0-6)	Variable and main findings (outcome direction, magnitude, statistical significance)
Strömberg (1997)<br><br>RefID: 8497	Sweden (1992)	Before/after	No ABF:<br>1990 (0%)<br><br>Early ABF:<br>1992 (ns)	Computerized diagnosis-based inpatient system<br><br>Convenience sample	Patients ≥ 65 years at time of injury, residents of the City of Stockholm, admitted from independent living situation, initially treated in orthopedic departments in 4 Stockholm hospitals.<br><br>2238/4	4	**Discharge Destination:**<br><br>See forest plot
Jauss (2010)<br><br>RefID: 1421	Germany (2004)	Before/after	No ABF:<br>2003 (0%)<br><br>Early ABF:<br>2004 (100%)<br><br>Late ABF:<br>2006 (100%)	Administrative database of the German state of Hessen (6.1 million inhabitants) including all patients with stroke.<br><br>All eligible institutions in a jurisdiction	All patients diagnosed with ischemic stroke (excluding transitory ischemic stroke, hemorrhagic stroke).<br><br>27005/ ns	1	**Discharge Destination:**<br><br>See forest plot
Onder (2001)<br><br>RefID: 7313	Italy (1994)	Before/after	No ABF:<br>1988-1993 (0%)<br><br>Late ABF:<br>1995-1998 (100%)	Gruppo Italiano di Farmacoepidemiologia nell'Anziano (GIFA) database<br><br>Convenience sample	All patients ≥ 65 with pneumonia admitted to 40 medical centres distributed throughout Italy<br><br>963/40	1	**Discharge Destination:**<br><br>See forest plot

*****US Medicare ABF Phase-in Schedule:
Beginning on or after October 1, 1983 and before October 1, 1984: 25% ABF; Beginning on or after October 1, 1984 and before October 1, 1985: 50% ABF; Beginning on or after October 1, 1985 and before October 1, 1986: 75% ABF; Beginning on or after October 1, 1986: 100% ABF; If the percentage of ABF changed during the data reporting period, we used the percent ABF present for the majority of the period.
**ns=not specified

Appendix 23: Discharge Destination Study Descriptions and Main Findings Table (Non-Pooled)

Author (year)	Country (start year ABF)	Study Design	Data Dates (% funding from ABF)*	Primary data source<br><br>Sampling method	Study population<br><br>(N patients / N institutions)	Overall Credibility Score (0-6)	Variable and main findings (outcome direction, magnitude, statistical significance)
**US**							
Gianfrancesco (1990)<br><br>RefID: 5320	USA (1983)	Before/After	No ABF:<br>1981-1983 (0%)<br><br>Late ABF:<br>1984-1986 (25-75%)	Tracer Discharge Episode Files.<br><br>Random sample<br><br>All eligible institutions in a jurisdiction	Medicare patients admitted with stroke, pneumonia, or for hip replacement.<br><br>32657/ns	4	SNF admissions following discharge from acute hospital:<br><br>Before ABF vs. Late ABF: increase stroke and hip replacement (fracture and arthritis), but not pneumonia.<br>*(Outcome summary: increase late, magnitude indeterminate, statistical significance not reported)*<br><br>HHA admissions following discharge from acute hospital:<br><br>Before ABF vs. Late ABF: increase pneumonia and hip replacement (arthritis), but not stroke or hip replacement (fracture)<br>*(Outcome summary: mixed late, magnitude indeterminate, statistical significance not reported)*<br><br>*Overall outcome summary: mixed late, magnitude indeterminate, statistical significance not reported*
Long (1990)<br><br>RefID: 6621	USA (1983)	Before/After	No ABF:<br>1983 (0%)<br><br>Early ABF:<br>1984 (25%)	The Quality of Care Data File of the Commission of Professional and Hospital Activities (CPHA).<br><br>Convenience sample	All patients with complete data between 1980-1985 admitted to 646 non-Federal short-term hospitals.<br><br>ns/646	4	Change in "hospital products index":<br><br>Before vs. Early ABF: Proportion of patients discharged to home self-care fell by 3.7 index points in the first year after ABF. Discharge to all other locations rose as follows: +7.7 to short term hospital; +10.5 to skilled nursing facility/intermediate care; +79.6 home health agency; +0.5 dead.<br>*(Outcome summary: increase early, magnitude ≥ 5%, statistical significance not reported)*

Author (year)	Country (start year ABF)	Study Design	Data Dates (% funding from ABF)*	Primary data source<br><br>Sampling method	Study population<br><br>(N patients / N institutions)	Overall Credibility Score (0-6)	Variable and main findings (outcome direction, magnitude, statistical significance)
Long (1989)<br><br>RefID: 1712	USA (1983)	Before/After	No ABF:<br>1983 (0%)<br><br>Early ABF:<br>1984 (25%)	The Quality of Care Data File of the Commission on Professional and Hospital Activities (CPHA).<br><br>Convenience sample	US Medicare patients.<br><br>ns/646	3	% discharged to any post acute care:<br><br>Before ABF vs. Early ABF:<br>40 DRGs - increase<br>7 DRGs - unchanged<br>2 DRGs – decrease<br>*(Outcome summary: increase early, magnitude indeterminate, statistical significance not reported)*<br><br>% discharged to home health agencies (HHA):<br><br>Before ABF vs. Early ABF:<br>45 DRGs - increase<br>(*Outcome summary: increase early, magnitude indeterminate, statistical significance not reported)*<br><br>% discharged to skilled nursing facility (SNF) or intermediate care facility (ICF):<br><br>Before ABF vs. Early ABF:<br>22 DRGs - increase<br>24 DRGs – unchanged<br>*(Outcome summary: mixed early, magnitude indeterminate, statistical significance not reported)*<br><br>% transferred to another short term hospital:<br><br>Before ABF vs. Early ABF:<br>9 DRGs – decrease<br>35 DRGs – unchanged<br>*(Outcome summary: no difference early, magnitude indeterminate, statistical significance not reported)*<br><br>*Overall outcome summary: mixed early, magnitude indeterminate, statistical significance not reported*
Wells (1993)<br><br>RefID: 9035	USA (1983)	Before/After	No ABF:<br>1981-1982 (0%)<br><br>Late ABF:<br>1985-1986 (50-75%)	Primary: explicit and implicit information from patients' medical records.<br><br>Secondary: PPS Quality of Care study, and 1981-1982 and 1985-1986 Medpar files (HCFA).<br><br>Convenience sample	Sample of 2,746 US Medicare in-patients with first-list diagnosis of unipolar or unspecified depression (ICD-9 Code) from 297 hospitals in five US states, excluding patients with at least one immediately life-threatening condition, or surgery during hospitalization.<br><br>2746/297	3	% Patients admitted to hospital from home or retirement home and discharged to hospital or nursing home:<br><br>Before ABF: 15.5%<br>Late ABF: 9.0%<br>*(Outcome summary: decrease late, magnitude ≥ 5%, $p<0.05$)*
Friedman (1995)<br><br>RefID: 1017	USA (1983)	Before/After	No ABF:<br>1980-1983 (0%)<br><br>Late ABF:<br>1984-1987 (25-100%)	The Hospital Cost and Utilization Project (HCUP) database.<br><br>Convenience sample	US Medicare patients.<br><br>ns/415	2	Risk-weighted discharge rate to long-term care facilities (RWDIS-L):<br><br>Before ABF vs. Late ABF: 40% increase, statistical significance not reported<br>*(Outcome summary: increase late, magnitude ≥ 5%, statistical significance not reported)*

Author (year)	Country (start year ABF)	Study Design	Data Dates (% funding from ABF)*	Primary data source<br><br>Sampling method	Study population<br><br>(N patients / N institutions)	Overall Credibility Score (0-6)	Variable and main findings (outcome direction, magnitude, statistical significance)
DesHarnais (1990)<br><br>RefID: 4740	USA (1983)	Before/After	No ABF:<br>1980-1983 (0%)<br><br>Early ABF:<br>1984 (25%)<br><br>Late ABF:<br>1987 (100%)	The Professional Activity Study (PAS) database of the Commission on Professional and Hospital Activities (CPHA).<br><br>Convenience sample	All patients with a psychiatric disease admitted to the qualifying hospitals.<br><br>ns/386	1	Change in % patients discharged to skilled nursing facilities, other type of hospital, home health, "other" for Medicare patients:<br><br>Before ABF: discharge to SNF 12.5%; to short term hospital 2.3%; to other type of hospital 9.2%; to HHA 2.1%.<br><br>Early ABF: discharge to SNF 8.8%; to short term hospital 4.4%; other type of hospital 12%; to HHA 3.3%.<br><br>Late ABF: discharge to SNF 8.3%; to short term hospital 5.0%; to other type of hospital 11.9%; to HHA 3.3%<br><br>*Outcome summary: increase early, increase late, magnitude ≥ 5%, statistical significance not reported*
DesHarnais (1991)<br><br>RefID: 783	USA (1983)	Before/After	No ABF:<br>1980-1983 (0%)<br><br>Late ABF:<br>1984-1987 (25-100%)	Hospital abstract data files from the Professional Activity Study (PAS) data base of the Commission on Professional and Hospital Activities (CPHA).<br><br>Convenience sample	Medicare psychiatric patients in three vulnerable sub-groups discharged in the third quarters of 1980-1987, all with subsequent readmission to the same hospital.<br><br>Subgroup 1= patients more than 75 years old;<br><br>Subgroup 2=patients with comorbidities.<br><br>Subgroup 3= patients with secondary diagnoses indicating additional psychiatric and/or substance abuse diagnoses.<br><br>ns/151	1	Discharge location for 3 sub-groups (age, comorbidities, psychiatric patients):<br><br>Before ABF vs. late ABF: for all three subgroups, increase "home with home health services", increase "other hospitals"; decrease "skilled nursing facilities" (SNF). Greatest effect: increase discharge to "other hospitals".<br><br>*(Outcome summary: mixed late, magnitude indeterminate, statistical significance not reported)*
Gaumer (1992)<br><br>RefID: 5293	USA (1983)	Before/After + Parallel Groups	No ABF:<br>1982 (0%)<br><br>Early ABF:<br>1984 (25%)<br><br>Late ABF:<br>1986 (75%)	5 % Medicare eligibility file.<br><br>Random sample	US Medicare patients with valid indicator of the date of death, Part A entitlement during the year of death, no indication of enrollment in a Medicare hospice program or health maintenance organization in the year of death, and residence in Washington, D.C. or one of the contiguous states.<br><br>34576/ns	1	Percent discharged to homecare in no ABF vs. ABF states:<br><br>Before ABF: 17.2% vs. 13.2%<br>Early ABF: 17.0 % vs. 17.4%<br>Late ABF: 19.5% % vs. 18.7%<br>*(Outcome summary: increase early in ABF states, magnitude ≥ 5%, statistical significance not reported; increase late in ABF states, magnitude ≥ 5%, statistical significance not reported)*<br><br>Percent discharged to nursing facilities in no ABF vs. ABF states:<br><br>Before ABF: 4.1% vs. 6.0%<br>Early ABF: 3.8% vs. 7.0%<br>Late ABF: 3.9% vs. 6.6%<br>*(Outcome summary: increase early and late in ABF states, magnitude ≥ 5%, statistical significance not reported)*<br><br>*Overall outcome summary: increase early and late in ABF states, magnitude ≥ 5%, statistical significance not reported*

Author (year)	Country (start year ABF)	Study Design	Data Dates (% funding from ABF)*	Primary data source<br><br>Sampling method	Study population<br><br>(N patients / N institutions)	Overall Credibility Score (0-6)	Variable and main findings (outcome direction, magnitude, statistical significance)
Gay (1994)<br><br>RefID: 5295	USA (1983)	Before/After	No ABF:<br>1981-1982 (0%)<br><br>Early ABF:<br>1983-1985 (25-50%)	4th quarter Medicare discharge summaries from all reporting South Carolina, short-term, non-federal, acute-care hospitals.<br><br>All eligible institutions in a jurisdiction	Medicare patients from South Carolina acute care hospitals.<br><br>ns/68	1	% of market discharged to HHS:<br><br>Before ABF: 1.74%<br>Early ABF: 5.10%<br>*(Outcome summary: increase early, magnitude ≥ 5%, statistical significance not reported)*
Guterman (1988)<br><br>RefID: 5562	USA (1983)	Before/After + Parallel Groups	No ABF:<br>1981-1983 (0%)<br><br>Late ABF:<br>1984-1986 (25-75%)	Health Care Financing Administration (HCFA), National Centre for Health Statistics, American Hospital Association.<br><br>All eligible institutions in a jurisdiction	Not specified.<br><br>ns/ns	1	Percent chance patient goes into SNF for 7 days or less:<br><br>Before ABF: 0.41%<br>Late ABF: 0.65%<br>*(Outcome summary: increase late, magnitude ≥ 5%, statistical significance not reported)*<br><br>Percent chance patient goes into SNF for 30 days or more:<br><br>Before ABF: 0.86%<br>Late ABF: 0.88%<br>*(Outcome summary: increase late, magnitude ≥ 1% to < 5%, statistical significance not reported)*<br><br>*Overall outcome summary: increase late, magnitude mixed, statistical significance not reported*
Manton (1993)<br><br>RefID: 1775	USA (1983)	Before/After	No ABF:<br>1982 (0%)<br><br>Early ABF:<br>1984 (25%)	National Long Term Care Surveys (NLTCS) linked to Medicare Part A administrative records.<br><br>Random sample	All Medicare patients.<br><br>ns/ns	1	Proportion discharge to PAC (% SNF and % HHA):<br>Before ABF: 16.7%<br>Early ABF: 20.0%<br><br>*(Outcome summary: increase early, magnitude ≥ 5%, statistical significance not reported)*
Morrisey (1988)<br><br>RefID: 1953	USA (1983)	Before/After	No ABF:<br>1980-1983 (0%)<br><br>Early ABF:<br>1984-1985 (25-50%)	Discharge abstracts submitted to the Commission on Professional and Hospital Activities (CPHA) & American Hospital Association Annual Survey data.<br><br>Convenience sample	Five DRG groups: specific cerebrovascular disorders except transient ischemic attacks (DRG14), COPD (DRG 88), simple pneumonia or pleurisy (DRG 89-91), heart failure and shock (DRG 127), major joint procedures (DRG 209-212).<br><br>ns/501	1	Adjusted probability of transfer to PAC (SNF, ICF, HH):<br><br>Before vs. Early ABF: increase 3-14% absolute (corresponding relative increase >75%) for all DRGs (DRG 14, 88, 89-91,127, 209-212) and all discharge destinations, particularly SNF.<br><br>*(Outcome summary: increase early, magnitude ≥ 5%, p<0.01)*
Sloan (1988)<br><br>RefID: 2487	USA (1983)	Before/After	No ABF:<br>1980-1983 (0%)<br><br>Early ABF:<br>1984 (25%)<br><br>Late ABF:<br>1985 (50%)	Discharge abstracts submitted to the Commission on Professional and Hospital Activities (CPHA) on a sample of 467 hospitals.<br><br>Convenience sample	Medicare and non-Medicare patients.<br><br>ns/467	1	% Patients transferred to another hospital:<br><br>Medicare:<br>Before ABF: 1.9<br>Early ABF: 1.0<br>Late ABF: 2.2<br>*(Outcome summary: Decrease early, magnitude ≥ 5%, statistical significance not reported; increase late, magnitude ≥ 5%, statistical significance not reported)*

Author (year)	Country (start year ABF)	Study Design	Data Dates (% funding from ABF)*	Primary data source<br><br>Sampling method	Study population<br><br>(N patients / N institutions)	Overall Credibility Score (0-6)	Variable and main findings (outcome direction, magnitude, statistical significance)
Van Gelder (1986)<br><br>RefID: 8785	USA (1983)	Before/After	No ABF:<br>1983 (0%)<br><br>Early ABF:<br>1984 (25%)	Draft report of the congressionally mandated first-year study of the impact of Medicare PPS, Department of Health and Human Services (HHS).<br>2. Health Care Financing Administration (HCFA).<br><br>Convenience sample	Not specified.<br><br>ns/ns	0	Rate of admissions to Home Health Care:<br><br>Before ABF vs. Early ABF: 45% increase<br>*(Outcome summary: increase early, magnitude ≥ 5%, statistical significance not reported)*

*****US Medicare ABF Phase-in Schedule:
Beginning on or after October 1, 1983 and before October 1, 1984: 25% ABF; Beginning on or after October 1, 1984 and before October 1, 1985: 50% ABF; Beginning on or after October 1, 1985 and before October 1, 1986: 75% ABF; Beginning on or after October 1, 1986: 100% ABF; If the percentage of ABF changed during the data reporting period, we used the percent ABF present for the majority of the period.
**ns=not specified
Note: Studies stratified by US and International and ordered by credibility (highest to lowest) and then alphabetical by first author's last name.

Appendix 24: Discharge Destination Analysis Table (Non-Pooled)

Significance of Effect	Magnitude ≥5%*		Magn ≥1% to <5%		Magn <1%		Magn Indeterminate or mixed			
	Direction									
	Inc	Dec	Inc	Dec	Inc	Dec	Inc	Dec	No diff	Mixed
**Early ABF**										
p >.05										
p ≤ .05-.02										
p ≤ .01-.002	1953									
p ≤ .001										
**Stat sig not reported	5295<br>6621<br>8785<br>1775<br>5293<br>4740	2487								1712
**Late ABF**										
p >.05										
p ≤ .05-.02		9035								
p ≤ .01-.002										
p ≤ .001										
**Stat sig not reported	2487<br>1017<br>5293<br>4740						5562			783<br>5320

* Formula for relative percent difference = (T2-T1/T1)(100)

**Abstracter judged increase, decrease, or no diff, but authors did not report statistical significance OR authors state results were significant or not significant, but did not report test

Notes:

If both early and late data reported, we counted each period once (study gets two counts, one for early, one for late)

If p-value > 0.05 we counted as no difference irrespective of magnitude of effect

Appendix 25: Discharge Destination Vote Count Table (Non-Pooled)

	Early ABF			Late ABF		
	Increase	Decrease	No diff/mixed	Increase	Decrease	No diff/mixed
Magnitude ≥ 5% and p ≤ .01	1953					
Magnitude ≥ 1% to < 5% and p ≤ .01						
Magnitude <1% and p ≤ .01;<br>or<br>Mag.≥ 5% and stat sig. not reported or mixed	5295<br>6621<br>8785<br>1775<br>5293<br>4740	2487		2487<br>1017<br>5293<br>4740		
Other results*			1712	5562	9035	5320<br>783

*Other results" refers to studies reporting combinations of magnitude and p-value other than the classifications in this table

Appendix 26: Severity of Illness Study Descriptions and Main Findings Table (Non-Pooled)

Author (year)	Country (start year ABF)	Study Design	Data Dates (% funding from ABF)*	Primary data source<br><br>Sampling method	Study population<br><br>(N patients / N institutions)	Overall Credibility Score (0-6)	Variable and main findings (outcome direction, magnitude, statistical significance)
**US**							
Fitzgerald (1987)<br><br>RefID: 5100	USA (1983)	Before/after	No ABF:<br>1981-1983 (0%)<br><br>Early ABF: 1984-1985 (25-50%)	Patient hospital charts.<br><br>All eligible institutions in a jurisdiction	All patients admitted to one Indianapolis tertiary hospital with a non-pathologic hip fracture.<br><br>70/1	6	**Fracture type, comorbidities, complications, and pre-fracture ambulation:**<br><br>Before ABF vs. Early ABF: No significant difference<br>*(Outcome summary: no difference early, p =.29-.68)*
Fitzgerald (1988)<br><br>RefID: 5101	USA (1983)	Before/after	No ABF:<br>1981-1983 (0%)<br><br>Late ABF: 1984-1986 (25-75%)	3. Hospital records in a community hospital in a large Midwestern city.<br><br>4. Telephone interviews.<br><br>All eligible institutions in a jurisdiction	All patients >65 years old admitted to hospital with a new hip fracture between October 1, 1981 and March 1, 1986.<br><br>338/1	6	**Percent with pre-existing conditions (none, cardiovascular disease, dementia, stroke, independent pre-fracture ambulation):**<br><br>Before ABF vs. Late ABF: No difference<br>*(Outcome summary: no difference late, statistical significance not reported)*
Hing (1989)<br><br>RefID: 5806	USA (1983)	Before/after	No ABF:<br>1977 (0%)<br><br>Early ABF: 1985 (50%)	1982-1985 National Hospital Discharge Survey (NHDS) and the current resident and discharged resident surveys of the 1977-1985 National Nursing Home Survey (NNHS).<br><br>All eligible institutions in a jurisdiction	Persons aged 65 years and over utilizing non-Federal short-stay hospitals and nursing homes.<br><br>ns/ns	6	**Primary diagnosis upon admission to nursing home:**<br><br>malignant neoplasms:<br>Before ABF: 4.1%<br>Early ABF: 3.3%<br><br>mental disorder:<br>Before ABF: 11.0%<br>Early ABF: 15.4%<br><br>diseases of the circulatory system:<br>Before ABF: 41.5%<br>Early ABF: 33.8%<br><br>fractures of the neck of femur:<br>Before ABF: 2.9%<br>Early ABF: 4.4%<br><br>Average number of dependencies in activities of daily living:<br>Before ABF: 3.7%<br>Early ABF: 4.0%<br>*(Outcome summary: mixed early, magnitude ≥ 5%, statistical significance not reported)*

Author (year)	Country (start year ABF)	Study Design	Data Dates (% funding from ABF)*	Primary data source<br><br>Sampling method	Study population<br><br>(N patients / N institutions)	Overall Credibility Score (0-6)	Variable and main findings (outcome direction, magnitude, statistical significance)
Leibson (1991)<br><br>RefID: 1654	USA (1983)	Before/after	No ABF:<br>1980 (0%)<br><br>Late ABF: 1987 (100%)	Medical records in Olmsted County, Minnesota (Mayo Clinic and affiliated hospitals).<br><br>All eligible institutions in a jurisdiction	All Medicare patients 65 years of age or older.<br><br>3710/5	6	**Complexity index (%>2 score):**<br><br>Before ABF: 17.2<br>Late ABF: 22.7<br>*(Outcome summary: increase late, magnitude ≥ 5%; statistical significance not reported)*<br><br>**Diagnostic cardiac procedure (%):**<br><br>Before ABF: 1.3<br>Late ABF: 6.1<br>*(Outcome summary: increase late, magnitude ≥ 5%; statistical significance not reported)*<br><br>**Chronic diagnosis (%):**<br><br>Before ABF: 32.0<br>Late ABF: 35.8<br>*(Outcome summary: increase late, magnitude ≥ 5%; statistical significance not reported)*<br><br>*Overall outcome summary: increase late, magnitude ≥ 5%; statistical significance not reported*
Lewis (1987)<br><br>RefID: 1664	USA (1983)	Before/after	No ABF:<br>1980-1983 (0%)<br><br>Early ABF: 1984 (25%)	Medical records of SNF in Southern California.<br><br>Random sample	Medicare patients admitted to SNF for the first time.<br><br>563/45	6	**Functional status of patients transferred from hospitals to SNFs (comatose, bed confined, continent):**<br><br>Before ABF vs. Early ABF: no difference<br>*(Outcome summary: no difference early, statistical significance not reported)*<br><br>**Diagnosis for patients transferred from hospitals to SNFs (Cancer, Cerebral vascular accident, dementia, hip fracture):**<br><br>Before ABF vs. Early ABF: no difference, except CVA<br>*(Outcome summary: no difference early, (except CVA increase p<.005)*<br><br>*Overall outcome summary: no difference early, statistical significance mixed*
Weinberger (1988)<br><br>RefID: 8981	USA (1983)	Before/after	No ABF:<br>1981 (0%)<br><br>Early ABF: 1983-1984 (25-50%)	Individual medical records.<br><br>All eligible institutions in a jurisdiction	Non-insulin dependent diabetics admitted to the ward with an admitting and discharge diagnosis of uncomplicated diabetes.<br><br>84/1	6	**Admitting mean serum glucose level (mg/dL):**<br><br>Before ABF: 448<br>Late ABF: 436<br>*(Outcome summary: decrease late, magnitude ≥ 5%, statistical significance not reported)*

Author (year)	Country (start year ABF)	Study Design	Data Dates (% funding from ABF)*	Primary data source<br><br>Sampling method	Study population<br><br>(N patients / N institutions)	Overall Credibility Score (0-6)	Variable and main findings<br>(outcome direction, magnitude, statistical significance)
Leibson (1990)<br><br>RefID: 6510	USA (1983)	Before/after	No ABF:<br>1980 (0%)<br><br>Early ABF: 1985 (50%)<br><br>Late ABF: 1987 (100%)	All eligible institutions in a jurisdiction	Patients 65 years and older discharged from 3 hospitals in (Olmsted County.<br><br>5854/3	5	**% with high severity (PGRADE>3):**<br><br>Before ABF:17%<br>Early ABF: 24%<br>Late ABF: 23%<br>*(Outcome summary: increase early, magnitude ≥ 5%, statistical significance not reported; increase late, magnitude ≥ 5%; statistical significance not reported)*<br><br>**Mean value for complexity (COMPLEX, a measure of complexity):**<br><br>Before ABF: avg=0.77<br>Early ABF: avg=0.98<br>Late ABF: avg=0.92<br>*(Outcome summary: increase early, magnitude ≥ 5%, p<0.001; increase late, magnitude ≥ 5%, p<0.001)*<br><br>*Overall outcome summary: increase early, increase late, magnitude ≥ 5%, statistical significance mixed*
DesHarnais (1987)<br><br>RefID 4737	USA (1983)	Before/after	No ABF:<br>1980 (0%)<br><br>Early ABF: 1984 (25%)	1. Professional Activity Study of the CPHA.<br><br>2. Computer tape from Annual Survey of Hospitals of the American Hospital Association.<br><br>Convenience sample	All discharges during the third quarter of each year from the hospitals included.<br><br>650596/729	4	**% of cases with at least 1 secondary diagnosis indicating comorbidity and/or complication:**<br><br>Before ABF: 52.93%<br>Early ABF: 60.14%<br>*(Outcome summary: increase early, magnitude ≥ 5%, statistical significance not reported)*<br><br>**Average number of complications and comorbidities:**<br><br>Before ABF: 1.77<br>Early ABF: 1.95<br>*(Outcome summary: increase early, magnitude ≥ 5%, statistical significance not reported)*<br><br>*Overall outcome summary: increase early, magnitude ≥ 5%, statistical significance not reported*
Gerety (1989)<br><br>RefID: 1058	USA (1983)	Before/after	No ABF:<br>1982-1984 (0%)<br><br>Late ABF: 1984-1986 (25-75%)	Patient medical charts.<br><br>Convenience sample	Medicare patients 69 years of age or older with ICD or DRG codes for hip fracture admitted to Stanford University Medical Centre.<br><br>180/1	4	**Horn Severity Index mean (a casemix measure):**<br><br>Before ABF: 1.7<br>Late ABF: 1.8<br>*(Outcome summary: increase late, magnitude ≥ 5%; statistical significance not reported)*
Ray (1990)<br><br>RefID: 14607	USA (1983)	Before/after	No ABF:<br>1981-1983 (0%)<br><br>Late ABF: 1984-1986 (25-75%)	Central Health Care Financing Records for Michigan.<br><br>Random sample	Michigan Medicare patients (65 years and older) admitted with a hip fracture.<br><br>4368/ns	4	**% with at least one hospitalization in past year:**<br><br>Before ABF: 34.1<br>Late ABF: 30.7<br>*(Outcome summary: decrease late, magnitude ≥ 5%, statistical significance not reported)*

Author (year)	Country (start year ABF)	Study Design	Data Dates<br>(% funding from ABF)*	Primary data source<br><br>Sampling method	Study population<br><br>(N patients / N institutions)	Overall Credibility Score (0-6)	Variable and main findings<br>(outcome direction, magnitude, statistical significance)
Rich (1988)<br><br>RefID: 7763	USA (1983)	Before/after	No ABF:<br>1983 (0%)<br><br>Early ABF: 1984 (25%)<br><br>Late ABF: 1986 (75%)	Computerized medical records database at Jewish Hospital, St. Louis, US.<br><br>Convenience sample	Only patients with congestive heart failure (DRG 127) discharged from Jewish Hospital between January 1, 1983 through June 30th, 1986 who are 70 years of age or older with cardiovascular DRG codes, excluding those with prior cardiac admissions, and those who died during initial hospitalization.<br><br>410/1	4	**Number of congestive heart failure patients with secondary diagnoses:**<br><br>Hypertension:<br>Before ABF: 33 (27.5%)<br>Early ABF: 32 (24.4%)<br>Late ABF: 23 (41.1%)<br>*(Outcome summary: decrease early, magnitude ≥ 1% to < 5%, statistical significance not reported; increase late, magnitude ≥ 5%; statistical significance not reported)*<br><br>Diabetes:<br>Before ABF: 24 (20.0%)<br>Early ABF: 24 (18.3%)<br>Late ABF: 11 (19.6%)<br>*(Outcome summary: decrease early, magnitude ≥ 5%, statistical significance not reported; decrease late, magnitude ≥ 1% to < 5%, statistical significance not reported)*<br><br>Cerebrovascular:<br>Before ABF: 4 (3.3%)<br>Early ABF: 6 (4.6%)<br>Late ABF: 1 (1.8%)<br>*(Outcome summary: increase early, magnitude ≥ 5%, statistical significance not reported; decrease late, magnitude ≥ 5%; statistical significance not reported)*<br><br>Peripheral vascular:<br>Before ABF: 4 (3.3%)<br>Early ABF: 5 (3.8%)<br>Late ABF: 6 (10.7%)<br>*(Outcome summary: increase early, magnitude ≥ 5%, statistical significance not reported; increase late, magnitude ≥ 5%; statistical significance not reported)*<br><br>COPD:<br>Before ABF: 9 (7.5%)<br>Early ABF: 17 (13.0%)<br>Late ABF: 8 (14.3%)<br>*(Outcome summary: increase early, magnitude ≥ 5%, statistical significance not reported; increase late, magnitude ≥ 5%; statistical significance not reported)*<br><br>Malignancy:<br>Before ABF: 2 (1.7%)<br>Early ABF: 7 (5.3%)<br>Late ABF: 1 (1.8%)<br>*(Outcome summary: increase early, magnitude ≥ 5%, statistical significance not reported; increase late, magnitude ≥ 5%; statistical significance not reported)*<br><br>*Overall outcome summary: mixed early; mixed late; magnitude ≥ 5%; statistical significance not reported*

Author (year)	Country (start year ABF)	Study Design	Data Dates (% funding from ABF)*	Primary data source<br><br>Sampling method	Study population<br><br>(N patients / N institutions)	Overall Credibility Score (0-6)	Variable and main findings (outcome direction, magnitude, statistical significance)
Carroll (1987)<br><br>RefID 4266	USA (1983)	Before/after	No ABF:<br>1982-1983 (0%)<br><br>Early ABF: 1984-1985 (25-50%)	All data were abstracted from hospital transfer forms, physician or nurse assessment forms and progress notes, state certification for level of care forms, and physician drug order forms.<br><br>Convenience sample	Patients discharged directly from the hospital to the long-term care facility, and his or her hospital stay must have been covered by Medicare.<br><br>353/10	3	**% patients with poor prognosis:**<br><br>Before ABF: 33.9%<br>Early ABF: 34.8%<br>*(Outcome summary: increase early, magnitude ≥ 1% to < 5%, p= 0.75)*
Carroll (1990)<br><br>RefID: 4265	USA (1983)	Before/after	No ABF:<br>1982-1983 (0%)<br><br>Late ABF: 1985-1986 (50-75%)	Data were collected from medical records of seven long-term care facilities patients for August 1982 through July 1983 (pre-PPS) and August 1985 through July 1986 (post-PPS). All data were abstracted from hospital transfer forms, physicians' or nurses' assessment forms and progress notes, state certification for level of care forms, and physicians' drug order forms.<br><br>Convenience sample	Data was collected from the medical records of patients admitted to seven LTCFs in Pennsylvania. To be included in the sample, patients must have been admitted directly from hospitals and had their hospital stays reimbursed by Medicare.<br><br>609/7	3	**Number (%) of patients admitted to LTCFs with selected diagnoses:**<br><br>Before ABF (n=312) vs. Late ABF (n=297):<br>Circulatory system diseases: 173 (55.5%) vs. 186 (62.6%)<br>Fracture, sprain or dislocation: 81 (26.0%) vs. 68 (22.9%)<br>Endocrine, nutritional and metabolic diseases: 36 (11.5%) vs. 74 (24.9%) (p<.01)<br>Musculoskeletal system diseases: 37 (11.9%) vs. 71 (23.9%) (p<.01)<br>Nervous system or sense organ diseases: 35 (11.2%) vs. 67 (22.6%) (p<.01)<br>Mental disorder: 46 (14.7%) vs. 54 (18.2%)<br>Respiratory system diseases: 41 (13.1%) vs. 57 (19.2%) (p<.05)<br>Infection: 32 (10.3%) vs. 64 (21.6%), (p<.01)<br>Neoplasm: 38 (12.2%) vs. 36 (12.1%),<br>Genitourinary diseases: 26 (8.3%) vs. 43 (14.5%) (p<.05)<br>Ill-defined conditions: 18 (5.8%) vs. 40 (13.5%) (p<.01)<br>Digestive system diseases: 26 (8.3%) vs. 28 (9.4%)<br>Skin diseases: 13 (4.2%) vs. 23 (7.7%)<br>Other diagnoses: 20 (6.3%) vs. 25 (8.4%)<br><br>*Overall outcome summary: increase late, magnitude mixed, significance mixed*
Easton (1991)<br><br>RefID 4900	USA (1983)	Before/after	No ABF:<br>1982 (0%)<br><br>Late ABF: 1984-1987 (25-100%)	Patient charts, admitted to large Home Health Agency (HHA) over 5-year period.<br><br>Random sample	Elderly patients admitted to a large Home Health Agency.<br><br>329/1	3	**Changes in activities of daily living (Katz ADL Scale summary scores; lower scores represent independence):**<br><br>Before ABF: 2.44<br>Late ABF: 2.53<br>*(Outcome summary: increase late, magnitude ≥ 1% to < 5%, not statistically significant)*<br><br>**Mental status (subjectively rated by health care providers):**<br><br>Before ABF vs. Late ABF: no difference<br>*(Outcome summary: no difference late, statistical significance not reported)*<br><br>*Overall outcome summary: mixed late, magnitude indeterminate, statistical significance not reported)*
Friedman (1995)<br><br>RefID: 1017	USA (1983)	Before/after	No ABF:<br>1980 (0%)<br><br>Early ABF: 1984<br><br>Late ABF: 1987 (50-100%)	The Hospital Cost and Utilization Project (HCUP).<br><br>Convenience sample	Medicare patients.<br><br>ns/415	3	**Risk-weighted case mix:**<br><br>Before ABF: 9.623<br>Early ABF: 9.066<br>Late ABF: 9.883<br>*(Outcome summary: decrease early, magnitude ≥ 5%, statistical significance not reported; increase late, ≥ 1% to < 5%, statistical significance not reported)*

Author (year)	Country (start year ABF)	Study Design	Data Dates (% funding from ABF)*	Primary data source<br><br>Sampling method	Study population<br><br>(N patients / N institutions)	Overall Credibility Score (0-6)	Variable and main findings (outcome direction, magnitude, statistical significance)
Gay (1990)<br><br>RefID: 1049	USA (1983)	Before/after	No ABF:<br>1981 (0%)<br><br>Early ABF: 1984 (25%)	Medicare discharge abstracts from all reporting South Carolina, short-term, non-federal acute care hospitals.<br><br>All eligible institutions in a jurisdiction	All Medicare patients.<br><br>227771/68	3	**Medicare case mix index:**<br><br>Before ABF vs. Early ABF: increase of 0.07 to 0.12 across age groups, resulting in increased consumption of 7-12% more resources than the national average<br>*(Outcome summary: increase early, magnitude ≥ 5%, statistical significance not reported)*<br><br>**Number of diagnoses:**<br><br>Before ABF vs. Early ABF: increased by 16%<br>*(Outcome summary: increase early, magnitude ≥ 5%; p<0.0001)*<br><br>*Overall outcome summary: increase early, magnitude ≥ 5%; significance mixed*
Gay (1994)<br><br>RefID: 5295	USA (1983)	Before/after	No ABF:<br>1981-1982 (0%)<br><br>Early ABF: 1983-1985 (25-50%)	Fourth quarter Medicare discharge summaries from all reporting South Carolina, short-term, non-federal, acute-care hospitals.<br><br>All eligible institutions in a jurisdiction	Patients from South Carolina acute care hospitals on Medicare.<br><br>ns/68	3	**Medicare Case Mix Index (MCMI) overall:**<br><br>Before ABF: MCMI=1.056<br>Early ABF: MCMI=1.157<br>Late ABF: MCMI=1.159<br>*(Outcome summary: increase early, magnitude ≥ 5%, statistical significance not reported; increase late, magnitude ≥ 5%; statistical significance not reported)*
Kane (1987)<br><br>RefID: 6128	USA (1983)	Before/after	No ABF:<br>1983-1984 (0%)<br><br>Early ABF: 1984-1985 (25-50%)	Medical records of patients admitted to rehabilitation hospital.<br><br>Convenience sample	Not specified.<br><br>516/1	3	**Patient profile scores of functional impairment at admission:**<br><br>Before ABF: 3.15<br>Early ABF: 3.42<br>*(Outcome summary: increase early, magnitude ≥ 5%, p<.01)*<br><br>**Patient profile scores of functional impairment at discharge:**<br><br>Before ABF: 2.19<br>Early ABF: 2.34<br>*(Outcome summary: increase early, magnitude ≥ 5%, statistical significance not reported)*<br><br>*Overall outcome summary: increase early, magnitude ≥ 5%, statistical significance mixed*
Mayer-Oakes (1988)<br><br>RefID: 6863	USA (1983)	Before/after	No ABF:<br>1981-1982 (0%)<br><br>Early ABF: 1984-1985 (25-50%)	MICU log books, medical record.<br><br>Convenience sample	Medicare patients aged 65 or older and comparison patients aged 50-64 admitted to MICUs during the study period. Medicare patients less than 65 years old, primarily those with end stage renal disease were excluded given an oversampling of the group.<br><br>398/3	3	**Severity of illness based on APACHE II score on 1st MICU day:**<br><br>Before ABF: 21.7<br>Early ABF: 19.9<br>*(Outcome summary: decrease early, magnitude ≥ 5%, statistical significance not reported)*

Author (year)	Country (start year ABF)	Study Design	Data Dates (% funding from ABF)*	Primary data source<br><br>Sampling method	Study population<br><br>(N patients / N institutions)	Overall Credibility Score (0-6)	Variable and main findings (outcome direction, magnitude, statistical significance)
Palmer (1989)<br><br>RefID: 7376	USA (1983)	Before/after	No ABF:<br>1981-1984 (0%)<br><br>Late ABF: 1984-1987 (25-100%)	Medical chart review.<br><br>Random sample	Patients over the age of 65 years hospitalized between January 1, 1981 and December 31, 1987, with newly diagnosed hip fractures from a teaching hospital in Indianapolis.<br><br>386/1	3	**Hip fracture patient clinical characteristics:**<br><br>No complications:<br>Before ABF: 119 (60.7%)<br>Late ABF: 133 (67.8%)<br>*(Outcome summary: increase late, magnitude ≥ 5%, statistical significance not reported)*<br><br>Wound infection:<br>Before ABF: 2 (1.0%)<br>Late ABF: 3 (1.5%)<br>*(Outcome summary: increase late, magnitude ≥ 5%, statistical significance not reported)*<br><br>Pulmonary embolism:<br>Before ABF: 1 (0.5%)<br>Late ABF: 3 (1.5%)<br>*(Outcome summary: increase late, magnitude ≥ 5%, statistical significance not reported)*<br><br>Acute MI:<br>Before ABF: 2 (1.0%)<br>Late ABF: 2 (1.0%)<br>*(Outcome summary: no difference late, statistical significance not reported)*<br><br>Urinary tract infection:<br>Before ABF: 34 (17.9%)<br>Late ABF: 15 (7.6%)<br>*(Outcome summary: decrease late, magnitude ≥ 5%, statistical significance not reported)*<br><br>Cerebrovascular accident:<br>Before ABF: 1 (0.5%)<br>Late ABF: 2 (1.0%)<br>*(Outcome summary: increase late, magnitude ≥ 5%, statistical significance not reported)*<br><br>Other complications resulting in surgery:<br>Before ABF: 2 (11.6%)<br>Late ABF: 18 (9.2%)<br>*(Outcome summary: decrease late, magnitude ≥ 5%, statistical significance not reported)*<br><br>Other complications not resulting in surgery:<br>Before ABF: 9 (4.7%)<br>Late ABF: 20 (10.0%)<br>*(Outcome summary: increase late, magnitude ≥ 5%, statistical significance not reported)*<br><br>*Overall outcome summary: mixed late, magnitude ≥ 5%, overall p=.048*

Author (year)	Country (start year ABF)	Study Design	Data Dates (% funding from ABF)*	Primary data source<br><br>Sampling method	Study population<br><br>(N patients / N institutions)	Overall Credibility Score (0-6)	Variable and main findings (outcome direction, magnitude, statistical significance)
Rogers (1990)<br><br>RefID: 2293	USA (1983)	Before/after	No ABF:<br>1981-1982 (0%)<br><br>Late ABF: 1985-1986 (50-75%)	Health Care Financing Administration (HCFA) database.<br><br>Convenience sample	All patients hospitalized in each study year with one of the study diseases.<br><br>17000/300	3	**Percentage of patients in unstable condition at discharge x diagnosis x location of care:**<br><br>Before ABF vs. Late ABF<br>**CHF:**<br>rural non-teaching: +4.3%<br>urban non-teaching: +3.5%<br>urban teaching: +2.8%<br><br>**Acute MI:**<br>rural non-teaching: +1.7%<br>urban non-teaching: +1.1%<br>urban teaching: +4.7%<br><br>**Pneumonia:**<br>rural non-teaching: +11.6%<br>urban non-teaching: -0.3%<br>urban teaching: +2.5%<br><br>**CVA:**<br>rural non-teaching: +6.3%<br>urban non-teaching: +3.7%<br>urban teaching: +2.3%<br><br>**Hip Fracture:**<br>rural non-teaching: +11.31%<br>urban non-teaching: +3.1%<br>urban teaching: +2.9%<br><br>*(Outcome summary: increase late, magnitude mixed, statistical significance not reported)*
Sloan (1988)<br><br>RefID: 2487	USA (1983)	Before/after	No ABF:<br>1980 (0%)<br><br>Early ABF: 1983 (25%)<br><br>Late ABF: 1985 (50%)	Discharge abstracts submitted to the Commission on Professional and Hospital Activities (CPHA) on a sample of 467 hospitals.<br><br>Convenience sample	Not specified.<br><br>ns/467	3	**Medicare Case Mix Index (MCI); Resource Need Index (RNI); % Very Complex Cases (VC=MCI>3.0):**<br><br>Before ABF: MCI=1.12; RNI=1.56; %VC=1.0<br>Early ABF: MCI=1.14; RNI=1.58; %VC=1.3<br>Late ABF: MCI=1.22; RNI=1.72; %VC=1.6<br>*(Outcome summary: increase early, magnitude mixed, statistical significance not reported; increase late, magnitude ≥ 5%; statistical significance not reported)*
**International**							

Author (year)	Country (start year ABF)	Study Design	Data Dates (% funding from ABF)*	Primary data source<br><br>Sampling method	Study population<br><br>(N patients / N institutions)	Overall Credibility Score (0-6)	Variable and main findings (outcome direction, magnitude, statistical significance)
Kerr (1998)<br><br>RefID: 1510	Australia (1993)	Before/after	No ABF:<br>1992 (0%)<br><br>Early ABF: 1993 (100%)	Randomized prospective study.<br><br>Convenience sample	All patients admitted to the coronary care unit with uncomplicated, suspected unstable angina.<br><br>336/1	6	**Coronary Risk Factors:**<br><br>Before ABF: 79%<br>Early ABF: 79%<br>*(Outcome summary: no difference early, statistical significance not reported)*<br><br>**Diabetes Mellitus:**<br><br>Before ABF: 28%<br>Early ABF: 33%<br>*(Outcome summary: increase early, magnitude ≥ 5%; statistical significance not reported)*<br><br>**History of ischemic heart disease:**<br><br>Before ABF: 53%<br>Early ABF: 58%<br>(increase, statistical significance not reported)<br>*(Outcome summary: increase early, magnitude ≥ 5%; statistical significance not reported)*<br><br>*Overall outcome summary: increase early, magnitude ≥ 5%; statistical significance not reported*

Author (year)	Country (start year ABF)	Study Design	Data Dates (% funding from ABF)*	Primary data source<br><br>Sampling method	Study population<br><br>(N patients / N institutions)	Overall Credibility Score (0-6)	Variable and main findings (outcome direction, magnitude, statistical significance)
Schuetz (2011)<br><br>RefID: 8140	Switzerland (2001)	Parallel groups	No ABF:<br>2006-2008 (0%)<br><br>Late ABF: 2006-2008 (100%)	This is a post-hoc analysis from a previous randomised-controlled trial.<br><br>Convenience sample	a cohort of patients with community acquired pneumonia, >18 years old.<br><br>925/6	6	**Pneumonia Severity Index:**<br><br>No ABF: 93%<br>Late ABF: 90 %<br>*(Outcome summary: decrease late, magnitude ≥ 5%, p=0.25)*<br><br>**Co-existing chronic renal failure:**<br><br>No ABF: 20%<br>Late ABF: 27%<br>*(Outcome summary: increase late, magnitude ≥ 5%, p=0.02)*<br><br>**Co-existing congestive heart failure:**<br><br>No ABF: 19%<br>Late ABF: 11%<br>*(Outcome summary: decrease late, magnitude ≥ 5%, p<0.01)*<br><br>**Co-existing COPD:**<br><br>No ABF: 32%<br>Late ABF: 27%<br>*(Outcome summary: decrease late, magnitude ≥ 5%, p=0.21, no significant difference)*<br><br>**Diabetes:**<br><br>No ABF: 17%<br>Late ABF: 20%<br>*(Outcome summary: increase late, magnitude ≥ 5%, p=0.20, no significant difference)*<br><br>*Overall outcome summary: mixed late, magnitude ≥ 5%, statistical significance mixed*
Louis (1999)<br><br>RefID: 1719	Italy (1995)	Before/after	No ABF:<br>1993 (0%)<br><br>Early ABF: 1996 (ns)	Hospital discharge abstract data from 1993 through 1996 for all hospitals (N = 32) in the Friuli-Venezia-Giulia region of Italy. Regional population data were used to calculate rates.<br><br>All eligible institutions in a jurisdiction	Not specified.<br><br>ns/32	4	**Severity of patient illness (% stage 2-3 admissions):**<br><br>Before ABF vs. Early ABF: increased significantly (p=.01) for all medical and surgical conditions studied (i.e. appendicitis, diabetes mellitus, colorectal cancer, cholecystitis, COPD, bacterial pneumonia, coronary artery disease, cerebrovascular disease), except hip fracture, where no change was observed (p=0.80)<br>*(Outcome summary: increase early, magnitude indeterminate, p=.01)*

Author (year)	Country (start year ABF)	Study Design	Data Dates (% funding from ABF)*	Primary data source<br><br>Sampling method	Study population<br><br>(N patients / N institutions)	Overall Credibility Score (0-6)	Variable and main findings (outcome direction, magnitude, statistical significance)
von Eiff (2011)<br><br>RefID: 8845	Germany (2004)	Before/after	No ABF:<br>2003-2004 (0%)<br><br>Late ABF: 2008-2009 (100%)	Prospective, multi-centered, national-wide study assessing the impact of ABF.<br><br>Convenience sample	Patients admitted in rehabilitation after cardiac (myocardial infarction, coronary bypass) or orthopedic procedure (hip or knee surgery).<br><br>1562/24	4	**Proportion of patients admitted to PAC with Hemoglobin level less than 10g/dl.:**<br><br>Before ABF vs. Late ABF: Relative increase of more than 3 times<br>*(Outcome summary: increase late, magnitude ≥ 1% to < 5%, statistical significance not reported)*<br><br>**C-reactive protein blood levels at admission in PAC:**<br><br>Before ABF vs. Late ABF: Relative increase ranging from 1.5 to 1.7 among hip/knee surgery patients<br>*(Outcome summary: increase late, magnitude ≥ 1% to < 5%, statistical significance not reported)*<br><br>*Overall outcome summary: increase late, magnitude ≥ 1% to < 5%, statistical significance not reported*
Jauss (2010)<br><br>RefID: 1421	Germany (2004)	Before/after	No ABF:<br>2003-2004 (0%)<br><br>Early ABF: 2004 (ns)<br><br>Late ABF: 2006 (ns)	Administrative database of the German state of Hessen including all patients with stroke.<br><br>All eligible institutions in a jurisdiction	All patients diagnosed with ischemic stroke (excluding transitory ischemic stroke, hemorrhagic stroke).<br><br>27005/ns	3	**Severity of stroke (proportion of Rankin score >=3) at admission:**<br><br>Before ABF: 66%<br>Early ABF: 64%<br>Late ABF: 61%<br>*(Outcome summary: decrease early, magnitude ≥ 1% to < 5%, p<0.001; decrease late, magnitude ≥ 5%; p<0.001)*<br><br>**Proportion of patients with neurological deficits at admission:**<br><br>Before ABF vs. Early ABF:<br>Decrease in % paresis (absolute -6%, relative reduction of 8%).<br>Decrease in % level of consciousness (absolute -3%, relative reduction of 15%).<br>Decrease in % dysarthria (absolute -6%, relative reduction of 18%).<br>Increase in % aphasia (absolute +2%, relative increase of 6%).<br>Increase in % dysphagia (absolute +2%, relative increase of 11%).<br>*(Outcome summary: decrease early, magnitude ≥ 5%; p< .001)*<br><br>*Overall outcome summary: decrease early, magnitude mixed, p<0.001; decrease late, magnitude ≥ 5%; p<0.001)*
Onder (2001)<br><br>RefID: 7313	Italy (1994)	Before/after	No ABF:<br>1988-1993 (0%)<br><br>Late ABF: 1995-1998 (100%)	Gruppo Italiano di Farmacoepidemiologia nell'Anziano (GIFA) database.<br><br>Convenience sample	All patients 65 years or older with pneumonia admitted to 40 medical centres distributed throughout Italy.<br><br>963/40	3	**Clinical complexity (Charlson Comorbidity Index) [mean +/- SD]:**<br><br>Before ABF: 1.38 +/- 1.70<br>Early ABF: 1.70 +/- 1.72<br>*(Outcome summary: increase early, magnitude ≥ 5%, p=0.005)*<br>**Diagnosis of Pneumonia:**<br><br>Before ABF: 4.2%<br>Early ABF: 4.1%<br>*(Outcome summary: decrease early, magnitude ≥ 1% to < 5%, statistical significance not reported)*<br><br>*Overall outcome summary: mixed early, magnitude mixed, statistical significance mixed*

Author (year)	Country (start year ABF)	Study Design	Data Dates (% funding from ABF)*	Primary data source<br><br>Sampling method	Study population<br><br>(N patients / N institutions)	Overall Credibility Score (0-6)	Variable and main findings (outcome direction, magnitude, statistical significance)
Researchers of the Italian Group of Pharmaco-epidemiology in the Aged (GIFA) (1996)<br><br>RefID: 3228	Italy (1994)	Before/after	No ABF:<br>1993 (0%)<br><br>Early ABF: 1995 (ns)	GIFA (Group of Pharmaco-epidemiology in the Aged).<br><br>All eligible institutions in a jurisdiction	Not specified.<br><br>ns/83	3	**Proportion of warranted admissions:**<br><br>Before ABF:85.5%<br>Early ABF: 87.2%<br>*(Outcome summary: increase early, magnitude ≥ 5%; p< 0.0001)*
Xiao (2000)<br><br>RefID: 2859	Australia (1996)	Before/after	No ABF:<br>1991-1996 (0%)<br><br>Early ABF: 1996-1997 (100%)	Northern Territory aggregated Hospital Morbidity Databases, Australia.<br><br>Convenience sample	Patients admitted to five Northern Territory public hospitals, excluding renal dialysis patients.<br><br>ns/5	3	**Case Mix Index:**<br><br>Before ABF vs. Early ABF: No apparent change<br>*(Outcome summary: no difference early, statistical significance not reported)*

*****US Medicare ABF Phase-in Schedule:
Beginning on or after October 1, 1983 and before October 1, 1984: 25% ABF; Beginning on or after October 1, 1984 and before October 1, 1985: 50% ABF; Beginning on or after October 1, 1985 and before October 1, 1986: 75% ABF; Beginning on or after October 1, 1986: 100% ABF; If the percentage of ABF changed during the data reporting period, we used the percent ABF present for the majority of the period.
**ns=not specified
Note: Studies stratified by US and International and ordered by credibility (highest to lowest) and then alphabetical by first author's last name.

Appendix 27: Severity of Illness Analysis Table (Non-Pooled)

Significance of Effect	Magnitude ≥5%*			Magn ≥1% to <5%		Magn <1%		Magn Indeterminate or mixed			
	Direction										
	Inc	Dec	Mixed	Inc	Dec	Inc	Dec	Inc	Dec	No diff	Mixed
**Early ABF**											
p >.05				4266						5100	
p ≤ .05-.02											
p ≤ .01-.002								1719✓			
p ≤ .001	3228✓								1421 ✓		
**Stat sig not reported	4737<br>5295	1017<br>6863	5806<br>7763					2487		2859✓	
Stat sig mixed	1049<br>1510✓<br>6128<br>6510									1664	7313✓
**Late ABF**											
p >.05											
p ≤ .05-.02			7376								
p ≤ .01-.002											
p ≤ .001		1421✓									
**Stat sig not reported	1058<br>1654<br>2487<br>5295	8981<br>14607	7763	8845✓<br>1017				2293		5101	4900
Stag sig mixed	6510		8140✓					4265			

* Formula for relative percent difference = (T2-T1/T1)(100)
**Abstracter judged increase, decrease, or no diff, but authors did not report statistical significance OR authors state results were significant or not significant, but did not report test
✓International study
Notes:
If both early and late data reported, we counted each period once (study gets two counts, one for early, one for late)
If p-value > 0.05 we counted as no difference irrespective of magnitude of effect

Appendix 28: Severity of Illness Vote Count Table (Non-Pooled)

	Early ABF			Late ABF		
	Increase	Decrease	No diff/mixed	Increase	Decrease	No diff/mixed
Magnitude ≥ 5% and p ≤ .01	3228✓					
Magnitude ≥ 1% to < 5% and p ≤ .01					1421✓	
Magnitude <1% and p ≤ .01;<br>or<br>Mag.≥ 5% and stat sig. not reported or mixed	4737<br>5295<br>1049<br>1510✓<br>6128<br>6510	1017<br>6863	5806	1058<br>1654<br>2487<br>5295<br>6510	8981<br>14607	8140✓
*Other results	1719✓<br>2487<br>4266	1421✓	7763<br>5100<br>2859✓<br>7313✓<br>1664	8845✓<br>1017<br>2293<br>4265		7376<br>7763<br>5101<br>4900

*Other results" refers to studies reporting combinations of magnitude and p-value other than the classifications in this table
✓International study

Appendix 29: Volume of Care Study Descriptions and Main Findings Table (Non-Pooled)

Author (year)	Country (start year ABF)	Study Design	Data Dates (% funding from ABF)*	Primary data source<br><br>Sampling method	Study population<br><br>(N patients / N institutions)	Overall Credibility Score (0-6)	Variable and main findings (outcome direction, magnitude, statistical significance)
**US**							
Leibson (1991)<br><br>RefID: 1654	USA (1983)	Before/after	No ABF:<br>1980 (0%)<br><br>Late ABF: 1987 (100%)	Medical records in Olmsted County, Minnesota, Mayo Clinics and affiliated hospitals.<br><br>All eligible institutions in a jurisdiction	All patients with 65 years of age or older.<br><br>3710/5	6	Hospital days of care:<br><br>Before ABF vs. Late ABF: decreased by 9.8%<br>*(Outcome summary: decrease late, magnitude ≥ 5%, statistical significance not reported)*<br><br>Number of persons hospitalized:<br><br>Before vs. Late ABF: increased of 1.5%<br>*(Outcome summary: increase late, magnitude ≥ 1% to < 5% statistical significance not reported)*<br><br>*Overall outcome summary: mixed late, magnitude mixed, statistical significance not reported*
Fitzgerald (1988)<br><br>RefID: 5101	USA (1983)	Before/after	No ABF:<br>1981-1983 (0%)<br><br>Late ABF: 1984-1986 (25-75%)	Hospital records in a community hospital in a large Midwestern city<br><br>Telephone interviews.<br><br>All eligible institutions in a jurisdiction	All patients >65 years old admitted to hospital with a new hip fracture between October 1, 1981 and March 1, 1986.<br><br>338/1	4	Number of patients with hip fracture:<br><br>Before ABF: 149<br>Late ABF: 189<br>*(Outcome summary: increase late, magnitude ≥ 5%, statistical significance not reported)*
Eggers (1987)<br><br>RefID: 4917	USA (1983)	Before/after	No ABF:<br>1980 (0%)<br><br>Early ABF: 1983-1984 (ns)	Health Care Financing Administration (HCFA) database.<br><br>All eligible institutions in a jurisdiction	All Medicare patients.<br><br>ns/ns	3	% change discharge rate per 1000:<br><br>Before ABF: 2.0%<br>Early ABF: 3.5%<br>*(Outcome summary: increase early, magnitude ≥ 5%, statistical significance not reported)*
Leibson (1990)<br><br>RefID: 6510	USA (1983)	Before/after	No ABF:<br>1980 (0%)<br><br>Early ABF: 1985 (50%)<br><br>Late ABF: 1987 (100%)	Medical record of two Mayo affiliated hospital and Olmsted community hospital.<br><br>All eligible institutions in a jurisdiction	Patients 65 years and older discharged from 3 hospitals in Olmsted County.<br><br>5854/3	3	Total number of admissions for patients 65 years and older:<br><br>Before ABF: 1783 admits of 8574 at risk (21%)<br>Early ABF 1978/9516 (21%)<br>Late ABF: 2093/9728 (21%)<br>*(Outcome summary: No difference early, no difference late, statistical significance not reported)*
DesHarnais (1987)<br><br>RefID: 4737	USA (1983)	Before/after	No ABF:<br>1980 (0%)<br><br>Early ABF: 1984 (25%)	The Professional Activity Study of the CPHA<br>The computer tape from the Annual Survey of Hospitals of the American Hospital Association<br><br>Convenience sample	All discharges during the third quarter of each year from the hospitals included.<br><br>650596/729	2	Number of discharges:<br><br>Before ABF: 36,651<br>Late ABF: 34,663<br>*(Outcome summary: decrease late, magnitude ≥ 5%, p<.05)*

Author (year)	Country (start year ABF)	Study Design	Data Dates (% funding from ABF)*	Primary data source<br><br>Sampling method	Study population<br><br>(N patients / N institutions)	Overall Credibility Score (0-6)	Variable and main findings (outcome direction, magnitude, statistical significance)
Gerety (1989)<br><br>RefID: 1058	USA (1983)	Before/after	No ABF:<br>1982-Sept 1,1984 (0%)<br><br>Late ABF: Sept. 2, 1984-1986 (25-75%)	Patient medical charts.<br><br>Convenience sample	Patients 69 years of age or older with ICD or DRG codes for hip fracture admitted to Stanford University Medical Centre.<br><br>180/1	2	Number of Admissions:<br><br>Before ABF: 64<br>Late ABF: 111<br>*(Outcome summary: increase late, magnitude ≥ 5%, statistical significance not reported)*
Carroll (1990)<br><br>RefID: 4265	USA (1983)	Before/after	No ABF:<br>1982-1983 (0%)<br><br>Late ABF: 1985-1986 (50-75%)	Data were collected from medical records of seven long-term care facilities patients for August 1982 through July 1983 (pre-PPS) and August 1985 through July 1986 (post-PPS). All data were abstracted from hospital transfer forms, physicians' or nurses' assessment forms and progress notes, state certification for level of care forms, and physicians' drug order forms.<br><br>Convenience sample	Data was collected from the medical records of patients admitted to seven LTCFs in Pennsylvania. To be included in the sample, patients must have been admitted directly from hospitals and had their hospital stays reimbursed by Medicare.<br><br>609/7	1	Number of patients admitted to long-term care facilities with selected diagnoses:<br><br>Before ABF: n=312<br>Late ABF: n=297<br>*(Outcome summary: decrease early, magnitude ≥ 1% to < 5%, statistical significance not reported)*
DesHarnais (1990)<br><br>RefID: 4740	USA (1983)	Before/after	No ABF:<br>1980-1983 (0%)<br><br>Early ABF: 1984 (25%)<br><br>Late ABF: 1987 (100%)	The Professional Activity Study (PAS) database of the Commission on Professional and Hospital Activities (CPHA).<br><br>Convenience sample	All patients with a psychiatric disease admitted to the qualifying hospitals.<br><br>ns/386	1	Year-over-year % change in number of hospital admissions for psychiatric patients treated in short-term general hospitals:<br><br>Before ABF: 6.3%<br><br>Early ABF: -8.2%<br>Late ABF: .2%<br>*(Outcome summary: decrease early, magnitude ≥ 5%, statistical significance not reported; decrease late, magnitude ≥ 5%, statistical significance not reported)*
Easton (1991)<br><br>RefID: 4900	USA (1983)	Before/after	No ABF:<br>1982 (0%)<br><br>Late ABF: 1984-1987 (25-100%)	Patient charts, admitted to large Home Health Agency (HHA) over 5-year period.<br><br>Random sample	Elderly patients admitted to a large Home Health Agency.<br><br>329/1	1	Mean number of services received by patients in the first 2 weeks in the HHA:<br><br>Before ABF: 2.17<br>Late ABF: 2.45<br>*(Outcome summary: increase late, magnitude ≥ 5%, p<0.05)*

Author (year)	Country (start year ABF)	Study Design	Data Dates (% funding from ABF)*	Primary data source<br><br>Sampling method	Study population<br><br>(N patients / N institutions)	Overall Credibility Score (0-6)	Variable and main findings (outcome direction, magnitude, statistical significance)
Gay (1990)<br><br>RefID: 1049	USA (1983)	Before/after	No ABF:<br>1981 (0%)<br><br>Early ABF: 1984 (25%)	Medicare discharge abstracts from all reporting South Carolina, short-term, non-federal acute care hospitals.<br><br>All eligible institutions in a jurisdiction	All Medicare patients.<br><br>227771/68	1	Number of admissions:<br><br>Before ABF: 110,117<br>Early ABF: 117,654<br>*(Outcome summary: increase early, magnitude ≥ 5%, p<0.001)*<br><br>Proportion of admissions per age group:<br><br>Before ABF: 41.6% (65-70 years); 49.2% (70-84 years); 9.2% (>85 years)<br>Early ABF: 36.8% (65-70 years); 53.3% (70-84); 9.9% (>85 years)<br>*(Outcome summary: mixed early, magnitude ≥ 5%, all p<0.001*<br><br>Average number of procedures per age group:<br><br>Before vs. Early ABF: increased overall by 1.6% (slightly increased for younger patients and decreased for older patients)<br>*(Outcome summary: increase early, magnitude ≥* 1% to < 5%, *statistical significance not reported)*<br><br>*Overall Outcome summary: mixed early, magnitude mixed, statistical significance mixed*
Kane (1987)<br><br>RefID: 6128	USA (1983)	Before/after	No ABF:<br>1983 (0%)<br><br>Early ABF: 1984-1985 (25-50%)	medical records of patients admitted to rehabilitation hospital.<br><br>Convenience sample	Not specified.<br><br>516/1	1	Total number of patients completing the rehab program:<br><br>Before ABF: 215<br>Late ABF: 240<br>*(Outcome summary: increase late, magnitude ≥ 5%, statistical significance not reported)*
Manton (1990)<br><br>RefID: 6774	USA (1983)	Before/after	No ABF:<br>1982-1983 (0%)<br><br>Early ABF: 1984-1985 (25-50%)	1982 and 1984 National long-term care surveys (Health care financing Administration, HCFA) of disabled elderly Medicare beneficiaries and their Medicare part A bills and Medicare records on mortality.<br><br>Random sample	Nationally representative samples (~6000 people in each) of disabled Medicare beneficiaries that are not institutionalized.<br><br>6000/ns	1	Total weighted episodes of hospital discharges:<br><br>Before ABF vs. Early ABF: no significant change after adjustment for case mix<br>*(Outcome summary: no difference early, p= 0.55)*
Manton (1993)<br><br>RefID: 1775	USA (1983)	Before/after	No ABF:<br>1982 (0%)<br><br>Early ABF: 1984 (25%)	National Long Term Care Surveys (NLTCS) linked to Medicare Part A administrative records.<br><br>Random sample	All Medicare patients<br><br>ns/ns	1	Number of hospital stays (life table estimates standardized for case):<br><br>Before ABF: 9,924,159<br>Early ABF: 8,848,323<br>*(Outcome summary: decrease early, magnitude ≥ 5%, statistical significance not reported)*
Palmer (1989)<br><br>RefID: 7376	USA (1983)	Before/after	No ABF:<br>1981-1984 (0%)<br><br>Late ABF: 1984-1987 (25-100%)	Medical chart review/<br><br>Random sample	Patients over the age of 65 years hospitalized between January 1, 1981 and December 31, 1987, with newly diagnosed hip fractures from a teaching hospital in Indianapolis.<br><br>386/1	1	Number of orthopedic hip fracture cases:<br><br>Before ABF : 47.9%<br>Early ABF: 33.0%<br>Late ABF: 32.5%<br>*(Outcome summary: decrease early, magnitude ≥ 5%, statistical significance not reported; decrease late, magnitude ≥ 5%, statistical significance not reported)*

Author (year)	Country (start year ABF)	Study Design	Data Dates (% funding from ABF)*	Primary data source<br><br>Sampling method	Study population<br><br>(N patients / N institutions)	Overall Credibility Score (0-6)	Variable and main findings (outcome direction, magnitude, statistical significance)
DesHarnais (1988)<br><br>RefID: 4735	USA (1983)	Before/after	No ABF:<br>1980-1983 (0%)<br><br>Early ABF: 1983-1984 (25%)<br><br>Late ABF: 1984-1985 (25-50%)	Primary source: The Professional Activity Study (PAS) of the Commission on Professional and Hospital Activities (CPHA)<br><br>American Hospital Association's Annual Survey of Hospitals<br><br>Convenience sample	Medicare patients: Patients 65 years and older where Medicare is designated as the principal source of payment on the hospital abstract<br><br>Non-Medicare patients: Patients under 65 where payment sources other than Medicare are given on the hospital abstract<br><br>ns/646	0	Total Medicare discharges (thousands):<br><br>Before ABF: 28471<br>Early ABF: 30974<br>Late ABF: 29289<br>*(Outcome summary: increase early, magnitude ≥ 5%, statistical significance not reported; increase late, magnitude ≥ 1% to < 5%, statistical significance not reported)*
Helms (1987)<br><br>RefID: 5724	USA (1983)	Before/after	No ABF:<br>1980 (0%)<br><br>Early ABF: 1984 (25%)<br><br>Late ABF: 1986 (75%)	1980-83: Medical records. 1984-86: Blue Cross billing records.<br><br>All eligible institutions in a jurisdiction	Patients >18 in Iowa admitted with septicemia.<br><br>4888/7	0	Number of discharges coded as Septicemia/total discharges for all diagnoses:<br><br>Before ABF: 295/172,000 (17.2/10,000)<br>Early ABF: 932/154,000 (60.6/10,000)<br>Late ABF: 1,143/140,000 (82.0/10,000)<br>*(Outcome summary: increase early, magnitude ≥ 5%, statistical significance not reported; increase late, magnitude ≥ 5%, statistical significance not reported)*<br><br>Case fatality rate (%):<br><br>Before ABF: case fatality rate 25.1<br>Early ABF: case fatality rate 10.8<br>Late ABF: case fatality rate 18.9<br>*(Outcome summary: decrease early, decrease late, magnitude ≥ 5%, statistical significance not reported)*<br><br>*Overall Outcome Summary: mixed early, mixed late, magnitude ≥ 5%, statistical significance not reported)*<br><br>*NB. Dramatic increase in volume of septicemia diagnoses with ABF, coupled with dramatic decrease in case fatality rate, highly suggestive of upcoding. Not included in vote count.

Author (year)	Country (start year ABF)	Study Design	Data Dates (% funding from ABF)*	Primary data source<br><br>Sampling method	Study population<br><br>(N patients / N institutions)	Overall Credibility Score (0-6)	Variable and main findings (outcome direction, magnitude, statistical significance)
Long (1987)<br><br>RefID: 1711	USA (1983)	Before/after	No ABF:<br>1980-1983 (0%)<br><br>Early ABF: 1984 (25%)	The third-quarter cohort in the Professional Activity Study of the Commission on Professional and Hospital Activities (CPHA).<br><br>Convenience sample	Medicare patients.<br><br>ns/729	0	Mean number of laboratory test per patient:<br><br>Before ABF: 15.6<br>Early ABF: 10.3<br>*(Outcome summary: decrease early, magnitude ≥ 5%, statistical significance not reported)*<br><br>Mean number of diagnostic test per patient:<br><br>Before ABF: 1.154<br>Early ABF: 1.003<br>*(Outcome summary: decrease early, magnitude ≥ 5%, statistical significance not reported)*<br><br>Mean number of X-rays per patient:<br><br>Before ABF : 0.98<br>Early ABF: 0.84<br>*(Outcome summary: decrease early, magnitude ≥ 5%, statistical significance not reported)*<br><br>*Overall Outcome Summary: decrease early, magnitude ≥ 5%, statistical significance not reported*
**International**							
Strömberg (1997)<br><br>RefID: 8497	Sweden (1992)	Before/after	No ABF:<br>1990 (0%)<br><br>Early ABF: 1992 (ns)	Computerized diagnosis-based inpatient system.<br><br>Convenience sample	65 years or older at the time of the injury, residents of the City of Stockholm, and admitted from an independent living situation, and initially treated in orthopedic departments in 4 Stockholm hospitals.<br><br>2238/4	4	Number of patients admitted to acute hospitals:<br><br>Before ABF:1060<br>Early ABF: 1178<br>*(Outcome summary: increase early, magnitude ≥ 5%, statistical significance not reported)*
Farrar (2009)<br><br>RefID: 924	England and Scotland (2004 in England)	Before/after + Parallel Groups	No ABF:<br>2003-2004 (0%)<br><br>Early ABF: 2003-2005 (100%)<br><br>Late ABF: 2003-2006 (100%)	Hospital episode statistics (England, ABF-adopter).<br><br>Scottish morbidity records (Scotland, ABF non-adopter).<br><br>All eligible institutions in each jurisdiction	All patients admitted to acute care hospitals in England and Scotland<br><br>ns/297	3	% difference-in-difference volume of care between England and Scotland:<br><br>No ABF vs. Early ABF: 1.33% greater increase<br>No ABF vs. Late ABF: 4.95% greater increase<br>*(Outcome summary: greater increase early and late, magnitude indeterminate, p<0.01)*<br><br>Day cases as a proportion of elective admissions:<br><br>No ABF vs. Early ABF: 0.4% greater increase<br>No ABF vs. Late ABF: 1.5% greater increase<br>*(Outcome summary: greater increase in early and late, magnitude indeterminate, p<0.01)*<br><br>*(Outcome summary: greater increase in early and late, magnitude indeterminate, p<0.01)*

Author (year)	Country (start year ABF)	Study Design	Data Dates (% funding from ABF)*	Primary data source<br><br>Sampling method	Study population<br><br>(N patients / N institutions)	Overall Credibility Score (0-6)	Variable and main findings (outcome direction, magnitude, statistical significance)
Louis (1999)<br><br>RefID: 1719	Italy (1995)	Before/after	No ABF:<br>1993 (0%)<br><br>Early ABF: 1996 (ns)	Hospital discharge abstract data from 1993 through 1996 for all hospitals (N = 32) in the Friuli-Venezia-Giulia region of Italy. Regional population data were used to calculate rates.<br><br>All eligible institutions in a jurisdiction	Not specified.<br><br>ns/32	3	Total number of ordinary (inpatient) hospital admissions:<br><br>Before ABF: 244,581<br>Early ABF: 204,054<br>*(Outcome summary: decrease early, magnitude ≥ 5%, p<.001)*
South (1997)<br><br>RefID: 2524	Australia (1993)	Before/after	No ABF:<br>1989-1993 (0%)<br><br>Late ABF: 1993-1996 (ns)	Prospective data collection.<br><br>Convenience sample	All children discharged from the Royal Children's Hospital general medical and thoracic units with a primary diagnosis of asthma.<br><br>11939/1	3	Mean number of children admitted with asthma:<br><br>Before ABF: 1607<br>Early ABF: 1804<br>Late ABF: 1520<br>*(Outcome summary: increase early, magnitude ≥ 5%, statistical significance not reported; decrease late, magnitude ≥ 5%, statistical significance not reported)*
Xiao (2000)<br><br>RefID: 2859	Australia (1996)	Before/after	No ABF:<br>1991-1996 (0%)<br><br>Early ABF: 1996-1997 (100%)	Northern Territory aggregated Hospital Morbidity Databases, Australia.<br><br>Convenience sample	Patients admitted to five Northern Territory public hospitals, excluding renal dialysis patients<br><br>ns/5	3	Weighted separations evident for teaching and non-teaching hospitals (sum of numbers of separations multiplied by the cost weights):<br><br>Before ABF:150 (teaching), 550 (non-teaching)<br>Early ABF: 300 (teaching), 750 (non-teaching<br>*(Outcome summary: increase early, magnitude ≥ 5%, statistical significance not reported)*<br><br>Number of bed-days (product of average LOS and number of separations):<br><br>Before ABF vs. Early ABF: decline in bed-days, though still a general upward trend for teaching hospitals<br>*(Outcome summary: decrease early, magnitude indeterminate, statistical significance not reported)*<br><br>*Overall Outcome Summary: mixed early, magnitude indeterminate, statistical significance not reported*
Brizioli (1996)<br><br>RefID: 477	Italy (1995)	Before/After	No ABF:<br>1994 (0%)<br><br>Early ABF: 1995 (100%)	Italian Health Ministry Official Reports Regione Marche Hospital Activity Annual Report hospital specific data.<br><br>Convenience sample	Elderly patients included in DRG127 (heart failure and shock).<br><br>1987/4	2	Total discharges:<br><br>Before ABF vs. Early ABF: increased by 10.34%<br>*(Outcome summary: increase early, magnitude ≥ 5%, statistical significance not reported)*
Frick (2001)<br><br>RefID: 1015	Austria (1997)	Before/after	No ABF:<br>1991-1996 (0%)<br><br>Early ABF: 1997-1998 (100%)	Complete hospital discharge statistics of the Salzburg province and residents from Salzburg hospitalized outside the province (compulsory by law).<br><br>All eligible institutions in a jurisdiction	All inpatients in psychiatry (regardless of specific diagnosis).<br><br>37215/ns	2	Hospitalization rates:<br><br>Before ABF vs. Early ABF: No impact on hospitalization rates<br>*(Outcome summary: no difference early, statistical significance not reported)*

Author (year)	Country (start year ABF)	Study Design	Data Dates (% funding from ABF)*	Primary data source<br><br>Sampling method	Study population<br><br>(N patients / N institutions)	Overall Credibility Score (0-6)	Variable and main findings (outcome direction, magnitude, statistical significance)
Busato (2010)<br><br>RefID: 4207	Switzerland (2003)	Parallel groups	No ABF:<br>2003-2007 (0%)<br><br>Early ABF: 2003-2007	Complete dataset of all hospital discharges in Switzerland 2003-2007 (Swiss Federal Statistical Office;) complete claims data at the expense of basic health insurance of physicians in own practice for the same period.<br><br>All eligible institutions in a jurisdiction	All hospitalized patients except for those in psychiatry and rehabilitation institutions.<br><br>ns/ns	1	Hospitalization rate/1000 inhabitants:<br><br>No ABF: 147.57<br>Early ABF: 143.51<br>*(Outcome summary: decrease early, magnitude ≥ 1% to < 5%, statistical significance not reported)*
Researchers of the Italian Group of Pharmaco-epidemiology in the Aged (GIFA) (1996)<br><br>RefID: 3228	Italy (1994)	Before/after	No ABF:<br>1993 (0%)<br><br>Early ABF: 1995 (ns)	GIFA (Group of Pharmaco-epidemiology in the Aged).<br><br>All eligible institutions in a jurisdiction	Not specified.<br><br>ns/83	1	Number of hospital admissions:<br><br>Before ABF: 5662<br>Early ABF: 1818<br>*(Outcome summary: decrease early, magnitude ≥ 5%, statistical significance not reported)*

***US Medicare ABF Phase-in Schedule:**
Beginning on or after October 1, 1983 and before October 1, 1984: 25% ABF; Beginning on or after October 1, 1984 and before October 1, 1985: 50% ABF; Beginning on or after October 1, 1985 and before October 1, 1986: 75% ABF; Beginning on or after October 1, 1986: 100% ABF; If the percentage of ABF changed during the data reporting period, we used the percent ABF present for the majority of the period.
**ns=not specified
Note: Studies stratified by US and International and ordered by credibility (highest to lowest) and then alphabetical by first author's last name.
.

Appendix 30: Volume of Care Analysis Table (Non-Pooled)

Significance of Effect	Magnitude ≥5%**		Magn ≥1% to <5%		Magn <1%		Magn Indeterminate or mixed			
	Direction									
	Inc	Dec	Inc	Dec	Inc	Dec	Inc	Dec	No diff	Mixed
**Early ABF**										
p >.05									6774	
p ≤ .05-.02										
p ≤ .01-.002							924 ✓			
p ≤ .001		1719✓								
**Stat sig not reported	477✓<br>4735<br>2524✓<br>4917<br>8497✓	1711<br>1775<br>3228✓<br>4740<br>7376		4207✓<br>4265					6510<br>1015✓	2859✓
Stat sig mixed										1049
**Late ABF**										
p >.05										
p ≤ .05-.02	4900	4737								
p ≤ .01-.002							924 ✓			
p ≤ .001										
**Stat sig not reported	1058<br>5101<br>6128	2524✓<br>4740<br>7376	4735						6510	1654

* Formula for relative percent difference = (T2-T1/T1)(100)

**Abstracter judged increase, decrease, or no diff, but authors did not report statistical significance OR authors state results were significant or not significant, but did not report test

✓International study

Notes:

If both early and late data reported, we counted each period once (study gets two counts, one for early, one for late)

If p-value > 0.05 we counted as no difference irrespective of magnitude of effect

Appendix 31: Volume of Care Vote Count Table (Non-Pooled)

	Early ABF			Late ABF		
	Increase	Decrease	No diff/mixed	Increase	Decrease	No diff/mixed
Magnitude ≥ 5% and p ≤ .01		1719✓				
Magnitude ≥ 1% to < 5% and p ≤ .01						
Magnitude <1% and p ≤ .01;<br>or<br>Mag.≥ 5% and stat sig. not reported or mixed	477✓<br>4735<br>2524✓<br>4917<br>8497✓	1711<br>1775<br>3228✓<br>4740<br>7376		1058<br>5101<br>6128	2524✓<br>4740<br>7376	
Other results*	924✓	4207✓<br>4265	6774<br>6510<br>1015✓<br>2859✓<br>1049	4900<br>4735<br>924✓	4737	6510<br>1654

*Other results" refers to studies reporting combinations of magnitude and p-value other than the classifications in this table
✓International study
