# Supplementary material for: Activity-Based Funding of Hospitals and Its Impact on Mortality, Readmission, Discharge Destination, Severity of Illness, and Volume of Care: A Systematic Review and Meta-Analysis
Source: PLoS One. 2014 Oct 27;9(10):e109975. doi: 10.1371/journal.pone.0109975 (PMC4210200; doi:10.1371/journal.pone.0109975)
Supplement: Protocol S1 — (DOCX) [file pone.0109975.s003.docx]

# Protocol

Activity-Based Funding of Hospitals and its Impact on Mortality, Readmission, Discharge Destination, Severity of Illness, and Volume of Care: A Systematic Review and Meta-Analysis

Karen S Palmer, Thomas Agoritsas, Danielle Martin, Taryn Scott, Sohail M Mulla, Ashley P Miller, Arnav Agarwal, Andrew Bresnahan, Afeez Abiola Hazzan, Rebecca A Jeffery, Arnaud Merglen, Ahmed Negm, Reed A Siemieniuk, Neera Bhatnagar, Irfan A Dhalla, John N Lavis, John J You, Stephen J Duckett, Gordon H Guyatt

**Correspondence to:** kpalmer@sfu.ca

# 1.0 INTRODUCTION

Activity-based funding (ABF) is one of several possible payment systems for hospital funding.

In contrast to more traditional methods of hospital funding — such as negotiated funding through global budgets or block grants, per diem payments, or retrospective cost-based reimbursement — healthcare systems operating with ABF pay hospitals and non-hospital medical/surgical facilities per patient served according to a pre-determined fee that is calculated according to volume, type, and complexity of services/procedures.

The fee for each patient is based on prospectively classifying hospital services into clinically meaningful “bundles” or “episodes of care” that use similar levels of hospital resources. These bundled services are known by several terms internationally, including diagnosis-related groups (DRGs).

ABF is known by different names internationally, including payment-by-results (PbR) in the England; prospective payment system (PPS) or DRG-based funding in the United States; Patient-based Payment (Ontario); volume-based funding, payment-for-volume; and service-based funding.

ABF has three defining features:

1. A case mix classification system, such as DRGs, is used to describe bundled services;
2. A payment price is prospectively set for each case mix group in advance of the funding period (the “P” in PPS);
3. Payments to hospitals are made on a per case basis, irrespective of actual resources used or length of stay.

# 2.0 STUDY OBJECTIVE

The objective of this systematic review is to determine the impact of activity-based funding versus alternative funding methods of hospitals and non-hospital medical/surgical facilities on a variety of health system outcomes and to determine what factors may have influenced these effects.

Our systematic review includes 2 steps:

1. An overall systematic review on any outcomes related to: quality of care, access to care, equity, cost, length of stay, efficiency, mortality, readmission rates, and discharge destination.
2. The current systematic review reporting on the following outcomes: 1) Acute Care (AC) Mortality; 2) Post-Acute Care (PAC) Mortality; 3) Discharge to Post-Acute Care (PAC); 4) Hospital Readmission; 5) Severity of Illness; 6) Volume of Care.

# 3.0 METHODS

This study is a systematic review and meta-analysis, part of a larger systematic review in which more general eligibility criteria were used. **Figure 1** outlines the methodological steps for this systematic review.

## 3.1 Eligibility criteria

We will include studies that meet the following eligibility criteria.

### 3.1.1 Inclusion Criteria

1. The study addresses the impact of ABF versus alternative funding systems in hospitals and/or non-hospital medical/surgical facilities.
2. The study examines the effects of the implementation of ABF in acute care.
3. The study includes original quantitative data addressing one of the following outcomes: 1) Acute Care (AC) Mortality; 2) Post-Acute Care (PAC) Mortality; 3) Discharge to Post-Acute Care (PAC); 4) Hospital Readmission; 5) Severity of Illness; 6) Volume of Care.
4. The study appears from January 1980 until the date the search is completed.
5. Studies in any language, however only English search terms were used.
6. Any form of comparative study (e.g. before-after studies in single jurisdictions (before ABF vs. after ABF implementation), parallel group studies in multiple jurisdictions (no ABF jurisdiction vs. ABF jurisdiction), or a combination of both designs (e.g., difference-in-difference analyses, time-series).

The study contains a comparator with 0% ABF.

### 3.1.2 Exclusion criteria

1. Study includes original data about the functioning of an ABF system, but not data directly comparing ABF to an alternative system.
2. Studies examining the implementation of ABF in post-acute care, rather than implementation in acute care.
3. Studies where the focus of the publication is something other than ABF or a pre-specified related concept (e.g. Diagnosis-Related Groups (DRGs), or reported on pay-for-performance (P4P, a financial incentive for attaining targeted service goals).
4. Studies from PPS systems that we determined not to be DRG-based, or “per episode” of care, or “per case”, but instead were based on a flat per diem rate or other period of time, such as the Japanese Diagnosis Procedure Combination (DPC) system, Chinese Hainan system, and Medicare Resource Utilization Groups (RUGs).
5. Studies on activity-based costing (ABC) only (i.e. not also to ABF).
6. Studies that pertain to capital costs/acquisitions.
7. Other US non-Medicare studies (e.g. Veteran’s Administration, Medicaid)
8. Modeling studies that do not present data from both an ABF and no ABF group or include a modeled (hypothetical) comparison group.
9. Articles that include only qualitative data (mixed methods studies will be included if there is eligible quantitative data).
10. Studies that pertain to people’s opinions on ABF, even if the opinions are quantified (note: this does not pertain to patient satisfaction).
11. Publications that do not provide primary data, such as policy analyses, commentary pieces, newspapers, editorials, letters, notes, news, or other media release documents.
12. Books.

## 3.2 Study Identification

### 3.2.1 List of Databases and Search Strategy

1. Major databases including, but not limited to, MEDLINE, Embase, CINAHL, Business, Source Complete, EconLit, PAIS International, HealthSTAR, and the Cochrane Library, for peer-reviewed publications
2. Public domain grey literature sources, such as Google Scholar, Web of Science, Canadian Public Policy Collection, and international governmental and non-governmental databases
3. We will augment this search by employing a “snowball sampling” method, asking key informants identified from the grey literature, government or institutional contacts (both foreign and Canadian), and known stakeholders to provide further references or contacts pertaining to the subject matter. Through this process, we will identify “green” papers such as tentative government reports released by the European Commission, and authoritative “white” papers that may follow.
4. We will consult reference lists, personal files, selected websites, and experts to ensure no relevant articles are missed.
5. To avoid publication bias, we will include unpublished empirical studies as well.
6. We will search the citation index of retrieved studies to identify any additional potentially relevant studies.
7. In addition to the database search, expert reviewers identified important review and background articles. The references of these articles will be screened for any eligible articles that may not have been captured in our search. Selected websites and experts were also consulted to ensure no important references were missed.

### 3.2.2. Search Terms

1. Broadly define ABF, using relevant international terms and related concepts (such as Diagnosis-Related Groups - DRGs; Payment-by-Results – PbR; and Prospective Payment Systems - PPS);
2. Address the effects of ABF for in-patient and out-patient hospital and non-hospital medical/surgical care on health care system costs, quality of care, equity of care, and access to care, including a summary of quality and access measures used by other jurisdictions.

Initial sample search string may include, but will not be limited to (i.e. iterative search), some of the following terms:

Activity-based funding OR payment-by-results OR patient-focused funding OR prospective payment system OR case-mix funding OR service-based funding OR payment-for-volume OR volume-based funding OR diagnosis-related groups OR case-mix groups; AND hospital OR in-patient; OR out-patient; OR health care institution; OR surgical facility; OR medical facility; OR surgical centre; OR medical centre; OR treatment centre; AND economic analysis OR cost effectiveness analysis OR cost-benefit analysis OR mortality OR morbidity OR length-of-stay OR equity OR health care OR access OR waiting time, OR efficiency, OR technical efficiency, OR cost efficiency.

## 3.3 Titles and Abstract Screening

The purpose of screening is to examine titles and (when available) abstracts to determine whether or not the study meets eligibility criteria. Teams of two individuals will screen, with each screener independently evaluating all titles/abstracts assigned to their team.

Each screener will answer the following yes or no question:

*1. The study appears as if it may include original quantitative data that allows an evaluation of the relative impact of ABF versus alternative funding systems in hospitals and/or non-hospital medical/surgical facilities on any indicators of* quality of care, access to care, equity, cost, length of stay, efficiency, *mortality, readmission rates, and discharge destination.*

- *Yes*
- *No*

Screeners will be members of the study team. We will err on the side of inclusivity and if either one of the screening pair considers the article potentially eligible, the article will advance to full text screening.

## 3.4 Full-Text Screening

At this stage reviewers will screen all potentially eligible full texts. Unlike title/abstract screening, which looked at all potential outcomes in our overall systematic review (see 3.3), full-text screening for the current review will focus on how ABF affects six key measures of quality, access, and equity, each with the potential to affect patients and health care system capacity: both acute care mortality (AC mortality); and post-acute care mortality (PAC mortality); readmission rates; discharge destination measured by discharge to post-acute care (PAC) following hospitalization. In studies eligible for this review that report on at least one of the three primary outcomes of mortality, readmission rates, and discharge destination, we will also abstract for two secondary outcomes, severity of illness and volume of care:

Screeners will answer “yes” or “no” to the following question:

*The study includes original quantitative data that allows an evaluation of the relative impact of ABF versus alternative funding systems in hospitals and/or non-hospital medical/surgical facilities on mortality, readmission rates, discharge destination.*

- *Yes*
- *No*

Reviewers will check for all eligibility criteria listed in section 3.1. The detailed list of rules on how to apply each inclusion and exclusion criteria can be found at the end of this document (**Appendix 1**: Detailed Screening Rules),

To be considered as eligible for the current review, outcomes must correspond to the following definitions (see also section 3.6.3).

1. *Acute Care Mortality (AC Mortality)* is defined as death rate per population per common time period in the ABF and no ABF comparator, starting at admission to acute care (or at surgery). We will exclude mortality only measured from discharge as well as in-hospital mortality only, since the potential influence of ABF may be confounded by concurrent differences in length of stay. We will also exclude population-level mortality rates, measured only in the general population, such as overall crude mortality rate.
2. *Post Acute Care Mortality (PAC Mortality)* is defined similarly to acute care mortality, except that we will look at death rate starting at admission to post acute care, following a stay in acute care. For this variable, we will include only studies in which it was clear whether the stay in acute care occurred before or after ABF was implemented. We will measure PAC mortality to determine whether sicker patients at greater risk of death were being discharged to PAC.
3. *Hospital Readmission* is defined as readmission rates per population per common time period (preferentially at 30 days) in the ABF and no ABF comparator, as an indicator of quality of care.
4. *Discharge Destination* is defined as the proportion of patients discharged alive from acute to post-acute care (PAC), rather than to home. Depending on the country, PAC included (but was not limited to): intermediate care facilities, nursing homes, or similar PAC facilities such as those designated under the US Centers for Medicare and Medicaid (i.e. skilled nursing facilities (SNF); in-patient rehabilitation facility (IRF); long-term care hospital (LTCH); home health agency (HHA)). If data were reported in the same study for different PAC destinations, we aggregated them for our analysis. We measured this variable as an indicator of burden to the health care system.
5. *Changes in the distributions of severity of illness* between ABF and no ABF comparators (e.g. differences in case mix, diagnostic codes, DRG points, or number of patients with comorbidities). We considered this outcome as a measure of access, reflected as adverse risk selection (e.g. “case mix dumping”, “cherry picking”), or differences in coding that may represent legitimate differences in patient severity, or may not (e.g. fraudulent upcoding, “DRG creep”). To include data on severity of illness, we need to know the distribution of patients or other denominator measure in both the ABF and the ABF-comparison groups, not just a modelled (hypothetical) comparison.
6. *Changes in volume of care* between ABF and no ABF comparators. This we considered an indicator of access to care, as measured by the number of patients treated or admitted, or the number of procedure or tests performed. To include volume of care data, we need the volume of the patients or other denominator measure in both the ABF and the ABF-comparison groups, not just a modeled (hypothetical) comparison.

Each person in a screening pair will independently evaluate all assigned full text papers for eligibility; disagreements will be resolved by consensus. When screening conflicts occur, the screening partners will discuss the reasoning for their decisions, and if one screener realizes she/he has made an error then the process will be complete. If neither screener thinks she/he has made an error, then an independent reviewer who was not on the title/abstract screening pair will review the article and adjudicate. That reviewer’s decision will be final. A citation index or full-text copy of all articles that pass to full text review, whether included or excluded, will be kept and provided to readers upon request.

To ensure reliability, the full text screening process will begin by calibrating all screeners. We will choose ten studies that appear difficult in determining eligibility. All reviewers will independently adjudicate these ten studies and send their decisions to the Research Coordinator. In a conference call, we will discuss reasons for disagreement. We will repeat this calibration process with additional challenging studies until we are satisfied with the level of inter-rater reliability.

## 3.5 Credibility assessment

Teams of two individuals will independently review the methodology of all studies that fulfill our eligibility criteria. We will record information about the study design. Specifically, we will assess: 1) the source and documentation of data quality; 2) the number of variables simultaneously examined in the study (mortality, readmission, discharge destination, severity of illness, volume of care, and characteristics of study population); 3) the appropriateness and comprehensiveness of the statistical adjustment for each outcome of interest. We will provide pre-specified guidance on what variable adjustments were appropriate or inappropriate for each outcome, and response categories will be as follows: “comprehensive adjustment and all factors appropriate”; “limited adjustment or at least one factors is inappropriate”; or “minimal adjustment or more than one factor is inappropriate”. For example, in order to be considered comprehensive, the analysis of mortality or readmission data will need to be adjusted for age, sex and at least one measure of severity of illness of comorbidities, whereas adjustment for length of stay was deemed inappropriate

Each response for all of the credibility questions will be assigned a points value that will be factored into a composite credibility score (see **Table 1** for scoring details). By simply summing the points, the score ranges from 0 (lowest credibility) to 6 (highest credibility).  We will use a cut-off of ≥ 4 for high credibility. Therefore studies with a score of <4 will be considered low credibility.

All disagreements on credibility assessment will be resolved by discussion between the abstractors. If a disagreement occurs, the abstractors will discuss the reasoning for their decisions, and if one reviewer realizes she/he has made an error then the process will be complete. If neither reviewer thinks she/he has made an error, then an independent reviewer who was not on the abstracting team will review the article and adjudicate.

## 3.6 Data Abstraction

From all eligible studies, we will abstract data on the following variables: 1) AC mortality; 2) PAC mortality; 3) discharge to PAC; and 4) hospital readmission 5) severity of illness; and 6) volume of care.

Using standardized data abstraction forms custom built in DistillerSR™ and adapted to each study design, paired reviewers will independently abstract the following data, from all studies that fulfill our eligibility criteria: country and year of ABF implementation, data source, sampling methods, study population (type and number of patients and institutions), outcomes assessed, and results.

The abstraction forms will be pilot-tested among reviewers and revised based on issues encountered. To ensure reliability and reproducibility of the abstraction process, we will generate detailed instructions for each item in the data abstraction forms (see **Appendix 2**: General instructions for completing the data abstraction form)

Data will be abstracted in duplicate; differences will be resolved by consensus, or adjudicated by a senior team member if consensus cannot otherwise be reached. We will contact authors of the original papers if we identify any missing data.

### 3.6.1 Poolable Data Abstraction

If adequate data are available, we will separately pool and meta-analyze AC mortality, PAC mortality, hospital readmission, and discharge destination data. We pre-specified a set of conditions necessary to pool reported data in a meta-analysis. We will follow the algorithm in **Figure 2** to determine if data are eligible for pooling.

Data have to be either detailed unadjusted frequencies in the ABF and no ABF group, or combined effect estimates with: 1) a measure of variation (e.g. standard deviation, variance); or 2) sufficient statistical information (e.g. standard error, confidence intervals, exact p-values) along with exact number of patient at risks. Absent these criteria, or when the impact of ABF is reported separately for specific sub-groups, or reported only as interaction terms with other variables in complex regression models or time-series, we will consider data to be non-poolable. The study statistician will make the final decision as to whether abstracted data can be pooled.

### 3.6.2 Non-Poolable Data Abstraction

For severity of illness and volume of care outcome variables, as well as non-poolable data for AC mortality, PAC mortality, hospital readmission and discharge to PAC, data analysis will consist of constructing objective, comprehensive narrative summaries of results for each study.

This narrative summary will include information on country, study design, data collection dates, primary data source and sampling method, study population and sample size, overall credibility, and a narrative summary of the main findings (See **Table 2** for an example of how data will be presented).

Tables will be populated based on abstracted data. Abstractors will only include information in their summaries that is based on data provided by authors. If authors provide only a narrative summary or commentary of their interpretation of their data, but do not provide the data on which these inferences are based, the information will not be abstracted. If the data abstractors are uncertain of what to include, they will consult with a senior member of the team. All data will be checked by a senior member of the team.

*3.6.3 Outcome-specific Abstraction*

### Acute Care Mortality

We will define AC mortality as mortality that is measured over a fixed period of time, starting at (or near) patients admission to acute care. In order to be eligible, AC mortality has to be measured over any common time period for each of the study groups (e.g. 30 days, 60 days, 90 days, etc.). We will not include studies that only measure in-hospital mortality, or measure mortality only from the time of discharge. The rationale is that we suspect that ABF decreases length of stay which may impact in-hospital mortality, so in-hospital mortality that does not also include mortality soon after discharge will be artificially low if more patients die outside of hospital due to shortened length of stay after ABF. We will also exclude population-level mortality rates, measured only in the general population, such as overall crude mortality rate, as these go far beyond the acute care setting where ABF is implemented. If acute care mortality data are presented as a mean number of deaths over a specified population, we will report these results in the non-poolable tables, but will not pool this data.

If an article provides data for acute care mortality over multiple periods of time (e.g. 15 day and 30 day mortality), we will abstract mortality data for only one period as per the following hierarchy:

1. 90 day
2. 60 day
3. 30 day
4. Other time period (if multiple other periods abstract from the longest time period)

For acute care mortality we are most interested in abstracting adjusted data because we suspect that ABF may change case mix. Therefore, if a study provides both an adjusted and unadjusted analysis, we will only abstract the adjusted analysis. If a study only provides one type of analysis (i.e. adjusted or unadjusted), we will abstract data from whichever type of analysis is provided, and will record whether the analysis was adjusted or unadjusted.

### Post-Acute Care Mortality

We will define post-acute care mortality as mortality that is measured over a fixed period of time, starting at (or near) patients admission to post-acute care. In order to be eligible, post-acute care mortality had to be measured over any common time period for each of the study groups (e.g. 30 days, 60 days, 90 days, etc.). If an article provides data for PAC mortality over multiple periods of time (e.g. 15 day and 30 day mortality), we will abstract mortality data for only one period as per the following hierarchy:

1. 90 day
2. 60 day
3. 30 day
4. Other time period (if multiple other periods abstract from the longest time period)

For PAC mortality we are most interested in abstracting adjusted data because we suspect that ABF may change case mix. Therefore, if a study provides both an adjusted and unadjusted analysis, we will only abstract the adjusted analysis. If a study only provides one type of analysis (i.e. adjusted or unadjusted), we will abstract data from whichever type of analysis is provided, and will record whether the analysis was adjusted or unadjusted.

1. *Hospital Readmission*

We will define hospital readmission as any acute care re-hospitalization after a previous acute care hospitalization. We will include data assessing hospital readmission over any time period. If an article provides data for readmission over multiple periods of time (e.g. 15 day and 30 day readmission), we will abstract the data for only one period as per the following rules:

1. If multiple readmission periods each ≤ 30 days, abstract the latest readmission data (e.g. if 30 days, 15 days, and 5 days, abstract 30 days)
2. If multiple readmission periods each > 30 days, abstract the earliest readmission data (e.g. if 45 days and 60 days, abstract 45 days)
3. If multiple readmission periods with some ≤ 30 days and some > 30 days, abstract the readmission data closest to 30 days (e.g. if 14 days, 30 days, and 60 days, abstract 30 days)

For hospital readmission we are most interested in abstracting adjusted data because we suspect that ABF may change case mix. Therefore, if a study provides both an adjusted and unadjusted analysis, we will only abstract the adjusted analysis. If a study only provides one type of analysis (i.e. adjusted or unadjusted), we will abstract data from whichever type of analysis is provided, and will record whether the analysis was adjusted or unadjusted.

1. *Discharge Destination*

We will define discharge destination as the level of care to which a patient is discharged after receiving acute care (i.e. home, post-acute, dead). Discharge destination will be abstracted as a dichotomous variable whenever possible (i.e. discharged to post-acute care vs. not discharged to post-acute care). If data are presented in other ways we will try to convert the data to a dichotomous measure of discharge destination, according to the abstraction algorithm detailed in **Figure 3**).

For discharge destination, we are equally interested in adjusted and unadjusted data. The rationale is that ABF could lead to increases in discharge to PAC for two reasons: 1) a decrease in length of hospital stay and earlier discharge of patients still not well enough to go home; or 2) a change in case mix arising from a change in characteristics of patients admitted to hospital in the first place. An unadjusted analysis would inform in particular on whether a jurisdiction adopting ABF might face an increased burden to PAC facilities.

1. *Severity of Illness*

Severity of illness will be defined as any difference in apparent distribution of severity of illness. **Table 5** provides examples of variables that we consider indicators of this outcome. For severity of illness, we are most interested in unadjusted data. Therefore, if a study provides both an unadjusted and adjusted analysis, we will only abstract the unadjusted analysis. If a study only provides one type of analysis (i.e. adjusted or unadjusted), we will abstract data from whichever type of analysis is provided.

1. *Volume of Care*

Volume of care will be defined as degree of service availability and wait times for services. **Table 5** provides examples of variables that we are considering indicators of this outcome. For volume of care, we are equally interested in adjusted and unadjusted data.

### 3.6.4 Abstracting Articles Containing Multiple Data Points

For all study variables, some studies report data for many time periods before and after ABF. It is not feasible to abstract data from all time points so the following rules will be used to determine what data should be abstracted:

1. We will abstract data from three time periods: 1) No ABF; 2) Early ABF; 3) Late ABF.
2. No ABF will be defined as the data point furthest away from implementation of ABF, up to 3 years before ABF implementation.
3. Early ABF will be defined as the soonest time point after ABF implementation.
4. Late ABF will be defined as the furthest point after ABF implementation, up to 5 years after ABF implementation.

Specifically we will abstract 3 data points (if available):

1. No ABF - We will abstract the data point furthest away from implementation of ABF up to three years before ABF.
   - Example A: In a US study (year of ABF implementation is 1983) that provides AC mortality rates for the years 1979, 1980, 1981, 1982, 1984, 1985, 1986, 1987, 1988, 1989, 1990, 1991 we would abstract data from 1980 for the No ABF period.
   - Example B: In a US study (year of ABF implementation is 1983) that provides AC mortality rates for the years 1981, 1982, 1984, and 1985, we would abstract data from 1981.
   - Example C: In a US study (year of ABF implementation is 1983) that provides AC mortality rates for the years 1982 and 1984, we would abstract data from 1982.
2. Earliest ABF – we will abstract the data point at the soonest time point after ABF. We will only abstract data for this data point if there are two data points reported after ABF implementation. For example, in example A we would abstract data from 1984, in example B we would abstract data from 1984 and in example C we would not abstract any data.
3. Latest ABF – we will abstract the data point at the furthest reported point after ABF, up to five years after ABF was implemented. For example, in example A we would abstract data from 1988, in example B we would abstract data from 1985, and in example C we would abstract data from 1984.

The rationale for selecting a “No ABF” data point that is the furthest away from implementation of ABF (up to three years) is that the anticipation of ABF implementation may invoke system changes as facilities anticipate and prepare for implementation). The rationale for collecting an “Early” and a “Late” after ABF data point is that this will allow us to examine both the short and long-term effects of ABF.

# 4.0 *A PRIORI* HYPOTHESES TO EXPLAIN DIFFERENCES IN OUTCOMES

It is likely that individual studies will yield quantitatively, and possibly qualitatively, different results. A key issue for the review will be to attempt to explain these differences. Potential explanations are more credible if investigators offer them as *a priori*, rather than *post hoc*, hypotheses. We hypothesized *a priori* that variability in results across studies might be due to the following: study location (US vs. international); study design (before-after vs. parallel-controlled study); time after ABF implementation (2 years or less vs. more than 2 years); analysis (adjusted vs. unadjusted); credibility (higher - 4 or more on the credibility - score vs. lower); and time of assessment of mortality or readmission (within 30 days vs. more than 30 days). Other factors may explain variability but are unlikely to be available in published reports, and can therefore not be examined in sub-group analyses.

# 5.0 DATA ANALYSIS

## 5.1 Non-Pooled Data Analysis

We will analyze each outcome of interest using a structured analysis format and vote counting exercise to identify the direction of effect, magnitude of effect, and significance of effect of ABF. Finally this detailed vote counting will be synthesized in a summary of findings. We will present results in a narrative and tabular form.

Two abstractors will independently assess the outcome direction, magnitude, and statistical significance, which will be further checked by a third reviewer and confirmed by a fourth. We will summarize outcome directions (increase, decrease, mixed, no difference); magnitude (≥ 5%; ≥ 1% to < 5%; <1%; indeterminate or mixed); and statistical significance (p>.05; p≤ .05-.02; p≤.01-.002; p≤.001; p-value not reported) across studies, and then stratify results by US vs. international studies, early vs. late after ABF implementation, and by higher vs. lower credibility.

We will report results of each study in three tables:

1) A narrative summary table will report comprehensive results from the data that reviewers abstracted (**Table 2)**. When populating this table we will follow a pre-specified set of rules (**Table 6**).

2) A non-pooled analysis table will categorize and analyze these narrative data (**Table 3**); and

3) A non-pooled vote count summary table will present a summary of the data according to

direction, magnitude, and statistical significance) (**Table 4**).

## 5.2 Pooled Data Analysis

During abstraction, abstractors will determine whether or not the data are potentially eligible for pooling. Decisions will be made based on the algorithm in **Figure 2**. The study statistician will make the final decision as to whether or not data may be pooled.

For studies that report poolable data, we will conducted a quantitative meta-analysis of four dichotomous outcomes (AC mortality, PAC mortality, hospital readmission, discharge to PAC) using random effects models. When two time points (early and late ABF) are reported after ABF was implemented, we will use the latest in the pooled analysis. When both adjusted and unadjusted data are reported, we will use adjusted data. When either adjusted or unadjusted data were reported, and we will use whichever data were available for our primary meta-analysis, and analyze the impact of adjustment as a pre-specified subgroup analysis (see below).

## 5.3 A Priori Subgroup and Sensitivity Analyses

If data permit, we will conduct the subgroup analyses, specified is section 4.0 to assess for heterogeneity.

## **Tables and Figures**

## **Table 1:** Credibility Assessment Scoring

| **Item and Responses** | **Points** |
| --- | --- |
| Appropriateness and comprehensiveness of the statistical adjustment |  |
| *Comprehensive adjustment and all factors appropriate  (high credibility)* | 2 |
| *Limited adjustment or at least one factors is inappropriate* | 1 |
| *Minimal adjustment or more than one factor is inappropriate  (low credibility)* | 0 |
| Source and documentation of data quality |  |
| *Yes (high credibility)* | 3 |
| *Mostly yes* | 2 |
| *Mostly no* | 1 |
| *No (low credibility)* | 0 |
| Number of variables |  |
| *≥3* | 1 |
| *<3* | 0 |

## **Table 2:** Example of Narrative Summary Table

| **Author  (year)** | **Country  (start year ABF)** | **Study Design** | **Data Dates**  **(% funding from ABF)⁺** | **Primary data source**  **Sampling method** | **Study population**  **(N patients / N institutions)** | **Overall Credibility Score (0-6)** | **Variable and main findings**  (outcome direction, magnitude, statistical significance) |
| --- | --- | --- | --- | --- | --- | --- | --- |
| **US** | | | | | | | |
|  |  |  |  |  |  |  |  |
| **International** | | | | | | | |
|  |  |  |  |  |  |  |  |

## **Table 3:** Example of Non-Pooled Analysis Table

| **Significance of Effect** | | **Magnitude ≥ 5%*** | | **Magn ≥ 1% to < 5%** | | **Magn <1%** | | **Magn Indeterminate or mixed** | | | |
| --- | --- | --- | --- | --- | --- | --- | --- | --- | --- | --- | --- |
|  |  | **Direction** | | | | | | | | | |
|  |  | **inc** | **dec** | **inc** | **dec** | **inc** | **inc** | **dec** | **inc** | **dec** | **Direction Mixed** |
| **Early ABF** | | | | | | | | | | | |
| p >.05 | |  |  |  |  |  |  |  |  |  |  |
| p ≤ .05-.02 | |  |  |  |  |  |  |  |  |  |  |
| p ≤ .01-.002 | |  |  |  |  |  |  |  |  |  |  |
| p ≤ .001 | |  |  |  |  |  |  |  |  |  |  |
| ⁺Stat sig not reported | |  |  |  |  |  |  |  |  |  |  |
| **Late ABF** | | | | | | | | | | | |
| p >.05 |  | |  |  |  |  |  |  |  |  |  |
| p ≤ .05-.02 |  | |  |  |  |  |  |  |  |  |  |
| p ≤ .01-.002 |  | |  |  |  |  |  |  |  |  |  |
| p ≤ .001 |  | |  |  |  |  |  |  |  |  |  |
| ⁺Stat sig not reported |  | |  |  |  |  |  |  |  |  |  |

## **Table 4:** Example of Non-Pooled Vote Count Summary Table (direction, magnitude, and statistical significance)

|  | **Early ABF** | | | **Late ABF** | | |
| --- | --- | --- | --- | --- | --- | --- |
|  | **Increase** | **Decrease** | **No diff/mixed** | **Increase** | **Decrease** | **No diff/mixed** |
| Magnitude ≥ 5% and p ≤ .01 |  |  |  |  |  |  |
| Magnitude ≥ 1% to < 5% and p ≤ .01 |  |  |  |  |  |  |
| Magnitude <1% and p ≤ .01;  or  Mag.≥ 5% and stat sig. not reported or mixed |  |  |  |  |  |  |
| Other results* |  |  |  |  |  |  |

## **Table 5:** Examples of Non-Poolable Outcome Variables

| **Stratifying Question** | **Associated Variables** |
| --- | --- |
| **Volume of Care** | - Differences in service availability:  1. Differences in regional/geographic availability of services 2. Differences in average number of available beds 3. Differences in volume of care measured by number of patients treated/admitted  - Differences in wait times |
| **Severity of Illness** | - Difference in apparent distribution of severity of illness/casemix (as possible measure of DRG “creep” or risk selection/case mix dumping/cherry picking, or more appropriate coding) in ABF vs non-ABF periods:  1. Diagnostic codes (e.g. ICD 10 codes, DRG points produced) 2. Number of patients with co-morbidities |

**Table 6:** Main Finding Reporting Rules (Non-Pooled Narrative Summaries Rules)

| 1. | If multiple measures and multiple directions, report the direction with *≥* 75% or *≥* 3:1 (e.g. if *≥* 75% increase, then report increase) |
| --- | --- |
| 2. | If multiple measures and multiple magnitudes, report the magnitude with *≥* 75% or *≥* 3:1 (e.g. if *≥* 75% increase, then report increase); if no clear winner then magnitude is mixed |
| 3. | If < 75% or < 3:1, then report mixed direction in summary. |
| 4. | When significance is mixed, report as mixed |
| 5. | When magnitude is mixed, report as indeterminate |
| 6. | When direction is no difference, then magnitude is irrelevant, so don’t report magnitude |
| 7. | If multiple data points, summarize each separately as “outcome summary” and then add “overall outcome summary”. |
| 8. | For Readmission: if multiple periods are abstracted, each > 30 days, report earliest data. (Though the abstraction rule for non-poolable was to abstract all periods, we will only report earliest period, which is consistent with the abstraction rule for poolable data) |
| 9. | Discharge Destination means “Discharge to PAC”. As such, “discharge home” is not reported. |
| 10. | P-value categories: p >.05; p ≤ .05-.02; p ≤ .01-.002; p ≤ .001; Stat sig not reported |

## **Figure 1:** Flowchart of Methodological Steps

## **Figure 2:** Algorithm for determining whether to abstract poolable data


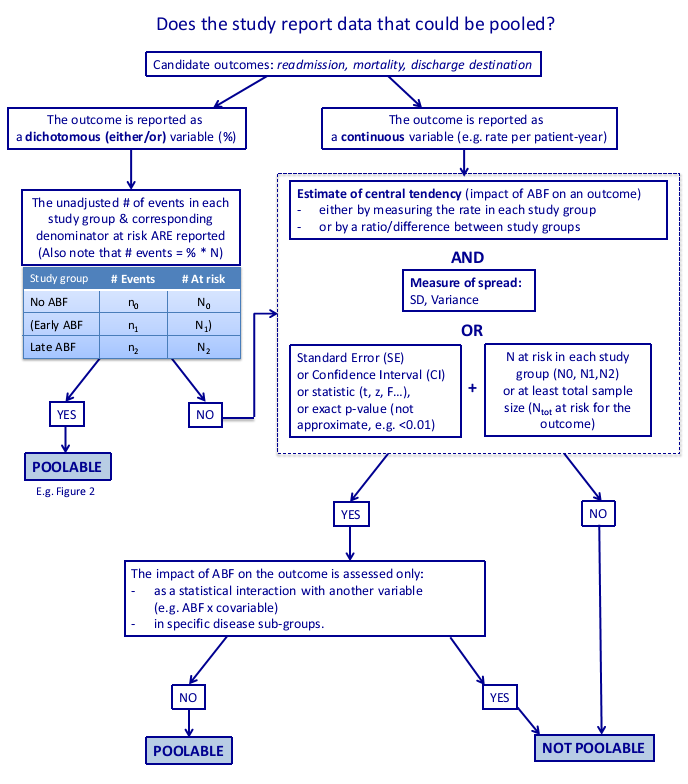


## **Figure 3:** Discharge destination data abstraction algorithm

##

## **Appendix 1: Detailed Screening Rules**

**Title and Abstract Screening (see section 3.3)**

Screening Question:

*1. The study appears as if it may include original quantitative data that allows an evaluation of the relative impact of ABF versus alternative funding systems in hospitals and/or non-hospital medical/surgical facilities on any indicators of* quality of care, access to care, equity, cost, length of stay, efficiency, *mortality, readmission rates, discharge destination.*

- *Yes*
- *No*

**Rule:** Err on the side of inclusivity and include any study that contains variables that may reasonably be considered an index of the outcomes of interest

**Full Text Screening (see section 3.4)**

Screening Questions:

*1. The study includes original quantitative data that allows an evaluation of the relative impact of ABF versus alternative funding systems in hospitals and/or non-hospital medical/surgical facilities on mortality, readmission rates, discharge destination..*

- *Yes, complete questions 2 and 3*
- *No, form is complete*

*2.  Does the article indicate that identical data with identical analysis may have been published in a different source?*

- *Yes*

**Rules:**

*Note*: The purpose of title and abstract/full text screening is to determine whether the study meets the criteria laid out in the screening forms, not the credibility of the study. Quality will be assessed using the credibility form.

1. If there is doubt whether the article includes original data (i.e. it is uncertain whether the data included come from another article) answer "Yes" to item 1 on the Full Text Screening Form. (Originated from OECD article, calibration 1). Final eligibility will be subsequently checked with senior members in the team.
2. If the article includes original data about the functioning of an ABF system, but not data directly comparing either ABF to an alternative system; (Amendment originated from refID 1612; Latta & Helbing, 1991).
3. If the article includes only qualitative data, answer “NO” to item 1 on the Full Text Screening Form. (Originated from RefID 2594; Sussex & Farrar, 2009).
4. If the article includes mixed-methods and there is a quantitative component presenting results for one of our outcomes, answer "Yes" to item 1 on the Full Text Screening Form. (Originated from RefID 2594; Sussex & Farrar, 2009).
5. To include an outcome as eligible we need the volume of the patients OR other denominator measure in both the ABF and the ABF-comparison groups, not just a modelled (hypothetical) comparison.
6. If the study pertains only to Activity-Based Costing (ABC) and not ABF, answer "No" to item 1 on the Full Text Screening Form. (Originated from RefID 2882; Young, 2007).
7. If there is uncertainty whether a comparison is between different periods of ABF funding vs. ABF funding plus a period of alternative funding, assume the latter and answer “Yes”. Final eligibility will be subsequently checked with senior members in the team.
8. If the study pertains to the Japanese Diagnosis Procedure Combination (DPC) system and not to Japanese DRG system, answer “No” to item 1 on the Full Text Screening Form.
9. If the study pertains to the Chinese Hainan system, answer “No” to item 1 on the Full Text Screening Form. (originated from refID 9200)
10. If data are previously published, but re-analyzed in a different way, consider these original data and answer "Yes" to item 1 on the Full Text Screening Form. (Reason for rule: OECD paper, Calibration 1).
11. If the data have been previously published, but in a duplicate source that may not have been included in our search answer "Yes" to item 1 and "Yes" to item 2 on the Full Text Screening Form. (Reason for rule: Refid 1815; May & Wasserman, 1984).
12. If the study reports on the US Interim Payment System (IPS) for Home Health Agencies (HHA), and if the outcomes measured in the study are among those we are studying, answer "Yes" to item 1 on the Full Text Screening Form.
13. If the modeling study presents data from both pre/post, answer “Yes” to item 1 on the Full Text Screening Form.
14. If the modeling study does NOT present data from both the pre/post, answer “No” to item 1 on the Full Text Screening Form.
15. If the funding in an acute care setting is not PPS based on DRGs (or a similar activity-based system), answer “No” to item 1 on the Full Text Screening Form. (Originated from refID 7265; Norton).

If the study pertains to people’s opinions on ABF, even if the opinions are quantified, answer “No” to item 1 on the Full Text Screening Form. (originated from refID 2249)

## **Appendix 2: *General instructions for completing the data abstraction form***

1. Go to <https://systematic-review.ca/Login/Login.php> to log into DistillerSR.
2. Under “Data Abstraction Section I: Study Demographics” click on the blue number.
3. If you have been assigned a specific set of refID’s, enter the refID of the first reference on your abstraction list. Click “Go”. Click on the reference that appears.
4. Click the blue link under “attachments” and the full text will open in an adjacent tab.
5. Complete the data abstraction form.

*Note: If after referring to the specific instructions section, you are still unsure of the answer to a question please consult with a senior member of the team (Taryn copying Karen).*

1. Once you have completed the Data Abstraction Form, choose “submit form”. Close the window and return to Distiller’s main menu. Repeat steps 2-5 to begin screening the next article on your list.

*Note: If you have missed any mandatory questions Distiller will not let you proceed. If Distiller has flagged a question and you do not know why, consult the manual to see if there are specific rules for how to answer the question.*

1. Once an article has been completed, it will not appear in the list of available articles awaiting your review. If you wish to go back to completed articles, go the “review” tab at the top left of the main screen. Click on “data abstraction” from the drop-down menu and select the section you would like to view. Change the filter item at the top of the articles list from “unreviewed” (this is the default) to “reviewed” in the drop down menu. Click “Go”. You can then select any article that you have previously reviewed and edit your answers. To edit an answer simply change the answer to one or more questions and click submit.
2. To facilitate conflict resolution, if you are uncertain of a response, record justification for response (e.g. either cut-and-paste or use other software (e.g. Adobe Professional) to highlight relevant section directly). For instructions on highlighting in Adobe see: see <http://www.wikihow.com/Highlight-Text-in-a-PDF-Document>.

**Saving:** Please note that you cannot save partially completed sections of the data abstraction form. If you exit the form, or click “skip to next” before you have completed all information for a section you will lose your work. If you do not have time to abstract all of an article in one session, complete the section you are in and click submit. When you resume abstraction for this article, pick up in the section following the one where you left off.

*General note: Some items in the abstraction form were removed as they do not apply to this review. This explains the presence of missing values in the numbering of the items*

***General Rules***

1. Answer all questions based on data that pertain to the variables of interest, not the study in general. For example, if the study looks at length of stay and mortality and the sample size for length of stay is 5,000 and for mortality it is 10,000, record 10,000 as the sample size because length of stay is not a variable of interest and mortality is.
2. If a study presents multiple sub-group analyses for a variable of interest, abstract data from the analysis that best meets the following criteria: i) contains a 0% ABF comparator; ii)provides the most relevant policy comparison as perceived by the data abstraction pair.
3. For all tables, enter as much information as you can from what is provided in the article.
4. If abstractors encounter issues abstracting, they are to first review the manual very carefully. If information is not in the manual, they are to email Taryn and Karen. **Exception:** if there is a question about whether to abstract variable *x* or *y*, contact your partner to discuss before abstracting.
5. 100% of articles from which we are abstracting will contain at least some primary data or original analysis of secondary data. Otherwise, the article would not have passed full text screening. If it is clear that some data in the article do not represent primary data or original analysis of secondary data, we will not abstract these particular data because we can assume we have captured the original source in our search. If it is unclear whether some data are primary data or an original analysis of secondary data, abstract the data.
6. For all studies included in the review, we will look specifically for the presence of upcoding of specific DRGs. In situations where the presence of upcoding is clear and unambiguous for specific DRGs, the data will be ineligible for pooling. If otherwise eligible these data will be included with the non-poolable results.
7. For both poolable and non-poolable outcomes, we will abstract 3 data points (if available):
8. No ABF - We will abstract the data point furthest away from implementation of ABF up to 3 years before ABF.
   - **Example A:** In a Medicare study (year of ABF implementation is 1983) that provides mortality rates for the years 1979, 1980, 1981, 1982, 1984, 1985, 1986, 1987, 1988, 1989, 1990, and 1991, we would abstract data from 1980 for the No ABF period.
   - **Example B:** In a Medicare study (year of ABF implementation is 1983) that provides mortality rates for the years 1981, 1982, 1984, and 1985, we would abstract data from 1981 for the No ABF period.
   - **Example C:** In a Medicare study (year of ABF implementation is 1983) that provides mortality rates for the years 1982 and 1984, we would abstract data from only 1982 for the No ABF period.
9. Earliest ABF – We will abstract the data point at the soonest time point after ABF. We will only abstract data for this data point if there are two data points reported after ABF implementation. For example, in example A we would abstract data from 1984, in example B we would abstract data from 1984 and in example C we would not abstract any data.
10. Latest ABF – We will abstract the data point at the furthest reported point after ABF, up to five years after ABF was implemented. For example, in example A we would abstract data from 1988, in example B we would abstract data from 1985, and in example C we would abstract data from 1984.

**Rationale**: We decided to abstract the furthest point up to 3 years before implementation of ABF, since anticipation of ABF implementation may invoke system changes as facilities anticipate and prepare for implementation. By collecting two data points after the implementation of ABF, we can examine both the short and long-term effects of ABF.

1. When the percent ABF is not the same throughout one calendar year, we will use the percent ABF that was present for the majority of the year. For example, in the US January to September of 1984 was 25% ABF and October to December was 50% ABF. Based on this rule, we will record 1984 as 25% ABF. We will note this rule in a footnote below the table. The exception to the rule is the year 1983 for US Medicare studies. We will treat 1983 as “before ABF” unless the author explicitly notes that s/he considers 1983 as “after ABF” or notes that the data were collected after October 1983, in which case it will be treated as “after ABF.”
2. For readmission and mortality, we are most interested in the adjusted analysis. Therefore, if an article reports both adjusted and unadjusted analyses (or more or less adjusted analyses), we will only abstract data from the most adjusted analysis. If only an unadjusted analysis is reported, we will abstract data from the unadjusted analysis. When abstracting, we will note whether the data are from an unadjusted or adjusted analysis. If it is adjusted we will abstract the variables for which the analysis adjusted. For abstraction, we will collapse the “unadjusted” and “less adjusted” rows into one row because unadjusted analyses are a special kind of “less adjusted” analyses. We will be able to determine if the analysis is adjusted or unadjusted based on whether adjustment variables are abstracted.
3. For discharge destination and volume of care, we are equally interested in the adjusted and unadjusted analyses (or the more and less adjusted analyses). Therefore, if an article reports both adjusted and unadjusted analyses (or more and less adjusted analyses), we will abstract data from the most adjusted analysis in Distiller and the unadjusted/less adjusted data outside of Distiller. If only an unadjusted or unadjusted analysis is reported, we will abstract data from whichever analysis is reported. When abstracting, we will note whether the data are unadjusted or adjusted. If it is adjusted, we will abstract the variables for which the analysis is adjusted. For abstraction, we will collapse the “unadjusted” and “less adjusted” rows into one row because unadjusted analyses are a special kind of “less adjusted” analyses. We will be able to determine if the analysis is adjusted or unadjusted based on whether adjustment variables are abstracted.

**Rationale:** Let us assume that the whole increase in patients being discharged to PAC is mediated by a change in the population. If that were the case then after you adjusted for changes in population characteristics you would find that the difference disappeared. An adjusted analysis under these circumstances would conclude that, after adjustment, the number of patients discharged to PAC is the same. However, an unadjusted analysis might conclude that the number of patients discharged to PAC has increased. It is possible that changes in population characteristics could be a result of ABF and therefore we want to abstract both adjusted and unadjusted analyses. The same scenario could be true for volume of care.

1. For characteristics of study population and severity of illness, we are most interested in the unadjusted analysis. Therefore, if an article reports both adjusted and unadjusted analyses (or more and less adjusted analyses), we will only abstract data from the least adjusted analysis. If only an adjusted analysis is reported, we will abstract data from the adjusted analysis. When abstracting, we will note whether the analysis is unadjusted or adjusted. If it is adjusted, we will abstract the variables for which the analysis is adjusted. For abstraction we will collapse the “unadjusted” and “less adjusted” rows into one row because unadjusted analyses are a special kind of “less adjusted” analyses. We will be able to determine if the analysis is adjusted or unadjusted based on whether adjustment variables are abstracted.
2. When abstracting non-poolable data, abstractors should record both: 1) key findings, and 2) a very short summary of the inference we should make from the data (aka “summary conclusions”). Ideally, **summary conclusions should be just a single sentence**. Data *plus* summary conclusions should be less than 20 lines max, preferably shorter. **Abstractors must make judgments and decisions** about two things: 1) deciding which data to abstract (e.g. when numerous sub-groups are analyzed, decide what is most relevant to our research questions), and 2) drawing summary conclusions. One of the senior authors will review summary conclusions and will discuss with abstracters if the summary statement appears inconsistent with the data.

**Note:** Abstracted data for non-poolable outcomes will *only* be used to support summary conclusions. We will not pool, or otherwise undertake statistical analyses, on data found in narrative summaries.

1. Abstractors will be provided with a list of variables identified in each study during screening. They are to look carefully for data of interest in each strata checked “yes” or “possibly” on the list. If both abstractors agree that: 1) no data can be abstracted for a variable identified during stratification, or 2) data for a variable of interest can be abstracted from the study but was not identified during stratification, we will accept the abstractors’ decisions as “final say” about what should be abstracted.

***Rules for the Non-Poolable Data Analysis***

**Rule #1:** All of the data abstraction rules should be applied when creating the non-poolable data analysis summaries (the “main findings” column of the table).

**Rule #2:** If the percentage of ABF changes during the calendar year, we will use the percent ABF that was present for most of the year. For example, in the US January to September of 1984 was 25% ABF and October to December was 50% ABF. Based on this rule we record 1984 as 25% ABF. We will note this rule in a footnote below the table. The exception to the rule is the year 1983 for US Mediare studies, we will treat this year as before ABF unless the author indicates that data were collected after October 1983, in which case it will be treated as after ABF.

- *0% ABF* – The earliest no ABF data point reported that is within 3 years of ABF implementation. For example, if ABF was implemented in 1983, and data are provided for the years 1979, 1980 1981, and 1982; abstract data from 1980.
- *Earliest ABF* – Abstract data for period immediately after ABF implementation. For example, if ABF is implemented in 1983, and hospital readmission data are provided for 1984, 1985, 1986, and 1987; abstract data for 1984.
- *Latest ABF* – Abstract data for the period furthest away from ABF implementation, up to 5 years after ABF implementation. For example, if ABF was implemented in 1983, and hospital readmission data are reported in 1984, 1985, 1986, 1987, 1988, and 1989; abstract the data reported in 1988.

**Rule #3**: Do not mention subgroups in the narrative summaries unless there is an interaction between the ABF and the subgroup (i.e. the effect of ABF varies across subgroups).

**Rule #4:** We will not include dates in the “main findings” column.

***Specific instructions for completing the data abstraction form***

SECTION I: STUDY DEMOGRAPHICS

| **Study Demographics Question Number** | **Specific Instruction** |
| --- | --- |
| 1. Record how the study was published: | 1. Most likely response= journal article. |
| 2. Record the geographical location(s) of the study (check all that apply): | 1. Select the “aggregate” response option only if data are reported in aggregate for multiple OECD countries or for multiple Europe and Central Asia countries. |
| 3. Record the type of care: | 1. Post-acute care (e.g. nursing home, skilled nursing facility, rehabilitation facility, home health agency, long-term care facility) 2. Acute care – hospital , surgical facility, or equivalent 3. Select “both” if article reports relevant outcome data about both acute and post-acute care |
| 4. Is the study assessing ABF in: | 1. If the study presents relevant data using multiple comparisons, select the study design that is used to report mortality, discharge destination, and/or hospital readmissions as these are the potentially poolable outcome measures. 2. See **Figure 1** for flow chart   Answers:   1. **Before-After study in a single jurisdiction**: 0 % ABF period (before) vs. periods with increasing ABF (after)   - The comparison is focused on *time of implementation* of ABF (e.g. before 1983, after 1983)   1. **Parallel study comparing ABF and non-ABF adopters at the same time period(s)**: Comparison of  2 (or more) jurisdictions - no ABF vs. ABF   **-** The comparison is *in two different sites at the same time period* (e.g. one outcome measure in 2003-2004 in jurisdiction 1 vs. the same outcome measure in 2003-2004 in jurisdiction 2. E.g. refID 8140)  -Some studies may conduct sequential parallel comparisons at different time periods, but note they do not account for time trends in their summary measures. Reviewers must choose the comparison(s) most relevant to our policy-level research questions. (E.g. OECD paper presents data for ABF adopting and non-adopting countries for multiple years, but does not conduct any form of time trend analysis.)   1. **Before-after study in ABF-adopters COUPLED WITH a parallel comparison with non-adopters, reporting repeated measures over time and *an analysis to account for time trends.***   **-**One (or more) jurisdictions which gradually implemented ABF (before-after) compared to one (or more) that did not implement ABF. The article reports data at several time-points and account for time trends in their analysis (e.g. Farrar, Kjerstad – in contrast with OECD).  **-**The *analysis has to account for time trend*, e.g. "difference in difference", "time series", or multivariate regression models accounting for time and jurisdiction (usually with interaction variable between the two). In these complex analyses, the outcome data CANNOT be pooled.  **-**We use the same guidance as for the other designs to judge appropriateness and comprehensiveness of the adjustment. E.g. Farrar reported a difference-in-difference for readmission, which is inappropriately unadjusted. |
| Table 4 a: | **“Year Reporting Period Start”** and **“Year Reporting Period End”** **columns**:   1. Only record year (not month or day). If only one year (e.g. data collected in 1984 only), record “1984” start and “1984” end. 2. If data are not available, leave blank. 3. *Numerical response* |
| Table 4 b: | **“Year Reporting Period Start”** and **“Year Reporting Period End”** **columns**:   1. Only record year. If only one year (e.g. data collected in 1984 only), record “1984” start and “1984” end. 2. If data are not available, leave blank.   **“No ABF” row:**   1. Record data for the jurisdiction(s) with 0% ABF   **“ABF” row:**   1. Record data for the jurisdiction(s) with the highest percentage of ABF 2. *Numerical response* |
| Table 4 c: | **Rule:** The jurisdiction with ABF (intervention group) will be recorded in the “Jurisdiction 1” row and the jurisdiction with no-ABF (control group) will be recorded in the “Jurisdiction 2” row.  **“Year Reporting Period Start”** and **“Year Reporting Period End”** **columns**:   1. Only record year. If only one year (e.g. data collected in 1984 only), record “1984” start and “1984” end. 2. If data are not available, leave blank.   “**Before ABF period”:** the period in which data were collected before the implementation of ABF  **“After ABF period”:** the period in which data were collected before the implementation of ABF  **“No ABF” row:**   1. Record data for the jurisdiction(s) with 0% ABF   **“ABF” row:**   1. Record data for the jurisdiction(s) with the highest percentage of ABF 2. *Numerical response* |
| 5. Record how the authors sampled the institutions: | 1. To check random sample or all eligible institutions, it has to be explicitly stated. If not specified, assume convenience sample. 2. For national registries, check all eligible jurisdictions. 3. If data are from a database but it is not clear if it is national database, select “convenience sample.” 4. If a database is used and it is not clear if it contained all eligible institutions in a jurisdiction, select “convenience sample.” 5. Eligible institutions mean all eligible institutions from which the study population and study question could be derived. |
| 6. Record the nature of the data analyzed | 1. The answer options will be defined as follows:  - **“Primary data collection”:** the author(s) collected data specifically for the current study - **“Secondary analysis of existing data”:** the author(s) did not collect data specifically for the current study and instead used data originally collected for a different purpose (e.g. federal registry, HICFA, data collected for another study). - **“Both”:** the author(s) used both primary and secondary data  1. Base answers to this question solely on data that are collected for our review’s outcomes of interest. E.g. if the study collected data from patients for a quality of life survey (primary data) but obtained mortality information from a pre-existing database (secondary data), select “secondary analysis of existing data” because only mortality is a variable of interest. |
| 7. Record the data source: | 1. Example: HCFA or national registries 2. *Free text response* |
| 8.Record the study population: | 1. Record age, sociodemographic variables, and disease if provided.  - E.g. men and women aged 65 and older - E.g. patients >age 18 with community acquired pneumonia  1. If two different populations are examined in the study, use the population from which the most relevant outcome is derived. 2. *Free text response* |
| 9. Did the study report total number of patients evaluated: | 1. Record “N” for total number of patients (not “n” patients per study group). For example, if there are 500 patients in the “before ABF group” and 500 patients in the “after ABF group” record “1,000”. 2. If the study examines outcomes not of interest as well as outcomes of interest and the number of patients evaluated is different, record the number of patients that pertain to our outcomes of interest. For example, if 1,000 patients were examined to determine length of stay, but only 200 patients were examined to determine mortality, record “200”. 3. *Numerical Response* |
| 10. Did the study report total number of facilities evaluated? | 1. Record “N” for total number of hospitals (not “n” hospitals per study group). E.g. if 100 hospitals in the “before ABF group” and 100 hospitals in the “after ABF group”, record “100”. 2. If the study examines outcomes that are not of interest as well as outcomes of interest and if the number of hospitals evaluated is different, record the number of hospitals for our outcomes of interest. For example, if 200 hospitals were examined to determine length of stay, but only 20 hospitals were examined to determine mortality, record “20”. 3. *Numerical Response* |
| 11. Is this a US Medicare study? | 1. Answer “Yes” if study examines ABF over time (before/after study) within the US Medicare system OR if the study compares ABF in the US Medicare system compared to a non-ABF jurisdiction |
| 12. Record the primary stated reason(s) for implementing ABF in the country under consideration as reported by the author (check all that apply): | 1. Record only the reason the author provides for why the authorities implemented ABF, not what other literature says or what you know to be true. We are only interested in explicit statements about why the government implemented ABF (e.g. The Swiss government implemented ABF to reduce costs and decrease wait times). 2. If the “other” response is selected, copy and paste passages from the study that are relevant to this question into the text box in Distiller. 3. *Free text response* |
| 13. **For-profit versus not-for-profit** - The study explicitly states that it does the following for a variable of interest: | 1. Select the third answer response only if the study reports on data considered as an outcome of interest for this study |
| 14. **Rural settings** - The study explicitly states that it does the following for a variable of interest: | 1. Select the third answer response only if the study reports on data considered as an outcome of interest for this study |
| 15. For any of the following variables of interest (readmission, mortality, discharge destination, volume of care, characteristics of study population, severity of illness/casemix) does the article provide data for any of the following procedures/conditions (check all that apply): | 1. Check all that apply for any of the following variables of interest (readmission, mortality, discharge destination, volume of care, characteristics of study population, severity of illness/casemix) |
| 16.Year ABF started: ________ | 1. If the study provides an approximate year of implementation, assume this is the year ABF was implemented. 2. If ABF was phased in, record the date in which phase-in began. 3. If data are aggregated across multiple ABF jurisdictions (i.e. jurisdictions with different ABF start dates), select “Not specified.” 4. For US Medicare studies only, assume these dates:  - Beginning on or after October 1, 1983 and before October 1, 1984: ABF=25% - Beginning on or after October 1, 1984 and before October 1, 1985: ABF=50% - Beginning on or after October 1, 1985 and before October 1, 1986: ABF=75% - Beginning on or after October 1, 1986: ABF=100%  1. *Numerical Response* |

SECTION V: HOSPITAL READMISSION

| **Readmission Question Number** | **Specific Instruction** | |
| --- | --- | --- |
| 16. Does the study contain hospital readmission data? |  | |
| 17. What is the time period over which hospital readmission is assessed? | If more than one time period are analyzed use the following guidelines:   1. For non-poolable data, abstract both time periods clearly indicating which results are for which periods. 2. For poolable data: 3. If multiple readmission periods each ≤ 30 days, abstract the latest readmission data (e.g. if 30 days, 15 days, and 5 days, abstract 30 days) 4. If multiple readmission periods each > 30 days, abstract the earliest readmission data (e.g. if 45 days and 60 days, abstract 45 days) 5. If multiple readmission periods with some ≤ 30 days and some > 30 days, abstract the readmission data closest to 30 days (e.g. if 14 days, 30 days, and 60 days, abstract 30 days) | |
| 18. Can data from an adjusted analysis for readmission rates be abstracted? | 1. If more than one analysis is presented, abstract data from the adjusted analysis. If more than one adjusted analysis is presented, answer based on the most adjusted analyses. 2. If standardized or weighted outcomes are reported, we will consider this an adjusted analysis and record all variables for which outcomes are standardized/weighted. | |
| 19a. What appropriate factors did the readmission analysis adjust for? (check all that apply): | 1. Select the most appropriate factor from the list for each adjustment variable reported in the article. Only select one adjustment factor from the list for each adjustor in the paper. 2. If the analysis adjusted for a variable not on the list, select other and notify Taryn immediately by email. Include in your email a description of the variable and if the variable is an appropriate or inappropriate adjuster. | |
| 19b. What inappropriate factors did the readmission analysis adjust for? (check all that apply): | 1. Select the most appropriate factor from the list for each adjustment variable reported in the article. Only select one adjustment factor from the list for each adjustor in the paper. 2. If the analysis adjusted for a variable not on the list, select other and notify Taryn immediately by email. Include in your email a description of the variable and if the variable is an appropriate or inappropriate adjuster. | |
| 20. Are the factors adjusted for in the readmission analysis comprehensive and appropriate? | 1. In order to be considered comprehensive, the analysis must adjust for all of the following:   -sex  -age  -at least one measure of index of severity (i.e. severity of principal diagnosis, severity of illness)  - at least one measure of casemix/comorbidities (i.e. comorbidities, DRG, casemix, ICD-9 code, presence of risk factors, patient required secondary procedure, disease, number of diagnoses)   1. If a variable shows clearly different distributions in ABF and non-ABF group, and the authors fail to adjust for that variable, one may rate down comprehensiveness of the adjustment (e.g. more rural hospitals in the non-ABF group and more urban hospitals in a ABF group, requires an adjustment for rural/urban location) | |
| 21. Does the study report data that could be pooled? | 1. See **Figure 2** 2. For all studies included in the review we will look specifically for the presence of upcoding of specific DRGs. In situations where the presence of upcoding is clear and unambiguous for specific DRGs, the data will be ineligible for pooling. If otherwise eligible, these data will be included with the non-poolable results. 3. If data meet poolability criteria but a subgroup analysis was conducted:   1. Abstract total for all sub-groups (i.e. "all ages" row) if provided  2.  If total for all sub-groups is not provided, try to calculate on your own by summing ABF and non ABF groups. If only percentages and denominators are provided, calculate numerators and sum ABF and non-ABF groups.  This is the same concept as we use for dealing with multiple PAC.  3.  If not possible to sum data, consider data to be non-poolable and abstract in the non-poolable outcomes table. | |
| 22. Does the study **REPORT** hospital readmission data in: | 1. See **Figure 1** for flow chart 2. Answers: 3. **Before-After study in a single jurisdiction**: 0 % ABF period (before) vs. periods with increasing ABF (after)   - The comparison is focused on *time of implementation* of ABF (e.g. before 1983, after 1983)   1. **Parallel study comparing ABF and non-ABF adopters at the same time period(s)**: Comparison of  2 (or more) jurisdictions - no ABF vs. ABF   **-** The comparison is *in two different sites at the same time period* (e.g. one outcome measure in 2003-2004 in jurisdiction 1 vs. the same outcome measure in 2003-2004 in jurisdiction 2. E.g. refID 8140)  -Some studies may conduct sequential parallel comparisons at different time periods, but note they do not account for time trends in their summary measures. Reviewers must choose the comparison(s) most relevant to our policy-level research questions. (E.g. OECD paper presents data for ABF adopting and non-adopting countries for multiple years, but does not conduct any form of time trend analysis.)   1. **Before-after study in ABF-adopters COUPLED WITH a parallel comparison with non-adopters, reporting repeated measures over time and an analysis to account for time trends.**   **-**One (or more) jurisdictions which gradually implemented ABF (before-after) compared to one (or more) that did not implement ABF. The article reports data at several time-points and account for time trends in their analysis (e.g. Farrar, Kjerstad – in contrast with OECD).  **-**The *analysis has to account for time trend*, e.g. "difference in difference", "time series", or multivariate regression models accounting for time and jurisdiction (usually with interaction variable between the two). In these complex analyses, the outcome data CANNOT be pooled.  **-**We use the same guidance as for the other designs to judge appropriateness and comprehensiveness of the adjustment. E.g. Farrar reported a difference-in-difference for readmission, which is inappropriately unadjusted. | |
| Table 22a | **“Earliest No ABF data point (within 3 years of ABF implementation) (data point A)” column:**   1. Only record year (not month or day if provided). If only one year (e.g. data collected in 1984 only) record “1984” start and “1984” end. 2. If data are not available, leave blank 3. Example, if ABF was implemented in 1983, and data are provided for the years 1979, 1980 1981, and 1982; abstract data from 1980 and record 1980 as the start and end date. If ABF implementation is in 1983 and one data point is provided for 1981 and 1982 (i.e. data are aggregated), abstract data from 1981-1982 and record 1981 as the start date and 1982 as the end date. 4. *Numerical response*   **“Earliest ABF data point (Data point B)” column:**   1. Only record year (not month or day). If only one year (e.g. data collected in 1984 only) record “1984” start and “1984” end. 2. If data are not available, leave blank. 3. Example, if ABF is implemented in 1983, and hospital readmission data are provided for 1984, 1985, 1986, and 1987; abstract data for 1984 and record 1984 as the start and end date. If ABF implementation is in 1983, and one data point is provided for 1984 and 1985 (i.e. data are aggregated), abstract data from 1984-1985 and record 1984 as the start date and 1985 as the end date. 4. If data are only reported for one time period after ABF, do not complete this column. For example, if ABF was implemented in 1983 and only data for 1984 are reported, skip this row and report data in the “latest ABF point” row. 5. *Numerical response*   **“Latest ABF point (within 5 years of ABF implementation) (Data point C)” column:**   1. Only record year (not month or day if provided). If only one year (e.g. data collected in 1984 only) record “1984” start and “1984” end. 2. If data are not available, leave blank. 3. Example, if ABF was implemented in 1983, and hospital readmission data are reported in 1984, 1985, 1986, 1987, 1988, and 1989; abstract the data reported in 1988 and record 1988 as the start and end date. If ABF was implemented in 1983, and one data point is provided for 1987 and 1988 (i.e. data are aggregated), abstract data from 1987-1988 and record 1987 as the start date and 1988 as the end date. 4. *Numerical response*   **Percent ABF**   1. Enter the ABF percentage for the ABF group 2. If a range is not reported and instead only a single percentage point is reported, complete only the “lower range text field.” 3. If percent not included, leave blank (except for US Medicare studies).   For US Medicare studies only, assume these dates:  Medicare Phase-In Period  1. Beginning on or after October 1, 1983 and before October 1, 1984: ABF=25%  2. Beginning on or after October 1, 1984 and before October 1, 1985: ABF=50%  3. Beginning on or after October 1, 1985 and before October 1, 1986: ABF=75%  4. Beginning on or after October 1, 1986: ABF=100%   1. *Numerical response*   **“Operational Variable” column:**   1. Name the specific variable, the units in which it is measured, and the time period over which it is measured (e.g. number of hospital readmissions within 90 days of discharge). 2. *Free text*   ****Note:** if more than one relevant outcome variable is reported for one of the listed variables, record data for both variables and use numbering to indicate what data goes with variable in the free text boxes (e.g. 1. Age (year); 2. Sex)  **“Summary of results” column:**   1. Abstractors will record key data as well as summary conclusions supported by key data. Summary conclusion should be a single sentence when possible. 2. We are interested in the comparison between data points labelled A vs. B and A vs. C (not B vs. C). 3. If the difference is not significant, still record the actual data numbers (e.g. percentage of readmission 33% in ABF vs. 35% in non-ABF (p=.06)). 4. If uncertain of what to abstract, consult one of the senior team members (likely Karen or Gordon or Danielle, the choice depending on the nature of the uncertainty). 5. Important to ensure that we are reporting data objectively, not inferences made by ourselves or the author(s). 6. When the author(s) provide only a narrative summary or commentary of their interpretation of data but do not provide data on which these inferences are based, do not include in results. 7. Free text   ****Note:** if more than one relevant outcome variable is reported for one of the listed variables, record data for both variables and use numbering to indicate what data goes with variable in the free text boxes (e.g. 1. mean age 66 in ABF vs. 68 in non-ABF (p=.06); 2. 1% more males in the ABF group (p = 0.80)).  **Difference between** **the 0% ABF vs.** **early ABF periods**   1. Answer below table in question 22ai   **Difference between** **the 0% ABF vs.** **later ABF periods**   1. Answer below table in question 22aii | |
| 22a i. **“Difference between the 0% ABF vs. early ABF periods”:** | 1. If a comparison between 0% and early ABF periods was not made select “comparison not made.” 2. If the comparison is reported in the article as not significant, select “no difference.” If the comparison is reported to be significant or no significance test was performed, the reviewer should record the direction of the effect and calculate the percentage change. 3. Use this link to calculate percentage change (enter the no ABF number first and the ABF number second): [http://www.csgnetwork.com/percentchangecalc.html](http://www.csgnetwork.com/percentchangecalc.html) 4. OR, The formula to calculate percent change is as follows: time 2-time 1/time 1 = x (100)= % change between time 1 and time 2   *(time 2= earlier ABF period and/ or later ABF period; time 1= 0% ABF period)  *e.g. if death rate was 2% before ABF, and 4% after ABF, then 4-2/2 = 1 (100)=100% difference  ****Note:** if more than one relevant outcome variable are reported in the above table check all that apply. | |
| 22 a ii. **“Difference between the 0% ABF vs. later ABF periods”:** | 1. If a comparison between 0% and late ABF periods was not made select “comparison not made.” 2. If the comparison is reported in the article as not significant, select “no difference.” If the comparison is reported to be significant or no significance test was performed, the reviewer should record the direction of the effect and calculate the percentage change. 3. Use this link to calculate percentage change (enter the no ABF number first and the ABF number second): [http://www.csgnetwork.com/percentchangecalc.html](http://www.csgnetwork.com/percentchangecalc.html) 4. OR, The formula to calculate percent change is as follows: time 2-time 1/time 1 = x (100)= % change between time 1 and time 2   *(time 2= earlier ABF period and/ or later ABF period; time 1= 0% ABF period)  *e.g. if death rate was 2% before ABF, and 4% after ABF, then 4-2/2 = 1 (100)=100% difference  ****Note:** if more than one relevant outcome variable are reported in the above table check all that apply. | |
| Table 22b | **“Start and End year of data point collection”:**   1. Only record year (not month or day). If only one year (e.g. data collected in 1984 only) record “1984” start and “1984” end. 2. If data are not available leave blank 3. *Numeric response*   **Percent ABF**   1. Enter the ABF percentage for the ABF group 2. If a range is not reported and instead only a single percentage point is reported, complete only the “lower range text field.” 3. If percent not included, leave blank (except for US Medicare studies).   For US Medicare studies only, assume these dates:  Medicare Phase-In Period  1. Beginning on or after October 1, 1983 and before October 1, 1984: ABF=25%  2. Beginning on or after October 1, 1984 and before October 1, 1985: ABF=50%  3. Beginning on or after October 1, 1985 and before October 1, 1986: ABF=75%  4. Beginning on or after October 1, 1986: ABF=100%   1. *Numerical response*   **“Operational Variable” column:**   1. Name the specific variable, the units in which it is measured, and the time period over which it is measured (e.g. number of hospital readmissions within 90 days of discharge). 2. *Free text*   ****Note:** if more than one relevant outcome variable is reported for one of the listed variables, record data for both variables and use numbering to indicate what data goes with variable in the free text boxes (e.g. 1. Age (year); 2. Sex)  **“Summary of results” column:**   1. Abstractors will record key data as well as summary conclusions supported by key data. Summary conclusion should be a single sentence when possible. 2. If the difference is not significant, still record the actual data numbers (e.g. percentage of readmission 33% in ABF vs. 35% in non-ABF (p=.06)). 3. If uncertain of what to abstract, consult one of the senior team members (likely Karen or Gordon or Danielle, the choice depending on the nature of the uncertainty). 4. Important to ensure that we are reporting data objectively, not inferences made by ourselves or the author(s). 5. When the author(s) provide only a narrative summary or commentary of their interpretation of data but do not provide data on which these inferences are based, do not include in results. 6. *Free text*   ****Note:** if more than one relevant outcome variable is reported for one of the listed variables, record data for both variables and use numbering to indicate what data goes with variable in the free text boxes (e.g. 1. mean age 66 in ABF vs. 68 in non-ABF (p=.06); 2. 1% more males in the ABF group (p = 0.80)).  **Difference between the** **0% ABF vs. most ABF** **jurisdiction**   1. Answer below in question 22bi | |
| 22 b i. **“Difference between the 0% ABF vs. most ABF jurisdictions”:** | 1. If a comparison between 0% and most ABF jurisdiction was not made, select “comparison not made.” 2. If the comparison is reported in the article as not significant, select “no difference.” If the comparison is reported to be significant or no significance test was performed, the reviewer should record the direction of the effect and calculate the percentage change. 3. Use this link to calculate percentage change (enter the no ABF number first and the ABF number second): [http://www.csgnetwork.com/percentchangecalc.html](http://www.csgnetwork.com/percentchangecalc.html) 4. OR, The formula to calculate percent change is as follows: time 2-time 1/time 1 = x (100)= % change between time 1 and time 2   *(Where time 2=ABF group and time 1= 0% ABF group)  *e.g. if death rate was 2% in the non-ABF, and 4% in the ABF, then 4-2/2 =1 (100)=100% difference  ****Note:** if more than one relevant outcome variable are reported in the above table check all that apply | |
| Table 22c | **“Earliest No ABF data point (within 3 years of ABF implementation) (data point A)” column:**   1. Only record year (not month or day if provided). If only one year (e.g. data collected in 1984 only) record “1984” start and “1984” end. 2. If data are not available, leave blank 3. Example, if ABF was implemented in 1983, and data are provided for the years 1979, 1980 1981, and 1982; abstract data from 1980 and record 1980 as the start and end date. If ABF implementation is in 1983 and one data point is provided for 1981 and 1982 (i.e. data are aggregated), abstract data from 1981-1982 and record 1981 as the start date and 1982 as the end date. 4. *Numerical response*   **“Earliest ABF data point (Data point B)” column:**   1. Only record year (not month or day). If only one year (e.g. data collected in 1984 only) record “1984” start and “1984” end. 2. If data are not available, leave blank. 3. Example, if ABF is implemented in 1983, and hospital readmission data are provided for 1984, 1985, 1986, and 1987; abstract data for 1984 and record 1984 as the start and end date. If ABF implementation is in 1983, and one data point is provided for 1984 and 1985 (i.e. data are aggregated), abstract data from 1984-1985 and record 1984 as the start date and 1985 as the end date. 4. If data are only reported for one time period after ABF, do not complete this column. For example, if ABF was implemented in 1983 and only data for 1984 are reported, skip this row and report data in the “latest ABF point” row. 5. *Numerical response*   **“Latest ABF point (within 5 years of ABF implementation) (Data point C)” column:**   1. Only record year (not month or day if provided). If only one year (e.g. data collected in 1984 only) record “1984” start and “1984” end. 2. If data are not available, leave blank. 3. Example, if ABF was implemented in 1983, and hospital readmission data are reported in 1984, 1985, 1986, 1987, 1988, and 1989; abstract the data reported in 1988 and record 1988 as the start and end date. If ABF was implemented in 1983, and one data point is provided for 1987 and 1988 (i.e. data are aggregated), abstract data from 1987-1988 and record 1987 as the start date and 1988 as the end date. 4. *Numerical response*   **Percent ABF**   1. Enter the ABF percentage for the ABF group 2. If a range is not reported and instead only a single percentage point is reported, complete only the “lower range text field.” 3. If percent not included, leave blank (except for US Medicare studies).   For US Medicare studies only, assume these dates:  Medicare Phase-In Period  1. Beginning on or after October 1, 1983 and before October 1, 1984: ABF=25%  2. Beginning on or after October 1, 1984 and before October 1, 1985: ABF=50%  3. Beginning on or after October 1, 1985 and before October 1, 1986: ABF=75%  4. Beginning on or after October 1, 1986: ABF=100%   1. *Numerical response*   **“Operational Variable” column:**   1. Name the specific variable, the units in which it is measured, and the time period over which it is measured (e.g. number of hospital readmissions within 90 days of discharge). 2. *Free text*   ****Note:** if more than one relevant outcome variable is reported for one of the listed variables, record data for both variables and use numbering to indicate what data goes with variable in the free text boxes (e.g. 1. Age (year); 2. Sex)  **“Summary of results” column:**   1. Abstractors will record key data as well as summary conclusions supported by key data. Summary conclusion should be a single sentence when possible. 2. We are interested in the comparison between data points labelled A vs. B and A vs. C (not B vs. C) in the ABF vs. no ABF jurisdictions. 3. If the difference is not significant, still record the actual data numbers (e.g. percentage of readmission 33% in ABF vs. 35% in non-ABF (p=.06)). 4. If uncertain of what to abstract, consult one of the senior team members (likely Karen or Gordon or Danielle, the choice depending on the nature of the uncertainty). 5. Important to ensure that we are reporting data objectively, not inferences made by ourselves or the author(s). 6. When the author(s) provide only a narrative summary or commentary of their interpretation of data but do not provide data on which these inferences are based, do not include in results. 7. *Free text*   ****Note:** if more than one relevant outcome variable is reported for one of the listed variables, record data for both variables and use numbering to indicate what data goes with variable in the free text boxes (e.g. 1. mean age 66 in ABF vs. 68 in non-ABF (p=.06); 2. 1% more males in the ABF group (p = 0.80)).    **Difference between** **A vs. B** **in no ABF** **jurisdiction  VS** **A vs B in ABF** **jurisdiction**   1. Answer below in question 22ci   **Difference between** **A vs. C** **in no ABF** **jurisdiction VS** **A vs C in ABF** **jurisdiction**   1. Answer below in question 22cii | |
| 22 c i. Difference between A vs B in no ABF jurisdiction VS A vs B in ABF jurisdiction: | 1. If a comparison between 0% and early ABF periods was not made in the ABF and no ABF jurisdictions select “comparison not made” 2. If the comparison is reported in the article as not significant, select “no difference”. If the comparison is reported to be significant, or no significance test was performed, the reviewer should record the direction of the effect and calculate the percentage change. 3. Use this link to calculate percentage change (enter the no ABF number first and the ABF number second): [http://www.csgnetwork.com/percentchangecalc.html](http://www.csgnetwork.com/percentchangecalc.html) 4. The formula is as follows:   time 2-time 1 in the 0% ABF periods = A  time 2 - time 1 in the ABF periods = B  then formula is B - A/A =x (100)  ****Note:** if more than one relevant outcome variable are reported in the above table check all that apply | |
| 22cii. Difference between A vs C in no ABF jurisdiction VS A vs C in ABF jurisdiction: | 1. If a comparison between 0% and later ABF periods was not made in the ABF and no ABF jurisdictions select “comparison not made” 2. If the comparison is reported in the article as not significant, select “no difference”. If the comparison is reported to be significant, or no significance test was performed, the reviewer should record the direction of the effect and calculate the percentage change. 3. Use this link to calculate percentage change (enter the no ABF number first and the ABF number second): [http://www.csgnetwork.com/percentchangecalc.html](http://www.csgnetwork.com/percentchangecalc.html) 4. The formula is as follows:   time 2-time 1 in the 0% ABF periods = A  time 2 - time 1 in the ABF periods = B  then formula is B - A/A =x (100)  ****Note:** if more than one relevant outcome variable are reported in the above table check all that apply | |
| 23. Does the study **REPORT** hospital readmission data in | 1. See **Figure 1** for flow chart 2. Answers: 3. **Before-After study in a single jurisdiction**: 0 % ABF period (before) vs. periods with increasing ABF (after)   - The comparison is focused on *time of implementation* of ABF (e.g. before 1983, after 1983)   1. **Parallel study comparing ABF and non-ABF adopters at the same time period(s)**: Comparison of  2 (or more) jurisdictions - no ABF vs. ABF   **-** The comparison is *in two different sites at the same time period* (e.g. one outcome measure in 2003-2004 in jurisdiction 1 vs. the same outcome measure in 2003-2004 in jurisdiction 2. E.g. refID 8140)  -Some studies may conduct sequential parallel comparisons at different time periods, but note they do not account for time trends in their summary measures. Reviewers must choose the comparison(s) most relevant to our policy-level research questions. (E.g. OECD paper presents data for ABF adopting and non-adopting countries for multiple years, but does not conduct any form of time trend analysis.) | |
| Table 23a | **“Earliest No ABF data point (within 3 years of ABF implementation) (data point A)” column:**   1. Only record year (not month or day if provided). If only one year (e.g. data collected in 1984 only) record “1984” start and “1984” end. 2. If data are not available, leave blank 3. Example, if ABF was implemented in 1983, and data are provided for the years 1979, 1980 1981, and 1982; abstract data from 1980 and record 1980 as the start and end date. If ABF implementation is in 1983 and one data point is provided for 1981 and 1982 (i.e. data are aggregated), abstract data from 1981-1982 and record 1981 as the start date and 1982 as the end date. 4. *Numerical response*   **“Earliest ABF data point (Data point B)” column:**   1. Only record year (not month or day). If only one year (e.g. data collected in 1984 only) record “1984” start and “1984” end. 2. If data are not available, leave blank. 3. Example, if ABF is implemented in 1983, and hospital readmission data are provided for 1984, 1985, 1986, and 1987; abstract data for 1984 and record 1984 as the start and end date. If ABF implementation is in 1983, and one data point is provided for 1984 and 1985 (i.e. data are aggregated), abstract data from 1984-1985 and record 1984 as the start date and 1985 as the end date. 4. If data are only reported for one time period after ABF, do not complete this column. For example, if ABF was implemented in 1983 and only data for 1984 are reported, skip this row and report data in the “latest ABF point” row. 5. *Numerical response*   **“Latest ABF point (within 5 years of ABF implementation) (Data point C)” column:**   1. Only record year (not month or day if provided). If only one year (e.g. data collected in 1984 only) record “1984” start and “1984” end. 2. If data are not available, leave blank. 3. Example, if ABF was implemented in 1983, and hospital readmission data are reported in 1984, 1985, 1986, 1987, 1988, and 1989; abstract the data reported in 1988 and record 1988 as the start and end date. If ABF was implemented in 1983, and one data point is provided for 1987 and 1988 (i.e. data are aggregated), abstract data from 1987-1988 and record 1987 as the start date and 1988 as the end date. 4. *Numerical response*   **Percent ABF**   1. Enter the ABF percentage for the ABF group 2. If a range is not reported and instead only a single percentage point is reported, complete only the “lower range text field.” 3. If percent not included, leave blank (except for US Medicare studies).   For US Medicare studies only, assume these dates:  Medicare Phase-In Period  1. Beginning on or after October 1, 1983 and before October 1, 1984: ABF=25%  2. Beginning on or after October 1, 1984 and before October 1, 1985: ABF=50%  3. Beginning on or after October 1, 1985 and before October 1, 1986: ABF=75%  4. Beginning on or after October 1, 1986: ABF=100%   1. *Numerical response*   **“# of events per group” column:**   1. Record the number of events for the “No ABF”, “Earliest ABF” and “Late ABF” groups in the corresponding columns 2. This is not applicable for the “Differences” rows (row 6 and 7) 3. *Numeric response*   **“Sample size of groups” column:**   1. Record the sample size for the “No ABF”, “Earliest ABF” and “Late ABF” groups in the corresponding columns 2. *Numeric response*   **“Total sample size” column:**   1. Record the total sample size (i.e. sum of sample size of all groups reported in the paper). For example, if data are collected from 1,000 patients in 1980, 1981, 1982, 1983, and 1984, then total sample size = 5,000 (1000 x 5 groups) 2. *Numeric response*   “**Name measure of central tendency” column:**   1. Check all measures that apply and record the applicable number in the *free text* box. Include relevant positive or negative sign and decimals if applicable**.**   **“Name measure of spread or equivalent” column:**   1. Check all measures that apply and record the applicable number in the *free text* box. Include relevant positive or negative sign and decimals if applicable**.** 2. Record ranges (e.g. confidence intervals) with a hyphen without spaces (e.g. 0.9-3.2)   **Abstracting Poolable data:**  In general, we need to abstract are as follows for each outcome of interest:   1. The estimate of effect for variable of interest (impact of ABF on an outcome) 2. Its measure of spread (SD, Variance)   🡪 If measure of spread is absent, report   - 1. Standard Error (SE) OR Confidence Interval (CI)   OR statistic (t, z, F…),  OR exact p-value (not possible from approximate p-value, e.g. <0.01)   - 1. AND the numbers in each group (N1, N2) OR, at worst, sample size (N)   No need to abstract R2 for the model, or coefficients for the other variables adjusted in the model.  **To pool specific statistics we require:** | |
|  | Rate Rate change  …per 100.000 patient days | 1. Estimate (rate per patient-days) 2. Measure of spread (SD)   OR   - 1. SE, CI, Statistic, exact p value  1. Numbers (n1,n2 OR n) |
|  | OR  RR  HR | 1. Estimate (OR, RR, HR) 2. Measure of spread (SD)   OR   1. SE, CI, Statistic, exact p-value 2. Numbers (n1,n2 OR n) |
|  | Percent change | 1. Estimate (%) 2. Measure of spread (SD)   OR   1. SE, CI, Statistic, exact p-value 2. Numbers (n1,n2 OR n) |
|  | Raw number (n) | 1. Estimate n1, n2 2. Denominator N1 N2 |
|  | Regression coefficients  (from a  Logistic  or  Cox survival model) | 1. Estimate = coefficient Beta assessing the impact of ABF   🡪 ABF variable in a parallel study.  🡪 Time in before-after studies but time can be reported in different ways:   - - Continuous   - Dichotomous   - Categorical   - Different dummy variables  1. Measure of spread (SD)   OR   1. SE, CI, Statistic, exact p-value 2. Sample (n) and/or (n1,n2) |
| 23 a ii. Difference between the 0% ABF vs. early ABF periods: | 1. If a comparison between 0% and early ABF periods was not made select “comparison not made” 2. If the comparison is reported in the article as not significant, select “no difference”. If the comparison is reported to be significant, or no significance test was performed, the reviewer should record the direction of the effect and calculate the percentage change. 3. Use this link to calculate percentage change (enter the no ABF number first and the ABF number second): [http://www.csgnetwork.com/percentchangecalc.html](http://www.csgnetwork.com/percentchangecalc.html) 4. The formula is as follows: time 2-time 1/time 1 =x (100)= % difference between time 1 and time 2   *(time 2= earlier ABF period and/ or later ABF period; time 1= 0% ABF period)  *e.g. if death rate was 2% before ABF, and 4% after ABF, then 4-2/2 =1 (100)=100% difference | |
| 23 a iii. Difference between the 0% ABF vs. late ABF periods: | 1. If a comparison between 0% and late ABF periods was not made select “comparison not made” 2. If the comparison is reported in the article as not significant, select “no difference”. If the comparison is reported to be significant, or no significance test was performed, the reviewer should record the direction of the effect and calculate the percentage change. 3. Use this link to calculate percentage change (enter the no ABF number first and the ABF number second): [http://www.csgnetwork.com/percentchangecalc.html](http://www.csgnetwork.com/percentchangecalc.html) 4. The formula is as follows: time 2-time 1/time 1 =x (100)= % difference between time 1 and time 2   *(time 2= earlier ABF period and/ or later ABF period; time 1= 0% ABF period)  *e.g. if death rate was 2% before ABF, and 4% after ABF, then 4-2/2 =1 (100)=100% difference | |
| Table 23b | **“Start and End year of data point collection”:**   1. Only record year (not month or day). If only one year (e.g. data collected in 1984 only) record “1984” start and “1984” end. 2. If data are not available leave blank 3. *Numeric response*   **Percent ABF**   1. Enter the ABF percentage for the ABF group 2. If a range is not reported and instead only a single percentage point is reported, complete only the “lower range text field.” 3. If percent not included, leave blank (except for US Medicare studies).   For US Medicare studies only, assume these dates:  Medicare Phase-In Period  1. Beginning on or after October 1, 1983 and before October 1, 1984: ABF=25%  2. Beginning on or after October 1, 1984 and before October 1, 1985: ABF=50%  3. Beginning on or after October 1, 1985 and before October 1, 1986: ABF=75%  4. Beginning on or after October 1, 1986: ABF=100%   1. *Numerical response*   ****Note:** If you are completing multiple rows of this table you only need to record “start and end years of data point collection” and “percent ABF” columns for the first row as the answers in subsequent rows of the table will be the same. This information should be recorded on at least one row each time you begin a new table.  **“# of events per group” column:**   1. Record the number of events for the “No ABF”, “Most ABF” groups in the corresponding columns 2. This is not applicable for the “Differences between ABF and 0% ABF jurisdictions” (row5) 3. *Numeric response*   **“Sample size of groups” column:**   1. Record the sample size the “No ABF”, “Most ABF” groups in the corresponding columns 2. *Numeric response*   **“Total sample size” column:**   1. Record the total sample size (i.e. sum of sample size of all groups reported in the paper). For example, if data are collected from 1,000 patients in the USA and 1,000 patients in Canada, total sample size = 2,000 2. *Numeric response*   “**Name measure of central tendency” column:**   1. Check all measures that apply and record the applicable number in the *free text* box. Include relevant positive or negative sign and decimals if applicable**.**   **“Name measure of spread or equivalent” column:**   1. Check all measures that apply and record the applicable number in the *free text* box. Include relevant positive or negative sign and decimals if applicable**.** 2. Record ranges (e.g. confidence intervals) with a hyphen without spaces (e.g. 0.9-3.2)   **Abstracting Poolable data:**  In general, we need to abstract as follows for each outcome of interest:   1. the estimate of effect for variable of interest (impact of ABF on an outcome) 2. its measure of spread (SD, Variance)   🡪 If measure of spread is absent, report   - 1. Standard Error (SE) OR Confidence Interval (CI)   OR statistic (t, z, F…),  OR exact p-value (not possible from approximate p-value, e.g. <0.01)   - 1. AND the numbers in each group (N1, N2) OR, at worst, sample size (N)   No need to abstract R2 for the model, or coefficients for the other variables adjusted in the model.  **To pool specific statistics we require:** | |
|  | Rate Rate change  …per 100.000 patient days | 1. Estimate (rate per patient-days) 2. Measure of spread (SD)   OR   1. SE, CI, Statistic, exact p value 2. Numbers (n1,n2 OR n) |
|  | OR  RR  HR | 1. Estimate (OR, RR, HR) 2. Measure of spread (SD)   OR   1. SE, CI, Statistic, exact p-value 2. Numbers (n1,n2 OR n) |
|  | Percent change | 1. Estimate (%) 2. Measure of spread (SD)   OR   1. SE, CI, Statistic, exact p-value 2. Numbers (n1,n2 OR n) |
|  | Raw number (n) | 1. Estimate n1, n2 2. Denominator N1 N2 |
|  | Regression coefficients  (from a  Logistic  or  Cox survival model) | 1. Estimate = coefficient Beta assessing the impact of ABF   🡪 ABF variable in a parallel study.  🡪 Time in before-after studies but time can be reported in different ways:   - - Continuous   - Dichotomous   - Categorical   - Different dummy variables  1. Measure of spread (SD)   OR   1. SE, CI, Statistic, exact p-value 2. Sample (n) and/or (n1,n2) |
| 23 b ii. Difference between the 0% ABF vs. most ABF jurisdictions: | 1. If the comparison is reported in the article as not significant, select “no difference”. If the comparison is reported to be significant, or no significance test was performed, the reviewer should record the direction of the effect and calculate the percentage change. 2. Use this link to calculate percentage change (enter the no ABF number first and the ABF number second): [http://www.csgnetwork.com/percentchangecalc.html](http://www.csgnetwork.com/percentchangecalc.html) 3. The formula is as follows: time 2-time 1/time 1 =x(100)= % change between time 1 and time 2   *(Where time 2=ABF group and time 1= 0% ABF group)  *e.g. if death rate was 2% in the non-ABF, and 4% in the ABF, then 4-2/2 =1 (100)=100% difference | |

SECTION III: MORTALITY DATA SECTION

| **Mortality Question Number** | **Specific Instruction** | |
| --- | --- | --- |
| 24. Does the study contain eligible mortality data? | 1. Eligible mortality rate refers to death rate per population per common time period (e.g. 30 day mortality rate) 2. If the period of time measured starts from “discharge from post-acute care”, it is ineligible. We will include all other events from which the period of time is measured (e.g. admission to acute care, post-surgery, admission to post-acute care). 3. We will exclude studies in which the mortality data are population-based. To be eligible mortality must be reported for an acute or post-acute care institution(s), and cannot be population-based. 4. When mortality data are reported for **patients in a post-acute care institution(s)**, the data will only be eligible for inclusion if it is clear that patients were recently in acute care prior to post-acute care, and are the same patients as those for whom death is reported in post-acute care. For example, we will not include studies if the data are analyzed in such a way that patients could have been in acute care before ABF began (and therefore not exposed to ABF) but subsequently died in post acute care after ABF began in acute care. This will happen if patients died during a post acute care year (say, 1984) but for all we know they were admitted to post acute care in 1980. This will be particularly relevant for nursing homes. 5. In-hospital mortality is ineligible. | |
| 25. What is the time period over which eligible mortality is assessed? | 1. If post discharge mortality is assessed over multiple time periods, we will only abstract data for 1 time period. The hierarchy for selecting which time period to abstract is as follow: (1) 90 day; (2) 60 day; (3) 30 day; (4) other time period (if multiple other periods abstract from the longest time period) | |
| 26. Can data from an adjusted analysis for mortality be abstracted? | 1. If more than one analysis is presented, abstract data from the adjusted analysis. If more than one adjusted analysis is presented, answer based on the most adjusted analyses. 2. If standardized or weighted outcomes are reported, we will consider this an adjusted analysis and record all variables for which outcomes are standardized/weighted. | |
| 27a. What appropriate factors did the mortality analysis adjust for? (check all that apply): | 1. Select the most appropriate factor from the list for each adjustment variable reported in the article. Only select one adjustment factor from the list for each adjustor in the paper. 2. If the analysis adjusted for a variable not on the list, select other and notify Taryn immediately by email. Include in your email a description of the variable and if the variable is an appropriate or inappropriate adjuster | |
| 27b. What inappropriate factors did the mortality analysis adjust for? (check all that apply): | 1. Select the most appropriate factor from the list for each adjustment variable reported in the article. Only select one adjustment factor from the list for each adjustor in the paper. 2. If the analysis adjusted for a variable not on the list, select other and notify Taryn immediately by email. Include in your email a description of the variable and if the variable is an appropriate or inappropriate adjuster | |
| 28. Are the factors adjusted for in the mortality analysis comprehensive and appropriate? | 1. In order to be considered comprehensive, the analysis must adjust for all of the following:   -sex  -age  -at least one measure of index of severity (i.e. severity of principal diagnosis, severity of illness)  - at least one measure of casemix/comorbidities (i.e. comorbidities, DRG, casemix, ICD-9 code, presence of risk factors, disease, number of diagnoses)   1. If a variable shows clearly different distributions in ABF and non-ABF group, and the authors fail to adjust for that variable, one may rate down comprehensiveness of the adjustment (e.g. More rural hospitals in the non-ABF group and more urban hospitals in a ABF group, requires an adjustment for rural/urban location) | |
| 29. Does the study report eligible mortality data that could be pooled | 1. See **Figure 2** 2. For all studies included in the review we will look specifically for the presence of upcoding of specific DRGs. In situations where the presence of upcoding is clear and unambiguous for specific DRGs, the data will be ineligible for pooling. If otherwise eligible these data will be included with the non-poolable results. 3. If data meet poolability criteria but a subgroup analysis was conducted:   1. Abstract total for all sub-groups (i.e. "all ages" row) if provided  2.  If total for all sub-groups is not provided, try to calculate on your own by summing ABF and non ABF groups. If only percentages and denominators are provided, calculate numerators and sum ABF and non-ABF groups.  This is the same concept as we use for dealing with multiple PAC.  3.  If not possible to sum data, consider data to be non-poolable and abstract in the non-poolable outcomes table. | |
| 30. Does the study **REPORT** eligible mortality data in: | 1. See **Figure 1** for flow chart 2. Answers: 3. **Before-After study in a single jurisdiction**: 0 % ABF period (before) vs. periods with increasing ABF (after)   - The comparison is focused on *time of implementation* of ABF (e.g. before 1983, after 1983)   1. **Parallel study comparing ABF and non-ABF adopters at the same time period(s)**: Comparison of  2 (or more) jurisdictions - no ABF vs. ABF   **-** The comparison is *in two different sites at the same time period* (e.g. one outcome measure in 2003-2004 in jurisdiction 1 vs. the same outcome measure in 2003-2004 in jurisdiction 2. E.g. refID 8140)  -Some studies may conduct sequential parallel comparisons at different time periods, but note they do not account for time trends in their summary measures. Reviewers must choose the comparison(s) most relevant to our policy-level research questions. (E.g. OECD paper presents data for ABF adopting and non-adopting countries for multiple years, but does not conduct any form of time trend analysis.)   1. **Before-after study in ABF-adopters COUPLED WITH a parallel comparison with non-adopters, reporting repeated measures over time and an analysis to account for time trends.**   **-**One (or more) jurisdictions which gradually implemented ABF (before-after) compared to one (or more) that did not implement ABF. The article reports data at several time-points and account for time trends in their analysis (e.g. Farrar, Kjerstad – in contrast with OECD).  **-**The *analysis has to account for time trend*, e.g. "difference in difference", "time series", or multivariate regression models accounting for time and jurisdiction (usually with interaction variable between the two). In these complex analyses, the outcome data CANNOT be pooled.  **-**We use the same guidance as for the other designs to judge appropriateness and comprehensiveness of the adjustment. E.g. Farrar reported a difference-in-difference for readmission, which is inappropriately unadjusted. | |
| Table 30a | **“Earliest No ABF data point (within 3 years of ABF implementation) (data point A)” column:**   1. Only record year (not month or day if provided). If only one year (e.g. data collected in 1984 only) record “1984” start and “1984” end. 2. If data are not available, leave blank 3. Example, if ABF was implemented in 1983, and data are provided for the years 1979, 1980 1981, and 1982; abstract data from 1980 and record 1980 as the start and end date. If ABF implementation is in 1983 and one data point is provided for 1981 and 1982 (i.e. data are aggregated), abstract data from 1981-1982 and record 1981 as the start date and 1982 as the end date. 4. *Numerical response*   **“Earliest ABF data point (Data point B)” column:**   1. Only record year (not month or day). If only one year (e.g. data collected in 1984 only) record “1984” start and “1984” end. 2. If data are not available, leave blank. 3. Example, if ABF is implemented in 1983, and hospital readmission data are provided for 1984, 1985, 1986, and 1987; abstract data for 1984 and record 1984 as the start and end date. If ABF implementation is in 1983, and one data point is provided for 1984 and 1985 (i.e. data are aggregated), abstract data from 1984-1985 and record 1984 as the start date and 1985 as the end date. 4. If data are only reported for one time period after ABF, do not complete this column. For example, if ABF was implemented in 1983 and only data for 1984 are reported, skip this row and report data in the “latest ABF point” row. 5. *Numerical response*   **“Latest ABF point (within 5 years of ABF implementation) (Data point C)” column:**   1. Only record year (not month or day if provided). If only one year (e.g. data collected in 1984 only) record “1984” start and “1984” end. 2. If data are not available, leave blank. 3. Example, if ABF was implemented in 1983, and hospital readmission data are reported in 1984, 1985, 1986, 1987, 1988, and 1989; abstract the data reported in 1988 and record 1988 as the start and end date. If ABF was implemented in 1983, and one data point is provided for 1987 and 1988 (i.e. data are aggregated), abstract data from 1987-1988 and record 1987 as the start date and 1988 as the end date. 4. *Numerical response*   **Percent ABF**   1. Enter the ABF percentage for the ABF group 2. If a range is not reported and instead only a single percentage point is reported, complete only the “lower range text field.” 3. If percent not included, leave blank (except for US Medicare studies).   For US Medicare studies only, assume these dates:  Medicare Phase-In Period  1. Beginning on or after October 1, 1983 and before October 1, 1984: ABF=25%  2. Beginning on or after October 1, 1984 and before October 1, 1985: ABF=50%  3. Beginning on or after October 1, 1985 and before October 1, 1986: ABF=75%  4. Beginning on or after October 1, 1986: ABF=100%   1. *Numerical response*   **“Operational Variable” column:**   1. Name the specific variable, the units in which it is measured, and the time period over which it is measured (e.g. number of hospital readmissions within 90 days of discharge). 2. *Free text*   ****Note:** if more than one relevant outcome variable is reported for one of the listed variables, record data for both variables and use numbering to indicate what data goes with variable in the free text boxes (e.g. 1. Age (year); 2. Sex)  **“Summary of results” column:**   1. Abstractors will record key data as well as summary conclusions supported by key data. Summary conclusion should be a single sentence when possible. 2. We are interested in the comparison between data points labelled A vs. B and A vs. C (not B vs. C). 3. If the difference is not significant, still record the actual data numbers (e.g. percentage of readmission 33% in ABF vs. 35% in non-ABF (p=.06)). 4. If uncertain of what to abstract, consult one of the senior team members (likely Karen or Gordon or Danielle, the choice depending on the nature of the uncertainty). 5. Important to ensure that we are reporting data objectively, not inferences made by ourselves or the author(s). 6. When the author(s) provide only a narrative summary or commentary of their interpretation of data but do not provide data on which these inferences are based, do not include in results. 7. *Free text*   ****Note:** if more than one relevant outcome variable is reported for one of the listed variables, record data for both variables and use numbering to indicate what data goes with variable in the free text boxes (e.g. 1. mean age 66 in ABF vs. 68 in non-ABF (p=.06); 2. 1% more males in the ABF group (p = 0.80)).  **Difference between** **the 0% ABF vs.** **early ABF periods**   1. Answer below table in question 30ai   **Difference between** **the 0% ABF vs.** **later ABF periods**   1. Answer below table in question 30aii | |
| **30ai. “Difference between the 0% ABF vs. early ABF periods”:** | 1. If a comparison between 0% and early ABF periods was not made select “comparison not made.” 2. If the comparison is reported in the article as not significant, select “no difference.” If the comparison is reported to be significant or no significance test was performed, the reviewer should record the direction of the effect and calculate the percentage change. 3. Use this link to calculate percentage change (enter the no ABF number first and the ABF number second): [http://www.csgnetwork.com/percentchangecalc.html](http://www.csgnetwork.com/percentchangecalc.html) 4. OR, The formula to calculate percent change is as follows: time 2-time 1/time 1 = x (100)= % change between time 1 and time 2   *(time 2= earlier ABF period and/ or later ABF period; time 1= 0% ABF period)  *e.g. if death rate was 2% before ABF, and 4% after ABF, then 4-2/2 = 1 (100)=100% difference  ****Note:** if more than one relevant outcome variable are reported in the above table check all that apply. | |
| **30aii. “Difference between the 0% ABF vs. later ABF periods”:** | 1. If a comparison between 0% and late ABF periods was not made select “comparison not made.” 2. If the comparison is reported in the article as not significant, select “no difference.” If the comparison is reported to be significant or no significance test was performed, the reviewer should record the direction of the effect and calculate the percentage change. 3. Use this link to calculate percentage change (enter the no ABF number first and the ABF number second): [http://www.csgnetwork.com/percentchangecalc.html](http://www.csgnetwork.com/percentchangecalc.html) 4. OR, The formula to calculate percent change is as follows: time 2-time 1/time 1 = x (100)= % change between time 1 and time 2   *(time 2= earlier ABF period and/ or later ABF period; time 1= 0% ABF period)  *e.g. if death rate was 2% before ABF, and 4% after ABF, then 4-2/2 = 1 (100)=100% difference  ****Note:** if more than one relevant outcome variable are reported in the above table check all that apply. | |
| Table 30b | **“Start and End year of data point collection”:**   1. Only record year (not month or day). If only one year (e.g. data collected in 1984 only) record “1984” start and “1984” end. 2. If data are not available leave blank 3. *Numeric response*   **Percent ABF**   1. Enter the ABF percentage for the ABF group 2. If a range is not reported and instead only a single percentage point is reported, complete only the “lower range text field.” 3. If percent not included, leave blank (except for US Medicare studies).   For US Medicare studies only, assume these dates:  Medicare Phase-In Period  1. Beginning on or after October 1, 1983 and before October 1, 1984: ABF=25%  2. Beginning on or after October 1, 1984 and before October 1, 1985: ABF=50%  3. Beginning on or after October 1, 1985 and before October 1, 1986: ABF=75%  4. Beginning on or after October 1, 1986: ABF=100%   1. *Numerical response*   **“Operational Variable” column:**   1. Name the specific variable, the units in which it is measured, and the time period over which it is measured (e.g. number of hospital readmissions within 90 days of discharge). 2. *Free text*   ****Note:** if more than one relevant outcome variable is reported for one of the listed variables, record data for both variables and use numbering to indicate what data goes with variable in the free text boxes (e.g. 1. Age (year); 2. Sex)  **“Summary of results” column:**   1. Abstractors will record key data as well as summary conclusions supported by key data. Summary conclusion should be a single sentence when possible. 2. If the difference is not significant, still record the actual data numbers (e.g. percentage of readmission 33% in ABF vs. 35% in non-ABF (p=.06)). 3. If uncertain of what to abstract, consult one of the senior team members (likely Karen or Gordon or Danielle, the choice depending on the nature of the uncertainty). 4. Important to ensure that we are reporting data objectively, not inferences made by ourselves or the author(s). 5. When the author(s) provide only a narrative summary or commentary of their interpretation of data but do not provide data on which these inferences are based, do not include in results. 6. *Free text*   ****Note:** if more than one relevant outcome variable is reported for one of the listed variables, record data for both variables and use numbering to indicate what data goes with variable in the free text boxes (e.g. 1. mean age 66 in ABF vs. 68 in non-ABF (p=.06); 2. 1% more males in the ABF group (p = 0.80)).  **Difference between the** **0% ABF vs. most ABF** **jurisdiction**   1. Answer below in question 30bi | |
| 30 b i. **“Difference between the 0% ABF vs. most ABF jurisdictions”:** | 1. If a comparison between 0% and most ABF jurisdiction was not made, select “comparison not made.” 2. If the comparison is reported in the article as not significant, select “no difference.” If the comparison is reported to be significant or no significance test was performed, the reviewer should record the direction of the effect and calculate the percentage change. 3. Use this link to calculate percentage change (enter the no ABF number first and the ABF number second): [http://www.csgnetwork.com/percentchangecalc.html](http://www.csgnetwork.com/percentchangecalc.html) 4. OR, The formula to calculate percent change is as follows: time 2-time 1/time 1 = x (100)= % change between time 1 and time 2   *(Where time 2=ABF group and time 1= 0% ABF group)  *e.g. if death rate was 2% in the non-ABF, and 4% in the ABF, then 4-2/2 =1 (100)=100% difference  ****Note:** if more than one relevant outcome variable are reported in the above table check all that apply | |
| Table 30c | **“Earliest No ABF data point (within 3 years of ABF implementation) (data point A)” column:**   1. Only record year (not month or day if provided). If only one year (e.g. data collected in 1984 only) record “1984” start and “1984” end. 2. If data are not available, leave blank 3. Example, if ABF was implemented in 1983, and data are provided for the years 1979, 1980 1981, and 1982; abstract data from 1980 and record 1980 as the start and end date. If ABF implementation is in 1983 and one data point is provided for 1981 and 1982 (i.e. data are aggregated), abstract data from 1981-1982 and record 1981 as the start date and 1982 as the end date. 4. *Numerical response*   **“Earliest ABF data point (Data point B)” column:**   1. Only record year (not month or day). If only one year (e.g. data collected in 1984 only) record “1984” start and “1984” end. 2. If data are not available, leave blank. 3. Example, if ABF is implemented in 1983, and hospital readmission data are provided for 1984, 1985, 1986, and 1987; abstract data for 1984 and record 1984 as the start and end date. If ABF implementation is in 1983, and one data point is provided for 1984 and 1985 (i.e. data are aggregated), abstract data from 1984-1985 and record 1984 as the start date and 1985 as the end date. 4. If data are only reported for one time period after ABF, do not complete this column. For example, if ABF was implemented in 1983 and only data for 1984 are reported, skip this row and report data in the “latest ABF point” row. 5. *Numerical response*   **“Latest ABF point (within 5 years of ABF implementation) (Data point C)” column:**   1. Only record year (not month or day if provided). If only one year (e.g. data collected in 1984 only) record “1984” start and “1984” end. 2. If data are not available, leave blank. 3. Example, if ABF was implemented in 1983, and hospital readmission data are reported in 1984, 1985, 1986, 1987, 1988, and 1989; abstract the data reported in 1988 and record 1988 as the start and end date. If ABF was implemented in 1983, and one data point is provided for 1987 and 1988 (i.e. data are aggregated), abstract data from 1987-1988 and record 1987 as the start date and 1988 as the end date. 4. *Numerical response*   **Percent ABF**   1. Enter the ABF percentage for the ABF group 2. If a range is not reported and instead only a single percentage point is reported, complete only the “lower range text field.” 3. If percent not included, leave blank (except for US Medicare studies).   For US Medicare studies only, assume these dates:  Medicare Phase-In Period  1. Beginning on or after October 1, 1983 and before October 1, 1984: ABF=25%  2. Beginning on or after October 1, 1984 and before October 1, 1985: ABF=50%  3. Beginning on or after October 1, 1985 and before October 1, 1986: ABF=75%  4. Beginning on or after October 1, 1986: ABF=100%   1. *Numerical response*   **“Operational Variable” column:**   1. Name the specific variable, the units in which it is measured, and the time period over which it is measured (e.g. number of hospital readmissions within 90 days of discharge). 2. *Free text*   ****Note:** if more than one relevant outcome variable is reported for one of the listed variables, record data for both variables and use numbering to indicate what data goes with variable in the free text boxes (e.g. 1. Age (year); 2. Sex)  **“Summary of results” column:**   1. Abstractors will record key data as well as summary conclusions supported by key data. Summary conclusion should be a single sentence when possible. 2. We are interested in the comparison between data points labelled A vs. B and A vs. C (not B vs. C) in the ABF vs. no ABF jurisdictions. 3. If the difference is not significant, still record the actual data numbers (e.g. percentage of readmission 33% in ABF vs. 35% in non-ABF (p=.06)). 4. If uncertain of what to abstract, consult one of the senior team members (likely Karen or Gordon or Danielle, the choice depending on the nature of the uncertainty). 5. Important to ensure that we are reporting data objectively, not inferences made by ourselves or the author(s). 6. When the author(s) provide only a narrative summary or commentary of their interpretation of data but do not provide data on which these inferences are based, do not include in results. 7. *Free text*   ****Note:** if more than one relevant outcome variable is reported for one of the listed variables, record data for both variables and use numbering to indicate what data goes with variable in the free text boxes (e.g. 1. mean age 66 in ABF vs. 68 in non-ABF (p=.06); 2. 1% more males in the ABF group (p = 0.80)).    **Difference between** **A vs. B** **in no ABF** **jurisdiction  VS** **A vs B in ABF** **jurisdiction**   1. Answer below in question 30ci   **Difference between** **A vs. C** **in no ABF** **jurisdiction VS** **A vs C in ABF** **jurisdiction**   1. Answer below in question 30cii | |
| 30 c i. **“Difference between the 0% ABF vs. early ABF periods”:** | 1. If a comparison between 0% and early ABF periods was not made in the ABF and no ABF jurisdictions select “comparison not made” 2. If the comparison is reported in the article as not significant, select “no difference”. If the comparison is reported to be significant, or no significance test was performed, the reviewer should record the direction of the effect and calculate the percentage change. 3. Use this link to calculate percentage change (enter the no ABF number first and the ABF number second): [http://www.csgnetwork.com/percentchangecalc.html](http://www.csgnetwork.com/percentchangecalc.html) 4. The formula is as follows:   time 2-time 1 in the 0% ABF periods = A  time 2 - time 1 in the ABF periods = B  then formula is B - A/A =x (100)  ****Note:** if more than one relevant outcome variable are reported in the above table check all that apply | |
| 30 c ii. **“Difference between the 0% ABF vs. later ABF periods”:** | 1. If a comparison between 0% and later ABF periods was not made in the ABF and no ABF jurisdictions select “comparison not made” 2. If the comparison is reported in the article as not significant, select “no difference”. If the comparison is reported to be significant, or no significance test was performed, the reviewer should record the direction of the effect and calculate the percentage change. 3. Use this link to calculate percentage change (enter the no ABF number first and the ABF number second): [http://www.csgnetwork.com/percentchangecalc.html](http://www.csgnetwork.com/percentchangecalc.html) 4. The formula is as follows:   time 2-time 1 in the 0% ABF periods = A  time 2 - time 1 in the ABF periods = B  then formula is B - A/A =x (100)  ****Note:** if more than one relevant outcome variable are reported in the above table check all that apply | |
| 31. Does the study **REPORT** mortality data in: | 1. See **Figure 1** for flow chart 2. Answers: 3. **Before-After study in a single jurisdiction**: 0 % ABF period (before) vs. periods with increasing ABF (after)   - The comparison is focused on *time of implementation* of ABF (e.g. before 1983, after 1983)   1. **Parallel study comparing ABF and non-ABF adopters at the same time period(s)**: Comparison of  2 (or more) jurisdictions - no ABF vs. ABF   **-** The comparison is *in two different sites at the same time period* (e.g. one outcome measure in 2003-2004 in jurisdiction 1 vs. the same outcome measure in 2003-2004 in jurisdiction 2. E.g. refID 8140)  -Some studies may conduct sequential parallel comparisons at different time periods, but note they do not account for time trends in their summary measures. Reviewers must choose the comparison(s) most relevant to our policy-level research questions. (E.g. OECD paper presents data for ABF adopting and non-adopting countries for multiple years, but does not conduct any form of time trend analysis.) | |
| Table 31a | **“Earliest No ABF data point (within 3 years of ABF implementation) (data point A)” column:**   1. Only record year (not month or day if provided). If only one year (e.g. data collected in 1984 only) record “1984” start and “1984” end. 2. If data are not available, leave blank 3. Example, if ABF was implemented in 1983, and data are provided for the years 1979, 1980 1981, and 1982; abstract data from 1980 and record 1980 as the start and end date. If ABF implementation is in 1983 and one data point is provided for 1981 and 1982 (i.e. data are aggregated), abstract data from 1981-1982 and record 1981 as the start date and 1982 as the end date. 4. *Numerical response*   **“Earliest ABF data point (Data point B)” column:**   1. Only record year (not month or day). If only one year (e.g. data collected in 1984 only) record “1984” start and “1984” end. 2. If data are not available, leave blank. 3. Example, if ABF is implemented in 1983, and hospital readmission data are provided for 1984, 1985, 1986, and 1987; abstract data for 1984 and record 1984 as the start and end date. If ABF implementation is in 1983, and one data point is provided for 1984 and 1985 (i.e. data are aggregated), abstract data from 1984-1985 and record 1984 as the start date and 1985 as the end date. 4. If data are only reported for one time period after ABF, do not complete this column. For example, if ABF was implemented in 1983 and only data for 1984 are reported, skip this row and report data in the “latest ABF point” row. 5. *Numerical response*   **“Latest ABF point (within 5 years of ABF implementation) (Data point C)” column:**   1. Only record year (not month or day if provided). If only one year (e.g. data collected in 1984 only) record “1984” start and “1984” end. 2. If data are not available, leave blank. 3. Example, if ABF was implemented in 1983, and hospital readmission data are reported in 1984, 1985, 1986, 1987, 1988, and 1989; abstract the data reported in 1988 and record 1988 as the start and end date. If ABF was implemented in 1983, and one data point is provided for 1987 and 1988 (i.e. data are aggregated), abstract data from 1987-1988 and record 1987 as the start date and 1988 as the end date. 4. *Numerical response*   **Percent ABF**   1. Enter the ABF percentage for the ABF group 2. If a range is not reported and instead only a single percentage point is reported, complete only the “lower range text field.” 3. If percent not included, leave blank (except for US Medicare studies).   For US Medicare studies only, assume these dates:  Medicare Phase-In Period  1. Beginning on or after October 1, 1983 and before October 1, 1984: ABF=25%  2. Beginning on or after October 1, 1984 and before October 1, 1985: ABF=50%  3. Beginning on or after October 1, 1985 and before October 1, 1986: ABF=75%  4. Beginning on or after October 1, 1986: ABF=100%   1. *Numerical response*   **“# of events per group” column:**   1. Record the number of events for the “No ABF”, “Earliest ABF” and “Late ABF” groups in the corresponding columns 2. This is not applicable for the “Differences” rows (row 6 and 7) 3. *Numeric response*   **“Sample size of groups” column:**   1. Record the sample size for the “No ABF”, “Earliest ABF” and “Late ABF” groups in the corresponding columns 2. *Numeric response*   **“Total sample size” column:**   1. Record the total sample size (i.e. sum of sample size of all groups reported in the paper). For example, if data are collected from 1,000 patients in 1980, 1981, 1982, 1983, and 1984, then total sample size = 5,000 (1000 x 5 groups) 2. *Numeric response*   “**Name measure of central tendency” column:**   1. Check all measures that apply and record the applicable number in the *free text* box. Include relevant positive or negative sign and decimals if applicable**.**   **“Name measure of spread or equivalent” column:**   1. Check all measures that apply and record the applicable number in the *free text* box. Include relevant positive or negative sign and decimals if applicable**.** 2. Record ranges (e.g. confidence intervals) with a hyphen without spaces (e.g. 0.9-3.2)   **Abstracting Poolable data:**  In general, we need to abstract are as follows for each outcome of interest:   1. The estimate of effect for variable of interest (impact of ABF on an outcome) 2. Its measure of spread (SD, Variance)   🡪 If measure of spread is absent, report   - 1. Standard Error (SE) OR Confidence Interval (CI)   OR statistic (t, z, F…),  OR exact p-value (not possible from approximate p-value, e.g. <0.01)   - 1. AND the numbers in each group (N1, N2) OR, at worst, sample size (N)   No need to abstract R2 for the model, or coefficients for the other variables adjusted in the model.  **To pool specific statistics we require:** | |
|  | Rate Rate change  …per 100.000 patient days | 1. Estimate (rate per patient-days) 2. Measure of spread (SD)   OR   1. SE, CI, Statistic, exact p value 2. Numbers (n1,n2 OR n) |
|  | OR  RR  HR | 1. Estimate (OR, RR, HR) 2. Measure of spread (SD)   OR   1. SE, CI, Statistic, exact p-value 2. Numbers (n1,n2 OR n) |
|  | Percent change | 1. Estimate (%) 2. Measure of spread (SD)   OR   1. SE, CI, Statistic, exact p-value 2. Numbers (n1,n2 OR n) |
|  | Raw number (n) | 1. Estimate n1, n2 2. Denominator N1 N2 |
|  | Regression coefficients  (from a  Logistic  or  Cox survival model) | 1. Estimate = coefficient Beta assessing the impact of ABF   🡪 ABF variable in a parallel study.  🡪 Time in before-after studies but time can be reported in different ways:   - - Continuous   - Dichotomous   - Categorical   - Different dummy variables  1. Measure of spread (SD)   OR   1. SE, CI, Statistic, exact p-value 2. Sample (n) and/or (n1,n2) |
| 31 a ii. Difference between the 0% ABF vs. early ABF periods: | 1. If a comparison between 0% and early ABF periods was not made select “comparison not made” 2. If the comparison is reported in the article as not significant, select “no difference”. If the comparison is reported to be significant, or no significance test was performed, the reviewer should record the direction of the effect and calculate the percentage change. 3. Use this link to calculate percentage change (enter the no ABF number first and the ABF number second): [http://www.csgnetwork.com/percentchangecalc.html](http://www.csgnetwork.com/percentchangecalc.html) 4. The formula is as follows: time 2-time 1/time 1 =x (100)= % difference between time 1 and time 2   *(time 2= earlier ABF period and/ or later ABF period; time 1= 0% ABF period)  *e.g. if death rate was 2% before ABF, and 4% after ABF, then 4-2/2 =1 (100)=100% difference | |
| 31 a iii. Difference between the 0% ABF vs. late ABF periods: | 1. If a comparison between 0% and late ABF periods was not made select “comparison not made” 2. If the comparison is reported in the article as not significant, select “no difference”. If the comparison is reported to be significant, or no significance test was performed, the reviewer should record the direction of the effect and calculate the percentage change. 3. Use this link to calculate percentage change (enter the no ABF number first and the ABF number second): [http://www.csgnetwork.com/percentchangecalc.html](http://www.csgnetwork.com/percentchangecalc.html) 4. The formula is as follows: time 2-time 1/time 1 =x (100)= % difference between time 1 and time 2   *(time 2= earlier ABF period and/ or later ABF period; time 1= 0% ABF period)  *e.g. if death rate was 2% before ABF, and 4% after ABF, then 4-2/2 =1 (100)=100% difference | |
| Table 31b | **“Start and End year of data point collection”:**   1. Only record year (not month or day). If only one year (e.g. data collected in 1984 only) record “1984” start and “1984” end. 2. If data are not available leave blank 3. *Numeric response*   **Percent ABF**   1. Enter the ABF percentage for the ABF group 2. If a range is not reported and instead only a single percentage point is reported, complete only the “lower range text field.” 3. If percent not included, leave blank (except for US Medicare studies).   For US Medicare studies only, assume these dates:  Medicare Phase-In Period  1. Beginning on or after October 1, 1983 and before October 1, 1984: ABF=25%  2. Beginning on or after October 1, 1984 and before October 1, 1985: ABF=50%  3. Beginning on or after October 1, 1985 and before October 1, 1986: ABF=75%  4. Beginning on or after October 1, 1986: ABF=100%   1. *Numerical response*   ****Note:** If you are completing multiple rows of this table you only need to record “start and end years of data point collection” and “percent ABF” columns for the first row as the answers in subsequent rows of the table will be the same. This information should be recorded on at least one row each time you begin a new table.  **“# of events per group” column:**   1. Record the number of events for the “No ABF”, “Most ABF” groups in the corresponding columns 2. This is not applicable for the “Differences between ABF and 0% ABF jurisdictions” (row5) 3. *Numeric response*   **“Sample size of groups” column:**   1. Record the sample size the “No ABF”, “Most ABF” groups in the corresponding columns 2. *Numeric response*   **“Total sample size” column:**   1. Record the total sample size (i.e. sum of sample size of all groups reported in the paper). For example, if data are collected from 1,000 patients in the USA and 1,000 patients in Canada, total sample size = 2,000 2. *Numeric response*   “**Name measure of central tendency” column:**   1. Check all measures that apply and record the applicable number in the *free text* box. Include relevant positive or negative sign and decimals if applicable**.**   **“Name measure of spread or equivalent” column:**   1. Check all measures that apply and record the applicable number in the *free text* box. Include relevant positive or negative sign and decimals if applicable**.** 2. Record ranges (e.g. confidence intervals) with a hyphen without spaces (e.g. 0.9-3.2)   **Abstracting Poolable data:**  In general, we need to abstract as follows for each outcome of interest:   1. the estimate of effect for variable of interest (impact of ABF on an outcome) 2. its measure of spread (SD, Variance)   🡪 If measure of spread is absent, report   - 1. Standard Error (SE) OR Confidence Interval (CI)   OR statistic (t, z, F…),  OR exact p-value (not possible from approximate p-value, e.g. <0.01)   - 1. AND the numbers in each group (N1, N2) OR, at worst, sample size (N)   No need to abstract R2 for the model, or coefficients for the other variables adjusted in the model.  **To pool specific statistics we require:** | |
|  | Rate Rate change  …per 100.000 patient days | 1. Estimate (rate per patient-days) 2. Measure of spread (SD)   OR   1. SE, CI, Statistic, exact p value 2. Numbers (n1,n2 OR n) |
|  | OR  RR  HR | 1. Estimate (OR, RR, HR) 2. Measure of spread (SD)   OR   1. SE, CI, Statistic, exact p-value 2. Numbers (n1,n2 OR n) |
|  | Percent change | 1. Estimate (%) 2. Measure of spread (SD)   OR   1. SE, CI, Statistic, exact p-value 2. Numbers (n1,n2 OR n) |
|  | Raw number (n) | 1. Estimate n1, n2 2. Denominator N1 N2 |
|  | Regression coefficients  (from a  Logistic  or  Cox survival model) | 1. Estimate = coefficient Beta assessing the impact of ABF   🡪 ABF variable in a parallel study.  🡪 Time in before-after studies but time can be reported in different ways:   - - Continuous   - Dichotomous   - Categorical   - Different dummy variables  1. Measure of spread (SD)   OR   1. SE, CI, Statistic, exact p-value 2. Sample (n) and/or (n1,n2) |
| 31 b ii. Difference between the 0% ABF vs. most ABF jurisdiction: | 1. If the comparison is reported in the article as not significant, select “no difference”. If the comparison is reported to be significant, or no significance test was performed, the reviewer should record the direction of the effect and calculate the percentage change. 2. Use this link to calculate percentage change (enter the no ABF number first and the ABF number second): [http://www.csgnetwork.com/percentchangecalc.html](http://www.csgnetwork.com/percentchangecalc.html) 3. The formula is as follows: time 2-time 1/time 1 =x(100)= % change between time 1 and time 2   *(Where time 2=ABF group and time 1= 0% ABF group)  *e.g. if death rate was 2% in the non-ABF, and 4% in the ABF, then 4-2/2 =1 (100)=100% difference | |

SECTION IV: DISCHARGE DESTINATION DATA SECTION

| **Discharge Destination Question Number** | **Specific Instruction** | |
| --- | --- | --- |
| 32. Does the study contain data about discharge destination after leaving acute care? |  | |
| 33. Can data from an adjusted analysis for discharge destination be abstracted? | 1. If standardized or weighted outcomes are reported, we will consider this an adjusted analysis and record all variables for which outcomes are standardized/weighted. 2. Instructions to follow if more than one analysis is presented:   **If data are non-poolable:**   1. Answer “yes” to question 33. In the table columns “operational variable” and summary of results” record both adjusted and unadjusted analysis indicating which data are which using numbers. E.g. For operational variable record: “(1) adjusted percentage discharged to PAC; (2) unadjusted percentage discharged to PAC”. For summary of results record: “(1) the adjusted percentage discharged to PAC was 20% for the ABF group and 10% for the non-ABF group; (2) the unadjusted percentage discharged to PAC was 30% for the ABF group and 15% for the non-ABF group”.   **If data are poolable:**   1. Abstract data from the adjusted analysis in Distiller and the unadjusted or less adjusted analysis in the excel document provided by Taryn. 2. Data abstracted in the Excel document will also need to be checked for agreement during conflict resolution | |
| 34a. What appropriate factors did the discharge destination analysis adjust for? | 1. Select the most appropriate factor from the list for each adjustment variable reported in the article. Only select one adjustment factor from the list for each adjustor in the paper. 2. If the analysis adjusted for a variable not on the list, select other and notify Taryn immediately by email. Include in your email a description of the variable and if the variable is an appropriate or inappropriate adjuster | |
| 34b. What possibly appropriate factors did the discharge destination analysis adjust for? | 1. Select the most appropriate factor from the list per adjustment variable reported in the article. Only select one adjustment factor from the list for each adjustor in the paper. 2. If the analysis adjusted for a variable on the list, select other and notify Taryn immediately by email. Include in your email a description of the variable and if the variable is an appropriate or inappropriate adjuster | |
| 34c. What inappropriate factors did the mortality analysis adjust for? | 1. Select the most appropriate factor from the list for each adjustment variable reported in the article. Only select one adjustment factor from the list for each adjustor in the paper. 2. If the analysis adjusted for a variable not on the list, select other and notify Taryn immediately by email. Include in your email a description of the variable and if the variable is an appropriate or inappropriate adjuster | |
| 35. Are the factors adjusted for in the discharge destination analysis comprehensive and appropriate? | 1. Rate as comprehensive adjustment unless clearly different distributions in ABF and non-ABF group, and the authors fail to adjust for that variable (e.g. More rural hospitals in the non-ABF group and more urban hospitals in a ABF group, requires an adjustment for rural/urban location) | |
| 36. Does the study report discharge destination data that could be pooled | 1. See **Figure 2** 2. See **Figure 3** 3. For all studies included in the review we will look specifically for the presence of upcoding of specific DRGs. In situations where the presence of upcoding is clear and unambiguous for specific DRGs, the data will be ineligible for pooling. If otherwise eligible these data will be included with the non-poolable results. 4. If data meet poolability criteria but a subgroup analysis was conducted:   1. Abstract total for all sub-groups (i.e. "all ages" row) if provided  2.  If total for all sub-groups is not provided, try to calculate on your own by summing ABF and non ABF groups. If only percentages and denominators are provided, calculate numerators and sum ABF and non-ABF groups.  This is the same concept as we use for dealing with multiple PAC.  3.  If not possible to sum data, consider data to be non-poolable and abstract in the non-poolable outcomes table. | |
| 37. Does the study REPORT discharge destination data in: | 1. See **Figure 1** for flow chart 2. Answers: 3. **Before-After study in a single jurisdiction**: 0 % ABF period (before) vs. periods with increasing ABF (after)   - The comparison is focused on *time of implementation* of ABF (e.g. before 1983, after 1983)   1. **Parallel study comparing ABF and non-ABF adopters at the same time period(s)**: Comparison of  2 (or more) jurisdictions - no ABF vs. ABF   **-** The comparison is *in two different sites at the same time period* (e.g. one outcome measure in 2003-2004 in jurisdiction 1 vs. the same outcome measure in 2003-2004 in jurisdiction 2. E.g. refID 8140)  -Some studies may conduct sequential parallel comparisons at different time periods, but note they do not account for time trends in their summary measures. Reviewers must choose the comparison(s) most relevant to our policy-level research questions. (E.g. OECD paper presents data for ABF adopting and non-adopting countries for multiple years, but does not conduct any form of time trend analysis.)   1. **Before-after study in ABF-adopters COUPLED WITH a parallel comparison with non-adopters, reporting repeated measures over time and an analysis to account for time trends.**   **-**One (or more) jurisdictions which gradually implemented ABF (before-after) compared to one (or more) that did not implement ABF. The article reports data at several time-points and account for time trends in their analysis (e.g. Farrar, Kjerstad – in contrast with OECD).  **-**The *analysis has to account for time trend*, e.g. "difference in difference", "time series", or multivariate regression models accounting for time and jurisdiction (usually with interaction variable between the two). In these complex analyses, the outcome data CANNOT be pooled.  **-**We use the same guidance as for the other designs to judge appropriateness and comprehensiveness of the adjustment. E.g. Farrar reported a difference-in-difference for readmission, which is inappropriately unadjusted. | |
| Table 37a | **“Earliest No ABF data point (within 3 years of ABF implementation) (data point A)” column:**   1. Only record year (not month or day if provided). If only one year (e.g. data collected in 1984 only) record “1984” start and “1984” end. 2. If data are not available, leave blank 3. Example, if ABF was implemented in 1983, and data are provided for the years 1979, 1980 1981, and 1982; abstract data from 1980 and record 1980 as the start and end date. If ABF implementation is in 1983 and one data point is provided for 1981 and 1982 (i.e. data are aggregated), abstract data from 1981-1982 and record 1981 as the start date and 1982 as the end date. 4. *Numerical response*   **“Earliest ABF data point (Data point B)” column:**   1. Only record year (not month or day). If only one year (e.g. data collected in 1984 only) record “1984” start and “1984” end. 2. If data are not available, leave blank. 3. Example, if ABF is implemented in 1983, and hospital readmission data are provided for 1984, 1985, 1986, and 1987; abstract data for 1984 and record 1984 as the start and end date. If ABF implementation is in 1983, and one data point is provided for 1984 and 1985 (i.e. data are aggregated), abstract data from 1984-1985 and record 1984 as the start date and 1985 as the end date. 4. If data are only reported for one time period after ABF, do not complete this column. For example, if ABF was implemented in 1983 and only data for 1984 are reported, skip this row and report data in the “latest ABF point” row. 5. *Numerical response*   **“Latest ABF point (within 5 years of ABF implementation) (Data point C)” column:**   1. Only record year (not month or day if provided). If only one year (e.g. data collected in 1984 only) record “1984” start and “1984” end. 2. If data are not available, leave blank. 3. Example, if ABF was implemented in 1983, and hospital readmission data are reported in 1984, 1985, 1986, 1987, 1988, and 1989; abstract the data reported in 1988 and record 1988 as the start and end date. If ABF was implemented in 1983, and one data point is provided for 1987 and 1988 (i.e. data are aggregated), abstract data from 1987-1988 and record 1987 as the start date and 1988 as the end date. 4. *Numerical response*   **Percent ABF**   1. Enter the ABF percentage for the ABF group 2. If a range is not reported and instead only a single percentage point is reported, complete only the “lower range text field.” 3. If percent not included, leave blank (except for US Medicare studies).   For US Medicare studies only, assume these dates:  Medicare Phase-In Period  1. Beginning on or after October 1, 1983 and before October 1, 1984: ABF=25%  2. Beginning on or after October 1, 1984 and before October 1, 1985: ABF=50%  3. Beginning on or after October 1, 1985 and before October 1, 1986: ABF=75%  4. Beginning on or after October 1, 1986: ABF=100%   1. *Numerical response*   **“Operational Variable” column:**   1. Name the specific variable, the units in which it is measured, and the time period over which it is measured (e.g. number of hospital readmissions within 90 days of discharge). 2. *Free text*   ****Note:** if more than one relevant outcome variable is reported for one of the listed variables, record data for both variables and use numbering to indicate what data goes with variable in the free text boxes (e.g. 1. Age (year); 2. Sex)  **“Summary of results” column:**   1. Abstractors will record key data as well as summary conclusions supported by key data. Summary conclusion should be a single sentence when possible. 2. We are interested in the comparison between data points labelled A vs. B and A vs. C (not B vs. C). 3. If the difference is not significant, still record the actual data numbers (e.g. percentage of readmission 33% in ABF vs. 35% in non-ABF (p=.06)). 4. If uncertain of what to abstract, consult one of the senior team members (likely Karen or Gordon or Danielle, the choice depending on the nature of the uncertainty). 5. Important to ensure that we are reporting data objectively, not inferences made by ourselves or the author(s). 6. When the author(s) provide only a narrative summary or commentary of their interpretation of data but do not provide data on which these inferences are based, do not include in results. 7. *Free text*   ****Note:** if more than one relevant outcome variable is reported for one of the listed variables, record data for both variables and use numbering to indicate what data goes with variable in the free text boxes (e.g. 1. mean age 66 in ABF vs. 68 in non-ABF (p=.06); 2. 1% more males in the ABF group (p = 0.80)).  **Difference between** **the 0% ABF vs.** **early ABF periods**   1. Answer below table in question 37ai   **Difference between** **the 0% ABF vs.** **later ABF periods**   1. Answer below table in question 37aii | |
| 37 a i. **“Difference between the 0% ABF vs. early ABF periods”:** | 1. If a comparison between 0% and early ABF periods was not made select “comparison not made.” 2. If the comparison is reported in the article as not significant, select “no difference.” If the comparison is reported to be significant or no significance test was performed, the reviewer should record the direction of the effect and calculate the percentage change. 3. Use this link to calculate percentage change (enter the no ABF number first and the ABF number second): [http://www.csgnetwork.com/percentchangecalc.html](http://www.csgnetwork.com/percentchangecalc.html) 4. OR, The formula to calculate percent change is as follows: time 2-time 1/time 1 = x (100)= % change between time 1 and time 2   *(time 2= earlier ABF period and/ or later ABF period; time 1= 0% ABF period)  *e.g. if death rate was 2% before ABF, and 4% after ABF, then 4-2/2 = 1 (100)=100% difference  ****Note:** if more than one relevant outcome variable are reported in the above table check all that apply. | |
| 37 a ii **“Difference between the 0% ABF vs. later ABF periods”:** | 1. If a comparison between 0% and late ABF periods was not made select “comparison not made.” 2. If the comparison is reported in the article as not significant, select “no difference.” If the comparison is reported to be significant or no significance test was performed, the reviewer should record the direction of the effect and calculate the percentage change. 3. Use this link to calculate percentage change (enter the no ABF number first and the ABF number second): [http://www.csgnetwork.com/percentchangecalc.html](http://www.csgnetwork.com/percentchangecalc.html) 4. OR, The formula to calculate percent change is as follows: time 2-time 1/time 1 = x (100)= % change between time 1 and time 2   *(time 2= earlier ABF period and/ or later ABF period; time 1= 0% ABF period)  *e.g. if death rate was 2% before ABF, and 4% after ABF, then 4-2/2 = 1 (100)=100% difference  ****Note:** if more than one relevant outcome variable are reported in the above table check all that apply. | |
| Table 37b | **“Start and End year of data point collection”:**   1. Only record year (not month or day). If only one year (e.g. data collected in 1984 only) record “1984” start and “1984” end. 2. If data are not available leave blank 3. *Numeric response*   **Percent ABF**   1. Enter the ABF percentage for the ABF group 2. If a range is not reported and instead only a single percentage point is reported, complete only the “lower range text field.” 3. If percent not included, leave blank (except for US Medicare studies).   For US Medicare studies only, assume these dates:  Medicare Phase-In Period  1. Beginning on or after October 1, 1983 and before October 1, 1984: ABF=25%  2. Beginning on or after October 1, 1984 and before October 1, 1985: ABF=50%  3. Beginning on or after October 1, 1985 and before October 1, 1986: ABF=75%  4. Beginning on or after October 1, 1986: ABF=100%   1. Numerical response   **“Operational Variable” column:**   1. Name the specific variable, the units in which it is measured, and the time period over which it is measured (e.g. number of hospital readmissions within 90 days of discharge). 2. Free text   ****Note:** if more than one relevant outcome variable is reported for one of the listed variables, record data for both variables and use numbering to indicate what data goes with variable in the free text boxes (e.g. 1. Age (year); 2. Sex)  **“Summary of results” column:**   1. Abstractors will record key data as well as summary conclusions supported by key data. Summary conclusion should be a single sentence when possible. 2. If the difference is not significant, still record the actual data numbers (e.g. percentage of readmission 33% in ABF vs. 35% in non-ABF (p=.06)). 3. If uncertain of what to abstract, consult one of the senior team members (likely Karen or Gordon or Danielle, the choice depending on the nature of the uncertainty). 4. Important to ensure that we are reporting data objectively, not inferences made by ourselves or the author(s). 5. When the author(s) provide only a narrative summary or commentary of their interpretation of data but do not provide data on which these inferences are based, do not include in results. 6. Free text   ****Note:** if more than one relevant outcome variable is reported for one of the listed variables, record data for both variables and use numbering to indicate what data goes with variable in the free text boxes (e.g. 1. mean age 66 in ABF vs. 68 in non-ABF (p=.06); 2. 1% more males in the ABF group (p = 0.80)).  **Difference between the** **0% ABF vs. most ABF** **jurisdiction**   1. Answer below in question 37bi | |
| 37 b i. **“Difference between the 0% ABF vs. most ABF jurisdictions”:** | 1. If a comparison between 0% and most ABF jurisdiction was not made, select “comparison not made.” 2. If the comparison is reported in the article as not significant, select “no difference.” If the comparison is reported to be significant or no significance test was performed, the reviewer should record the direction of the effect and calculate the percentage change. 3. Use this link to calculate percentage change (enter the no ABF number first and the ABF number second): [http://www.csgnetwork.com/percentchangecalc.html](http://www.csgnetwork.com/percentchangecalc.html) 4. OR, The formula to calculate percent change is as follows: time 2-time 1/time 1 = x (100)= % change between time 1 and time 2   *(Where time 2=ABF group and time 1= 0% ABF group)  *e.g. if death rate was 2% in the non-ABF, and 4% in the ABF, then 4-2/2 =1 (100)=100% difference  ****Note:** if more than one relevant outcome variable are reported in the above table check all that apply | |
| Table 37c | **“Earliest No ABF data point (within 3 years of ABF implementation) (data point A)” column:**   1. Only record year (not month or day if provided). If only one year (e.g. data collected in 1984 only) record “1984” start and “1984” end. 2. If data are not available, leave blank 3. Example, if ABF was implemented in 1983, and data are provided for the years 1979, 1980 1981, and 1982; abstract data from 1980 and record 1980 as the start and end date. If ABF implementation is in 1983 and one data point is provided for 1981 and 1982 (i.e. data are aggregated), abstract data from 1981-1982 and record 1981 as the start date and 1982 as the end date. 4. *Numerical response*   **“Earliest ABF data point (Data point B)” column:**   1. Only record year (not month or day). If only one year (e.g. data collected in 1984 only) record “1984” start and “1984” end. 2. If data are not available, leave blank. 3. Example, if ABF is implemented in 1983, and hospital readmission data are provided for 1984, 1985, 1986, and 1987; abstract data for 1984 and record 1984 as the start and end date. If ABF implementation is in 1983, and one data point is provided for 1984 and 1985 (i.e. data are aggregated), abstract data from 1984-1985 and record 1984 as the start date and 1985 as the end date. 4. If data are only reported for one time period after ABF, do not complete this column. For example, if ABF was implemented in 1983 and only data for 1984 are reported, skip this row and report data in the “latest ABF point” row. 5. *Numerical response*   **“Latest ABF point (within 5 years of ABF implementation) (Data point C)” column:**   1. Only record year (not month or day if provided). If only one year (e.g. data collected in 1984 only) record “1984” start and “1984” end. 2. If data are not available, leave blank. 3. Example, if ABF was implemented in 1983, and hospital readmission data are reported in 1984, 1985, 1986, 1987, 1988, and 1989; abstract the data reported in 1988 and record 1988 as the start and end date. If ABF was implemented in 1983, and one data point is provided for 1987 and 1988 (i.e. data are aggregated), abstract data from 1987-1988 and record 1987 as the start date and 1988 as the end date. 4. *Numerical response*   **Percent ABF**   1. Enter the ABF percentage for the ABF group 2. If a range is not reported and instead only a single percentage point is reported, complete only the “lower range text field.” 3. If percent not included, leave blank (except for US Medicare studies).   For US Medicare studies only, assume these dates:  Medicare Phase-In Period  1. Beginning on or after October 1, 1983 and before October 1, 1984: ABF=25%  2. Beginning on or after October 1, 1984 and before October 1, 1985: ABF=50%  3. Beginning on or after October 1, 1985 and before October 1, 1986: ABF=75%  4. Beginning on or after October 1, 1986: ABF=100%   1. *Numerical response*   **“Operational Variable” column:**   1. Name the specific variable, the units in which it is measured, and the time period over which it is measured (e.g. number of hospital readmissions within 90 days of discharge). 2. *Free text*   ****Note:** if more than one relevant outcome variable is reported for one of the listed variables, record data for both variables and use numbering to indicate what data goes with variable in the free text boxes (e.g. 1. Age (year); 2. Sex)  **“Summary of results” column:**   1. Abstractors will record key data as well as summary conclusions supported by key data. Summary conclusion should be a single sentence when possible. 2. We are interested in the comparison between data points labelled A vs. B and A vs. C (not B vs. C) in the ABF vs. no ABF jurisdictions. 3. If the difference is not significant, still record the actual data numbers (e.g. percentage of readmission 33% in ABF vs. 35% in non-ABF (p=.06)). 4. If uncertain of what to abstract, consult one of the senior team members (likely Karen or Gordon or Danielle, the choice depending on the nature of the uncertainty). 5. Important to ensure that we are reporting data objectively, not inferences made by ourselves or the author(s). 6. When the author(s) provide only a narrative summary or commentary of their interpretation of data but do not provide data on which these inferences are based, do not include in results. 7. *Free text*   ****Note:** if more than one relevant outcome variable is reported for one of the listed variables, record data for both variables and use numbering to indicate what data goes with variable in the free text boxes (e.g. 1. mean age 66 in ABF vs. 68 in non-ABF (p=.06); 2. 1% more males in the ABF group (p = 0.80)).    **Difference between** **A vs. B** **in no ABF** **jurisdiction  VS** **A vs B in ABF** **jurisdiction**   1. Answer below in question 37ci   **Difference between** **A vs. C** **in no ABF** **jurisdiction VS** **A vs C in ABF** **jurisdiction**   1. Answer below in question 37cii | |
| 37 c i. **“Difference between the 0% ABF vs. early ABF periods”:** | 1. If a comparison between 0% and early ABF periods was not made in the ABF and no ABF jurisdictions select “comparison not made” 2. If the comparison is reported in the article as not significant, select “no difference”. If the comparison is reported to be significant, or no significance test was performed, the reviewer should record the direction of the effect and calculate the percentage change. 3. Use this link to calculate percentage change (enter the no ABF number first and the ABF number second): [http://www.csgnetwork.com/percentchangecalc.html](http://www.csgnetwork.com/percentchangecalc.html) 4. The formula is as follows:   time 2-time 1 in the 0% ABF periods = A  time 2 - time 1 in the ABF periods = B  then formula is B - A/A =x (100)  ****Note:** if more than one relevant outcome variable are reported in the above table check all that apply | |
| 37 c ii. **“Difference between the 0% ABF vs. later ABF periods”:** | 1. If a comparison between 0% and later ABF periods was not made in the ABF and no ABF jurisdictions select “comparison not made” 2. If the comparison is reported in the article as not significant, select “no difference”. If the comparison is reported to be significant, or no significance test was performed, the reviewer should record the direction of the effect and calculate the percentage change. 3. Use this link to calculate percentage change (enter the no ABF number first and the ABF number second): [http://www.csgnetwork.com/percentchangecalc.html](http://www.csgnetwork.com/percentchangecalc.html) 4. The formula is as follows:   time 2-time 1 in the 0% ABF periods = A  time 2 - time 1 in the ABF periods = B  then formula is B - A/A =x (100)  ****Note:** if more than one relevant outcome variable are reported in the above table check all that apply | |
| 38. Does the study REPORT discharge destination data in: | 1. See **Figure 1** for flow chart 2. Answers: 3. **Before-After study in a single jurisdiction**: 0 % ABF period (before) vs. periods with increasing ABF (after)   - The comparison is focused on *time of implementation* of ABF (e.g. before 1983, after 1983)   1. **Parallel study comparing ABF and non-ABF adopters at the same time period(s)**: Comparison of  2 (or more) jurisdictions - no ABF vs. ABF   **-** The comparison is *in two different sites at the same time period* (e.g. one outcome measure in 2003-2004 in jurisdiction 1 vs. the same outcome measure in 2003-2004 in jurisdiction 2. E.g. refID 8140)  -Some studies may conduct sequential parallel comparisons at different time periods, but note they do not account for time trends in their summary measures. Reviewers must choose the comparison(s) most relevant to our policy-level research questions. (E.g. OECD paper presents data for ABF adopting and non-adopting countries for multiple years, but does not conduct any form of time trend analysis.) | |
| Table 38a | 1. Abstract data based on the aggregated PAC data (but *not* data for discharged home or dead). Home health agency (HHA) should be considered PAC, as this is not the same as “home”. If only ratios or percent changes are reported (and therefore PAC data cannot be aggregated), then email Taryn for instructions on how to answer this question (note: this is a very rare case and if it occurs Taryn will record all applicable answers in data cleaning). Refer to **Figure 3** for instructions on how to abstract discharge destination data. 2. Select applicable discharge destination   **Home** = discharged home with self/family care  **HHA** = discharged home with Home Health Agency care (US Medicare)  **SNF** = discharged to Skilled Nursing Facility care (US Medicare)  **IRF** = discharged to Inpatient Rehabilitation Facility care (US Medicare)  **LTCH** = discharged to Long-Term Care Hospital care (US Medicare)  **PAC NOS** = discharged to Post-Acute Care Not Otherwise Specified (use if data are aggregated across different post-acute care destinations)*  **Dead** = discharged dead  *we will use the “PAC NOS” category for any non-Medicare post-acute care identified in the international literature, as all other post-acute care categories are US-Medicare specific.   1. **NOTE:** we will not consider the following as post-acute care:   **Short-stay hospitals**  **Transferred to another hospital/facility**  If the article does not provide guidance on whether a facility can be classified as post-acute care, contact Taryn copying Karen.  ****Note:** the focus of these categories is on US Medicare as the majority of the discharge destination literature is from US Medicare studies. This is likely because ABF has not been implemented in other countries long enough for this body of literature to have emerged.   1. Follow the below hierarchy for how to abstract poolable data with the most preferable option listed as number 1 and the least preferable option listed as 2c. 2. If the proportions and denominators are directly available for discharge to PAC, we abstract them. *This is simple case were you have 4 numbers in the 2x2 table and you need no maths (n/N in ABF, n/N in non-ABF) or (%/N in ABF, %/N in non-ABF)* 3. If the proportions and denominators are broken into several PAC destination, we can do one of the following: 4. Add the numbers: PAC =PAC1 + PAC2 + PAC3, in the ABF and non-ABF group, and abstract the denominators. *This is when discharge destination are reported separately and you need to try and sum up numerators.*   *****Note****: we will not consider “transferred to another hospital” as discharged to PAC as this could reflect transfer to an acute care hospital.*   1. Deduce this number from those discharged home minus those discharged dead: PAC = 100% - Home - discharged dead. (This is when discharged dead can be useful). 2. If A or B are not possible (e.g. one regression coefficient for 3 different destinations), we will abstract a narrative summary (i.e. abstract in the non-poolable outcome table). 3. Select next applicable discharge destination and repeat until data are recorded for all applicable discharge destinations   **“Earliest No ABF data point (within 3 years of ABF implementation) (data point A)” column:**   1. Only record year (not month or day if provided). If only one year (e.g. data collected in 1984 only) record “1984” start and “1984” end. 2. If data are not available, leave blank 3. Example, if ABF was implemented in 1983, and data are provided for the years 1979, 1980 1981, and 1982; abstract data from 1980 and record 1980 as the start and end date. If ABF implementation is in 1983 and one data point is provided for 1981 and 1982 (i.e. data are aggregated), abstract data from 1981-1982 and record 1981 as the start date and 1982 as the end date. 4. *Numerical response*   **“Earliest ABF data point (Data point B)” column:**   1. Only record year (not month or day). If only one year (e.g. data collected in 1984 only) record “1984” start and “1984” end. 2. If data are not available, leave blank. 3. Example, if ABF is implemented in 1983, and hospital readmission data are provided for 1984, 1985, 1986, and 1987; abstract data for 1984 and record 1984 as the start and end date. If ABF implementation is in 1983, and one data point is provided for 1984 and 1985 (i.e. data are aggregated), abstract data from 1984-1985 and record 1984 as the start date and 1985 as the end date. 4. If data are only reported for one time period after ABF, do not complete this column. For example, if ABF was implemented in 1983 and only data for 1984 are reported, skip this row and report data in the “latest ABF point” row. 5. *Numerical response*   **“Latest ABF point (within 5 years of ABF implementation) (Data point C)” column:**   1. Only record year (not month or day if provided). If only one year (e.g. data collected in 1984 only) record “1984” start and “1984” end. 2. If data are not available, leave blank. 3. Example, if ABF was implemented in 1983, and hospital readmission data are reported in 1984, 1985, 1986, 1987, 1988, and 1989; abstract the data reported in 1988 and record 1988 as the start and end date. If ABF was implemented in 1983, and one data point is provided for 1987 and 1988 (i.e. data are aggregated), abstract data from 1987-1988 and record 1987 as the start date and 1988 as the end date. 4. *Numerical response*   **Percent ABF**   1. Enter the ABF percentage for the ABF group 2. If a range is not reported and instead only a single percentage point is reported, complete only the “lower range text field.” 3. If percent not included, leave blank (except for US Medicare studies).   For US Medicare studies only, assume these dates:  Medicare Phase-In Period  1. Beginning on or after October 1, 1983 and before October 1, 1984: ABF=25%  2. Beginning on or after October 1, 1984 and before October 1, 1985: ABF=50%  3. Beginning on or after October 1, 1985 and before October 1, 1986: ABF=75%  4. Beginning on or after October 1, 1986: ABF=100%   1. *Numerical response*   **“# of events per group” column:**   1. Record the number of events for the “No ABF”, “Earliest ABF” and “Late ABF” groups in the corresponding columns 2. This is not applicable for the “Differences” rows (row 6 and 7) 3. *Numeric response*   **“Sample size of groups” column:**   1. Record the sample size for the “No ABF”, “Earliest ABF” and “Late ABF” groups in the corresponding columns 2. *Numeric response*   **“Total sample size” column:**   1. Record the total sample size (i.e. sum of sample size of all groups reported in the paper). For example, if data are collected from 1,000 patients in 1980, 1981, 1982, 1983, and 1984, then total sample size = 5,000 (1000 x 5 groups) 2. *Numeric response*   “**Name measure of central tendency” column:**   1. Check all measures that apply and record the applicable number in the *free text* box. Include relevant positive or negative sign and decimals if applicable**.**   **“Name measure of spread or equivalent” column:**   1. Check all measures that apply and record the applicable number in the *free text* box. Include relevant positive or negative sign and decimals if applicable**.** 2. Record ranges (e.g. confidence intervals) with a hyphen without spaces (e.g. 0.9-3.2)   **Abstracting Poolable data:**  In general, we need to abstract are as follows for each outcome of interest:   1. The estimate of effect for variable of interest (impact of ABF on an outcome) 2. Its measure of spread (SD, Variance)   🡪 If measure of spread is absent, report   - 1. Standard Error (SE) OR Confidence Interval (CI)   OR statistic (t, z, F…),  OR exact p-value (not possible from approximate p-value, e.g. <0.01)   - 1. AND the numbers in each group (N1, N2) OR, at worst, sample size (N)   No need to abstract R2 for the model, or coefficients for the other variables adjusted in the model.  **To pool specific statistics we require:** | |
|  | Rate Rate change  …per 100.000 patient days | 1. Estimate (rate per patient-days) 2. Measure of spread (SD)   OR   1. SE, CI, Statistic, exact p value 2. Numbers (n1,n2 OR n) |
|  | OR  RR  HR | 1. Estimate (OR, RR, HR) 2. Measure of spread (SD)   OR   1. SE, CI, Statistic, exact p-value 2. Numbers (n1,n2 OR n) |
|  | Percent change | 1. Estimate (%) 2. Measure of spread (SD)   OR   1. SE, CI, Statistic, exact p-value 2. Numbers (n1,n2 OR n) |
|  | Raw number (n) | 1. Estimate n1, n2 2. Denominator N1 N2 |
|  | Regression coefficients  (from a  Logistic  or  Cox survival model) | 1. Estimate = coefficient Beta assessing the impact of ABF   🡪 ABF variable in a parallel study.  🡪 Time in before-after studies but time can be reported in different ways:   - - Continuous   - Dichotomous   - Categorical   - Different dummy variables  1. Measure of spread (SD)   OR   1. SE, CI, Statistic, exact p-value 2. Sample (n) and/or (n1,n2) |
| 38 a ii. Difference between the 0% ABF vs. early ABF periods: | 1. If a comparison between 0% and early ABF periods was not made select “comparison not made” 2. If the comparison is reported in the article as not significant, select “no difference”. If the comparison is reported to be significant, or no significance test was performed, the reviewer should record the direction of the effect and calculate the percentage change. 3. Use this link to calculate percentage change (enter the no ABF number first and the ABF number second): [http://www.csgnetwork.com/percentchangecalc.html](http://www.csgnetwork.com/percentchangecalc.html) 4. The formula is as follows: time 2-time 1/time 1 =x (100)= % difference between time 1 and time 2   *(time 2= earlier ABF period and/ or later ABF period; time 1= 0% ABF period)  *e.g. if death rate was 2% before ABF, and 4% after ABF, then 4-2/2 =1 (100)=100% difference | |
| 38 a iii. Difference between the 0% ABF vs. late ABF periods: | 1. If a comparison between 0% and late ABF periods was not made select “comparison not made” 2. If the comparison is reported in the article as not significant, select “no difference”. If the comparison is reported to be significant, or no significance test was performed, the reviewer should record the direction of the effect and calculate the percentage change. 3. Use this link to calculate percentage change (enter the no ABF number first and the ABF number second): [http://www.csgnetwork.com/percentchangecalc.html](http://www.csgnetwork.com/percentchangecalc.html) 4. The formula is as follows: time 2-time 1/time 1 =x (100)= % difference between time 1 and time 2   *(time 2= earlier ABF period and/ or later ABF period; time 1= 0% ABF period)  *e.g. if death rate was 2% before ABF, and 4% after ABF, then 4-2/2 =1 (100)=100% difference | |
| Table 38b | 1. Abstract data based on the aggregated PAC data (but *not* data for discharged home or dead). Home health agency (HHA) should be considered PAC, as this is not the same as “home”. If only ratios or percent changes are reported (and therefore PAC data cannot be aggregated), then email Taryn for instructions on how to answer this question (note: this is a very rare case and if it occurs Taryn will record all applicable answers in data cleaning). Refer to **Figure 3** for instructions on how to abstract discharge destination data. 2. Select applicable discharge destination   **Home** = discharged home with self/family care  **HHA** = discharged home with Home Health Agency care (US Medicare)  **SNF** = discharged to Skilled Nursing Facility care (US Medicare)  **IRF** = discharged to Inpatient Rehabilitation Facility care (US Medicare)  **LTCH** = discharged to Long-Term Care Hospital care (US Medicare)  **PAC NOS** = discharged to Post-Acute Care Not Otherwise Specified (use if data are aggregated across different post-acute care destinations)*  **Dead** = discharged dead  *we will use the “PAC NOS” category for any non-Medicare post-acute care identified in the international literature, as all other post-acute care categories are US-Medicare specific.   1. **NOTE:** we will not consider the following as post-acute care:   **Short-stay hospitals**  **Transferred to another hospital/facility**  If the article does not provide guidance on whether a facility can be classified as post-acute care, contact Taryn copying Karen.  ****Note:** the focus of these categories is on US Medicare as the majority of the discharge destination literature is from US Medicare studies. This is likely because ABF has not been implemented in other countries long enough for this body of literature to have emerged.   1. Follow the below hierarchy for how to abstract poolable data with the most preferable option listed as number 1 and the least preferable option listed as 2c. 2. If the proportions and denominators are directly available for discharge to PAC, we abstract them. *This is simple case were you have 4 numbers in the 2x2 table and you need no maths (n/N in ABF, n/N in non-ABF) or (%/N in ABF, %/N in non-ABF)* 3. If the proportions and denominators are broken into several PAC destination, we can do one of the following: 4. Add the numbers: PAC =PAC1 + PAC2 + PAC3, in the ABF and non-ABF group, and abstract the denominators. *This is when discharge destination are reported separately and you need to try and sum up numerators.*   ****Note:** we will not consider “transferred to another hospital” as discharged to PAC as this could reflect transfer to an acute care hospital*.*   1. Deduce this number from those discharged home minus those discharged dead: PAC = 100% - Home - discharged dead. (This is when discharged dead can be useful). 2. If A or B are not possible (e.g. one regression coefficient for 3 different destinations), we will abstract a narrative summary (i.e. abstract in the non-poolable outcome table). 3. Select next applicable discharge destination and repeat until data are recorded for all applicable discharge destinations   **“Start and End year of data point collection”:**   1. Only record year (not month or day). If only one year (e.g. data collected in 1984 only) record “1984” start and “1984” end. 2. If data are not available leave blank 3. *Numeric response*   **Percent ABF**   1. Enter the ABF percentage for the ABF group 2. If a range is not reported and instead only a single percentage point is reported, complete only the “lower range text field.” 3. If percent not included, leave blank (except for US Medicare studies).   For US Medicare studies only, assume these dates:  Medicare Phase-In Period  1. Beginning on or after October 1, 1983 and before October 1, 1984: ABF=25%  2. Beginning on or after October 1, 1984 and before October 1, 1985: ABF=50%  3. Beginning on or after October 1, 1985 and before October 1, 1986: ABF=75%  4. Beginning on or after October 1, 1986: ABF=100%   1. *Numerical response*   **Note: If you are completing multiple rows of this table you only need to record “start and end years of data point collection” and “percent ABF” columns for the first row as the answers in subsequent rows of the table will be the same. This information should be recorded on at least one row each time you begin a new table.  **“# of events per group” column:**   1. Record the number of events for the “No ABF”, “Most ABF” groups in the corresponding columns 2. This is not applicable for the “Differences between ABF and 0% ABF jurisdictions” (row5) 3. *Numeric response*   **“Sample size of groups” column:**   1. Record the sample size the “No ABF”, “Most ABF” groups in the corresponding columns 2. *Numeric response*   **“Total sample size” column:**   1. Record the total sample size (i.e. sum of sample size of all groups reported in the paper). For example, if data are collected from 1,000 patients in the USA and 1,000 patients in Canada, total sample size = 2,000 2. *Numeric response*   “**Name measure of central tendency” column:**   1. Check all measures that apply and record the applicable number in the *free text* box. Include relevant positive or negative sign and decimals if applicable**.**   **“Name measure of spread or equivalent” column:**   1. Check all measures that apply and record the applicable number in the *free text* box. Include relevant positive or negative sign and decimals if applicable**.** 2. Record ranges (e.g. confidence intervals) with a hyphen without spaces (e.g. 0.9-3.2)   **Abstracting Poolable data:**  In general, we need to abstract as follows for each outcome of interest:   1. the estimate of effect for variable of interest (impact of ABF on an outcome) 2. its measure of spread (SD, Variance)   🡪 If measure of spread is absent, report   - 1. Standard Error (SE) OR Confidence Interval (CI)   OR statistic (t, z, F…),  OR exact p-value (not possible from approximate p-value, e.g. <0.01)   - 1. AND the numbers in each group (N1, N2) OR, at worst, sample size (N)   No need to abstract R2 for the model, or coefficients for the other variables adjusted in the model.  **To pool specific statistics we require:** | |
|  | Rate Rate change  …per 100.000 patient days | 1. Estimate (rate per patient-days) 2. Measure of spread (SD)   OR   1. SE, CI, Statistic, exact p value 2. Numbers (n1,n2 OR n) |
|  | OR  RR  HR | 1. Estimate (OR, RR, HR) 2. Measure of spread (SD)   OR   1. SE, CI, Statistic, exact p-value 2. Numbers (n1,n2 OR n) |
|  | Percent change | 1. Estimate (%) 2. Measure of spread (SD)   OR   1. SE, CI, Statistic, exact p-value 2. Numbers (n1,n2 OR n) |
|  | Raw number (n) | 1. Estimate n1, n2 2. Denominator N1 N2 |
|  | Regression coefficients  (from a  Logistic  or  Cox survival model) | 1. Estimate = coefficient Beta assessing the impact of ABF   🡪 ABF variable in a parallel study.  🡪 Time in before-after studies but time can be reported in different ways:   - - Continuous   - Dichotomous   - Categorical   - Different dummy variables  1. Measure of spread (SD)   OR   1. SE, CI, Statistic, exact p-value 2. Sample (n) and/or (n1,n2) |
| 38 b ii. Difference between the 0% ABF vs. most ABF jurisdiction: | 1. If the comparison is reported in the article as not significant, select “no difference”. If the comparison is reported to be significant, or no significance test was performed, the reviewer should record the direction of the effect and calculate the percentage change. 2. Use this link to calculate percentage change (enter the no ABF number first and the ABF number second): [http://www.csgnetwork.com/percentchangecalc.html](http://www.csgnetwork.com/percentchangecalc.html) 3. The formula is as follows: time 2-time 1/time 1 =x(100)= % change between time 1 and time 2   *(Where time 2=ABF group and time 1= 0% ABF group)  *e.g. if death rate was 2% in the non-ABF, and 4% in the ABF, then 4-2/2 =1 (100)=100% difference | |

SECTION VI: NON-POOLABLE OUTCOMES

| **Non-Poolable Outcomes Question Number** | **Specific Instruction** |
| --- | --- |
| 39. Does the study report data on non-poolable outcome variables (cost, volume of care, characteristics of study population, or severity of illness/casemix) |  |
| 40. Does the study **REPORT** non-poolable outcome measures data in: | 1. See **Figure 1** for flow chart 2. Answers: 3. **Before-After study in a single jurisdiction**: 0 % ABF period (before) vs. periods with increasing ABF (after)   - The comparison is focused on *time of implementation* of ABF (e.g. before 1983, after 1983)   1. **Parallel study comparing ABF and non-ABF adopters at the same time period(s)**: Comparison of  2 (or more) jurisdictions - no ABF vs. ABF   **-** The comparison is *in two different sites at the same time period* (e.g. one outcome measure in 2003-2004 in jurisdiction 1 vs. the same outcome measure in 2003-2004 in jurisdiction 2. E.g. refID 8140)  -Some studies may conduct sequential parallel comparisons at different time periods, but note they do not account for time trends in their summary measures. Reviewers must choose the comparison(s) most relevant to our policy-level research questions. (E.g. OECD paper presents data for ABF adopting and non-adopting countries for multiple years, but does not conduct any form of time trend analysis.)   1. **Before-after study in ABF-adopters COUPLED WITH a parallel comparison with non-adopters, reporting repeated measures over time and an analysis to account for time trends.**   **-**One (or more) jurisdictions which gradually implemented ABF (before-after) compared to one (or more) that did not implement ABF. The article reports data at several time-points and account for time trends in their analysis (e.g. Farrar, Kjerstad – in contrast with OECD).  **-**The *analysis has to account for time trend*, e.g. "difference in difference", "time series", or multivariate regression models accounting for time and jurisdiction (usually with interaction variable between the two). In these complex analyses, the outcome data CANNOT be pooled.  **-**We use the same guidance as for the other designs to judge appropriateness and comprehensiveness of the adjustment. E.g. Farrar reported a difference-in-difference for readmission, which is inappropriately unadjusted. |
| 45. Does the study report differences in volume of care? | 1. E.g. number of admissions, number of patients treated |
| 46. Can data from an adjusted analysis for volume of care be abstracted? | 1. If more than one analysis is presented: Answer “yes” to question 46. In the table columns “operational variable” and summary of results” record both adjusted and unadjusted analysis indicating which data are which using numbers. E.g. For operational variable record: “(1) adjusted number of admissions per year; (2) unadjusted number of admissions per year”. For summary of results record: “(1) the adjusted number of admissions per year was 100,000 for the ABF group and 50,000 for the non-ABF group; (2) the unadjusted number of admissions per year was 75,000 for the ABF group and 175,000 for the non-ABF group”. 2. If standardized or weighted outcomes are reported, we will consider this an adjusted analysis and record all variables for which outcomes are standardized/weighted. |
| 46a. What appropriate factors did the volume of care analysis adjust for? | 1. Select the most appropriate factor from the list for each adjustment variable reported in the article. Only select one adjustment factor from the list for each adjustor in the paper. 2. If the analysis adjusted for a variable not on the list, select other and notify Taryn immediately by email. Include in your email a description of the variable and if the variable is an appropriate or inappropriate adjuster |
| 46b. What possibly appropriate factors did the volume of care analysis adjust for? | 1. Select the most appropriate factor from the list for each adjustment variable reported in the article. Only select one adjustment factor from the list for each adjustor in the paper. 2. If the analysis adjusted for a variable not on the list, select other and notify Taryn immediately by email. Include in your email a description of the variable and if the variable is an appropriate or inappropriate adjuster |
| 46c. What inappropriate factors did the volume of care analysis adjust for? | 1. Select the most appropriate factor from the list for each adjustment variable reported in the article. Only select one adjustment factor from the list for each adjustor in the paper. 2. If the analysis adjusted for a variable not on the list, select other and notify Taryn immediately by email. Include in your email a description of the variable and if the variable is an appropriate or inappropriate adjuster |
| 47. Are the factors adjusted for in the volume of care analysis comprehensive and appropriate? | 1. Rate as comprehensive adjustment unless clearly different distributions in ABF and non-ABF group, and the authors fail to adjust for that variable (e.g. More rural hospitals in the non-ABF group and more urban hospitals in a ABF group, requires an adjustment for rural/urban location) |
| Table 48: Volume of Care | **“Earliest No ABF data point (within 3 years of ABF implementation) (data point A)” column:**   1. Only record year (not month or day if provided). If only one year (e.g. data collected in 1984 only) record “1984” start and “1984” end. 2. If data are not available, leave blank 3. Example, if ABF was implemented in 1983, and data are provided for the years 1979, 1980 1981, and 1982; abstract data from 1980 and record 1980 as the start and end date. If ABF implementation is in 1983 and one data point is provided for 1981 and 1982 (i.e. data are aggregated), abstract data from 1981-1982 and record 1981 as the start date and 1982 as the end date. 4. *Numerical response*   **“Earliest ABF data point (Data point B)” column:**   1. Only record year (not month or day). If only one year (e.g. data collected in 1984 only) record “1984” start and “1984” end. 2. If data are not available, leave blank. 3. Example, if ABF is implemented in 1983, and hospital readmission data are provided for 1984, 1985, 1986, and 1987; abstract data for 1984 and record 1984 as the start and end date. If ABF implementation is in 1983, and one data point is provided for 1984 and 1985 (i.e. data are aggregated), abstract data from 1984-1985 and record 1984 as the start date and 1985 as the end date. 4. If data are only reported for one time period after ABF, do not complete this column. For example, if ABF was implemented in 1983 and only data for 1984 are reported, skip this row and report data in the “latest ABF point” row. 5. *Numerical response*   **“Latest ABF point (within 5 years of ABF implementation) (Data point C)” column:**   1. Only record year (not month or day if provided). If only one year (e.g. data collected in 1984 only) record “1984” start and “1984” end. 2. If data are not available, leave blank. 3. Example, if ABF was implemented in 1983, and hospital readmission data are reported in 1984, 1985, 1986, 1987, 1988, and 1989; abstract the data reported in 1988 and record 1988 as the start and end date. If ABF was implemented in 1983, and one data point is provided for 1987 and 1988 (i.e. data are aggregated), abstract data from 1987-1988 and record 1987 as the start date and 1988 as the end date. 4. *Numerical response*   **Percent ABF**   1. Enter the ABF percentage for the ABF group 2. If a range is not reported and instead only a single percentage point is reported, complete only the “lower range text field.” 3. If percent not included, leave blank (except for US Medicare studies).   For US Medicare studies only, assume these dates:  Medicare Phase-In Period  1. Beginning on or after October 1, 1983 and before October 1, 1984: ABF=25%  2. Beginning on or after October 1, 1984 and before October 1, 1985: ABF=50%  3. Beginning on or after October 1, 1985 and before October 1, 1986: ABF=75%  4. Beginning on or after October 1, 1986: ABF=100%   1. *Numerical response*   **“Operational Variable” column:**   1. Name the specific variable, the units in which it is measured, and the time period over which it is measured (e.g. number of hospital readmissions within 90 days of discharge). 2. *Free text*   ****Note:** if more than one relevant outcome variable is reported for one of the listed variables, record data for both variables and use numbering to indicate what data goes with variable in the free text boxes (e.g. 1. Age (year); 2. Sex)  **“Summary of results” column:**   1. Abstractors will record key data as well as summary conclusions supported by key data. Summary conclusion should be a single sentence when possible. 2. We are interested in the comparison between data points labelled A vs. B and A vs. C (not B vs. C). 3. If the difference is not significant, still record the actual data numbers (e.g. percentage of readmission 33% in ABF vs. 35% in non-ABF (p=.06)). 4. If uncertain of what to abstract, consult one of the senior team members (likely Karen or Gordon or Danielle, the choice depending on the nature of the uncertainty). 5. Important to ensure that we are reporting data objectively, not inferences made by ourselves or the author(s). 6. When the author(s) provide only a narrative summary or commentary of their interpretation of data but do not provide data on which these inferences are based, do not include in results. 7. Free text   ****Note:** if more than one relevant outcome variable is reported for one of the listed variables, record data for both variables and use numbering to indicate what data goes with variable in the free text boxes (e.g. 1. mean age 66 in ABF vs. 68 in non-ABF (p=.06); 2. 1% more males in the ABF group (p = 0.80)).  **Difference between** **the 0% ABF vs.** **early ABF periods**   1. Answer below table in question 48 i   **Difference between** **the 0% ABF vs.** **later ABF periods**   1. Answer below table in question 48 ii |
| 48 i. **“Difference between the 0% ABF vs. early ABF periods”:** | 1. If a comparison between 0% and early ABF periods was not made select “comparison not made.” 2. If the comparison is reported in the article as not significant, select “no difference.” If the comparison is reported to be significant or no significance test was performed, the reviewer should record the direction of the effect and calculate the percentage change. 3. Use this link to calculate percentage change (enter the no ABF number first and the ABF number second): [http://www.csgnetwork.com/percentchangecalc.html](http://www.csgnetwork.com/percentchangecalc.html) 4. OR, The formula to calculate percent change is as follows: time 2-time 1/time 1 = x (100)= % change between time 1 and time 2   *(time 2= earlier ABF period and/ or later ABF period; time 1= 0% ABF period)  *e.g. if death rate was 2% before ABF, and 4% after ABF, then 4-2/2 = 1 (100)=100% difference  ****Note:** if more than one relevant outcome variable are reported in the above table check all that apply. |
| 48 ii. **“Difference between the 0% ABF vs. later ABF periods”:** | 1. If a comparison between 0% and late ABF periods was not made select “comparison not made.” 2. If the comparison is reported in the article as not significant, select “no difference.” If the comparison is reported to be significant or no significance test was performed, the reviewer should record the direction of the effect and calculate the percentage change. 3. Use this link to calculate percentage change (enter the no ABF number first and the ABF number second): [http://www.csgnetwork.com/percentchangecalc.html](http://www.csgnetwork.com/percentchangecalc.html) 4. OR, The formula to calculate percent change is as follows: time 2-time 1/time 1 = x (100)= % change between time 1 and time 2   *(time 2= earlier ABF period and/ or later ABF period; time 1= 0% ABF period)  *e.g. if death rate was 2% before ABF, and 4% after ABF, then 4-2/2 = 1 (100)=100% difference  ****Note:** if more than one relevant outcome variable are reported in the above table check all that apply. |
| 53. Does the study report differences in severity of illness/casemix? | 1. Examples of casemix variables include: 2. Diagnostic codes (e.g. ICD codes, DRG point produced) 3. comorbidities 4. For a comprehensive definition of casemix see the following link: [http://askalex.stanford.edu/archives/2010/12/what-is-the-hos.html](http://askalex.stanford.edu/archives/2010/12/what-is-the-hos.html) |
| 54. Can data from an unadjusted analysis for differences in severity of illness/casemix be abstracted? | 1. If more than one analysis is presented, abstract data from the unadjusted analysis. If only adjusted analyses were performed, abstract data from the less adjusted analysis. 2. If standardized or weighted outcomes are reported, we will consider this an adjusted analysis and record all variables for which outcomes are standardized/weighted. |
| 54a. What appropriate factors did the severity of illness/casemix adjust for? | 1. Select the most appropriate factor from the list for each adjustment variable reported in the article. Only select one adjustment factor from the list for each adjustor in the paper. 2. If the analysis adjusted for a variable not on the list, select other and notify Taryn immediately by email. Include in your email a description of the variable and if the variable is an appropriate or inappropriate adjuster |
| 54b. What inappropriate factors did the severity of illness/casemix analysis adjust for? | 1. Select the most appropriate factor from the list for each adjustment variable reported in the article. Only select one adjustment factor from the list for each adjustor in the paper. 2. If the analysis adjusted for a variable not on the list, select other and notify Taryn immediately by email. Include in your email a description of the variable and if the variable is an appropriate or inappropriate adjuster |
| 55. Are the factors adjusted for in the severity of illness/casemix analysis comprehensive and appropriate? | 1. There are no potentially appropriate adjustors that are crucial to adjust for. Analyses for these variables will usually come from descriptive tables without any adjustment. |
| Table 56: severity of illness/casemix | **“Earliest No ABF data point (within 3 years of ABF implementation) (data point A)” column:**   1. Only record year (not month or day if provided). If only one year (e.g. data collected in 1984 only) record “1984” start and “1984” end. 2. If data are not available, leave blank 3. Example, if ABF was implemented in 1983, and data are provided for the years 1979, 1980 1981, and 1982; abstract data from 1980 and record 1980 as the start and end date. If ABF implementation is in 1983 and one data point is provided for 1981 and 1982 (i.e. data are aggregated), abstract data from 1981-1982 and record 1981 as the start date and 1982 as the end date. 4. *Numerical response*   **“Earliest ABF data point (Data point B)” column:**   1. Only record year (not month or day). If only one year (e.g. data collected in 1984 only) record “1984” start and “1984” end. 2. If data are not available, leave blank. 3. Example, if ABF is implemented in 1983, and hospital readmission data are provided for 1984, 1985, 1986, and 1987; abstract data for 1984 and record 1984 as the start and end date. If ABF implementation is in 1983, and one data point is provided for 1984 and 1985 (i.e. data are aggregated), abstract data from 1984-1985 and record 1984 as the start date and 1985 as the end date. 4. If data are only reported for one time period after ABF, do not complete this column. For example, if ABF was implemented in 1983 and only data for 1984 are reported, skip this row and report data in the “latest ABF point” row. 5. *Numerical response*   **“Latest ABF point (within 5 years of ABF implementation) (Data point C)” column:**   1. Only record year (not month or day if provided). If only one year (e.g. data collected in 1984 only) record “1984” start and “1984” end. 2. If data are not available, leave blank. 3. Example, if ABF was implemented in 1983, and hospital readmission data are reported in 1984, 1985, 1986, 1987, 1988, and 1989; abstract the data reported in 1988 and record 1988 as the start and end date. If ABF was implemented in 1983, and one data point is provided for 1987 and 1988 (i.e. data are aggregated), abstract data from 1987-1988 and record 1987 as the start date and 1988 as the end date. 4. *Numerical response*   **Percent ABF**   1. Enter the ABF percentage for the ABF group 2. If a range is not reported and instead only a single percentage point is reported, complete only the “lower range text field.” 3. If percent not included, leave blank (except for US Medicare studies).   For US Medicare studies only, assume these dates:  Medicare Phase-In Period  1. Beginning on or after October 1, 1983 and before October 1, 1984: ABF=25%  2. Beginning on or after October 1, 1984 and before October 1, 1985: ABF=50%  3. Beginning on or after October 1, 1985 and before October 1, 1986: ABF=75%  4. Beginning on or after October 1, 1986: ABF=100%   1. *Numerical response*   **“Operational Variable” column:**   1. Name the specific variable, the units in which it is measured, and the time period over which it is measured (e.g. number of hospital readmissions within 90 days of discharge). 2. *Free text*   ****Note:** if more than one relevant outcome variable is reported for one of the listed variables, record data for both variables and use numbering to indicate what data goes with variable in the free text boxes (e.g. 1. Age (year); 2. Sex)  **“Summary of results” column:**   1. Abstractors will record key data as well as summary conclusions supported by key data. Summary conclusion should be a single sentence when possible. 2. We are interested in the comparison between data points labelled A vs. B and A vs. C (not B vs. C). 3. If the difference is not significant, still record the actual data numbers (e.g. percentage of readmission 33% in ABF vs. 35% in non-ABF (p=.06)). 4. If uncertain of what to abstract, consult one of the senior team members (likely Karen or Gordon or Danielle, the choice depending on the nature of the uncertainty). 5. Important to ensure that we are reporting data objectively, not inferences made by ourselves or the author(s). 6. When the author(s) provide only a narrative summary or commentary of their interpretation of data but do not provide data on which these inferences are based, do not include in results. 7. Free text   ****Note:** if more than one relevant outcome variable is reported for one of the listed variables, record data for both variables and use numbering to indicate what data goes with variable in the free text boxes (e.g. 1. mean age 66 in ABF vs. 68 in non-ABF (p=.06); 2. 1% more males in the ABF group (p = 0.80)).  **Difference between** **the 0% ABF vs.** **early ABF periods**   1. Answer below table in question 56 i   **Difference between** **the 0% ABF vs.** **later ABF periods**   1. Answer below table in question 56 i |
| 56 i. **“Difference between the 0% ABF vs. early ABF periods”:** | 1. If a comparison between 0% and early ABF periods was not made select “comparison not made.” 2. If the comparison is reported in the article as not significant, select “no difference.” If the comparison is reported to be significant or no significance test was performed, the reviewer should record the direction of the effect and calculate the percentage change. 3. Use this link to calculate percentage change (enter the no ABF number first and the ABF number second): [http://www.csgnetwork.com/percentchangecalc.html](http://www.csgnetwork.com/percentchangecalc.html) 4. OR, The formula to calculate percent change is as follows: time 2-time 1/time 1 = x (100)= % change between time 1 and time 2   *(time 2= earlier ABF period and/ or later ABF period; time 1= 0% ABF period)  *e.g. if death rate was 2% before ABF, and 4% after ABF, then 4-2/2 = 1 (100)=100% difference  ****Note:** if more than one relevant outcome variable are reported in the above table check all that apply. |
| 56 ii. **“Difference between the 0% ABF vs. later ABF periods”:** | 1. If a comparison between 0% and late ABF periods was not made select “comparison not made.” 2. If the comparison is reported in the article as not significant, select “no difference.” If the comparison is reported to be significant or no significance test was performed, the reviewer should record the direction of the effect and calculate the percentage change. 3. Use this link to calculate percentage change (enter the no ABF number first and the ABF number second): [http://www.csgnetwork.com/percentchangecalc.html](http://www.csgnetwork.com/percentchangecalc.html) 4. OR, The formula to calculate percent change is as follows: time 2-time 1/time 1 = x (100)= % change between time 1 and time 2   *(time 2= earlier ABF period and/ or later ABF period; time 1= 0% ABF period)  *e.g. if death rate was 2% before ABF, and 4% after ABF, then 4-2/2 = 1 (100)=100% difference  ****Note:** if more than one relevant outcome variable are reported in the above table check all that apply. |
| 61. Does the study report differences in volume of care? | 1. E.g. number of admissions, number of patients treated |
| 62. Can data from an adjusted analysis for volume of care be abstracted? | 1. If more than one analysis is presented: Answer “yes” to question 62. In the table columns “operational variable” and summary of results” record both adjusted and unadjusted analysis indicating which data are which using numbers. E.g. For operational variable record: “(1) adjusted number of admissions per year; (2) unadjusted number of admissions per year”. For summary of results record: “(1) the adjusted number of admissions per year was 100,000 for the ABF group and 50,000 for the non-ABF group; (2) the unadjusted number of admissions per year was 75,000 for the ABF group and 175,000 for the non-ABF group”. 2. If standardized or weighted outcomes are reported, we will consider this an adjusted analysis and record all variables for which outcomes are standardized/weighted. |
| 62a. What appropriate factors did the volume of care analysis adjust for? | 1. Select the most appropriate factor from the list for each adjustment variable reported in the article. Only select one adjustment factor from the list for each adjustor in the paper. 2. If the analysis adjusted for a variable not on the list, select other and notify Taryn immediately by email. Include in your email a description of the variable and if the variable is an appropriate or inappropriate adjuster |
| 62b. What possibly appropriate factors did the volume of care analysis adjust for? | 1. Select the most appropriate factor from the list for each adjustment variable reported in the article. Only select one adjustment factor from the list for each adjustor in the paper. 2. If the analysis adjusted for a variable not on the list, select other and notify Taryn immediately by email. Include in your email a description of the variable and if the variable is an appropriate or inappropriate adjuster |
| 62c. What inappropriate factors did the volume of care analysis adjust for? | 1. Select the most appropriate factor from the list for each adjustment variable reported in the article. Only select one adjustment factor from the list for each adjustor in the paper. 2. If the analysis adjusted for a variable not on the list, select other and notify Taryn immediately by email. Include in your email a description of the variable and if the variable is an appropriate or inappropriate adjuster |
| 63. Are the factors adjusted for in the volume of care analysis comprehensive and appropriate? | 1. Rate as comprehensive adjustment unless clearly different distributions in ABF and non-ABF group, and the authors fail to adjust for that variable (e.g. More rural hospitals in the non-ABF group and more urban hospitals in a ABF group, requires an adjustment for rural/urban location) |
| Table 63: Volume of Care | **“Start and End year of data point collection”:**   1. Only record year (not month or day). If only one year (e.g. data collected in 1984 only) record “1984” start and “1984” end. 2. If data are not available leave blank 3. *Numeric response*   **Percent ABF**   1. Enter the ABF percentage for the ABF group 2. If a range is not reported and instead only a single percentage point is reported, complete only the “lower range text field.” 3. If percent not included, leave blank (except for US Medicare studies).   For US Medicare studies only, assume these dates:  Medicare Phase-In Period  1. Beginning on or after October 1, 1983 and before October 1, 1984: ABF=25%  2. Beginning on or after October 1, 1984 and before October 1, 1985: ABF=50%  3. Beginning on or after October 1, 1985 and before October 1, 1986: ABF=75%  4. Beginning on or after October 1, 1986: ABF=100%   1. *Numerical response*   **“Operational Variable” column:**   1. Name the specific variable, the units in which it is measured, and the time period over which it is measured (e.g. number of hospital readmissions within 90 days of discharge). 2. *Free text*   ****Note:** if more than one relevant outcome variable is reported for one of the listed variables, record data for both variables and use numbering to indicate what data goes with variable in the free text boxes (e.g. 1. Age (year); 2. Sex)  **“Summary of results” column:**   1. Abstractors will record key data as well as summary conclusions supported by key data. Summary conclusion should be a single sentence when possible. 2. If the difference is not significant, still record the actual data numbers (e.g. percentage of readmission 33% in ABF vs. 35% in non-ABF (p=.06)). 3. If uncertain of what to abstract, consult one of the senior team members (likely Karen or Gordon or Danielle, the choice depending on the nature of the uncertainty). 4. Important to ensure that we are reporting data objectively, not inferences made by ourselves or the author(s). 5. When the author(s) provide only a narrative summary or commentary of their interpretation of data but do not provide data on which these inferences are based, do not include in results. 6. *Free text*   ****Note:** if more than one relevant outcome variable is reported for one of the listed variables, record data for both variables and use numbering to indicate what data goes with variable in the free text boxes (e.g. 1. mean age 66 in ABF vs. 68 in non-ABF (p=.06); 2. 1% more males in the ABF group (p = 0.80)).  **Difference between the** **0% ABF vs. most ABF** **jurisdiction**   1. Answer below in question 63 i |
| 63 i. **“Difference between the 0% ABF vs. most ABF jurisdictions”:** | 1. If a comparison between 0% and most ABF jurisdiction was not made, select “comparison not made.” 2. If the comparison is reported in the article as not significant, select “no difference.” If the comparison is reported to be significant or no significance test was performed, the reviewer should record the direction of the effect and calculate the percentage change. 3. Use this link to calculate percentage change (enter the no ABF number first and the ABF number second): [http://www.csgnetwork.com/percentchangecalc.html](http://www.csgnetwork.com/percentchangecalc.html) 4. OR, The formula to calculate percent change is as follows: time 2-time 1/time 1 = x (100)= % change between time 1 and time 2   *(Where time 2=ABF group and time 1= 0% ABF group)  *e.g. if death rate was 2% in the non-ABF, and 4% in the ABF, then 4-2/2 =1 (100)=100% difference  ****Note:** if more than one relevant outcome variable are reported in the above table check all that apply |
| 69. Does the study report differences severity of illness/casemix? | 1. Examples of casemix variables include: 2. Diagnostic codes (e.g. ICD codes, DRG point produced) 3. comorbidities 4. For a comprehensive definition of casemix see the following link: [http://askalex.stanford.edu/archives/2010/12/what-is-the-hos.html](http://askalex.stanford.edu/archives/2010/12/what-is-the-hos.html) |
| 70. Can data from an unadjusted analysis for difference in characteristics of study population be abstracted? | 1. If more than one analysis is presented, abstract data from the unadjusted analysis. If only adjusted analyses were performed, abstract data from the less adjusted analysis. 2. If standardized or weighted outcomes are reported, we will consider this an adjusted analysis and record all variables for which outcomes are standardized/weighted. |
| 70a. What appropriate factors did the severity of illness/casemix adjust for? | 1. Select the most appropriate factor from the list for each adjustment variable reported in the article. Only select one adjustment factor from the list for each adjustor in the paper. 2. If the analysis adjusted for a variable not on the list, select other and notify Taryn immediately by email. Include in your email a description of the variable and if the variable is an appropriate or inappropriate adjuster |
| 70b. What inappropriate factors did the severity of illness/casemix analysis adjust for? | 1. Select the most appropriate factor from the list for each adjustment variable reported in the article. Only select one adjustment factor from the list for each adjustor in the paper. 2. If the analysis adjusted for a variable not on the list, select other and notify Taryn immediately by email. Include in your email a description of the variable and if the variable is an appropriate or inappropriate adjuster |
| 71. Are the factors adjusted for in the severity of illness/casemix analysis comprehensive and appropriate? | 1. There are no potentially appropriate adjustors that are crucial to adjust for. Analyses for these variables will usually come from descriptive tables without any adjustment. |
| Table 72: severity of illness/casemix | **“Start and End year of data point collection”:**   1. Only record year (not month or day). If only one year (e.g. data collected in 1984 only) record “1984” start and “1984” end. 2. If data are not available leave blank 3. *Numeric response*   **Percent ABF**   1. Enter the ABF percentage for the ABF group 2. If a range is not reported and instead only a single percentage point is reported, complete only the “lower range text field.” 3. If percent not included, leave blank (except for US Medicare studies).   For US Medicare studies only, assume these dates:  Medicare Phase-In Period  1. Beginning on or after October 1, 1983 and before October 1, 1984: ABF=25%  2. Beginning on or after October 1, 1984 and before October 1, 1985: ABF=50%  3. Beginning on or after October 1, 1985 and before October 1, 1986: ABF=75%  4. Beginning on or after October 1, 1986: ABF=100%   1. *Numerical response*   **“Operational Variable” column:**   1. Name the specific variable, the units in which it is measured, and the time period over which it is measured (e.g. number of hospital readmissions within 90 days of discharge). 2. *Free text*   ****Note:** if more than one relevant outcome variable is reported for one of the listed variables, record data for both variables and use numbering to indicate what data goes with variable in the free text boxes (e.g. 1. Age (year); 2. Sex)  **“Summary of results” column:**   1. Abstractors will record key data as well as summary conclusions supported by key data. Summary conclusion should be a single sentence when possible. 2. If the difference is not significant, still record the actual data numbers (e.g. percentage of readmission 33% in ABF vs. 35% in non-ABF (p=.06)). 3. If uncertain of what to abstract, consult one of the senior team members (likely Karen or Gordon or Danielle, the choice depending on the nature of the uncertainty). 4. Important to ensure that we are reporting data objectively, not inferences made by ourselves or the author(s). 5. When the author(s) provide only a narrative summary or commentary of their interpretation of data but do not provide data on which these inferences are based, do not include in results. 6. *Free text*   ****Note:** if more than one relevant outcome variable is reported for one of the listed variables, record data for both variables and use numbering to indicate what data goes with variable in the free text boxes (e.g. 1. mean age 66 in ABF vs. 68 in non-ABF (p=.06); 2. 1% more males in the ABF group (p = 0.80)).  **Difference between the** **0% ABF vs. most ABF** **jurisdiction**   1. Answer below in question 72 i |
| 72 i. **“Difference between the 0% ABF vs. most ABF jurisdictions”:** | 1. If a comparison between 0% and most ABF jurisdiction was not made, select “comparison not made.” 2. If the comparison is reported in the article as not significant, select “no difference.” If the comparison is reported to be significant or no significance test was performed, the reviewer should record the direction of the effect and calculate the percentage change. 3. Use this link to calculate percentage change (enter the no ABF number first and the ABF number second): [http://www.csgnetwork.com/percentchangecalc.html](http://www.csgnetwork.com/percentchangecalc.html) 4. OR, The formula to calculate percent change is as follows: time 2-time 1/time 1 = x (100)= % change between time 1 and time 2   *(Where time 2=ABF group and time 1= 0% ABF group)  *e.g. if death rate was 2% in the non-ABF, and 4% in the ABF, then 4-2/2 =1 (100)=100% difference  ****Note:** if more than one relevant outcome variable are reported in the above table check all that apply |
| 77. Does the study report differences in volume of care? | 1. E.g. number of admissions, number of patients treated |
| 78. Can data from an adjusted analysis for volume of care be abstracted? | 1. If more than one analysis is presented: Answer “yes” to question 78. In the table columns “operational variable” and summary of results” record both adjusted and unadjusted analysis indicating which data are which using numbers. E.g. For operational variable record: “(1) adjusted number of admissions per year; (2) unadjusted number of admissions per year”. For summary of results record: “(1) the adjusted number of admissions per year was 100,000 for the ABF group and 50,000 for the non-ABF group; (2) the unadjusted number of admissions per year was 75,000 for the ABF group and 175,000 for the non-ABF group”. 2. If standardized or weighted outcomes are reported, we will consider this an adjusted analysis and record all variables for which outcomes are standardized/weighted. |
| 78a. What appropriate factors did the volume of care analysis adjust for? | 1. Select the most appropriate factor from the list for each adjustment variable reported in the article. Only select one adjustment factor from the list for each adjustor in the paper. 2. If the analysis adjusted for a variable not on the list, select other and notify Taryn immediately by email. Include in your email a description of the variable and if the variable is an appropriate or inappropriate adjuster |
| 78b. What possibly appropriate factors did the volume of care analysis adjust for? | 1. Select the most appropriate factor from the list for each adjustment variable reported in the article. Only select one adjustment factor from the list for each adjustor in the paper. 2. If the analysis adjusted for a variable not on the list, select other and notify Taryn immediately by email. Include in your email a description of the variable and if the variable is an appropriate or inappropriate adjuster |
| 78c. What inappropriate factors did the volume of care analysis adjust for? | 1. Select the most appropriate factor from the list for each adjustment variable reported in the article. Only select one adjustment factor from the list for each adjustor in the paper. 2. If the analysis adjusted for a variable not on the list, select other and notify Taryn immediately by email. Include in your email a description of the variable and if the variable is an appropriate or inappropriate adjuster |
| 79. Are the factors adjusted for in the volume of care analysis comprehensive and appropriate? | 1. Rate as comprehensive adjustment unless clearly different distributions in ABF and non-ABF group, and the authors fail to adjust for that variable (e.g. More rural hospitals in the non-ABF group and more urban hospitals in a ABF group, requires an adjustment for rural/urban location) |
| Table 80: volume of care | **“Earliest No ABF data point (within 3 years of ABF implementation) (data point A)” column:**   1. Only record year (not month or day if provided). If only one year (e.g. data collected in 1984 only) record “1984” start and “1984” end. 2. If data are not available, leave blank 3. Example, if ABF was implemented in 1983, and data are provided for the years 1979, 1980 1981, and 1982; abstract data from 1980 and record 1980 as the start and end date. If ABF implementation is in 1983 and one data point is provided for 1981 and 1982 (i.e. data are aggregated), abstract data from 1981-1982 and record 1981 as the start date and 1982 as the end date. 4. *Numerical response*   **“Earliest ABF data point (Data point B)” column:**   1. Only record year (not month or day). If only one year (e.g. data collected in 1984 only) record “1984” start and “1984” end. 2. If data are not available, leave blank. 3. Example, if ABF is implemented in 1983, and hospital readmission data are provided for 1984, 1985, 1986, and 1987; abstract data for 1984 and record 1984 as the start and end date. If ABF implementation is in 1983, and one data point is provided for 1984 and 1985 (i.e. data are aggregated), abstract data from 1984-1985 and record 1984 as the start date and 1985 as the end date. 4. If data are only reported for one time period after ABF, do not complete this column. For example, if ABF was implemented in 1983 and only data for 1984 are reported, skip this row and report data in the “latest ABF point” row. 5. *Numerical response*   **“Latest ABF point (within 5 years of ABF implementation) (Data point C)” column:**   1. Only record year (not month or day if provided). If only one year (e.g. data collected in 1984 only) record “1984” start and “1984” end. 2. If data are not available, leave blank. 3. Example, if ABF was implemented in 1983, and hospital readmission data are reported in 1984, 1985, 1986, 1987, 1988, and 1989; abstract the data reported in 1988 and record 1988 as the start and end date. If ABF was implemented in 1983, and one data point is provided for 1987 and 1988 (i.e. data are aggregated), abstract data from 1987-1988 and record 1987 as the start date and 1988 as the end date. 4. *Numerical response*   **Percent ABF**   1. Enter the ABF percentage for the ABF group 2. If a range is not reported and instead only a single percentage point is reported, complete only the “lower range text field.” 3. If percent not included, leave blank (except for US Medicare studies).   For US Medicare studies only, assume these dates:  Medicare Phase-In Period  1. Beginning on or after October 1, 1983 and before October 1, 1984: ABF=25%  2. Beginning on or after October 1, 1984 and before October 1, 1985: ABF=50%  3. Beginning on or after October 1, 1985 and before October 1, 1986: ABF=75%  4. Beginning on or after October 1, 1986: ABF=100%   1. *Numerical response*   **“Operational Variable” column:**   1. Name the specific variable, the units in which it is measured, and the time period over which it is measured (e.g. number of hospital readmissions within 90 days of discharge). 2. *Free text*   ****Note:** if more than one relevant outcome variable is reported for one of the listed variables, record data for both variables and use numbering to indicate what data goes with variable in the free text boxes (e.g. 1. Age (year); 2. Sex)  **“Summary of results” column:**   1. Abstractors will record key data as well as summary conclusions supported by key data. Summary conclusion should be a single sentence when possible. 2. We are interested in the comparison between data points labelled A vs. B and A vs. C (not B vs. C) in the ABF vs. no ABF jurisdictions. 3. If the difference is not significant, still record the actual data numbers (e.g. percentage of readmission 33% in ABF vs. 35% in non-ABF (p=.06)). 4. If uncertain of what to abstract, consult one of the senior team members (likely Karen or Gordon or Danielle, the choice depending on the nature of the uncertainty). 5. Important to ensure that we are reporting data objectively, not inferences made by ourselves or the author(s). 6. When the author(s) provide only a narrative summary or commentary of their interpretation of data but do not provide data on which these inferences are based, do not include in results. 7. *Free text*   ****Note:** if more than one relevant outcome variable is reported for one of the listed variables, record data for both variables and use numbering to indicate what data goes with variable in the free text boxes (e.g. 1. mean age 66 in ABF vs. 68 in non-ABF (p=.06); 2. 1% more males in the ABF group (p = 0.80)).    **Difference between** **A vs. B** **in no ABF** **jurisdiction  VS** **A vs B in ABF** **jurisdiction**   1. Answer below in question 80 i   **Difference between** **A vs. C** **in no ABF** **jurisdiction VS** **A vs C in ABF** **jurisdiction**   1. Answer below in question 80 ii |
| 80 i. **“Difference between the 0% ABF vs. early ABF periods”:** | 1. If a comparison between 0% and early ABF periods was not made in the ABF and no ABF jurisdictions select “comparison not made” 2. If the comparison is reported in the article as not significant, select “no difference”. If the comparison is reported to be significant, or no significance test was performed, the reviewer should record the direction of the effect and calculate the percentage change. 3. Use this link to calculate percentage change (enter the no ABF number first and the ABF number second): [http://www.csgnetwork.com/percentchangecalc.html](http://www.csgnetwork.com/percentchangecalc.html) 4. The formula is as follows:   time 2-time 1 in the 0% ABF periods = A  time 2 - time 1 in the ABF periods = B  then formula is B - A/A =x (100)  ****Note:** if more than one relevant outcome variable are reported in the above table check all that apply |
| 80 ii. **“Difference between the 0% ABF vs. later ABF periods”:** | 1. If a comparison between 0% and later ABF periods was not made in the ABF and no ABF jurisdictions select “comparison not made” 2. If the comparison is reported in the article as not significant, select “no difference”. If the comparison is reported to be significant, or no significance test was performed, the reviewer should record the direction of the effect and calculate the percentage change. 3. Use this link to calculate percentage change (enter the no ABF number first and the ABF number second): [http://www.csgnetwork.com/percentchangecalc.html](http://www.csgnetwork.com/percentchangecalc.html) 4. The formula is as follows:   time 2-time 1 in the 0% ABF periods = A  time 2 - time 1 in the ABF periods = B  then formula is B - A/A =x (100)  ****Note:** if more than one relevant outcome variable are reported in the above table check all that apply |
| 85. Does the study report differences severity of illness/casemix? | 1. Examples of casemix variables include: 2. Diagnostic codes (e.g. ICD codes, DRG point produced) 3. comorbidities 4. For a comprehensive definition of casemix see the following link: [http://askalex.stanford.edu/archives/2010/12/what-is-the-hos.html](http://askalex.stanford.edu/archives/2010/12/what-is-the-hos.html) |
| 86. Can data from an unadjusted analysis for difference in severity of illness/casemix be abstracted? | 1. If more than one analysis is presented, abstract data from the unadjusted analysis. If only adjusted analyses were performed, abstract data from the less adjusted analysis. 2. If standardized or weighted outcomes are reported, we will consider this an adjusted analysis and record all variables for which outcomes are standardized/weighted. |
| 86a. What appropriate factors did the severity of illness/casemix adjust for? | 1. Select the most appropriate factor from the list for each adjustment variable reported in the article. Only select one adjustment factor from the list for each adjustor in the paper. 2. If the analysis adjusted for a variable not on the list, select other and notify Taryn immediately by email. Include in your email a description of the variable and if the variable is an appropriate or inappropriate adjuster |
| 86b. What inappropriate factors did the severity of illness/casemix analysis adjust for? | 1. Select the most appropriate factor from the list for each adjustment variable reported in the article. Only select one adjustment factor from the list for each adjustor in the paper. 2. If the analysis adjusted for a variable not on the list, select other and notify Taryn immediately by email. Include in your email a description of the variable and if the variable is an appropriate or inappropriate adjuster |
| 87. Are the factors adjusted for in the severity of illness/casemix analysis comprehensive and appropriate? | 1. There are no potentially appropriate adjustors that are crucial to adjust for. Analyses for these variables will usually come from descriptive tables without any adjustment. |
| Table 88: severity of illness/casemix | **“Earliest No ABF data point (within 3 years of ABF implementation) (data point A)” column:**   1. Only record year (not month or day if provided). If only one year (e.g. data collected in 1984 only) record “1984” start and “1984” end. 2. If data are not available, leave blank 3. Example, if ABF was implemented in 1983, and data are provided for the years 1979, 1980 1981, and 1982; abstract data from 1980 and record 1980 as the start and end date. If ABF implementation is in 1983 and one data point is provided for 1981 and 1982 (i.e. data are aggregated), abstract data from 1981-1982 and record 1981 as the start date and 1982 as the end date. 4. *Numerical response*   **“Earliest ABF data point (Data point B)” column:**   1. Only record year (not month or day). If only one year (e.g. data collected in 1984 only) record “1984” start and “1984” end. 2. If data are not available, leave blank. 3. Example, if ABF is implemented in 1983, and hospital readmission data are provided for 1984, 1985, 1986, and 1987; abstract data for 1984 and record 1984 as the start and end date. If ABF implementation is in 1983, and one data point is provided for 1984 and 1985 (i.e. data are aggregated), abstract data from 1984-1985 and record 1984 as the start date and 1985 as the end date. 4. If data are only reported for one time period after ABF, do not complete this column. For example, if ABF was implemented in 1983 and only data for 1984 are reported, skip this row and report data in the “latest ABF point” row. 5. *Numerical response*   **“Latest ABF point (within 5 years of ABF implementation) (Data point C)” column:**   1. Only record year (not month or day if provided). If only one year (e.g. data collected in 1984 only) record “1984” start and “1984” end. 2. If data are not available, leave blank. 3. Example, if ABF was implemented in 1983, and hospital readmission data are reported in 1984, 1985, 1986, 1987, 1988, and 1989; abstract the data reported in 1988 and record 1988 as the start and end date. If ABF was implemented in 1983, and one data point is provided for 1987 and 1988 (i.e. data are aggregated), abstract data from 1987-1988 and record 1987 as the start date and 1988 as the end date. 4. *Numerical response*   **Percent ABF**   1. Enter the ABF percentage for the ABF group 2. If a range is not reported and instead only a single percentage point is reported, complete only the “lower range text field.” 3. If percent not included, leave blank (except for US Medicare studies).   For US Medicare studies only, assume these dates:  Medicare Phase-In Period  1. Beginning on or after October 1, 1983 and before October 1, 1984: ABF=25%  2. Beginning on or after October 1, 1984 and before October 1, 1985: ABF=50%  3. Beginning on or after October 1, 1985 and before October 1, 1986: ABF=75%  4. Beginning on or after October 1, 1986: ABF=100%   1. *Numerical response*   **“Operational Variable” column:**   1. Name the specific variable, the units in which it is measured, and the time period over which it is measured (e.g. number of hospital readmissions within 90 days of discharge). 2. *Free text*   ****Note:** if more than one relevant outcome variable is reported for one of the listed variables, record data for both variables and use numbering to indicate what data goes with variable in the free text boxes (e.g. 1. Age (year); 2. Sex)  **“Summary of results” column:**   1. Abstractors will record key data as well as summary conclusions supported by key data. Summary conclusion should be a single sentence when possible. 2. We are interested in the comparison between data points labelled A vs. B and A vs. C (not B vs. C) in the ABF vs. no ABF jurisdictions. 3. If the difference is not significant, still record the actual data numbers (e.g. percentage of readmission 33% in ABF vs. 35% in non-ABF (p=.06)). 4. If uncertain of what to abstract, consult one of the senior team members (likely Karen or Gordon or Danielle, the choice depending on the nature of the uncertainty). 5. Important to ensure that we are reporting data objectively, not inferences made by ourselves or the author(s). 6. When the author(s) provide only a narrative summary or commentary of their interpretation of data but do not provide data on which these inferences are based, do not include in results. 7. *Free text*   ****Note:** if more than one relevant outcome variable is reported for one of the listed variables, record data for both variables and use numbering to indicate what data goes with variable in the free text boxes (e.g. 1. mean age 66 in ABF vs. 68 in non-ABF (p=.06); 2. 1% more males in the ABF group (p = 0.80)).    **Difference between** **A vs. B** **in no ABF** **jurisdiction  VS** **A vs B in ABF** **jurisdiction**   1. Answer below in question 88 i   **Difference between** **A vs. C** **in no ABF** **jurisdiction VS** **A vs C in ABF** **jurisdiction**   1. Answer below in question 88 ii |
| 88 i. **“Difference between the 0% ABF vs. early ABF periods”:** | 1. If a comparison between 0% and early ABF periods was not made in the ABF and no ABF jurisdictions select “comparison not made” 2. If the comparison is reported in the article as not significant, select “no difference”. If the comparison is reported to be significant, or no significance test was performed, the reviewer should record the direction of the effect and calculate the percentage change. 3. Use this link to calculate percentage change (enter the no ABF number first and the ABF number second): [http://www.csgnetwork.com/percentchangecalc.html](http://www.csgnetwork.com/percentchangecalc.html) 4. The formula is as follows:   time 2-time 1 in the 0% ABF periods = A  time 2 - time 1 in the ABF periods = B  then formula is B - A/A =x (100)  ****Note:** if more than one relevant outcome variable are reported in the above table check all that apply |
| 88 ii. **Difference between the 0% ABF vs. later ABF periods”:** | 1. If a comparison between 0% and later ABF periods was not made in the ABF and no ABF jurisdictions select “comparison not made” 2. If the comparison is reported in the article as not significant, select “no difference”. If the comparison is reported to be significant, or no significance test was performed, the reviewer should record the direction of the effect and calculate the percentage change. 3. Use this link to calculate percentage change (enter the no ABF number first and the ABF number second): [http://www.csgnetwork.com/percentchangecalc.html](http://www.csgnetwork.com/percentchangecalc.html) 4. The formula is as follows:   time 2-time 1 in the 0% ABF periods = A  time 2 - time 1 in the ABF periods = B  then formula is B - A/A =x (100)  ****Note:** if more than one relevant outcome variable are reported in the above table check all that apply |

SECTION VI: CREDIBILITY ASSESSMENT

| **Credibility Question Number** | **Specific Instruction** |
| --- | --- |
| 89. Did authors use original data collection or, if using an administrative data base(s), did they document that their data source is of high quality? | 1. If the data are original OR if the data are from an administrative database that documented adequate quality measures (low error rates on the adjustment variables and outcomes in which we are interested), answer “yes”; 2. **Rule:** If the authors document anything about low error rates for any of the variables of interest answer “mostly yes”. If the author’s document any weaker measures of data quality (e.g. missing data), but do not document error rates, answer “mostly no”. 3. If none of these are documented, answer “no”. 4. **Rule:** If the data analyzed are from a previously conducted randomized controlled trial, select “Yes” |
| - - 1. Check all of the variables for which the study simultaneously examined the impact of ABF (check all that apply): |  |

**Figure 1:** Study Design Flow Chart


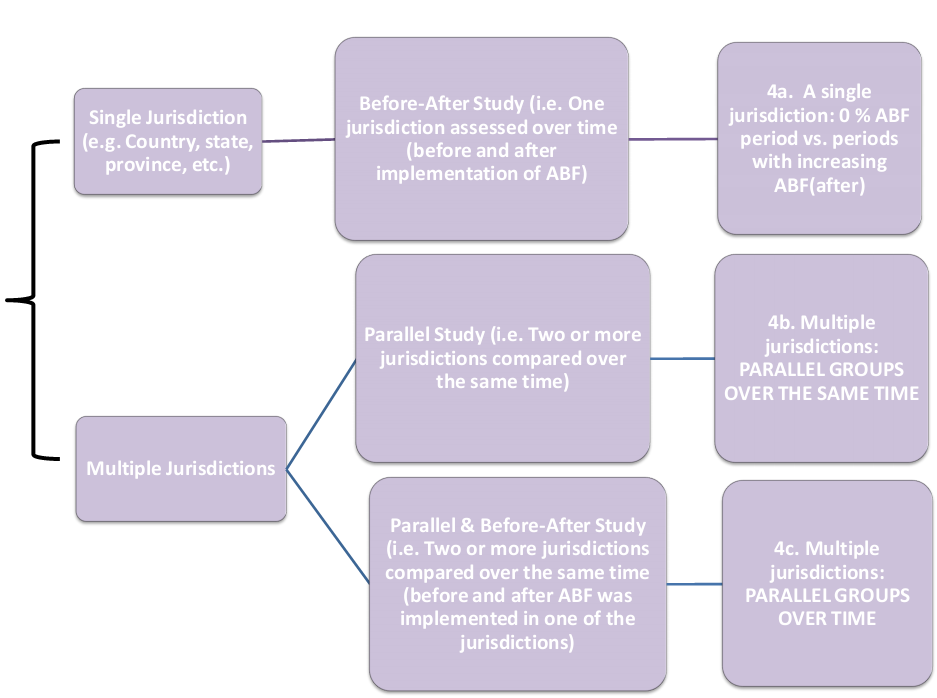


**Figure 2a:** Algorithm for determining whether to abstract poolable data


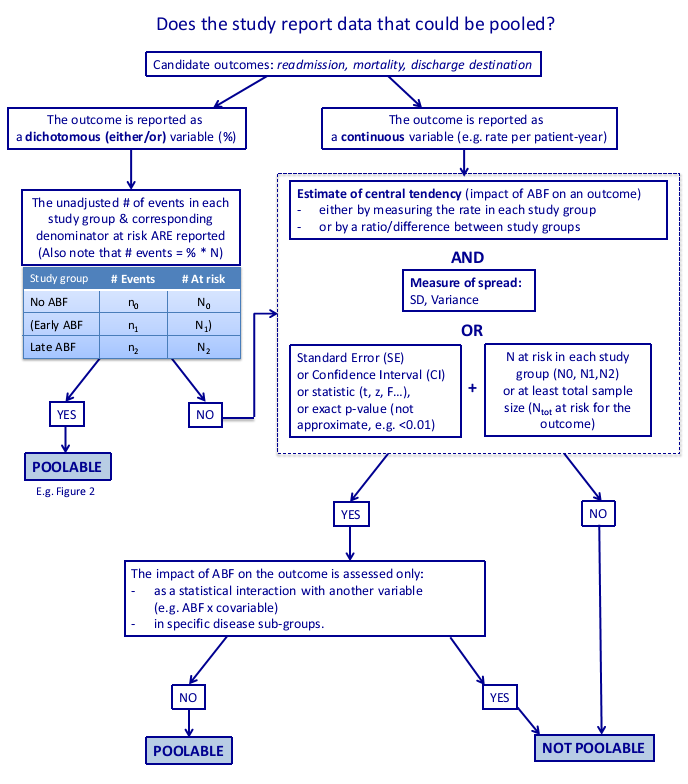


**E.g. Figure 2b**

**Figure 2b:** Example of potentially poolable dichotomous outcomes


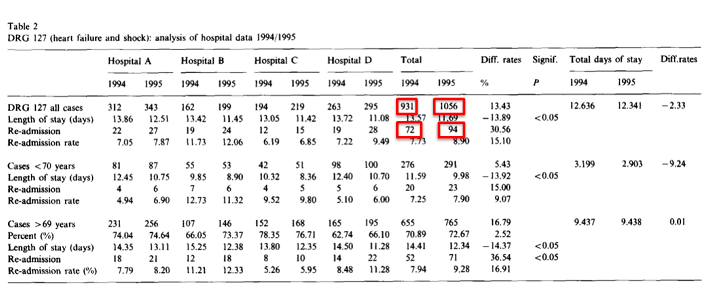


**Figure 3:** Algorithm for abstracting discharge destination data

**Yes**

**No**

**No**

**Yes**

**No**

**Yes**

**No**

**Yes**
